# Electrooxidation enables highly regioselective dearomative annulation of indole and benzofuran derivatives

**DOI:** 10.1038/s41467-019-13829-4

**Published:** 2020-01-07

**Authors:** Kun Liu, Wenxu Song, Yuqi Deng, Huiyue Yang, Chunlan Song, Takfaoui Abdelilah, Shengchun Wang, Hengjiang Cong, Shan Tang, Aiwen Lei

**Affiliations:** College of Chemistry and Molecular Sciences, Institute for Advanced Studies (IAS), Wuhan University, Wuhan, 430072 P. R. China

**Keywords:** Catalytic mechanisms, Electrochemistry, Synthetic chemistry methodology

## Abstract

The dearomatization of arenes represents a powerful synthetic methodology to provide three-dimensional chemicals of high added value. Here we report a general and practical protocol for regioselective dearomative annulation of indole and benzofuran derivatives in an electrochemical way. Under undivided electrolytic conditions, a series of highly functionalized five to eight-membered heterocycle-2,3-fused indolines and dihydrobenzofurans, which are typically unattainable under thermal conditions, can be successfully accessed in high yield with excellent regio- and stereo-selectivity. This transformation can also tolerate a wide range of functional groups and achieve good efficiency in large-scale synthesis under oxidant-free conditions. In addition, cyclic voltammetry, electron paramagnetic resonance (EPR) and kinetic studies indicate that the dehydrogenative dearomatization annulations arise from the anodic oxidation of indole into indole radical cation, and this process is the rate-determining step.

## Abstract (web summary)

Enriching the chemical space of polycyclic heterocycles is of high value in chemical biology. Here, the authors report an electrooxidative protocol for the regioselective dearomative annulation of indoles and benzofurans under undivided cell conditions and obtain five- to eight-membered fused heterocycles.

## Introduction

**Fig. 1:**
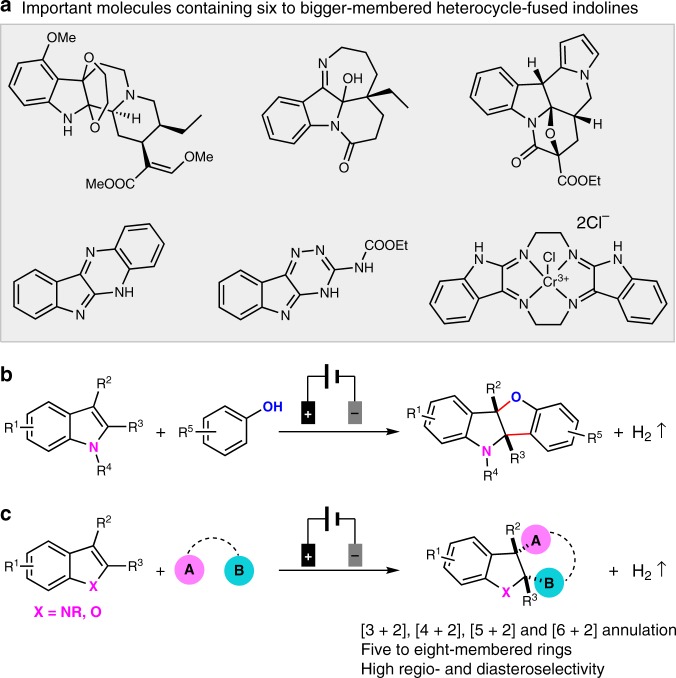
Electrooxidation enables dearomative annulation of indoles to synthesize polycyclic indoline derivatives. **a** Important molecules containing six to bigger-membered heterocycle-fused indolines. **b** Electrochemical [3 + 2] annulation between indole and phenol to access benzofuro[3,2-b]indolines. **c** Electrochemical [3 + 2], [4 + 2], [5 + 2], and [6 + 2] dearomative annulation of indoles and benzofurans with various bis-nucleophiles.

Breaking the aromatic systems of electron-rich arenes or heteroarenes provides three-dimensional chemicals of high added value^1–7^. In this field, polycyclic indoline-based alkaloids derived from indole dearomatization have promoted chemists to develop numerous methods for their efficient preparation owing to the important biological activities^8–11^. Over the past several decades, dearomative annulation of indoles has served as one of the most popular avenues for preparing polycyclic indoline skelotons^12–15^. Some powerful protocols like cyclopropanation^16,17^, 1,3-dipolar cycloadditions^18^, [2 + 2] photo-cycloadditions^19^, Diels-Alder^20–22^, and cascade electrophilic addition/annulation reactions^23–26^ have been well established. In these cases, most of the strategies took advantage of the C2–C3 π bond or the inherent strong nucleophilicity of C3 position, and had the advantage of rapid construction for highly functionalized indoline frameworks in one step. On the other hand, recent advances in oxidation-induced C-H functionalization have opened up other possibilities for constructing various polycyclic indolines^27–31^. In this regard, many efficient strategies for activating indoles have been developed under mild conditions^32–36^. In 2012, Ma and coworkers^37^ reported an attractive procedure to prepare polycyclic pyrroloindolines with iodine as oxidizing agents. In 2014, the group of Xiao developed a visible light-induced intramolecular oxyamidation reaction of indoles using molecular oxygen as the oxidant^38^. Obviously, significant progress has been made in the synthesis of five-membered ring-fused 2,3-indolines. As compared with this, these types of methodologies failed to provide a practically efficient route toward biologically valuable six to bigger-membered heterocycle-fused indolines (Fig. 1a)^39–44^. Therefore, it is highly appealing to develop efficient approaches to allow for their preparation.

Electrochemical oxidation offers a mild and efficient alternative to the use of hazardous chemical oxidants and demonstrates unique reaction selectivity compared with the results under thermal conditions^45–59^. With sufficient potential bias, organic substrates can lose one electron at the anode to generate highly reactive intermediates^60–63^. In 2017, our group developed an electrochemical dearomative [3 + 2] annulation between phenols and *N*-acetyl indoles for the synthesis of benzofuro[3,2-b]indolines (Fig. 1b)^64^. This dearomative annulation was proved to be proceeded through the radical/radical cross-coupling between in situ generated indole radical cations and phenol radicals, but only five-membered ring-fused 2,3-indolines were afforded. The approach toward dehydrogenative [4 + 2] dearomatization annulation of indoles has been rare. Fascinated by the distinctive reactivity of indole radical cation, we anticipate that the reports for adequately studied alkene radical cation may give a hand to the development of dehydrogenative [4 + 2] annulation of indoles^65,66^. For example, in 2018, our group has demonstrated a dehydrogenative [4 + 2] annulation of styrenes with alkynes to afford a variety of six-membered aromatic rings^67^. This reaction went through the nucleophilic attack of dienophile to alkene radical cation and second nucleophilic attack from arene to in situ generated alkenyl carbon cation to afford the cyclic intermediates. Therefore, we envision that introducing a reagent that incorporates seperate radical and nucleophilic centers or two nucleophilic centers to react with indoles radical cation may achieve the desired [4 + 2] dearomative annulation. Very recently, Vincent and coworkers reported an electrochemical dearomative dialkoxylation and diazidation of indoles with methanol or TMSN_3_, albeit only one example of intermolecular dearomatization annulation was reported in 43% yield with ethylene glycol^68^. Here we present a versatile protocol for efficient electrooxidative [4 + 2] dearomatization annulation between indoles and various bis-nucleophiles that contain O-, N-, and S-nucleophilic groups. This method can be extended to intermolecular [3 + 2], [5 + 2], and [6 + 2] annulations. Many biologically valuable five to eight-membered heterocycle-fused 2,3-indolines are constructed under external oxidant- and catalyst-free conditions, which are unattainable with thermal methods. Benzofurans can also serve as two-carbon synthon to afford dihydrobenzofuran. Notably, excellent regio- and stereo-selectivity are achieved in these transformations (Fig. 1c).

## Result

### Investigation of reaction conditions and substrate scope

To probe the feasibility of this strategy, we commenced the study on the reaction of 3-methyl *N*-acetyl indole (**1a**) with ethylene glycol (**2a**). To our delight, the desired dearomative [4 + 2] annulation product **3aa** was obtained in 75% yield under 10 mA constant current for 4 h in a simple undivided cell with carbon cloth as both anode and cathode. The structure of **3aa** was further confirmed by X-ray diffraction analysis. Adjusting the current seldom effected the reaction efficiency (Table 1, entry 2 and 3). Using electrolyte like ^n^Bu_4_NPF_6_ or ^n^Bu_4_NClO_4_ would to some extent decrease the yield (Table 1, entry 4 and 5). Decreasing the volume of ethylene glycol to 0.75 mL, 61% yield of product could still be obtained. If increasing to 2.0 mL, almost the same yield was afforded (Table 1, entry 6 and 7). The effect of electrode materials was also explored. Poor efficiency for the [4 + 2] annulation was obtained with carbon rod or carbon plate as anode (Table 1, entry 8 and 9). The major side reaction was the decomposition of 3-methyl *N*-acetyl indole and no indole dimerization product was detected, which indicated that the electrode with larger surface might enable better reaction efficiency and selectivity. With platinum plate cathode, moderate yield was gained (Table 1, entry 10). Replacing MeCN with DCM did not favor the [4 + 2] annulation and a majority part of indole disappeared after electrolysis (Table 1, entry 11). No reaction took place without electric current under air atmosphere accompanied by the residue of almost all of starting materials (Table 1, entry 12). Notably, the reaction could be conducted under air atmosphere with a good yield (Table 1, entry 13).

**Table 1:** Investigation of reaction conditions^a^.


Entry	Variation from standard conditions	Yield
1	None	75%
2	5 mA, 8 h	74%
3	20 mA, 2 h	73%
4	^*n*^Bu_4_NPF_6_ instead of ^*n*^Bu_4_NBF_4_	65%
5	^*n*^Bu_4_NClO_4_ instead of ^*n*^Bu_4_NBF_4_	43%
6	0.75 mL of **2a**	61%
7	2.0 mL of **2a**	77%
8	Carbon rod anode	7%
9	Carbon plate anode	12%
10	Pt plate cathode	55%
11	DCM instead of MeCN	n.d.
12	Without electric current, under air	n.r.
13	Conducted under air	73%

^a^Reaction conditions: carbon cloth anode (20 mm × 20 mm), carbon cloth cathode (20 mm × 20 mm), constant current = 10 mA (*J*_anode_ = 2.5 mA cm^−2^), **1a** (0.50 mmol), **2a** (1.2 mL), ^*n*^Bu_4_NBF_4_ (0.30 mmol), MeCN (9.0 mL), N_2_, r.t., 4 h (3.0F). Isolated yields were shown. *n.d.* not detected, *n.r.* no reaction.

### Scope of bis-nucleophiles

**Fig. 2:**
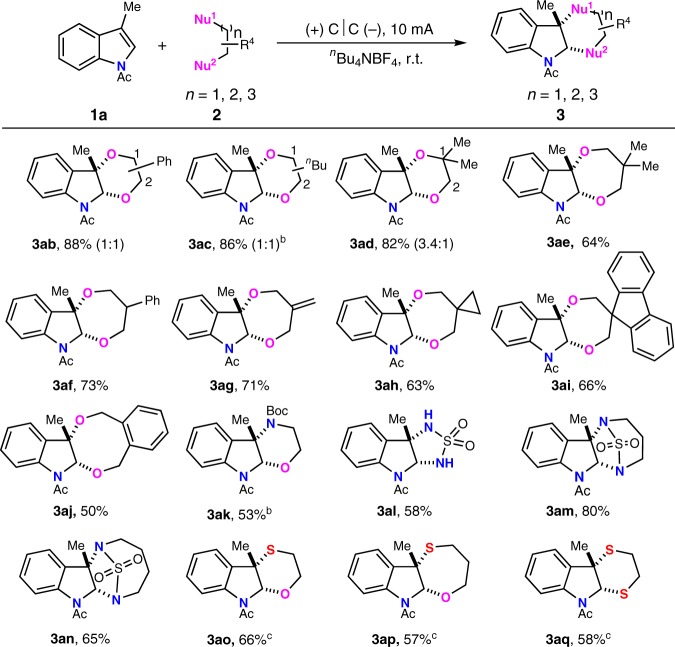
Scope of bis-nucleophiles. ^a^Reaction conditions: carbon cloth anode (20 mm × 20 mm), carbon cloth cathode (20 mm × 20 mm), constant current = 10 mA (*J*_anode_ = 2.5 mA cm^−2^), **1a** (0.50 mmol), **2** (1.2 mL with liquid glycols, 1.3 g with solid glycols, 4 eq. with sulfamide, 8 eq. with mercapto containing bis-nucleophiles), ^*n*^Bu_4_NBF_4_ (0.30 mmol), MeCN/DCM (5.0/4.0 mL), N_2_. ^b^MeCN (9.0 mL). ^c^15 mA (*J*_anode_ = 3.75 mA cm^−2^).

To demonstrate the applicability of this transformation, we first evaluated the applicability of various bis-nucleophiles (Fig. 2). With 1-phenylethane-1,2-diol and hexane-1,2-diol, two products were obtained with ratio of 1:1 (**3ab** and **3ac**) in enhanced yield. If ethylene glycol with bigger steric hindrance was applied, the reaction selectivity could be increased to 3.4:1 with 82% yield (**3ad**). Dearomative [4 + 3] annulations could also be achieved efficiently from indole and functionalized 1,3-diols (**3ae**–**3ag**). 1,3-Diols bearing another rings could afford the spirocyclic compounds in 63% and 66% yield, respectively (**3ah** and **3ai**). Moreover, eight-membered ring-fused 2,3-indoline (**3aj**) was obtained via [6 + 2] annulation from indole and 1,2-phenylenedimethanol in 50% yield. We then tested the possibility of forming C-N and C-S bonds instead of C-O bonds. With *tert*-butyl (2-hydroxyethyl)carbamate as bis-nucleophile, [4 + 2] dearomative annulation happened regioselectively with C2-O and C3-N bond formation (**3ak**) owing to the different nucleophilicity. At the same time, we have made much efforts to construct two C-N bonds with indoles. After a number of screening, we found that sulfuric diamide could act as a dinitrogen nucleophile to couple with **1a** in 58% yield (**3al**). The cyclic sulfamide with six or seven-membered ring afforded the desired product in higher yield (**3am** and **3an**). The diamines without the protection of electron-withdrawing substituent could not react with indole under the standard reaction conditions as for the lower oxidation potential. Remarkably, mercaptoethanol and 3-mercaptopropan-1-ol could also couple with indole to afford the six- to seven-membered rings with regioselective C2-O and C3-S bond formation (**3ao** and **3ap**). Interestingly, 58% yield was obtained for the annulation of ethane-1,2-dithiol with indole to afford bioactive 1,4-dithiane (**3aq**).

### Scope of indoles

**Fig. 3:**
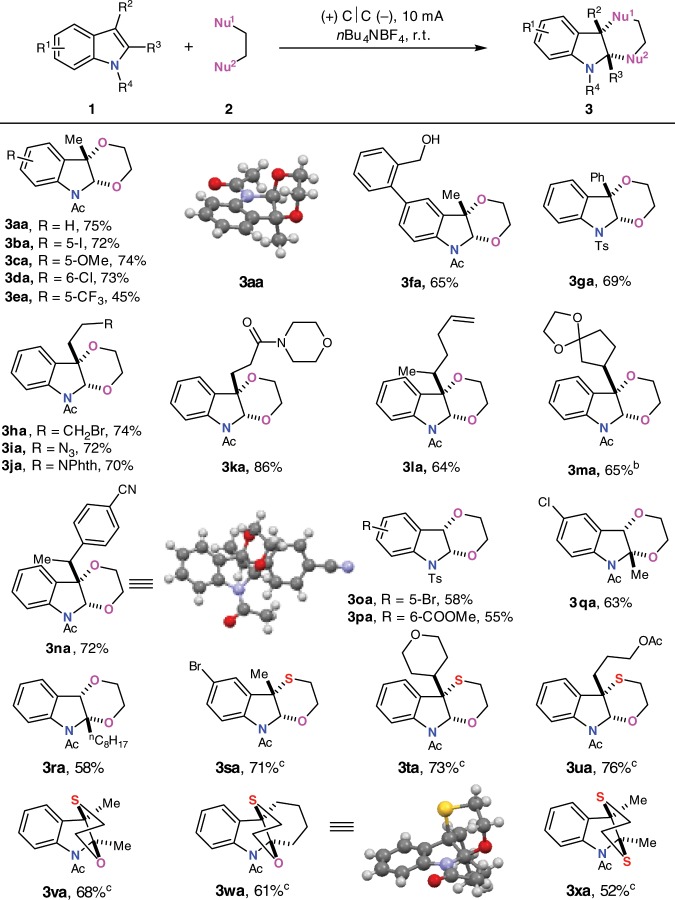
Scope of indoles. ^a^Reaction conditions: carbon cloth anode (20 mm × 20 mm), carbon cloth cathode (20 mm × 20 mm), constant current = 10 mA (*J*_anode_ = 2.5 mA cm^−2^), **1** (0.50 mmol), **2** (1.2 mL with liquid glycols, 1.3 g with solid glycols, 8 eq. with mercapto containing bis-nucleophiles), ^*n*^Bu_4_NBF_4_ (0.30 mmol), MeCN (9.0 mL), N_2_. Isolated yields were shown. ^b^Ketone and ethylene glycol condensation. ^c^MeCN/DCM = 5/4 mL as co-solvent, 15 mA (*J*_anode_ = 3.75 mA cm^−2^).

Next, various indoles were applied to couple with bis-nucleophiles under standard conditions (Fig. 3). The effect of substituents on the benzene ring was firstly scrutinized. Indole rings bearing electron-donating groups and halogens at different positions could afford the annulation product in high efficiency (**3aa**–**3da**). Moderate yield was obtained with electron-withdrawing group (**3ea**). The major side reaction was the decomposition of the indole under the reaction conditions. Free hydroxyl group at phenyl moiety was well tolerated with 65% yield (**3fa**). Phenyl substituent at C3 position of indole could also furnish the annulation product in 69% yield (**3ga**). *N*-acetyl indoles bearing different C-3 substituents such as simple functionalized alkyl, alkene and phenyl groups were suitable in this oxidative [4 + 2] dearomative annulation reaction (**3ha**–**3na**). Highly functional groups such as alkene, iodide, ester, amide, ketone, azide, and cyano were all well tolerated without hydrolysis or nucleophilic substitution by ethylene glycol. As for the reaction with unsubstituted and 2-substituted indoles (**3oa**–**3ra**), moderate yields were obtained, presumably owing to the less-stable reaction intermediates. By using mercaptoethanol as bis-nucleophile, [4 + 2] dearomative annulation also proceeded well with different phenyl- and C3 substituted indoles (**3sa**–**3ua**). When more sterically hindered 2,3-disubstituted indoles were applied, good reaction efficiency as well as regio- and stereo-selectivity were achieved (**3va–3xa**). The structure of **3wa** was confirmed by X-ray crystallographic analysis.

### Scope of benzofurans

**Fig. 4:**
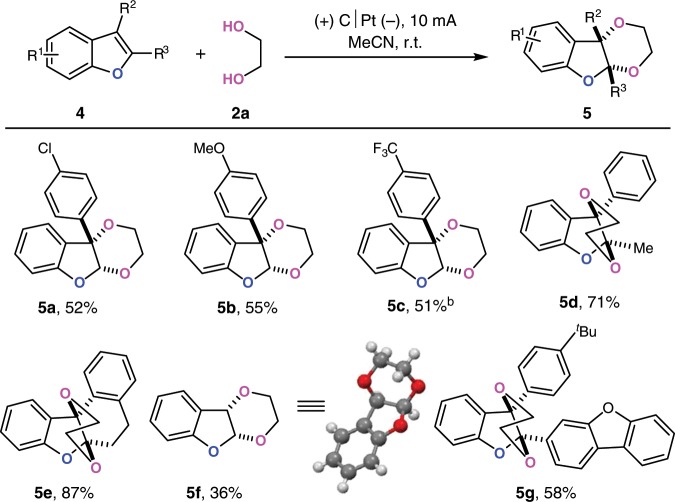
Scope of benzofurans. ^a^Reaction conditions: graphite rod anode (ϕ 6 mm), platinum plate cathode (15 mm × 15 mm × 0.3 mm), constant current = 10 mA (*J*_anode_ ≈ 11.2 mA cm^−2^), **4** (0.30 mmol), **2a** (1.0 mL), ^*n*^Bu_4_NBF_4_ (0.30 mmol), MeCN (5.0 mL), 4 h, N_2_. ^b^20 mA (*J*_anode_ ≈ 22.4 mA cm^−2^).

Interestingly, benzofurans could also couple with ethylene glycol to achieve the [4 + 2] dearomative annulation (Fig. 4). Successful reports in the oxidative dearomatization annulation of benzofurans were very limited. This reaction tolerated electron-donating groups, halogens or electron-withdrawing groups at 3-phenyl rings (**5a**–**5c**). Unsubstituted benzofuran could also couple with **2a** in moderate yield and high stereo-selectivity (**5f**). Enhanced yields were obtained from 2,3-disubstituted benzofuran (**5d** and **5e**). Moreover, benzofuran-bearing dibenzofuran group could also participate in this transformation (**5g**).

### Gram scale synthesis and product transformation

**Fig. 5:**
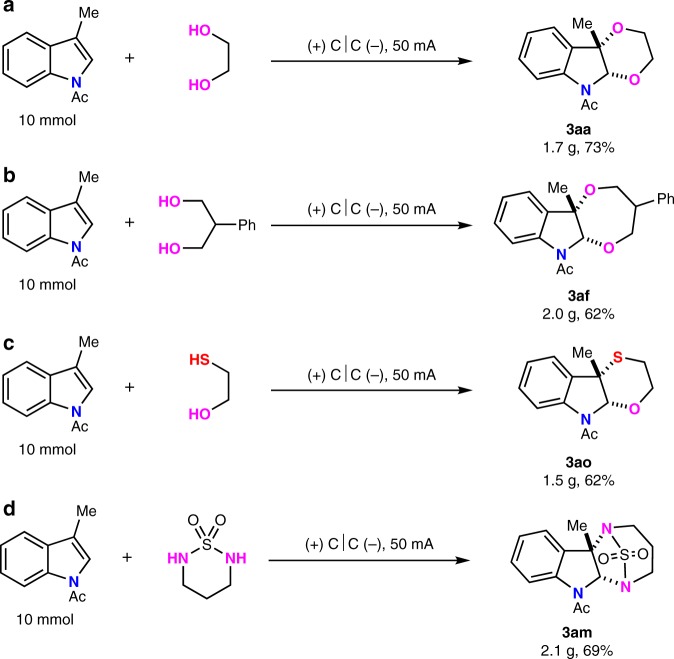
Large-scale synthesis. **a** Gram scale synthesis of **3aa**. **b** Gram scale synthesis of **3af**. **c** Gram scale synthesis of **3ao**. **d** Gram scale synthesis of **3am**.

**Fig. 6:**
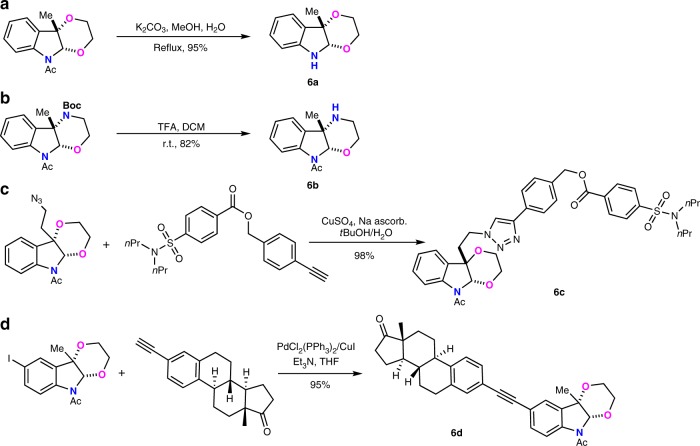
Product transformations. **a** Deprotection of **3aa**. **b** Deprotection of **3ak**. **c** Transformation of azide group in **3ia**. **d** Transformation of iodine group in **3ba**.

To further explore the utilities of this electrooxidative dearomative annulation for synthesizing polycyclic indolines, 10 mmol scale reactions were performed with four kinds of transformation in a simple beaker with 3 × 3 cm^2^ carbon cloth as both anode and cathode under air atomosphere. Using 50 mA high constant current, gram scale of polycyclic indolines could be obtained (Fig. 5a–d). The transformations for heterocycle-fused 2,3-indolines were also conducted. In the presence of K_2_CO_3_ and MeOH under reflux conditions, *N*-Ac protected **3aa**-delivered compound **6a** in 95% yield (Fig. 6a). Besides, the deprotection of N-Boc group occurred smoothly upon the treatment of **3ak** with trifluoroacetic acid, affording **6b** in 82% yield without affecting N-Ac group (Fig. 6b). The functional group of azide or iodine inside the indoline alkaloids could undergo the click or sonogashi reactions with drug molecules to afford the product **6c** and **6d**, respectively (Fig. 6c, d).

## Discussion

**Fig. 7:**
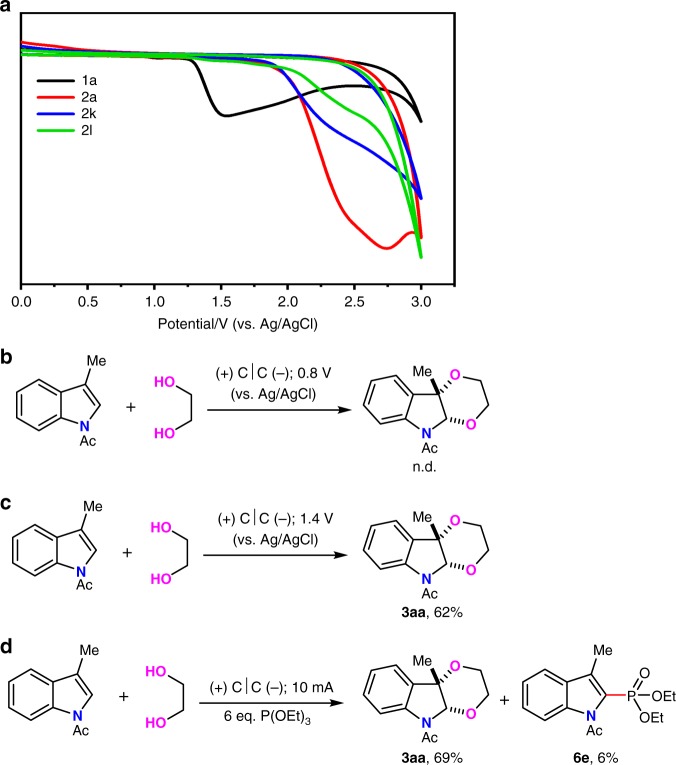
Mechanism study for the reaction of **1a** with **2a**. **a** Cyclic voltammograms on a glassy carbon electrode (ϕ 3 mm) at 0.1 Vs^−1^ under nitrogen. Black line, 3-methyl *N*-acetyl indole (**1a**); red line, ethane-1,2-diol (**2a**); blue line, *tert*-butyl (2-hydroxyethyl)carbamate (**2k**); green line, sulfuric diamide (**2l**). **b** Potential controlled electrolysis to 0.8 V (*vs* Ag/AgCl). **c** Potential controlled electrolysis to 1.4 V (vs Ag/AgCl). **d** Radical cation trapping experiment by P(OEt)_3_.

Since the method has been established, efforts were then paid to understand the mechanism for this selectively oxidative dearomatization reaction. First, cyclic voltammetry experiments of 3-methyl *N*-acetyl indole (**1a**), ethane-1,2-diol (**2a**), *tert*-butyl (2-hydroxyethyl)carbamate (**2k**), and sulfuric diamide (**2l**) were conducted (Fig. 7a). 3-Methyl *N*-acetyl indole started to be oxidized at ~1.20 V. By contrast, the oxidation onset of ethane-1,2-diol (**2a**), *tert*-butyl (2-hydroxyethyl)carbamate (**2k**), and sulfuric diamide (**2l**) was at ~ 1.86 V, so **1a** would be oxidized before **2a**, **2k** and **2l**. Then, the controlled potential electrolysis was carried out. If controlling the potential of anode to 0.8 V where both of the substrates could not be oxidized, no product was detected (Fig. 7b). If controlling the potential of anode to 1.4 V where only **1a** could be oxidized, 62% yield of product was afforded (Fig. 7c). Meanwhile, P(OEt)_3_ was added into the standard reaction to explore the existence of indole radical cation intermediate. An indole phosphorylation product could be isolated in 6% yield and 69% yield of [4 + 2] annulation product was obtained (Fig. 7d). The oxidation peak of P(OEt)_3_ was observed at 1.9 V, which was higher than that of indoles, so P(OEt)_3_ would not be oxidized under the standard reaction conditions. The large amount of ethylgloyl had better capacity of capturing in situ generated indole radical cation than P(OEt)_3_, which led to the low yield of phosphorylation product. These results indicated that the anodic oxidation of 3-methyl *N*-acetyl indole might initiate this transformation, albeit the ethane-1,2-diol (**2a**), *tert*-butyl (2-hydroxyethyl)carbamate (**2k**) and sulfuric diamide (**2l**) have not been oxidized and just acted as bis-nucleophiles. In-depth analysis of the regioselectivity issue in the case of **2k**, which contained both OH and NH nucleophilic group, C2-O and C3-N bond formation happened selectively with **1a**. We reasoned that the radical of indole radical cation mainly delocalized at C3 position with the stablization of phenyl group, and cation mainly existed at C2 position via the formation of imine. Since the nucloephilicity of OH is stronger than that of NHBoc, the nucleophilic attack of OH happened first at C2 position of indole radical cation to afford the benzylic carbon radical. The second oxidation of benzylic carbon radical gave carbon cation, followed by the intramolecular attack of NHBoc to form C-N bond at C3 position.

In addition, in-operando infrared spectroscopy analysis of the electrooxidative dearomative annulation benween **1a** and **2a** by react-IR technology did not show any sign for an induction period (see Supplementary Fig. 1a). Kinetic studies of this process were performed by detecting the initial reaction rate with different current, different loading of 3-methyl *N*-acetyl indole (**1a**) and ethylene glycol (**2a**). It was found that the initial rate increased in a linear fashion with increasing current (see Supplementary Fig. 1b). As for the kinetic profiles of substrates, the first-order dependencies at low concentration of **1a** was observed and the reaction rate was saturated at high concentrations (see Supplementary Fig. 1c). This might be attributed to the current controlled result at high concentration of **1a**. However, the initiate rate constants showed to be independent of the concentration of **2a** (see Supplementary Fig. 1d). Combining these kinetic behaviors with mechanism study above, it suggested that the anodic oxidation of **1a** to indole radical cation was the rate-determining step.

**Fig. 8:**
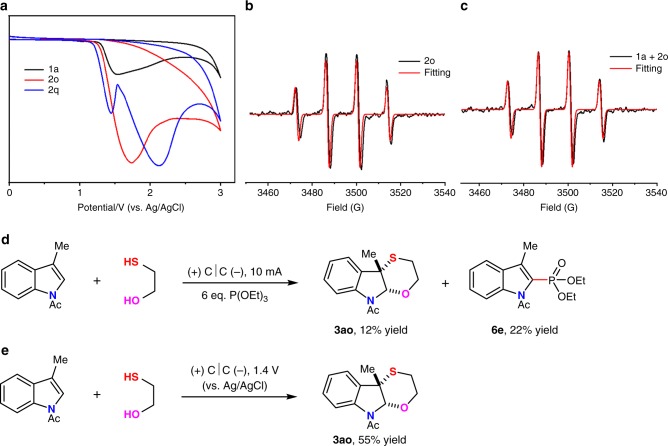
Mechanism study for the reaction of **1a** with **2o**. **a** Cyclic voltammograms on a glassy carbon electrode (ϕ 3 mm) at 0.1 Vs^−1^ under nitrogen. Black line, 3-methyl *N*-acetyl indole (**1a**); red line, mercaptoethanol (**2o**); blue line, ethane-1,2-dithiol (**2q**). **b** EPR measurements of **2o** in the presence of DMPO under constant current conditions for 6 min. **c** EPR measurements of **1a** and **2o** in the same conditions. **d** Radical cation trapping experiment by P(OEt)_3_. **e** Controlled potential electrolysis.

Next, cyclic voltammograms of 3-methyl *N*-acetyl indole (**1a**), 2-mercaptoethanol (**2o**), and ethane-1,2-dithiol (**2q**) were measured (Fig. 8a). 3-Methyl *N*-acetyl indole (**1a**), mercaptoethanol (**2o**), and ethane-1,2-dithiol (**2q**) all started to be oxidized at about 1.20 V. These results indicated that 3-methyl *N*-acetyl indole (**1a**), mercaptoethanol (**2o**), and ethane-1,2-dithiol (**2q**) might all be oxidized at the same time during the electrolysis to generate reactive intermediates. As both of the substrates could be oxidized, electron paramagnetic resonance (EPR) experiments were performed to explore the radical species with adding free radical spin trapping agent DMPO (5,5-dimethyl-1-pyrroline *N*-oxide) into the reaction system. When DMPO was added to the reaction of mercaptoethanol (**2o**) under the standard conditions, an obvious EPR signal was observed, which was suggested as an alkyl sulfur radical. This radical was quickly trapped by DMPO to afford a more stable radical (*g* = 2.00688, AN = AH = 13.8 G) (Fig. 8b). Next, when DMPO was added to the reaction mixture of **1a** and **2o**, the same radical signal was observed, which further proved the existence of sulfur-center radical during the reaction of **1a** and **2o** (Fig. 8c). On the other hand, if adding six equivalent of triethyl phosphite into the standard reaction, 12% yield of [4 + 2] annulation product could be gained. At the same time, an indole phosphorylation product **6e** could be isolated in 22% yield, which proved the existence of indole radical cation intermediate (Fig. 8d). Then, the controlled potential electrolysis was carried out. Controlling the potential of anode to 1.4 V where both of **1a** and **2o** could be oxidized, 55% corresponding product was obtained (Fig. 8e). These results indicated that the reaction might go through the cross-coupling of sulfur radical with indole cation radical intermediate. Subsequent intramolecular annulation afforded the [4 + 2] dearomative annulation product. As for the regioselective issue in the case of **1a** with **2o** where C-S bond formed at C3 position and C-O bond formed at C2 position selectively, we speculated that the reaction proceeded through the cross-coupling of sulfur radical with indole radical cation at C3 position where the radical species was stabilized by phenyl group. Subsequent intramolecular nucleophilic attack of OH to imine cation afforded the corresponding product.

Then, kinetic studies for the dearomative annulation of **1a** with **2o** were performed to determine the order of reaction components in this dearomative annulation with different loading of 3-methyl *N*-acetyl indole (**1a**) and mercaptoethanol (**2o**). As for the kinetic profiles of the substrates, the first-order dependencies of **1a** was observed (see Supplementary Fig. 2a). However, the initiate rate was almost invariant when using different concentrations of **2o**, indicating that the reaction rate is independent of the concentration of **2o** (see Supplementary Fig. 2b). Similar to the kinetic behavior of **1a** with **2a**, the results suggested that the anodic oxidation of **1a** into indole radical cation was the slowest step throughout the transformation, and the processes of anodic oxidation of S-H into sulfur radical, radical–radical cross-coupling and cascade nucleophilic attack might be relative fast steps.

**Fig. 9:**
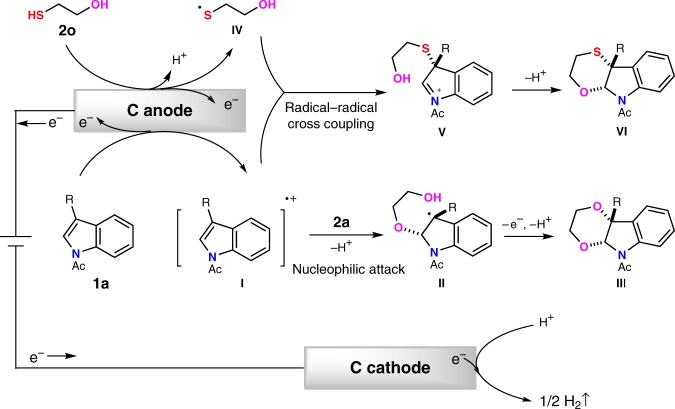
Plausible mechanism for the dearomative annulation of indole. The plausible mechanism involves anodic oxidation of indole to generate indole radical cation. Two steps of nucleophilic attack by ethylene glycol accompanied with an oxidation process would afford product **3aa**. On the other hand, radical–radical cross-coupling with in situ generated sulfur radical and subsequent intramolecular nucleophilic attack would furnish product **3ao**.

Based on the above experiment results, a plausible mechanism for these electrooxidative dearomatization annulations was shown in Fig. 9. In the first step, **1a** would be oxidized at carbon anode to generate a radical cation **I** where radical mostly lied at C3 position with stablization of phenyl group and cation mostly lied at C2 position stabilized by the formation of imine cation. This reactive intermediate could undergo two different transformations by making use of radical and cation centers. If ethane-1,2-diol (**2a**) was applied as bis-nucleophile, first nucleophilic attack at the C2 position of **I** afforded benzylic carbon radical intermediate **II**. The oxidation of benzylic carbon radical gave carbon cation, followed by the intramolecular attack of OH to form C-O bond at C3 position. If bis-nucleophiles contains mercaptan, single-electron oxidation of mercaptan could generate sulfur-center radical in the meantime. Radical–radical cross-coupling between indole radical cation and sulfur radical at C3 position afforded intermediate **V**. Subsequently, the intramolecular attack of nucleophilic side chain to iminium moiety yielded the corresponding polycyclic indolines with excellent *cis*-diastereo-selectivity. The processes of radical–radical cross-coupling and nucleophilic attack might also happen at the same time.

In summary, we have developed an electrochemical method for the oxidative dearomatization annulation of indole and benzofuran derivatives. Under undivided electrolytic conditions, [3 + 2], [4 + 2], [5 + 2], as well as [6 + 2] annulation happened regio- and setero-selectively with O-, N-, S-nucleophilic groups. A series of highly functionalized five to eight-membered heterocycle-2,3-fused indolines and dihydrobenzofurans were afforded, which are typically unattainable under thermal conditions. Notably, highly active functional groups such as iodide, hydroxyl, alkene, azide, ester, amide, cyano, carbonyl groups, etc. could all be well tolerated after electrolysis. A detailed mechanistic survey, including cyclic voltammetry, EPR, radical trapping experiments, and kinetic studies have been presented. These results demonstrate that anodic oxidation-induced indole radical cation has a vital role in these transformations and this process is the rate-determing step. The dearomative annulation of indoles with diols is believed to proceed through the two-step nucleophilic attack, whereas the annulation with mercapto group containing bis-nucleophiles proceeds through the radical–radical cross-coupling of indole radical cation and sulfur radical with subsequent nucleophilic attack. The unique reaction pathways have resulted in the high regio- and diastereo-selectivity. Finally, we believe this powerful strategy would stimulate broad interests in the journey of natural product total synthesis.

## Methods

### General procedure for the reaction of 1a with 2a

In an oven-dried undivided three-necked bottle (25 mL) equipped with a stir bar, *N*-acetyl indole (0.5 mmol), *n*Bu_4_NBF_4_ (98.7 mg, 0.30 mmol), and MeCN/ethane-1,2-diol (9.0 mL/1.2 mL) were combined and added. The bottle was equipped with carbon cloths (20 mm × 20 mm) as both the anode and cathode and then charged with nitrogen. Then the electrolysis system was stirred at a constant current of 10 mA at room temperature until the complete consumption of *N*-acetyl indole (detected by thin-layer chromatography; TLC). When the reaction finished, the reaction mixture was washed with water and extracted with diethyl ether (10 mL × 3). The organic layers were combined, dried over Na_2_SO_4_, and concentrated. The pure product was obtained by flash column chromatography on silica gel (petroleum: ethyl acetate = 7:1). Full experimental details and characterization of the compounds are given in the Supplementary Information.

### General procedure for the reaction of 1a with 2o

In an oven-dried undivided three-necked bottle (25 mL) equipped with a stir bar, *N*-acetyl indole (0.5 mmol), *n*Bu_4_NBF_4_ (98.7 mg, 0.3 mmol), **2o** (8 eq.), and MeCN/DCM (5.0 mL/4 mL) were combined and added. The bottle was equipped with carbon cloths (20 mm × 20 mm) as both the anode and cathode and then charged with nitrogen. Then the electrolysis system was stirred at a constant current of 15 mA at room temperature until the complete consumption of *N*-acetyl indole (detected by TLC). When the reaction finished, the reaction mixture was washed with water and extracted with diethyl ether (10 mL × 3). The organic layers were combined, dried over Na_2_SO_4_, and concentrated. The pure product was obtained by flash column chromatography on silica gel (petroleum: ethyl acetate = 7:1). Full experimental details and characterization of the compounds are given in the Supplementary Information.

## Supplementary information

Supplementary Information

## Data Availability

The X-ray crystallographic coordinates for structures reported in this article have been deposited at the Cambridge Crystallographic Data Centre (CCDC), under deposition number CCDC 1963165 (**3aa**), CCDC 1963166 (**3na′**), CCDC 1963167 (**3wa**), CCDC 1963168 (**5f**), CCDC 1963169 (**3na**). The data can be obtained free of charge from The Cambridge Crystallographic Data Centre [http://www.ccdc.cam.ac.uk/data_request/cif]. The data supporting the findings of this study are available within the article and its Supplementary Information files. Any further relevant data are available from the authors on request
