# Supplementary material for: Electrooxidation enables highly regioselective dearomative annulation of indole and benzofuran derivatives
Source: Nat Commun. 2020 Jan 7;11:3. doi: 10.1038/s41467-019-13829-4 (PMC6946675; doi:10.1038/s41467-019-13829-4)
Supplement: Supplementary file 1 — Supplementary Information [file 41467_2019_13829_MOESM1_ESM.pdf]

Supplementary Information

Electrooxidation Enables Highly Regioselective Dearomative Annulation of Indole and Benzofuran Derivatives

Liu et al

Supplementary Methods

All glasswares were oven dried at 110 °C for hours and cooled down under vacuum. **1a-1k, 1o-1s, 1u-1w**[1]; **1l, 1n, 1t**[2]; **1m**[3]; **4a-4e, 4g**[4] were prepared according to reported procedures. Unless otherwise noted, materials were obtained from commercial suppliers and used without further purification. The instrument for electrolysis is dual display potentiostat (DJS-292B) (made in China). Cyclic voltammograms were obtained on a CHI 605E potentiostat. EPR spectra were recorded on a Bruker X-band A-200 spectrometer. The samples were taken out by a capillary (borosilicate glass, 0.8-1.1 ×100 mm), and then recorded by EPR spectrometer at indicated temperature and parameters. For the reactions of *N*-acetylindole with bis-nucleophiles, carbon cloths (20 mm×20 mm) were used both as anode and cathode. For the reactions of benzofuran with bis-nucleophiles, the anodic electrode was graphite rod (ϕ 6 mm) and cathodic electrode was platinum plate (15 mm×15 mm×0.3 mm). Thin layer chromatography (TLC) employed glass 0.25 mm silica gel plates. Flash chromatography columns were packed with 200-300 mesh silica gel in petroleum (bp. 60-90 °C). ^{1}H and ^{13}C NMR data were recorded with Bruker Advance III (400 MHz) spectrometers with tetramethylsilane as an internal standard. All chemical shifts (δ) are reported in ppm and coupling constants (*J*) in Hz. All chemical shifts were reported relative to tetramethylsilane (0 ppm for ^{1}H), $CDCl_3$ (77.0 ppm for ^{13}C) and DMSO-d^6 (2.50 ppm for ^{1}H, 39.50 ppm for ^{13}C), respectively. High resolution mass spectra (HRMS) were measured with a Waters Micromass GCT instrument and accurate masses were reported for the molecular hydrogen ion $(M+H)^+$.

General procedure for the electrooxidative deromative [4+2] cyclization of *N*-acetylindole with ethane-1,2-diol: In an oven-dried undivided three-necked bottle (25 mL) equipped with a stir bar, *N*-acetylindole (0.5 mmol), $^{n}Bu_4NBF_4$ (98.7 mg, 0.30 mmol) and MeCN/ethane-1,2-diol (9.0 mL/1.2 mL) were combined and added. The bottle was equipped with carbon cloths (20 mm×20 mm) as both the anode and cathode and then charged with nitrogen. Then the electrolysis system was stirred at a constant current of 10 mA at room temperature until the complete consumption of *N*-acetylindole (detected by TLC). When the reaction finished, the reaction mixture was washed with water and extracted with diethyl ether (10 mL x 3). The organic layers were combined, dried over Na_2SO_4, and concentrated. The pure product was obtained by flash column chromatography on silica gel (petroleum: ethyl acetate = 7:1).

General procedure for the electrooxidative deromative [3+2] cyclization of *N*-acetylindole with sulfuric diamide: In an oven-dried undivided three-necked bottle (25 mL) equipped with a stir bar, *N*-acetylindole (0.5 mmol), $^{n}Bu_4NBF_4$ (98.7 mg, 0.30 mmol), sulfuric diamide (2.0 mmol, 4 equiv) and MeCN/DCM (5.0 mL/4.0 mL) were combined and added. The bottle was equipped with carbon cloths (20 mm×20 mm) as both the anode and cathode and then charged with nitrogen. Then the electrolysis system was stirred at a constant current of 10 mA at room temperature until the complete consumption of *N*-acetylindole (detected by TLC). When the reaction finished, the reaction mixture was washed with water and extracted with diethyl ether (10 mL x 3). The organic layers were combined, dried over Na_2SO_4, and concentrated. The pure product was obtained by flash column chromatography on silica gel (petroleum: ethyl acetate = 2:1).

General procedure for the electrooxidative deromative cyclization of *N*-acetylindole with mercapto containing bis-nucleophiles: In an oven-dried undivided three-necked bottle (25 mL) equipped with a stir bar, *N*-acetylindole (0.5 mmol), $^{n}Bu_4NBF_4$ (98.7 mg, 0.3 mmol), bis-nucleophile (8 equiv) and MeCN/DCM (5.0 mL/4 mL) were combined and added. The bottle was equipped with carbon cloths (20 mm×20 mm) as both the anode and cathode and then charged with nitrogen. Then the electrolysis system was stirred at a constant current of 15 mA at room temperature until the complete consumption of *N*-acetylindole (detected by TLC). When the reaction finished, the reaction mixture was washed with water and extracted with DCM (10 mL x 3). The organic layers were combined, dried over Na_2SO_4, and concentrated. The pure product was obtained by flash column chromatography on silica gel (petroleum: ethyl acetate = 7:1).

General procedure for the electrooxidative dearomative cyclization of benzofuran with different ethane-1,2-diol: In an oven-dried undivided three-necked bottle (15 mL) equipped with a stir bar, benzofuran (0.3 mmol), $^{n}Bu_4NBF_4$ (65.8 mg, 0.2 mmol), and MeCN/ethane-1,2-diol (5.0 mL/1.0 mL) were combined and added. The bottle was equipped with graphite rod (ϕ 6 mm, about 15 mm immersion depth in solution) as anode and platinum plate as cathodic electrode (15 mm×15 mm×0.3 mm) and then charged with nitrogen. Then the electrolysis system was stirred at a constant current of 10 mA at room temperature for 4 h. When the reaction finished, the reaction mixture was washed with water and extracted with diethyl ether (10 mL x 3). The organic layers were combined, dried over Na_2SO_4, and concentrated. The pure product was obtained by flash column chromatography on silica gel (petroleum: ethyl acetate = 100:1).

Procedure for gram scale synthesis of 1-(9b-methyl-2H-[1,4]dioxino[2,3-b]indol-5(3H,4aH,9bH)-yl)ethanone: In an oven-dried undivided three-necked bottle (250 mL) equipped with a stir bar, *N*-acetylindole (10 mmol), $^{n}Bu_4NBF_4$ (1.65 g, 5 mmol), ethane-1,2-diol (20 mL) and MeCN(180 mL) were combined and added. The bottle was equipped with carbon cloths (30 mm×30 mm) as both the anode and cathode. Then the electrolysis system was stirred at a constant current of 50 mA at room temperature under air atmosphere for 16 h. When the reaction finished, the reaction mixture was washed with water and extracted with diethyl ether (100 mL x 3). The organic layers were combined, dried over Na_2SO_4, and concentrated. The pure product was obtained by flash column chromatography on silica gel (petroleum: ethyl acetate = 7:1). White solid was obtained in 73% isolated yield (1.7 g).

Procedure for gram scale synthesis of 1-(10b-methyl-3-phenyl-3,4-dihydro-[1,4]dioxepino[2,3-b]indol-6(2H,5aH,10bH)-yl)ethanone or 1-(9b-methyl-2H-[1,4]oxathiino[2,3-b]indol-5(3H,4aH,9bH)-yl)ethanone: In an oven-dried undivided three-necked bottle (250 mL) equipped with a stir bar, *N*-acetylindole (10.0 mmol), $^{n}Bu_4NBF_4$ (1.65 g, 5.0 mmol), (2-phenylpropane-1,3-diol (23.0 g) or 2-mercaptoethanol (5 equiv)), and MeCN/DCM=100/80 mL were combined and added. The bottle was equipped with carbon cloths (30 mm×30 mm) as both the anode and cathode. Then the electrolysis system was stirred at a constant current of 50 mA until the total consumption of indole. When the reaction finished, the reaction mixture was washed with water and extracted with diethyl ether (100 mL x 3). The organic layers were combined, dried over Na_2SO_4, and concentrated. The pure product was obtained by flash column chromatography on silica gel (petroleum: ethyl acetate = 7:1). White solid was obtained in 61% isolated yield (2.0 g) for 1-(10b-methyl-3-phenyl-3,4-dihydro-[1,4]dioxepino[2,3-b]indol-6(2H,5aH,10bH)-yl)ethanone and colorless oil was obtained in 62% isolated yield (1.5 g) for 1-(9b-methyl-2H-[1,4]oxathiino[2,3-b]indol-5(3H,4aH,9bH)-yl)ethanone.

Procedure for gram scale synthesis of 1-(10b-methyl-11,11-dioxido-3,4-dihydro-1,5-epithio[1,4]diazepino[2,3-b]indol-6(2H,5aH,10bH)-yl)ethanone: In an oven-dried undivided three-necked bottle (250 mL) equipped with a stir bar, *N*-acetylindole (10.0 mmol), $^{n}Bu_4NBF_4$ (1.65 g, 5.0 mmol), 1,2,6-thiadiazinane 1,1-dioxide (4 equiv), and MeCN/DCM=100/80 mL were combined and added. The bottle was equipped with carbon cloths (30 mm×30 mm) as both the anode and cathode. Then the electrolysis system was stirred at a constant current of 50 mA until the

total consumption of indole. When the reaction finished, the reaction mixture was washed with water and extracted with diethyl ether (100 mL x 3). The organic layers were combined, dried over Na_2SO_4, and concentrated. The pure product was obtained by flash column chromatography on silica gel (petroleum: ethyl acetate = 7:1). White solid was obtained in 69% isolated yield for 1-(10b-methyl-11,11-dioxido-3,4-dihydro-1,5-epithio[1,4]diazepino[2,3-b]indol-6(2H,5aH,10bH)-yl)ethanone.

General procedure for cyclic voltammetry (CV): Cyclic voltammetry was performed in a three-electrode cell connected to a schlenk line under nitrogen at room temperature. The working electrode was a steady glassy carbon disk electrode, the counter electrode a platinum wire. The reference was a Ag/AgCl electrode submerged in saturated aqueous KCl solution, and separated from reaction by a salt bridge. 5.0 mL of acetonitrile containing 0.1 M $LiClO_4$ were poured into the electrochemical cell in all experiments. The scan rate is 0.1 V/s, ranging from 0 V to 3.0 V

General procedure for the Electron Paramagnetic Resonance (EPR) experiment:

Under constant current conditions, a dried three-necked flask equipped with a stir bar was loaded with 2-mercaptoethanol **(**4.0 mmol**)** or mixture of 2-mercaptoethanol (4.0 mmol) and **1a** (0.5 mmol), $^{n}Bu_4NBF_4$ (98.7 g, 0.3 mmol) and DMPO (30 μL) were electrolyzed in MeCN/DCM (5/4 mL) under N_2 atmosphere, respectively. After 8 min, the reaction solution was taken out by capillary and analyzed by EPR at room temperature. The samples were taken out by a capillary (borosilicate glass, 0.8-1.1 ×100 mm), and then recorded by EPR spectrometer at indicated temperature and parameters.

General procedure for kinetic study between 1a and 2a monitored by GC:

In an oven-dried undivided three-necked bottle (25 mL) equipped with a stir bar, **1a** (0.50 mmol) and $^{n}Bu_4NBF_4$ (98.8 mg, 0.3 mmol) was added. Diphenyl (20.0 mg) was added as an internal standard. The bottle was equipped with carbon cloth (20 mm×20 mm) as the anode and cathode and charged with nitrogen. Subsequently, **2a** (1.2 mL) and CH_3CN (9.0 mL) were added. Then the electrolysis system was stirred at a constant current of 10 mA. 0.1 mL solution were taken out from the cell via syringe at designated time interval (30, 60 min).

Order in Current: The order in current was determined by studying the initial rate of reaction under different current (4, 7, 10, 13, 16, 20 mA). Using the above mentioned general procedure, product yield from the corresponding reaction was monitored by GC using diphenyl as an internal standard. Finally, the profiles of relative concentrations *vs* time for product **3aa** could be obtained

to analyse the initial rate of reaction. As shown below, the reaction rate changed under different current, which demonstrated to be a first-order dependence.

Order in substrate 1a: The order in **1a** was determined by studying the initial rate of reaction with different concentration of **1a** (0.00952, 0.01429, 0.02381, 0.03333, 0.04, 0.04762, 0.05714，0.06667, 0.07619 M). Using the above mentioned general procedure, product yield from the corresponding reaction was monitored by GC using diphenyl as an internal standard. Finally, the profiles of relative concentrations *vs* time for product **3aa** could be obtained to analyse the initial rate of reaction. As shown below, the reaction rate changed with different concentration of **1a**, which demonstrated to be a saturation kinetics.

Order in substrate 2a: The order in **2a** was determined by studying the initial rate of reaction with different volume of **2a** (0.8, 1.0, 1.2, 1.5, 1.8, 2.1 mL), and the total volume maintain 10.2 mL. Using the above mentioned general procedure, product yield from the corresponding reaction was monitored by GC using diphenyl as an internal standard. Finally, the profiles of relative concentrations *vs* time for product **3aa** could be obtained to analyse the initial rate of reaction. As shown below, the reaction rate did not change with different concentration of **2a**.

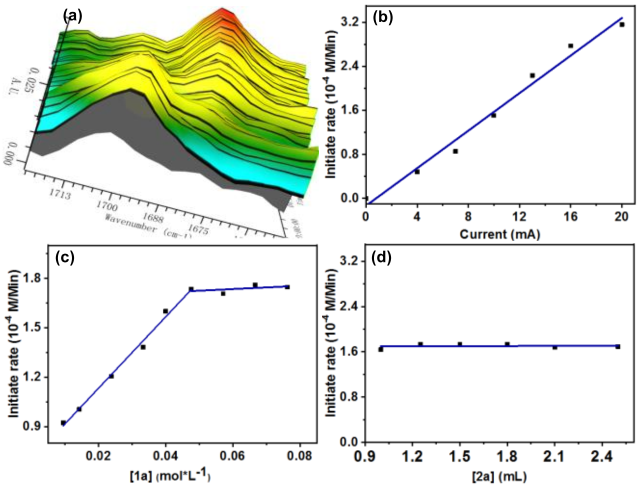

Supplementary Figure 1. Kinetic studies for the dearomative annulation of indole with ethylene glycol. **a** In-operando infrared spectroscopy analysis for the reaction of **1a** and **2a**. **b** kinetic profiles of current. **c** kinetic profiles of **1a. d** kinetic profiles of **2a**.

General procedure for kinetic study between 1a and 2o monitored by GC:

In an oven-dried undivided three-necked bottle (25 mL) equipped with a stir bar, **1a** (0.50 mmol) and $^{n}Bu_4NBF_4$ (98.8 mg, 0.3 mmol) was added. Diphenyl (10.0 mg) was added as an internal standard. The bottle was equipped with carbon cloth (20 mm×20 mm) as the anode and cathode and

charged with nitrogen. Subsequently, **2o** (8 equiv) and CH_3CN/DCM (5.0/4.0 mL) were added. Then the electrolysis system was stirred at a constant current of 15 mA. 0.1 mL solution were taken out from the cell via syringe at designated time interval (4h, 8h).

Order in substrate 1a: The order in **1a** was determined by studying the initial rate of reaction with different concentration of **1a** (0.01064, 0.01596, 0.0266, 0.03723, 0.05319, 0.06383 M). Using the above mentioned general procedure, product yield from the corresponding reaction was monitored by GC using diphenyl as an internal standard. Finally, the profiles of relative concentrations *vs* time for product **3ao** could be obtained to analyse the initial rate of reaction. As shown below, the reaction rate changed with different concentration of **1a**, which demonstrated to be a first-order kinetic.

Order in substrate 2o: The order in **2o** was determined by studying the initial rate of reaction with different volume of **2o** (0.22222, 0.27778, 0.38889, 0.44444, 0.55556 M). Using the above mentioned general procedure, product yield from the corresponding reaction was monitored by GC using diphenyl as an internal standard. Finally, the profiles of relative concentrations *vs* time for product **3ao** could be obtained to analyse the initial rate of reaction. As shown below, the reaction rate did not change with different concentration of **2o**.

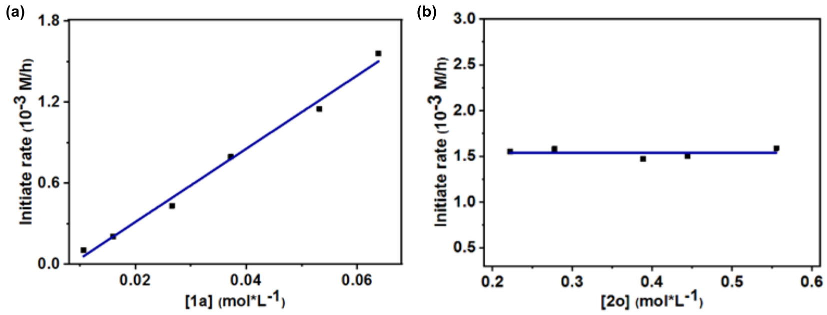

Supplementary Figure 2. Kinetic study between **1a** and **2o** monitored by GC. **a** kinetic profiles of **1a. b** kinetic profiles of **2o**.

Crystal structure determination of compounds 3aa, 3na and 3na′, 3wa and 5f: Single crystal of the compounds were selected, mounted onto a cryoloop, and transferred in a cold nitrogen gas stream. Intensity data were collected with a BRUKER Kappa-APEXII diffractometer with graphite-monochromated Cu-Kα radiation (λ = 0.71073 Å). Data collection were performed with APEX2 suite (BRUKER). Unitcell parameters refinement, integration and data reduction were carried out with SAINT program (BRUKER). SADABS (BRUKER) was used for scaling and multi-scan absorption corrections. In the WinGX suite of programs, the structure were solved with Sir2014

program and refined by fullmatrix least-squares methods using SHELXL-14.

3aa

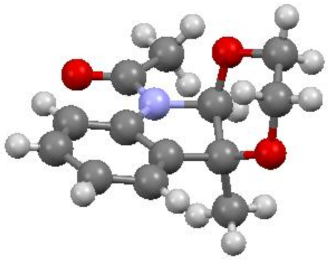

Supplementary Figure 3. Crystal structure of 3aa

Supplementary Table 1.Crystallography Data of 3aa.

Empirical formula	$C_{13}H_{15}NO_3$
Formula weight	233.1052
Space group	P n a 21
a (Å)	15.1129
b (Å)	8.7941
c (Å)	8.7383
α (deg)	90
β (deg)	90
γ (deg)	90
V (Å^3)	1161.36
Z	4
T (K)	150 K
ρ_{calcd} (g/cm^3)	1.334
μ (mm^{-1})	0.780
Significant reflections	1749
R[I > 2.5 *(I)*]	0.0313
R_w[I > 2.5 *(I)*]	0.0835

3na

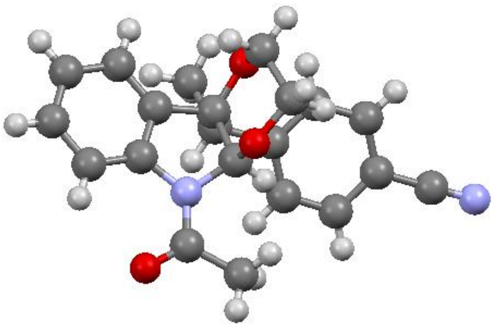

Supplementary Figure 4. Crystal structure of 3na.

Supplementary Table 2.Crystallography Data of 3na.

Empirical formula	$C_{21}H_{20}N_2O_3$
Formula weight	348.1474
Space group	P 21/n
a (Å)	12.1188
b (Å)	11.9598
c (Å)	13.2257
α (deg)	90
β (deg)	108.547
γ (deg)	90
V (Å^3)	1817.35
Z	4
T (K)	200 K
ρ_{calcd} (g/cm^3)	1.273
μ (mm^{-1})	0.086
Significant reflections	5120
R[I > 2.5 *(I)*]	0.0370
R_w[I > 2.5 *(I)*]	0.1022

3na′

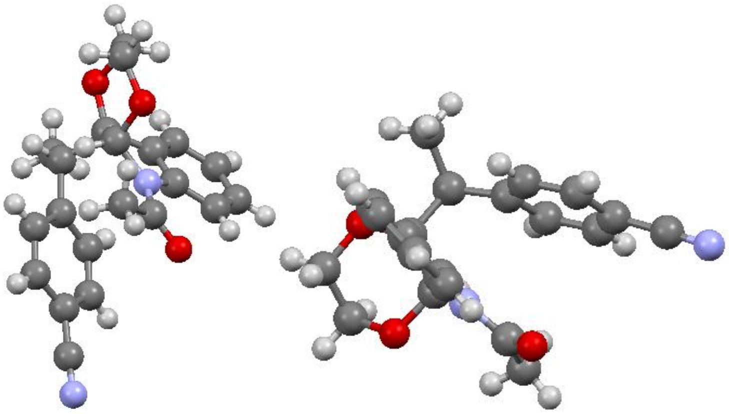

Supplementary Figure 5. Crystal structure of 3na′

Supplementary Table 3.Crystallography Data of 3na′.

Empirical formula	$C_{21}H_{20}N_2O_3$
Formula weight	348.1474
Space group	P 21/n
a (Å)	8.4756
b (Å)	15.0120
c (Å)	14.1226
α (deg)	90
β (deg)	94.873
γ (deg)	90

V (Å^3)	1412.12
Z	4
T (K)	200 K
ρ_{calcd} (g/cm^3)	1.292
μ (mm^{-1})	0.706
Significant reflections	3061
R[I > 2.5 *(I)*]	0.0431
R_w[I > 2.5 *(I)*]	0.1064

3wa

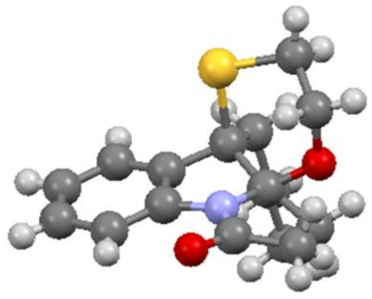

Supplementary Figure 6. Crystal structure of 3wa

Supplementary Table 4.Crystallography Data of 3wa.

Empirical formula	$C_{16}H_{19}NO_2S$
Formula weight	289.38
Space group	P 21/c
a (Å)	12.5892
b (Å)	12.4173
c (Å)	9.1785
α (deg)	90
β (deg)	94.197
γ (deg)	90
V (Å^3)	1430.97
Z	4
T (K)	200 K
ρ_{calcd} (g/cm^3)	1.343
μ (mm^{-1})	2.013
Significant reflections	2671
R[I > 2.5 *(I)*]	0.0801
R_w[I > 2.5 *(I)*]	0.2098

5f

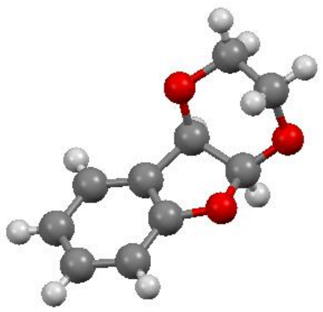

Supplementary Figure 7. Crystal structure of 5f

Supplementary Table 4.Crystallography Data of 5f.

Empirical formula	$C_{10}H_{10}O_3$
Formula weight	178.0630
Space group	P 21 21 21
a (Å)	8.9607
b (Å)	9.0141
c (Å)	10.4727
α (deg)	90
β (deg)	90
γ (deg)	90
V (Å^3)	845.91
Z	4
T (K)	150 K
ρ_{calcd} (g/cm^3)	1.339
μ (mm^{-1})	0.859
Significant reflections	1398
R[I > 2.5 *(I)*]	0.0231
R_w[I > 2.5 *(I)*]	0.059

Detail descriptions for products

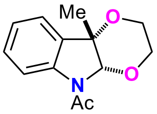

1-(9b-Methyl-2H-[1,4]dioxino[2,3-b]indol-5(3H,4aH,9bH)-yl)ethanone (3aa): white soild was obtained in 75% isolated yield. ^{1}H NMR (400 MHz, $CDCl_3$) δ 8.16 (d, *J* = 8.0 Hz, 1H), 7.36 – 7.28 (m, 2H), 7.18 - 7.10 (m, 1H), 5.12 (s, 1H), 3.83 – 3.73 (m, 1H), 3.71 – 3.56 (m, 3H), 2.37 (s, 3H), 1.44 (s, 3H). ^{13}C NMR (101 MHz, $CDCl_3$) δ 169.85, 141.08, 131.92, 129.41, 124.32, 122.51, 117.00, 89.16, 77.87, 61.38, 60.76, 26.90, 23.13. HRMS (ESI) calculated for $C_{13}H_{16}NO_3^+$ $[M+H]^+$: 234.1125; found: 234.1124.

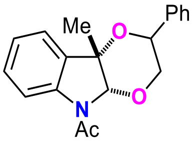

1-(9b-Methyl-2-phenyl-2H-[1,4]dioxino[2,3-b]indol-5(3H,4aH,9bH)-yl)ethanone (3ab): white solid was obtained in 44% isolated yield. ^{1}H NMR (400 MHz, DMSO-d_6) δ 8.11 (d, *J* = 8.4 Hz, 1H), 7.42 – 7.28 (m, 7H), 7.16 (td, *J* = 7.2, 0.8 Hz, 1H), 5.76 (s, 1H), 4.61 (dd, *J* = 6.8, 5.6 Hz, 1H), 3.77 – 3.59 (m, 2H), 2.27 (s, 3H), 1.55 (s, 3H). ^{13}C NMR (101 MHz, DMSO-d_6) δ 170.34, 141.83, 139.14, 131.65, 129.70, 128.51, 127.98, 126.31, 123.95, 123.59, 115.59, 88.69, 75.02, 67.79, 63.95, 24.43, 23.58. HRMS (ESI) calculated for $C_{19}H_{20}NO_3^+$ $[M+H]^+$: 310.1438; found: 310.1434.

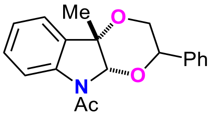

1-(9b-Methyl-3-phenyl-2H-[1,4]dioxino[2,3-b]indol-5(3H,4aH,9bH)-yl)ethanone (3ab′): white solid was obtained in 44% isolated yield. As for the partial π-π stacking interaction of phenyl ring with indole, the spectra demonstrate a mixture of isomers (80:20) where the oxygens of both isomers stand at the *cis*-position. ^{1}H NMR (400 MHz, DMSO-d_6) δ 8.12 – 8.00 (m, 1H), 7.45 – 7.13 (m, 8H), 5.65 (s, 0.8H), 5.50 (s, 0.2H), 4.89 (dd, *J* = 10.0, 2.4 Hz, 0.8H), 4.53 (dd, *J* = 9.6, 2.8 Hz, 0.2H), 3.84 (dd, *J* = 11.6, 2.4 Hz, 0.8H), 3.76 (dd, *J* = 12.0, 2.8 Hz, 0.2H), 3.64 – 3.56 (m, 0.2H), 3.26 (dd, *J* = 11.6, 10.4 Hz, 0.8H), 2.34 – 2.28 (m, 3H), 1.45 (s, 0.6H), 1.40 (s, 2.4H). ^{13}C NMR (101 MHz, DMSO-d_6) δ 169.66, 140.87, 137.03, 132.78, 129.11, 128.36, 128.06, 126.49, 126.02, 124.29, 122.54, 116.37, 88.78, 77.52, 72.93, 65.87, 26.61, 22.95. HRMS (ESI) calculated for $C_{19}H_{20}NO_3^+$ $[M+H]^+$: 310.1438; found: 310.1436.

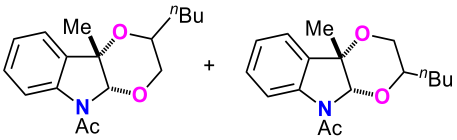

(1:1)

1-(2-Butyl-9b-methyl-2H-[1,4]dioxino[2,3-b]indol-5(3H,4aH,9bH)-yl)ethanone (3ac)
1-(3-Butyl-9b-methyl-2H-[1,4]dioxino[2,3-b]indol-5(3H,4aH,9bH)-yl)ethanone (3ac′): colorless oil was obtained in 86% isolated yield. ^{1}H NMR (400 MHz, DMSO-d_6) δ 8.04 (d, *J* = 8.0

Hz, 0.5H), 7.99 (d, J = 8.0 Hz, 0.5H), 7.36 – 7.22 (m, 2H), 7.14 (td, J = 7.2, 0.8 Hz, 0.5H), 7.08 (td, J = 7.2, 0.8 Hz, 0.5H), 5.52 (s, 0.5H), 5.35 (s, 0.5H), 3.85 – 3.67 (m, 1H), 3.61 – 3.52 (m, 0.5H), 3.41 - 3.32 (m, 1H), 3.25 (dd, J = 11.6, 9.2 Hz, 0.5H), 2.28 (s, 1.5H), 2.27 (s, 1.5H), 1.48 (s, 1.5H), 1.43 – 1.11 (m, 7.5H), 0.89 – 0.85 (m, 1.5H), 0.85 – 0.79 (m, 1.5H). ^{13}C NMR (101 MHz, DMSO-d_6) δ 170.27, 169.67, 141.08, 140.87, 134.46, 133.23, 129.11, 128.92, 124.13, 123.59, 123.32, 122.31, 116.17, 115.37, 88.87, 87.69, 78.26, 74.88, 69.28, 68.73, 65.61, 62.84, 31.68, 30.44, 26.92, 26.72, 26.55, 23.69, 23.50, 22.91, 22.07, 22.02, 13.88, 13.84. HRMS (ESI) calculated for $C_{17}H_{24}NO_3^+$ [M+H]$^+$: 290.1751; found: 290.1744.

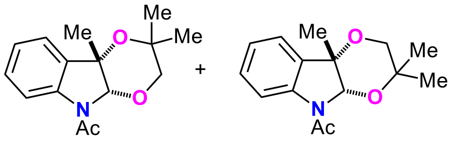

(3.4:1)

1-(2,2,9b-Trimethyl-2H-[1,4]dioxino[2,3-b]indol-5(3H,4aH,9bH)-yl)ethanone (3ad)
1-(3,3,9b-Trimethyl-2H-[1,4]dioxino[2,3-b]indol-5(3H,4aH,9bH)-yl)ethanone (3ad′): colorless oil was obtained in 82% isolated yield.^{1}H NMR (400 MHz, DMSO-d_6) δ 8.03 – 7.93 (m, 1H), 7.33 – 7.22 (m, 2H), 7.16 – 7.06 (m, 1H), 5.47 (s, 0.77H), 5.43 (s, 0.23H), 3.45 (d, J = 12.0 Hz, 0.25H), 3.41 (d, J = 12.0 Hz, 0.82H), 3.30 (d, J = 12.0 Hz, 0.24H), 3.16 (d, J = 11.2 Hz, 0.78H), 2.31 (s, 0.72H), 2.28 (s, 2.22H), 1.34 (m, 5.47H), 1.15 (s, 0.69H), 0.95 (s, 2.29H), 0.84 (s, 0.68H). ^{13}C NMR (101 MHz, DMSO) δ 169.86, 169.60, 140.69, 140.36, 135.64, 132.72, 129.09, 128.70, 124.11, 123.86, 123.49, 122.71, 116.20, 115.51, 88.96, 86.00, 77.07, 75.20, 70.85, 70.46, 68.20, 67.92, 29.13, 27.06, 26.15, 25.08, 24.54, 23.14, 22.92, 21.58. HRMS (ESI) calculated for $C_{15}H_{20}NO_3^+$ [M+H]$^+$: 262.1438; found: 262.1437.

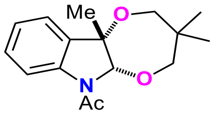

1-(3,3,10b-Trimethyl-3,4-dihydro-[1,4]dioxepino[2,3-b]indol-6(2H,5aH,10bH)-yl)ethanone (3ae): colorless oil was obtained in 64% isolated yield. ^{1}H NMR (400 MHz, DMSO-d_6) δ 8.02 (d, J = 8.0 Hz, 1H), 7.30 – 7.21 (m, 2H), 7.10 (t, J = 7.2 Hz, 1H), 5.34 (s, 1H), 3.54 – 3.16 (m, 4H), 2.26 (s, 3H), 1.40 (s, 3H), 0.95 – 0.48 (m, 6H). ^{13}C NMR (101 MHz, DMSO-d_6) δ 170.38, 141.12, 129.32, 124.37, 123.83, 115.89, 96.91, 83.96, 73.96, 67.26, 38.04, 23.50, 21.81. HRMS (ESI) calculated for $C_{16}H_{22}NO_3^+$ [M+H]$^+$: 276.1594; found: 276.1588.

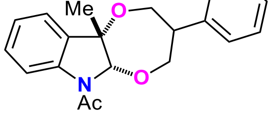

1-(10b-Methyl-3-phenyl-3,4-dihydro-[1,4]dioxepino[2,3-b]indol-6(2H,5aH,10bH)-yl)ethanone (3af): white solid was obtained in 73% isolated yield. As for the partial π-π stacking interaction of phenyl ring with indole, the spectra demonstrate a mixture of isomers (50:50) where the oxygens of both isomers stand at the *cis*-position. ^{1}H NMR (400 MHz, DMSO-d_6) δ 8.14 (d, J = 8.4 Hz, 0.5H), 8.04 – 7.98 (d, J = 7.6 Hz, 0.5H), 7.41 – 7.00 (m, 7H), 7.06 – 6.96 (m, 1H), 5.55 (s, 0.5H), 5.53 (s, 0.5H), 4.21 – 3.49 (m, 4H), 3.31 – 3.12 (m, 1H), 2.32 (s, 1.5H), 2.28 (s, 1.5H), 1.49 (s, 1.5H), 1.47 (s, 1.5H). ^{13}C NMR (101 MHz, DMSO-d_6) δ 170.59, 170.25, 141.87, 140.60,

135.22, 132.49, 129.80, 129.14, 128.98, 128.93, 128.57, 128.05, 127.51, 127.27, 124.47, 124.41, 124.11, 123.61, 116.15, 115.92, 97.63, 96.08, 84.48, 84.39, 69.51, 69.02,68.56, 48.88, 47.96, 23.66, 23.48. HRMS (ESI) calculated for $C_{20}H_{22}NO_3^+$ $[M+H]^+$: 324.1594; found: 324.1587.

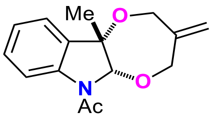

1-(10b-Methyl-3-methylene-3,4-dihydro-[1,4]dioxepino[2,3-b]indol-6(2H,5aH,10bH)-yl)ethanone (3ag): colorless oil was obtained in 71% isolated yield. ^{1}H NMR (400 MHz, DMSO-d_6) δ 8.01 (d, J = 8.0 Hz, 1H), 7.32 – 7.22 (m, 2H), 7.11 (td, J = 7.6, 1.2 Hz, 1H), 5.40 (s, 1H), 4.97 (d, J = 6.0 Hz, 2H), 4.52 – 4.15 (m, 4H), 2.28 (s, 3H), 1.41 (s, 3H). ^{13}C NMR (101 MHz, DMSO-d_6) δ 169.88, 146.01, 140.61, 133.21, 129.01, 123.97, 123.28, 115.69, 112.69, 95.55, 83.73, 69.39, 67.91, 25.83, 23.02. HRMS (ESI) calculated for $C_{15}H_{18}NO_3^+$ $[M+H]^+$: 260.1281; found: 260.1273.

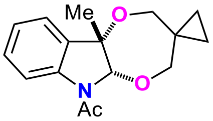

1-(10b-Methyl-2H-spiro[[1,4]dioxepino[2,3-b]indole-3,1'-cyclopropan]-6(4H,5aH,10bH)-yl)ethanone (3ah): colorless oil was obtained in 63% isolated yield. ^{1}H NMR (400 MHz, DMSO-d_6) δ 8.04 (d, J = 8.0 Hz, 1H), 7.32 – 7.20 (m, 2H), 7.17 – 7.09 (m, 1H), 5.48 (s, 1H), 3.75 – 3.54 (m, 2H), 3.40 – 3.33 (m, 2H), 2.26 (s, 3H), 1.44 (s, 3H), 0.49 – 0.42 (m, 2H), 0.41 - 0.31 (m, 2H). ^{13}C NMR (101 MHz, DMSO) δ 169.93, 140.60, 133.64, 128.83, 123.83, 123.52, 115.43, 96.28, 83.61, 73.17, 71.29, 26.22, 23.49, 23.06, 9.00, 7.82. HRMS (ESI) calculated for $C_{16}H_{20}NO_3^+$ $[M+H]^+$: 274.1438; found: 274.1435.

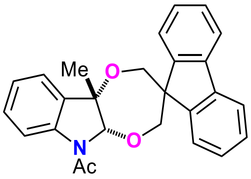

1-(10b-Methyl-2H-spiro[[1,4]dioxepino[2,3-b]indole-3,9'-fluoren]-6(4H,5aH,10bH)-yl)ethanone (3ai): white solid was obtained in 66% isolated yield. As for the partial π-π stacking interaction of phenyl ring with indole, the spectra demonstrate a mixture of isomers where the oxygens of both isomers stand at the *cis*-position. ^{1}H NMR (400 MHz, DMSO-d_6) δ 8.09 – 8.02 (m, 1H), 7.93 – 7.75 (m, 3H), 7.61 – 7.29 (m, 6H), 7.23 – 7.06 (m, 2H), 5.80 (s, 1H), 4.90 – 4.53 (m, 2H), 3.58 – 3.46 (m, 1H), 3.32 – 3.18 (m, 1H), 2.31 (s, 3H), 1.60 (s, 3H). ^{13}C NMR (101 MHz, DMSO) δ 170.34, 140.25, 139.70, 136.28, 135.63, 129.26, 128.87, 128.04, 127.79, 127.68, 127.19, 124.89, 124.70, 123.61, 120.83, 120.35, 115.89, 98.82, 98.56, 84.79, 84.72, 78.30, 76.57, 70.65, 70.63, 56.81, 56.78, 26.81, 26.31, 23.73, 23.55. HRMS (ESI) calculated for $C_{26}H_{24}NO_3^+$ $[M+H]^+$: 398.1751; found: 398.1745.

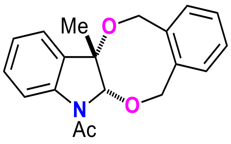

1-(13a-Methyl-5a,7,12,13a-tetrahydro-5H-benzo[6,7][1,4]dioxocino[2,3-b]indol-5-yl)ethanone (3aj): white solid was obtained in 50% isolated yield. As for the partial π-π stacking interaction of phenyl ring with indole, the spectra demonstrate a mixture of isomers where the oxygens of both isomers stand at the *cis*-position. ^{1}H NMR (400 MHz, DMSO-d_6) δ 8.23 – 8.00 (m, 1H), 7.46 – 7.36 (m, 1H), 7.34 – 7.28 (m, 1H), 7.26 – 7.06 (m, 5H), 5.51 (s, 1H), 5.31 – 5.08 (m, 1H), 5.06 – 4.91 (m, 1H), 4.85 – 4.67 (m, 1H), 4.63 – 4.45 (m, 1H), 2.37 (s, 3H), 1.51 (s, 3H). ^{13}C NMR (101 MHz, DMSO) δ 170.11, 141.13, 137.11, 136.33, 132.53, 129.38, 128.19, 127.52, 127.46, 124.15, 123.55, 115.71, 94.64, 82.44, 70.34, 69.48, 27.85, 23.20. HRMS (ESI) calculated for $C_{19}H_{20}NO_3^+$ [M+H]$^+$: 310.1438; found: 310.1431.

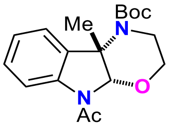

Tert-butyl 5-acetyl-9b-methyl-2,3,4a,5-tetrahydro-[1,4]oxazino[3,2-b]indole-4(9bH)-carboxylate (3ak): colorless oil was obtained in 53% isolated yield. ^{1}H NMR (400 MHz, DMSO-d_6) δ 7.97 (d, *J* = 7.6 Hz, 1H), 7.36 (dd, *J* = 7.6, 1.2 Hz, 1H), 7.25 (td, *J* = 7.6, 1.2 Hz, 1H), 7.09 (td, *J* = 7.6, 1.2 Hz, 1H), 5.32 (s, 1H), 3.88 – 3.62 (m, 3H), 2.87 – 2.70 (m, 1H), 2.30 (s, 3H), 1.73 (s, 3H), 1.50 (s, 9H). ^{13}C NMR (101 MHz, DMSO-d_6) δ 169.44, 155.41, 140.62, 134.33, 128.54, 124.12, 123.62, 116.08, 92.12, 80.28, 61.83, 61.62, 41.58, 28.01, 26.48, 22.98. HRMS (ESI) calculated for $C_{18}H_{25}N_2O_4^+$ [M+H]$^+$: 333.1809; found: 333.1793.

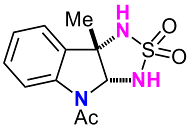

1-(8b-Methyl-2,2-dioxido-3,3a-dihydro-1H-[1,2,5]thiadiazolo[3,4-b]indol-4(8bH)-yl)ethanone (3al): white solid was obtained in 58% isolated yield. ^{1}H NMR (400 MHz, DMSO-d_6) δ 8.48 (d, *J* = 6.8 Hz, 1H), 8.02 (d, *J* = 8.0 Hz, 1H), 7.92 (s, 1H), 7.33 (d, *J* = 7.2 Hz, 1H), 7.25 (td, *J* = 7.6, 1.6 Hz, 1H), 7.09 (td, *J* = 7.6, 1.2 Hz, 1H), 5.71 (d, *J* = 6.0 Hz, 1H), 2.24 (s, 3H), 1.63 (s, 3H). ^{13}C NMR (101 MHz, DMSO) δ 169.51, 141.07, 133.69, 129.14, 123.89, 123.79, 115.72, 79.80, 67.42, 26.30, 24.06. HRMS (ESI) calculated for $C_{11}H_{14}N_3O_3S^+$ [M+H]$^+$: 268.0750; found: 268.0747.

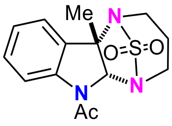

1-(10b-Methyl-11,11-dioxido-3,4-dihydro-1,5-epithio[1,4]diazepino[2,3-b]indol-6(2H,5aH,10bH)-yl)ethanone (3am): white solid was obtained in 80% yield. ^{1}H NMR (400 MHz, DMSO-d_6) δ 7.98 (d, *J* = 8.0 Hz, 1H), 7.25 (dd, *J* = 7.2, 1.6 Hz, 1H), 7.19 (td, *J* = 7.6, 1.2 Hz, 1H), 7.02 (td, *J* = 7.6, 1.2 Hz, 1H), 6.03 (s, 1H), 4.17 – 4.01 (m, 2H), 3.66 (dd, *J* = 14.4, 6.8 Hz, 1H), 3.57 (dd, *J* = 14.0, 7.6 Hz, 1H), 2.33 (s, 3H), 1.98 – 1.86 (m, 1H), 1.81 (s, 3H), 1.40 (dt, *J* = 15.6, 6.0 Hz, 1H). ^{13}C NMR (101 MHz, DMSO) δ 169.76, 141.37, 135.99, 128.56, 123.57, 122.97, 115.24, 83.99, 70.19, 53.54, 50.34, 24.05, 23.27, 14.84. HRMS (ESI) calculated for $C_{14}H_{18}N_3O_3S^+$ [M+H]$^+$: 308.1063; found: 308.1060.

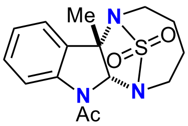

1-(11b-methyl-12,12-dioxido-4,5-dihydro-2H-1,6-epithio[1,4]diazocino[2,3-b]indol-7(3H,6aH,11bH)-yl)ethanone (3an): white solid was obtained in 65% yield. ^{1}H NMR (400 MHz, DMSO-d_6) δ 7.98 (d, J = 8.0 Hz, 1H), 7.26 (dd, J = 7.6, 1.6 Hz, 1H), 7.17 (td, J = 7.6, 1.6 Hz, 1H), 7.01 (td, J = 7.6, 1.2 Hz, 1H), 5.83 (s, 1H), 3.44 – 3.40 (m, 1H), 3.36 – 3.32 (m, 2H), 3.29 – 3.18 (m, 1H), 2.33 (s, 3H), 2.20 – 2.08 (m, 1H), 2.06 – 1.90 (m, 1H), 1.89 – 1.78 (m, 1H), 1.73 (s, 3H), 1.69 – 1.50 (m, 1H). ^{13}C NMR (101 MHz, DMSO) δ 169.80, 141.04, 136.52, 128.50, 123.58, 123.17, 115.22, 84.77, 69.86, 49.74, 43.08, 26.81, 26.02, 24.51, 24.14. HRMS (ESI) calculated for $C_{15}H_{20}N_3O_3S^+$ [M+H]$^+$: 322.1220; found: 322.1221.

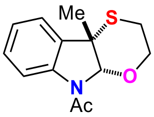

1-(9b-Methyl-2H-[1,4]oxathiino[2,3-b]indol-5(3H,4aH,9bH)-yl)ethanone (3ao): colorless oil was obtained in 66% isolated yield. ^{1}H NMR (400 MHz, DMSO-d_6) δ 7.97 (d, J = 8.0 Hz, 1H), 7.40 – 7.35 (m, 1H), 7.25 (td, J = 7.6, 1.6 Hz, 1H), 7.12 (td, J = 7.2, 1.2 Hz, 1H), 5.58 (s, 1H), 4.01 – 3.66 (m, 2H), 2.54 (dt, J = 13.6, 4.4 Hz, 1H), 2.48 – 2.40 (m, 1H), 2.30 (s, 3H), 1.37 (s, 3H). ^{13}C NMR (101 MHz, DMSO-d_6) δ 169.82, 140.70, 135.90, 128.33, 124.06, 122.92, 115.71, 91.81, 61.79, 46.76, 28.22, 23.62, 23.05. HRMS (ESI) calculated for $C_{13}H_{16}NO_2S^+$ [M+H]+: 250.0896; found: 250.0891.

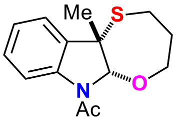

1-(10b-Methyl-3,4-dihydro-[1,4]oxathiepino[2,3-b]indol-6(2H,5aH,10bH)-yl)ethanone (3ap): colorless oil was obtained in 57% isolated yield. ^{1}H NMR (400 MHz, DMSO-d_6) δ 8.07 (d, J = 8.0 Hz, 1H), 7.32 – 7.24 (m, 2H), 7.14 (td, J = 8.0, 0.8 Hz, 1H), 5.55 (s, 1H), 3.71 – 3.63 (m, 1H), 3.35 - 3.20 (m, 1H), 2.67 – 2.56 (m, 1H), 2.37 – 2.25 (m, 4H), 1.90 – 1.73 (m, 2H), 1.45 (s, 3H). ^{13}C NMR (101 MHz, DMSO-d_6) δ 170.20, 141.33, 134.14, 128.60, 124.12, 123.33, 115.29, 97.71, 65.38, 57.83, 33.03, 29.15, 28.38, 23.22. HRMS (ESI) calculated for $C1_4H_{18}NO_2S^+$ [M+H]+: 264.1053; found: 264.1047.

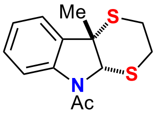

1-(9b-Methyl-2H-[1,4]dithiino[2,3-b]indol-5(3H,4aH,9bH)-yl)ethanone (3aq): colorless oil was obtained in 58% isolated yield. ^{1}H NMR (400 MHz, DMSO-d_6) δ 7.92 (d, J = 8.0 Hz, 1H), 7.37 – 7.33 (m, 1H), 7.26 (td, J = 8.0, 1.6 Hz, 1H), 7.15 (td, J = 7.6, 1.2 Hz, 1H), 5.69 (s, 1H), 2.93 – 2.84 (m, 1H), 2.82 – 2.73 (m, 1H), 2.62 (dt, J = 13.2, 5.2 Hz, 1H), 2.48 – 2.38 (m, 1H), 2.34 (s, 3H), 1.47 (s, 3H). ^{13}C NMR (101 MHz, DMSO-d_6) δ 168.79, 140.57, 135.98, 128.45, 124.37, 123.13, 116.37, 69.35, 50.72, 29.99, 25.70, 23.81, 23.75. HRMS (ESI) calculated for $C_{13}H_{16}NOS_2^+$ [M+H]$^+$: 266.0668; found: 266.0659.

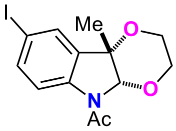

1-(8-Iodo-9b-methyl-2H-[1,4]dioxino[2,3-b]indol-5(3H,4aH,9bH)-yl)ethanone (3ba): white solid was obtained in 72% isolated yield. ^{1}H NMR (400 MHz, DMSO-d_6) δ 7.81 (d, J = 8.4 Hz, 1H), 7.64 (dd, J = 8.4, 2.0 Hz, 1H), 7.56 (d, J = 2,0 Hz, 1H), 5.40 (s, 1H), 3.76 – 3.62 (m, 2H), 3.57 (dt, J = 11.6, 2.4 Hz, 1H), 3.50 – 3.32 (m, 1H), 2.27 (s, 3H), 1.35 (s, 3H). ^{13}C NMR (101 MHz, DMSO-d_6) δ 169.89, 140.90, 137.77, 135.70, 131.19, 118.41, 87.98, 87.57, 77.18, 61.12, 60.53, 26.35, 22.95. HRMS (ESI) calculated for $C_{13}H_{15}INO_3^+$ [M+H]$^+$: 360.0091; found: 360.0088.

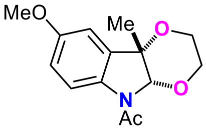

1-(8-Methoxy-9b-methyl-2H-[1,4]dioxino[2,3-b]indol-5(3H,4aH,9bH)-yl)ethanone (3ca): white solid was obtained in 74% isolated yield. ^{1}H NMR (400 MHz, $CDCl_3$) δ 8.07 (d, J = 8.8 Hz, 1H), 6.86 (d, J = 2.8 Hz, 1H), 6.82 (dd, J = 8.8, 2.8 Hz, 1H), 5.11 (s, 1H), 3.81 (s, 3H), 3.80 – 3.74 (m, 1H), 3.69 – 3.59 (m, 3H), 2.35 (s, 3H), 1.44 (s, 3H). ^{13}C NMR (101 MHz, $CDCl_3$) δ 169.21, 156.79, 134.61, 133.55, 117.89, 113.94, 108.30, 89.39, 77.85, 61.52, 60.88, 55.53, 26.82, 22.82. HRMS (ESI) calculated for $C_{14}H_{18}NO_4^+$ [M+H]$^+$: 264.1230; found: 264.1225.

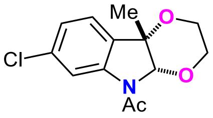

1-(7-Chloro-9b-methyl-2H-[1,4]dioxino[2,3-b]indol-5(3H,4aH,9bH)-yl)ethanone (3da): white solid was obtained in 73% isolated yield. ^{1}H NMR (400 MHz, DMSO-d_6) δ 8.01 (s, 1H), 7.30 (d, J = 8.0 Hz, 1H), 7.19 (dd, J = 8.0, 2.0 Hz, 1H), 5.46 (s, 1H), 3.75 – 3.62 (m, 2H), 3.60 – 3.54 (m, 1H), 3.43 – 3.35 (m, 1H), 2.29 (s, 3H), 1.35 (s, 3H). ^{13}C NMR (101 MHz, DMSO-d_6) δ 170.22, 142.25, 133.20, 131.89, 124.31, 123.94, 115.99, 88.27, 77.08, 61.02, 60.40, 26.32, 22.95. HRMS (ESI) calculated for $C_{13}H_{15}ClNO_3^+$ [M+H]$^+$: 268.0735; found: 268.0744.

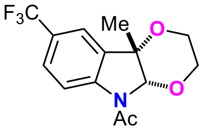

1-(9b-Methyl-8-(trifluoromethyl)-2H-[1,4]dioxino[2,3-b]indol-5(3H,4aH,9bH)-yl)ethanone (3ea): white solid was obtained in 45% isolated yield. ^{1}H NMR (400 MHz, $CDCl_3$) δ 8.26 (d, J = 8.4 Hz, 1H), 7.59 (dd, J = 8.4, 2.0 Hz, 1H), 7.55 (d, J = 2.0 Hz, 1H), 5.16 (s, 1H), 3.84 – 3.77 (m, 1H), 3.75 – 3.69 (m, 1H), 3.68 – 3.60 (m, 2H), 2.40 (s, 3H), 1.46 (s, 3H). ^{13}C NMR (101 MHz, $CDCl_3$) δ 170.28, 143.81, 133.04, 127.15 (q, $^3J_{C\text{-}F}$ = 4.1 Hz), 126.47 (q, $^2J_{C\text{-}F}$ = 32.3 Hz), 124.08 (q, $^1J_{C\text{-}F}$ = 272.7 Hz), 119.76 (q, $^4J_{C\text{-}F}$ = 3.1 Hz), 117.04, 89.29, 77.63, 61.71, 61.03, 26.85, 23.20. ^{19}F NMR (377 MHz, $CDCl_3$) δ -61.65. HRMS (ESI) calculated for $C_{14}H_{15}F_3NO_3^+$ [M+H]$^+$: 302.0999; found: 302.1003.

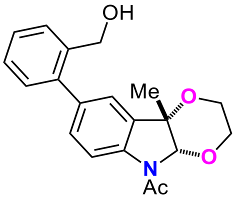

1-(8-(2-(Hydroxymethyl)phenyl)-9b-methyl-2H-[1,4]dioxino[2,3-b]indol-5(3H,4aH,9bH)-yl)ethanone (3fa): yellow oil was obtained in 65% isolated yield. ^{1}H NMR (400 MHz, DMSO-d_6) δ 8.04 (d, J = 8.4 Hz, 1H), 7.57 (dd, J = 8.0, 1.6 Hz, 1H), 7.40 – 7.29 (m, 4H), 7.26 (dd, J = 7.2, 1.2 Hz, 1H), 5.46 (s, 1H), 5.19 (t, J = 5.2 Hz, 1H), 4.49 – 4.35 (m, 2H), 3.79 – 3.57 (m, 3H), 3.55 – 3.44 (m, 1H), 2.31 (s, 3H), 1.40 (s, 3H). ^{13}C NMR (101 MHz, DMSO-d_6) δ 169.73, 140.20, 139.72, 139.24, 136.23, 132.77, 129.90, 129.43, 128.34, 127.25, 126.99, 123.36, 115.84, 88.34, 77.52, 61.22, 60.87, 60.43, 26.49, 22.97. HRMS (ESI) calculated for $C_{20}H_{22}NO_4^+$ [M+H]$^+$: 340.1543; found: 340.1534.

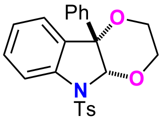

9b-Phenyl-5-tosyl-3,4a,5,9b-tetrahydro-2H-[1,4]dioxino[2,3-b]indole (3ga): white solid was obtained in 69% yield. ^{1}H NMR (400 MHz, DMSO-d_6) δ 7.67 – 7.63 (m, 2H), 7.48 – 7.38 (m, 2H), 7.29 – 7.23 (m, 3H), 7.21 – 7.15 (m, 4H), 6.94 – 6.88 (m, 2H), 5.52 (s, 1H), 3.97 – 3.89 (m, 1H), 3.80 (dt, J = 11.6, 3.6 Hz, 1H), 3.65 – 3.48 (m, 2H), 2.33 (s, 3H). ^{13}C NMR (101 MHz, DMSO) δ 144.41, 141.19, 140.89, 134.98, 130.42, 130.32, 129.75, 128.24, 128.06, 127.16, 126.10, 125.43, 124.36, 113.49, 91.24, 81.61, 60.18, 60.10, 21.02. HRMS (ESI) calculated for $C_{23}H_{22}NO_4S^+$ [M+H]$^+$: 408.1264; found: 408.1262.

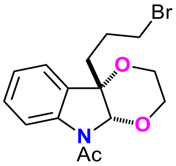

1-(9b-(3-Bromopropyl)-2H-[1,4]dioxino[2,3-b]indol-5(3H,4aH,9bH)-yl)ethanone (3ha): white solid was obtained in 74% isolated yield. ^{1}H NMR (400 MHz, DMSO-d_6) δ 8.02 (d, J = 8.0 Hz, 1H), 7.36 – 7.24 (m, 2H), 7.14 (td, J = 7.6, 1.2 Hz, 1H), 5.46 (s, 1H), 3.76 – 3.64 (m, 2H), 3.59 – 3.52 (m, 1H), 3.51 – 3.38 (m, 3H), 2.30 (s, 3H), 1.94 – 1.88 (m, 1H), 1.86 – 1.75 (m, 2H), 1.70 – 1.61 (m, 1H). ^{13}C NMR (101 MHz, DMSO-d_6) δ 169.67, 141.41, 130.92, 129.26, 123.85, 123.63, 116.27, 87.13, 79.30, 61.12, 60.45, 37.22, 35.08, 26.06, 22.97. HRMS (ESI) calculated for $C_{15}H_{19}BrNO_3^+$ [M+H]$^+$: 340.0543; found: 340.0543.

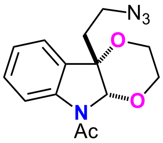

1-(9b-(2-Azidoethyl)-2H-[1,4]dioxino[2,3-b]indol-5(3H,4aH,9bH)-yl)ethanone (3ia): colorless oil was obtained in 72% yield. ^{1}H NMR (400 MHz, DMSO-d_6) δ 8.02 (d, J = 8.0 Hz, 1H), 7.37 – 7.27

(m, 2H), 7.15 (t, *J* = 7.6 Hz, 1H), 5.54 (s, 1H), 3.79 – 3.63 (m, 2H), 3.56 (dt, *J* = 11.6, 2.4 Hz, 1H), 3.53 – 3.33 (m, 3H), 2.30 (s, 3H), 2.03 – 1.81 (m, 2H). ^{13}C NMR (101 MHz, DMSO) δ 169.58, 141.42, 130.41, 129.41, 123.96, 123.52, 116.21, 86.57, 78.66, 60.91, 60.34, 45.46, 37.32, 22.90. HRMS (ESI) calculated for $C_{14}H_{17}N_4O_3^+$ [M+H]$^+$: 289.1295; found: 289.1290.

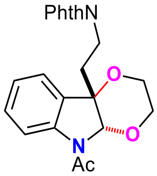

2-(2-(5-Acetyl-3,4a,5,9b-tetrahydro-2H-[1,4]dioxino[2,3-b]indol-9b-yl)ethyl)isoindoline-1,3-dione (3ja): white solid was obtained in 70% isolated yield. As for the partial π-π stacking interaction of carbonyl group with indole, the spectra demonstrate a mixture of isomers (50:50) where the oxygens of both isomers stand at the *cis*-position. ^{1}H NMR (400 MHz, DMSO-d_6) δ 8.02 (d, *J* = 8.0 Hz, 1H), 7.64 – 7.54 (m, 3H), 7.53 – 7.47 (m, 1H), 7.39 – 7.29 (m, 2H), 7.21 – 7.13 (m, 1H), 6.61 (d, *J* = 8.4 Hz, 0.5H), 6.56 (d, *J* = 8.4 Hz, 0.5H), 5.78 (d, *J* = 5.2 Hz, 0.5H), 5.76 (d, *J* = 5.2 Hz, 0.5H), 5.56 (s, 0.5H), 5.55 (s, 0.5H), 3.89 – 3.74 (m, 1H), 3.74 – 3.63 (m, 1H), 3.64 – 3.55 (m, 1H), 3.51 – 3.43 (m, 1H), 2.30 (s, 1.5H), 2.28 (s, 1.5H), 2.10 – 1.81 (m, 2H). ^{13}C NMR (101 MHz, DMSO) δ 174.593, 174.360, 169.61, 169.54, 165.94, 165.92, 145.00, 144.98, 141.38, 141.32, 131.92, 131.64, 130.96, 130.66, 129.31, 129.30, 124.02, 123.72, 123.63, 123.50, 122.20, 116.45, 116.41, 86.87, 86.77, 80.82, 80.63, 79.16, 79.09, 61.31, 60.50, 36.69, 33.86, 33.67, 22.97, 22.90. HRMS (ESI) calculated for $C_{22}H_{21}N_2O_5^+$ [M+H]$^+$: 393.1445; found: 393.1445.

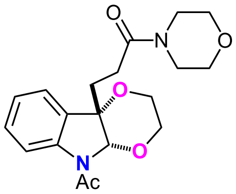

3-(5-Acetyl-3,4a,5,9b-tetrahydro-2H-[1,4]dioxino[2,3-b]indol-9b-yl)-1-morpholinopropan-1-one (3ka): white solid was obtained in 86% isolated yield. ^{1}H NMR (400 MHz, DMSO-d_6) δ 8.02 (d, *J* = 8.4 Hz, 1H), 7.35 – 7.28 (m, 2H), 7.14 (td, *J* = 7.2, 0.8 Hz, 1H), 5.49 (s, 1H), 3.79 – 3.63 (m, 2H), 3.59 – 3.39 (m, 10H), 2.59 – 2.46 (m, 1H), 2.29 (s, 3H), 2.27 – 2.19 (m, 1H), 1.95 – 1.78 (m, 2H). ^{13}C NMR (101 MHz, DMSO-d_6) δ 170.19, 169.67, 141.44, 131.05, 129.27, 123.93, 123.59, 116.21, 86.89, 79.36, 66.03, 66.01, 61.16, 60.55, 45.19, 41.48, 34.04, 25.88, 22.93. HRMS (ESI) calculated for $C_{19}H_{25}N_2O_5^+$ [M+H]$^+$: 361.1758; found: 361.1749.

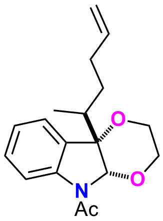

1-(9b-(Hex-5-en-2-yl)-2H-[1,4]dioxino[2,3-b]indol-5(3H,4aH,9bH)-yl)ethanone (3la): white solid was obtained in 64% isolated yield. As for the partial π-π stacking interaction of alkene with

indole, the spectra demonstrate a mixture of isomers (56:44) where the oxygens of both isomers stand at the *cis*-position. ^{1}H NMR (400 MHz, $CDCl_3$) δ 8.18 (dd, *J* = 8.4, 2.4 Hz, 1H), 7.33 (td, *J* = 7.6, 1.2 Hz, 1H), 7.25 (dd, *J* = 7.6, 3.2 Hz, 1H), 7.17 – 7.10 (m, 1H), 5.83 – 5.62 (m, 1H), 5.33 (s, 0.56H), 5.29 (s, 0.44H), 5.03 – 4.91 (m, 2H), 3.78 – 3.66 (m, 2H), 3.59 – 3.48 (m, 2H), 2.37 (s, 1.82H), 2.37 (s, 1.16H), 2.23 – 2.10 (m, 1H), 1.98 – 1.74 (m, 3H), 1.17 – 1.04 (m, 1H), 0.87 (d, *J* = 6.8 Hz, 1.46H), 0.80 (d, *J* = 6.8 Hz, 1.60H). ^{13}C NMR (101 MHz, $CDCl_3$) δ 169.69, 169.63, 142.44, 142.39, 138.26, 138.14, 129.69, 129.41, 128.84, 124.54, 124.21, 123.92, 123.83, 116.60, 116.58, 114.98, 114.79, 86.79, 86.25, 81.98, 81.81, 59.89, 59.78, 59.31, 59.01, 40.74, 40.53, 31.53, 29.22, 29.13, 23.31, 23.27, 13.07, 12.92. HRMS (ESI) calculated for $C_{18}H_{24}NO_3^+$ $[M+H]^+$: 302.1751; found: 302.1740.

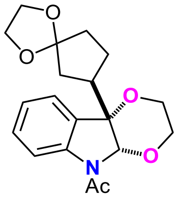

1-(9b-(1,4-Dioxaspiro[4.4]nonan-7-yl)-2H-[1,4]dioxino[2,3-b]indol-5(3H,4aH,9bH)-yl)ethanone (3ma): white solid was obtained in 65% isolated yield. As for the partial π-π stacking interaction of carbonyl group with indole, the spectra demonstrate a mixture of isomers (55:45) where the oxygens of both isomers stand at the *cis*-position. ^{1}H NMR (400 MHz, DMSO-d_6) δ 8.01 (d, *J* = 8.0 Hz, 1H), 7.31 (td, *J* = 7.6, 1.2 Hz, 1H), 7.26 (dt, *J* = 7.6, 1.6 Hz, 1H), 7.13 (tt, *J* = 7.2, 1.2 Hz, 1H), 5.48 (s, 0.55H), 5.43 (s, 0.45), 3.87 – 3.64 (m, 6H), 3.56 – 3.48 (m, 1H), 3.45 – 3.37 (m, 1H), 2.29 (s, 3H), 2.27 – 2.17 (m, 1H), 1.94 – 1.29 (m, 6H). ^{13}C NMR (101 MHz, DMSO-d_6) δ 169.47, 169.45, 141.84, 141.79, 129.98, 129.87, 129.29, 124.23, 124.10, 123.81, 123.72, 116.28, 116.19, 116.06, 116.03, 86.59, 86.35, 80.57, 80.50, 63.81, 63.68, 63.52, 63.50, 60.79, 60.73, 60.38, 44.67, 44.32, 36.09, 35.87, 35.08, 34.97, 23.29, 23.14, 22.95. HRMS (ESI) calculated for $C_{19}H_{24}NO_5^+$ $[M+H]^+$: 346.1649; found: 346.1642.

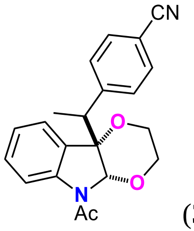

(**3la**: **3la′**) = 43:57

4-(1-(5-Acetyl-3,4a,5,9b-tetrahydro-2H-[1,4]dioxino[2,3-b]indol-9b-yl)ethyl)benzonitrile (3na): white solid of **3la** and **3la′** was obtained in 72% isolated yield. No π-π stacking interaction was observed with **3la** and π-π stacking interaction of phenyl ring with indole was observed in **3sa′** (43:57) where the oxygens of both isomers stand at the *cis*-position. ^{1}H NMR (400 MHz, DMSO-d_6) δ 7.87 (d, *J* = 8.0 Hz, 1H), 7.71 – 7.65 (m, 2H), 7.37 – 7.31 (m, 2H), 7.32 - 7.21 (m, 1H), 7.24 – 7.20 (m, 1H), 7.18 – 7.12 (m, 1H), 5.28 (s, 1H), 3.74 (dt, *J* = 10.8, 2.4 Hz, 1H), 3.50 – 3.39 (m, 3H), 3.07 (q, *J* = 6.8 Hz, 1H), 2.15 (s, 3H), 1.29 (d, *J* = 7.2 Hz, 3H). ^{13}C NMR (101 MHz, DMSO-d_6) δ 168.79, 147.01, 141.69, 131.54, 130.05, 129.69, 129.57, 124.59, 123.63, 118.93, 116.13, 109.60, 85.45, 81.54, 60.81, 60.77, 46.59, 22.86, 14.23. HRMS (ESI) calculated for $C_{21}H_{21}N_2O_3^+$ $[M+H]^+$: 349.1547; found: 349.1563.

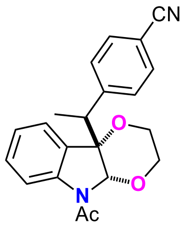

4-(1-(5-Acetyl-3,4a,5,9b-tetrahydro-2H-[1,4]dioxino[2,3-b]indol-9b-yl)ethyl)benzonitrile (3na′): ^{1}H NMR (400 MHz, DMSO-d_6) δ 7.81 (d, J = 8.0 Hz, 1H), 7.56 (d, J = 8.0 Hz, 2H), 7.27 (td, J = 7.6, 1.2 Hz, 1H), 7.04 (t, J = 7.2 Hz, 1H), 6.97 (d, J = 8.0 Hz, 2H), 6.94 (d, J = 7.2 Hz, 1H), 5.56 (s, 1H), 3.84 – 3.68 (m, 2H), 3.58 – 3.51 (m, 1H), 3.48 – 3.38 (m, 1H), 3.25 (q, J = 6.8 Hz, 1H), 2.09 (s, 3H), 1.32 (d, J = 6.8 Hz, 3H). ^{13}C NMR (101 MHz, DMSO-d_6) δ 168.52, 146.72, 142.20, 131.35, 129.57, 129.37, 128.21, 124.74, 123.35, 118.89, 116.06, 109.51, 86.36, 81.62, 60.97, 60.65, 46.62, 22.66, 14.72. HRMS (ESI) calculated for $C_{21}H_{21}N_2O_3^+$ [M+H]$^+$: 349.1547; found: 349.1553.

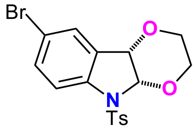

8-Bromo-5-tosyl-3,4a,5,9b-tetrahydro-2H-[1,4]dioxino[2,3-b]indole (3oa): colorless oil was obtained in 58% isolated yield. ^{1}H NMR (400 MHz, DMSO-d_6) δ 7.87 – 7.81 (m, 2H), 7.51 – 7.47 (m, 1H), 7.46 – 7.44 (m, 1H), 7.43 – 7.38 (m, 2H), 7.30 (d, J = 8.5 Hz, 1H), 5.61 (d, J = 5.2 Hz, 1H), 5.08 (d, J = 5.2 Hz, 1H), 3.77 – 3.67 (m, 1H), 3.64 – 3.46 (m, 3H), 2.35 (s, 3H). ^{13}C NMR (101 MHz, DMSO) δ 145.15, 140.12, 135.13, 132.87, 131.73, 130.39, 128.48, 128.09, 116.11, 115.91, 86.26, 72.18, 61.39, 60.81, 21.51. HRMS (ESI) calculated for $C_{17}H_{17}BrNO_4S^+$ [M+H]$^+$: 410.0056; found: 410.0050.

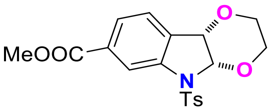

Methyl 5-tosyl-3,4a,5,9b-tetrahydro-2H-[1,4]oxathiino[2,3-b]indole-8-carboxylate (3pa): colorless oil was obtained in 55% isolated yield. ^{1}H NMR (400 MHz, DMSO-d_6) δ 7.86 – 7.81 (m, 3H), 7.72 (dd, J = 7.6, 1.6 Hz, 1H), 7.46 (d, J = 7.6 Hz, 1H), 7.41 (d, J = 8.0 Hz, 2H), 5.68 (d, J = 4.8 Hz, 1H), 5.15 (d, J = 4.8 Hz, 1H), 3.87 (s, 3H), 3.76 – 3.68 (m, 1H), 3.66 – 3.53 (m, 2H), 3.51 - 3.41 (m, 1H), 2.35 (s, 3H). ^{13}C NMR (101 MHz, DMSO) δ 165.60, 144.79, 140.65, 134.88, 134.12, 130.98, 130.04, 127.44, 125.54, 125.24, 113.49, 85.74, 72.08, 61.13, 60.44, 52.55, 21.06. HRMS (ESI) calculated for $C_{19}H_{20}NO_6S^+$ [M+H]$^+$: 390.1006; found: 390.0994.

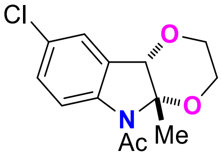

1-(8-Chloro-4a-methyl-2H-[1,4]dioxino[2,3-b]indol-5(3H,4aH,9bH)-yl)ethanone (3qa): colorless oil was obtained in 63% isolated yield. ^{1}H NMR (400 MHz, DMSO-d_6) δ 8.15 (d, J = 8.4 Hz, 1H), 7.50 (d, J = 2.0 Hz, 1H), 7.38 (dd, J = 8.8, 2.4 Hz, 1H), 4.45 (s, 1H), 3.80 - 3.73 (m, 1H), 3.72 – 3.63 (m, 3H), 2.29 (s, 3H), 1.43 (s, 3H). ^{13}C NMR (101 MHz, DMSO) δ 169.79, 141.63, 130.00, 129.64, 126.72, 125.90, 118.22, 92.15, 75.10, 61.57, 60.34, 23.99, 23.51. HRMS (ESI) calculated for $C_{13}H_{15}ClNO_3^+$ [M+H]$^+$: 268.0735; found: 268.0733.

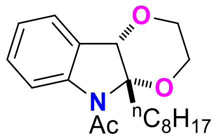

1-(4a-Octyl-2H-[1,4]dioxino[2,3-b]indol-5(3H,4aH,9bH)-yl)ethanone (3ra): colorless oil was obtained in 58% isolated yield. ^{1}H NMR (400 MHz, DMSO-d_6) δ 8.13 (d, J = 8.0 Hz, 1H), 7.39 (dd, J = 7.2, 1.2 Hz, 1H), 7.31 (td, J = 8.0, 1.2 Hz, 1H), 7.10 – 7.02 (m, 1H), 4.61 (s, 1H), 3.83 – 3.73 (m, 1H), 3.69 – 3.51 (m, 3H), 2.28 (s, 3H), 1.31 – 1.13 (m, 14H), 0.87 – 0.79 (m, 3H). ^{13}C NMR (101 MHz, DMSO) δ 169.57, 143.17, 129.81, 127.99, 125.72, 123.41, 116.59, 93.78, 73.50, 60.88, 60.13, 37.33, 31.20, 28.96, 28.75, 28.54, 23.54, 23.10, 22.05, 13.95. HRMS (ESI) calculated for $C_{20}H_{30}NO_3^+$ [M+H]$^+$: 332.2220; found: 332.2219.

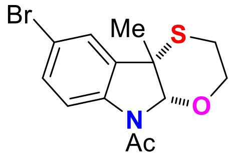

8-Bromo-9b-methyl-2H-[1,4]oxathiino[2,3-b]indol-5(3H,4aH,9bH)-yl)ethanone (3sa): colorless oil was obtained in 71% isolated yield. ^{1}H NMR (400 MHz, DMSO-d_6) δ 7.92 (d, J = 8.4 Hz, 1H), 7.50 (d, J = 2.0 Hz, 1H), 7.44 (dd, J = 8.4, 2.4 Hz, 1H), 5.62 (s, 1H), 3.91 – 3.71 (m, 2H), 2.64 – 2.43 (m, 2H), 2.30 (s, 3H), 1.39 (s, 3H). ^{13}C NMR (101 MHz, DMSO) δ 170.01, 140.06, 138.83, 131.17, 125.87, 117.62, 115.58, 91.84, 61.84, 46.49, 27.93, 23.59, 22.97. HRMS (ESI) calculated for $C_{13}H_{15}BrNO_2S^+$ [M+H]$^+$: 328.0001; found: 327.9988.

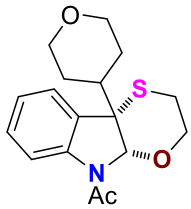

1-(9b-(Tetrahydro-2H-pyran-4-yl)-2H-[1,4]oxathiino[2,3-b]indol-5(3H,4aH,9bH)-yl)ethanone (3ta): colorless oil was obtained in 73% isolated yield. ^{1}H NMR (400 MHz, DMSO-d_6) δ 8.01 (d, J = 8.0 Hz, 1H), 7.35 – 7.25 (m, 2H), 7.14 (td, J = 7.6, 1.2 Hz, 1H), 5.77 (s, 1H), 3.88 – 3.64 (m, 3H), 3.49 – 3.41 (m, 1H), 3.24 - 3.14 (m, 2H), 2.78 – 2.67 (m, 1H), 2.36 – 2.32 (m, 1H), 2.31 (s, 3H), 2.02 – 1.89 (m, 1H), 1.60 – 1.47 (m, 2H), 1.47 – 1.32 (m, 1H), 1.10 – 0.96 (m, 1H). ^{13}C NMR (101 MHz, DMSO) δ 170.18, 142.45, 129.19, 125.64, 124.16, 115.65, 89.36, 67.35, 67.23, 58.28, 55.94, 43.90, 27.47, 27.41, 23.72, 23.22. HRMS (ESI) calculated for $C_{17}H_{22}NO_3S^+$ [M+H]$^+$: 320.1315; found: 320.1308.

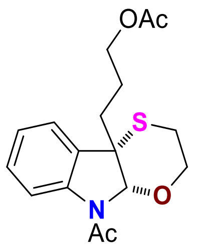

3-(5-Acetyl-3,4a,5,9b-tetrahydro-2H-[1,4]oxathiino[2,3-b]indol-9b-yl)propyl acetate (3ua): colorless oil was obtained in 76% isolated yield. ^{1}H NMR (400 MHz, DMSO-d_6) δ 7.99 (d, *J* = 8.0 Hz, 1H), 7.39 – 7.31 (m, 1H), 7.27 (td, *J* = 8.0, 1.2 Hz, 1H), 7.13 (td, *J* = 7.2, 1.2 Hz, 1H), 5.64 (s, 1H), 3.92 – 3.83 (m, 2H), 3.82 – 3.74 (m, 1H), 3.73 – 3.66 (m, 1H), 2.61 (dt, *J* = 13.2, 4.8 Hz, 1H), 2.47 – 2.36 (m, 1H), 2.30 (s, 3H), 1.94 (s, 3H), 1.82 – 1.73 (m, 1H), 1.72 - 1.64 (m, 1H), 1.58 – 1.43 (m, 2H). ^{13}C NMR (101 MHz, DMSO) δ 170.34, 169.83, 141.26, 133.69, 128.58, 124.15, 123.84, 115.72, 90.94, 63.56, 60.85, 50.69, 36.32, 23.17, 23.12, 22.82, 20.71. HRMS (ESI) calculated for $C_{17}H_{22}NO_4S^+$ $[M+H]^+$: 336.1263; found: 336.1251.

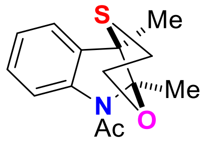

1-(4a,9b-Dimethyl-2H-[1,4]oxathiino[2,3-b]indol-5(3H,4aH,9bH)-yl)ethanone (3va): colorless oil was obtained in 68% isolated yield. ^{1}H NMR (400 MHz, DMSO-d_6) δ 8.09 (d, *J* = 8.0 Hz, 1H), 7.29 (dd, *J* = 7.6, 1.2 Hz, 1H), 7.25 – 7.19 (m, 1H), 7.05 (td, *J* = 7.6, 1.2 Hz, 1H), 4.05 (dt, *J* = 12.0, 3.6 Hz, 1H), 3.84 – 3.70 (m, 1H), 2.89 – 2.79 (m, 1H), 2.46 – 2.37 (m, 1H), 2.33 (s, 3H), 1.70 (s, 3H), 1.43 (s, 3H). ^{13}C NMR (101 MHz, DMSO) δ 169.96, 140.63, 133.78, 128.46, 123.61, 121.89, 116.53, 97.34, 62.04, 47.62, 23.47, 22.96, 22.49, 20.55. HRMS (ESI) calculated for $C_{14}H_{18}NO_2S^+$ $[M+H]^+$: 264.1053; found: 264.1044.

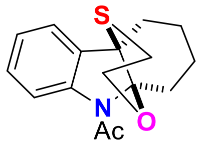

1-7,8-Dihydro-5H-8a,4b-(epoxyethanothio)carbazol-9(6H)-yl)ethanone (3wa): white solid was obtained in 61% isolated yield. 1H NMR (400 MHz, DMSO-d_6) δ 8.14 (d, *J* = 8.0 Hz, 1H), 7.29 (dd, *J* = 7.6, 1.2 Hz, 1H), 7.26 – 7.21 (m, 1H), 7.05 (td, *J* = 7.6, 1.2 Hz, 1H), 4.20 – 4.11 (m, 1H), 3.89 (td, *J* = 11.6, 2.0 Hz, 1H), 3.06 – 2.95 (m, 1H), 2.49 – 2.39 (m, 2H), 2.30 (s, 3H), 2.30 – 2.22 (m, 1H), 2.14 -2.06 (m, 1H), 1.66 – 1.56 (m, 1H), 1.47 – 1.35 (m, 3H), 1.27 – 1.06 (m, 1H). ^{13}C NMR (101 MHz, DMSO) δ 169.94, 141.19, 131.81, 128.59, 123.51, 121.29, 117.15, 96.06, 62.73, 47.63, 35.62, 30.26, 23.16, 22.71, 22.67, 20.69. HRMS (ESI) calculated for $C_{16}H_{20}NO_2S^+$ $[M+H]^+$: 290.1209; found: 290.1203.

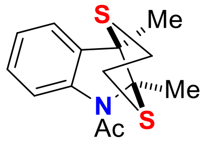

1-(4a,9b-Dimethyl-2H-[1,4]dithiino[2,3-b]indol-5(3H,4aH,9bH)-yl)ethanone (3xa): colorless oil was obtained in 52% isolated yield. ^{1}H NMR (400 MHz, DMSO-d_6) δ 7.75 – 7.65 (m, 1H), 7.43 – 7.37 (m, 1H), 7.28 – 7.22 (m, 1H), 7.17 – 7.12 (m, 1H), 2.98 – 2.84 (m, 1H), 2.82 – 2.71 (m, 1H), 2.71 – 2.61 (m, 2H), 2.43 (s, 3H), 2.02 (s, 3H), 1.33 (s, 3H). ^{13}C NMR (101 MHz, DMSO) δ 169.46, 140.95, 136.15, 128.20, 124.04, 122.45, 116.70, 74.85, 54.49, 27.93, 26.57, 25.61, 25.21, 22.39. HRMS (ESI) calculated for $C_{14}H_{18}NOS_2^+$ [M+H]$^+$: 280.0824; found: 280.0815.

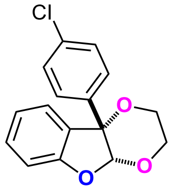

9b-(4-Chlorophenyl)-2,3,4a,9b-tetrahydro-[1,4]dioxino[2,3-b]benzofuran (5a): white solid was obtained in 52% isolated yield. ^{1}H NMR (400 MHz, DMSO-d_6) δ 7.57 – 7.45 (m, 4H), 7.37 – 7.28 (m, 1H), 7.00 (d, J = 8.0 Hz, 1H), 6.91 (td, J = 7.2, 0.8 Hz, 1H), 6.86 (dd, J = 7.2, 1.6 Hz, 1H), 6.11 (s, 1H), 3.99 – 3.89 (m, 1H), 3.80 – 3.60 (m, 3H). ^{13}C NMR (101 MHz, DMSO-d_6) δ 157.31, 137.92, 132.63, 130.88, 130.20, 128.57, 128.41, 125.04, 121.64, 110.35, 104.52, 78.26, 59.19, 59.04. HRMS (ESI) calculated for $C_{16}H_{14}FO_3^+$ [M+H]$^+$: 289.0626; found: 289.0631.

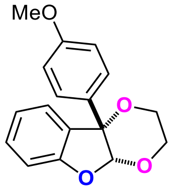

9b-(4-Methoxyphenyl)-2,3,4a,9b-tetrahydro-[1,4]dioxino[2,3-b]benzofuran (5b): white solid was obtained in 55% isolated yield. ^{1}H NMR (400 MHz, DMSO-d_6) δ 7.45 – 7.39 (m, 2H), 7.30 - 7.26 (m, 1H), 7.02 – 6.95 (m, 3H), 6.93 – 6.84 (m, 2H), 6.07 (s, 1H), 3.97 – 3.90 (m, 1H), 3.77 (s, 3H), 3.76 – 3.71 (m, 1H), 3.70 – 3.66 (m, 1H), 3.64 – 3.57 (m, 1H). ^{13}C NMR (101 MHz, DMSO-d_6) δ 158.78, 157.26, 130.78, 130.57, 130.51, 127.72, 125.07, 121.46, 113.92, 110.27, 104.68, 78.21, 59.19, 59.07, 55.15.HRMS (ESI) calculated for $C_{17}H_{17}O_4^+$ [M+H]$^+$: 285.1121; found: 285.1131.

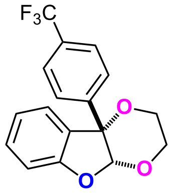

9b-(4-(Trifluoromethyl)phenyl)-2,3,4a,9b-tetrahydro-[1,4]dioxino[2,3-b]benzofuran (5c): white solid was obtained in 51% isolated yield. ^{1}H NMR (400 MHz, DMSO-d_6) δ 7.81 (d, J = 8.4 Hz, 2H), 7.74 (d, J = 8.4 Hz, 2H), 7.37 – 7.30 (m, 1H), 7.02 (dt, J = 8.0, 0.8 Hz, 1H), 6.92 (td, J = 7.2, 0.8 Hz, 1H), 6.86 (dd, J = 7.6, 1.2 Hz, 1H), 6.17 (s, 1H), 4.00 – 3.92 (m, 1H), 3.80 – 3.64 (m, 3H). ^{13}C NMR (101 MHz, DMSO-d_6) δ 157.38, 143.77, 131.05, 130.02, 128.47 (q, $^2J_{C-F}$ = 32.2 Hz), 127.37, 125.48 (q, $^3J_{C-F}$ = 4.0 Hz), 125.10, 124.18 (q, $^1J_{C-F}$ = 273.11 Hz), 121.75, 110.40, 104.62, 78.52, 59.28, 58.98. ^{19}F NMR (377 MHz, DMSO-d_6) δ -61.06. HRMS (ESI) calculated for $C_{17}H_{14}F_3O3^+$ [M+H]$^+$: 323.0890; found: 323.0880.

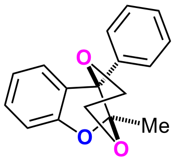

4a-Methyl-9b-phenyl-2,3,4a,9b-tetrahydro-[1,4]dioxino[2,3-b]benzofuran (5d): colorless oil was obtained in 71% isolated yield. ^{1}H NMR (400 MHz, DMSO-d_6) δ 7.41 – 7.28 (m, 4H), 7.22 – 7.18 (m, 2H), 7.11 (dd, *J* = 7.6, 1.6 Hz, 1H), 7.05 (td, *J* = 7.2, 1.2 Hz, 1H), 7.00 (d, *J* = 8.0 Hz, 1H), 3.98 – 3.89 (m, 1H), 3.88- 3.80 (m, 1H), 3.76 – 3.68 (m, 1H), 3.59 – 3.51 (m, 1H), 1.00 (s, 3H). ^{13}C NMR (101 MHz, DMSO-d_6) δ 158.29, 140.53, 130.94, 127.98, 127.95, 127.50, 126.65, 125.80, 122.04, 110.26, 109.08, 85.18, 59.80, 58.48, 22.06. HRMS (ESI) calculated for $C_{17}H_{17}O_3^+$ [M+H]$^+$: 269.1172; found: 269.1162.

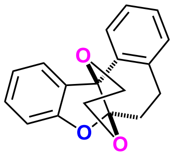

5,6-Dihydro-6a,11b-(epoxyethanooxy)naphtho[2,1-b]benzofuran (5e): colorless oil was obtained in 87% isolated yield. ^{1}H NMR (400 MHz, DMSO-d_6) δ 7.83 (dd, *J* = 7.6, 1.6 Hz, 1H), 7.39 (td, *J* = 7.6, 1.2 Hz, 1H), 7.30 (td, *J* = 7.6, 1.2 Hz, 1H), 7.27 – 7.16 (m, 3H), 6.92 (dt, *J* = 8.0, 0.8 Hz, 1H), 6.87 (td, *J* = 7.6, 0.8 Hz, 1H), 4.05 – 3.97 (m, 1H), 3.77 – 3.69 (m, 1H), 3.53 – 3.47 (m, 1H), 3.34 – 3.24 (m, 1H), 2.99 – 2.87 (m, 1H), 2.68 (dt, *J* = 16.8, 4.8 Hz, 1H), 2.08 (dt, *J* = 12.8, 4.4 Hz, 1H), 1.94 – 1.84 (m, 1H). ^{13}C NMR (101 MHz, DMSO-d_6) δ 157.19, 136.61, 134.42, 131.38, 130.28, 128.27, 128.04, 127.54, 126.90, 124.89, 120.84, 110.60, 107.25, 75.64, 60.42, 58.96, 31.69, 25.70. HRMS (ESI) calculated for $C_{18}H_{17}O_3^+$ [M+H]$^+$: 281.1172; found: 281.1173.

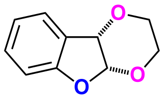

2,3,4a,9b-Tetrahydro-[1,4]dioxino[2,3-b]benzofuran (5f): white solid was obtained in 36% isolated yield. ^{1}H NMR (400 MHz, DMSO-d_6) δ 7.41 - 7.37 (m, 1H), 7.29 (td, *J* = 8.0, 1.6 Hz, 1H), 6.97 – 6.88 (m, 2H), 5.63 (dt, *J* = 4.0, 0.8 Hz, 1H), 4.76 (d, *J* = 4.0 Hz, 1H), 3.92 – 3.80 (m, 1H), 3.79 – 3.57 (m, 3H). ^{13}C NMR (101 MHz, DMSO-d_6) δ 157.87, 130.78, 126.84, 126.22, 120.96, 110.19, 100.76, 70.03, 61.32, 60.38. HRMS (ESI) calculated for $C_{10}H_{11}O_3^+$ [M+H]$^+$: 179.0703; found: 179.0707.

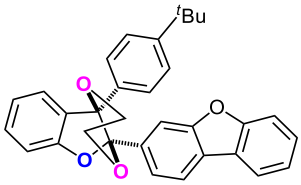

9b-(4-(Tert-butyl)phenyl)-4a-(dibenzo[b,d]furan-3-yl)-2,3,4a,9b-tetrahydro-[1,4]dioxino[2,3-b]benzofuran (5g): white solid was obtained in 58% isolated yield. ^{1}H NMR (400 MHz, DMSO-d_6) δ 7.90 – 7.86 (m, 1H), 7.60 – 7.48 (m, 3H), 7.46 – 7.41 (m, 1H), 7.32 – 7.22 (m, 3H), 7.12 –

7.08 (m, 2H), 7.01 (dd, *J* = 8.8, 2.0 Hz, 1H), 6.95 – 6.91 (m, 2H), 6.90 – 6.85 (m, 2H), 4.29 – 4.19 (m, 1H), 4.15 – 4.07 (m, 1H), 4.01 – 3.94 (m, 1H), 3.77 – 3.67 (m, 1H), 0.83 (s, 9H). ^{13}C NMR (101 MHz, DMSO-d_6) δ 159.21, 155.63, 154.40, 150.21, 135.55, 135.14, 131.60, 127.42, 127.20, 126.63, 126.55, 125.76, 123.68, 123.34, 122.92, 122.43, 120.85, 119.63, 111.45, 111.11, 109.88, 109.78, 86.72, 59.21, 57.50, 33.80, 30.57. HRMS (ESI) calculated for $C_{32}H_{29}O_4^+$ [M+H]$^+$: 477.2060; found: 477.2050.

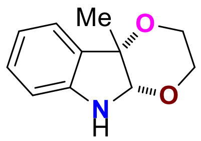

9b-Methyl-3,4a,5,9b-tetrahydro-2H-[1,4]dioxino[2,3-b]indole (6a): white solid was obtained in 95% isolated yield. ^{1}H NMR (400 MHz, CDCl$_3$) δ 7.26 – 7.21 (m, 1H), 7.16 (td, *J* = 7.6, 1.2 Hz, 1H), 6.87 (td, *J* = 7.2, 1.2 Hz, 1H), 6.70 (dt, *J* = 7.6, 0.8 Hz, 1H), 4.71 - 4.64 (m, 1H), 4.30 (s, 1H), 3.79 – 3.59 (m, 4H), 1.46 (s, 3H). ^{13}C NMR (101 MHz, CDCl$_3$) δ 148.12, 129.97, 129.14, 122.98, 119.96, 110.61, 90.65, 79.23, 61.80, 61.17, 24.42. HRMS (ESI) calculated for $C_{11}H_{14}NO_2S^+$ [M+H]$^+$: 192.1019; found: 192.1012.

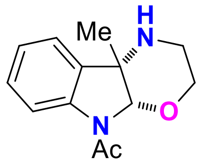

9b-Methyl-2,3,4,4a,5,9b-hexahydro-[1,4]oxazino[3,2-b]indole (6b): colorless oil was obtained in 82% isolated yield. ^{1}H NMR (400 MHz, DMSO-d_6) δ 7.95 (d, *J* = 7.6 Hz, 1H), 7.31 (dd, *J* = 7.2, 1.2 Hz, 1H), 7.21 (td, *J* = 7.6, 1.2 Hz, 1H), 7.08 (td, *J* = 7.6, 1.2 Hz, 1H), 5.15 (s, 1H), 3.56 – 3.43 (m, 2H), 2.94 (s, 1H), 2.62 (dt, *J* = 13.2, 2.8 Hz, 1H), 2.49 – 2.40 (m, 1H), 2.27 (s, 3H), 1.17 (s, 3H). ^{13}C NMR (101 MHz, DMSO) δ 169.77, 141.20, 136.12, 127.97, 123.81, 122.51, 115.90, 89.85, 62.75, 59.22, 40.02, 27.81, 22.96. HRMS (ESI) calculated for $C_{13}H_{17}N_2O_2S^+$ [M+H]$^+$: 233.1285; found: 233.1280.

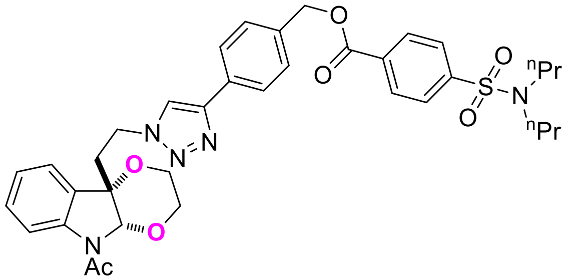

4-(1-(2-(5-Acetyl-3,4a,5,9b-tetrahydro-2H-[1,4]dioxino[2,3-b]indol-9b-yl)ethyl)-1H-1,2,3-triazol-4-yl)benzyl 4-(N,N-dipropylsulfamoyl)benzoate (6c): colorless liquid was obtained in 98% yield. ^{1}H NMR (400 MHz, DMSO-d_6) δ 8.65 (s, 1H), 8.25 – 8.14 (m, 2H), 8.06 (d, *J* = 8.0 Hz, 1H), 8.00 – 7.94 (m, 2H), 7.89 – 7.81 (m, 2H), 7.61 – 7.54 (m, 2H), 7.43 – 7.32 (m, 2H), 7.20 (td, *J* = 7.6, 1.2 Hz, 1H), 5.48 (s, 1H), 5.41 (s, 2H), 4.77 – 4.65 (m, 1H), 4.50 – 4.40 (m, 1H), 3.86 – 3.71 (m, 2H), 3.65 – 3.58 (m, 1H), 3.54 – 3.44 (m, 1H), 3.12 – 2.97 (m, 4H), 2.43 – 2.32 (m, 1H), 2.28 (s, 3H), 2.26 – 2.18 (m, 1H), 1.52 – 1.41 (m, 4H), 0.80 (t, *J* = 7.6 Hz, 6H). ^{13}C NMR (101 MHz,

DMSO) δ 170.09, 165.00, 146.45, 144.07, 141.89, 135.67, 133.39, 131.23, 130.74, 130.03, 129.34, 127.71, 125.66, 124.54, 124.14, 122.26, 116.89, 86.93, 79.12, 67.06, 61.73, 61.05, 50.02, 45.14, 39.01, 23.35, 22.02, 11.42. HRMS (ESI) calculated for $C_{36}H_{42}N_5O_7S^+$ $[M+H]^+$: 688.2799; found: 688.2791.

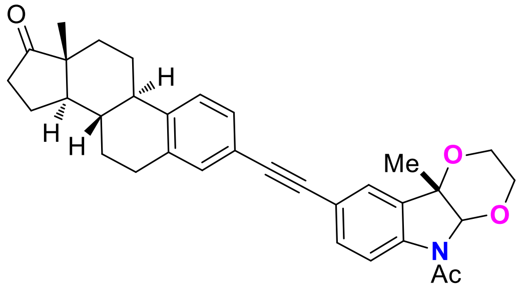

3-(((9bS)-5-Acetyl-9b-methyl-3,4a,5,9b-tetrahydro-2H-[1,4]dioxino[2,3-b]indol-8-yl)ethynyl)-13-methyl-7,8,9,11,12,13,15,16-octahydro-6H-cyclopenta[a]phenanthren-17(14H)-one (6d): white solid was obtained in 95% yield. ^{1}H NMR (400 MHz, $CDCl_3$) δ 8.11 (d, *J* = 8.0 Hz, 1H), 7.51 – 7.42 (m, 1H), 7.32 – 7.21 (m, 1H), 5.11 (s, 1H), 3.81 – 3.72 (m, 1H), 3.70 – 3.57 (m, 3H), 2.93 – 2.85 (m, 2H), 2.54 – 2.38 (m, 2H), 2.36 (s, 3H), 2.34 – 2.26 (m, 1H), 2.20 – 1.93 (m, 4H), 1.68 – 1.45 (m, 6H), 1.43 (s, 3H), 0.90 (s, 3H). ^{13}C NMR (101 MHz, $CDCl_3$) δ 220.82, 169.94, 140.88, 140.16, 136.58, 133.21, 132.38, 131.93, 128.78, 125.79, 125.38, 120.45, 119.28, 117.00, 89.27, 89.11, 88.57, 77.70, 61.50, 60.91, 50.41, 47.90, 44.41, 37.88, 35.80, 31.48, 29.07, 26.93, 26.29, 25.52, 21.53, 13.79, 0.98. HRMS (ESI) calculated for $C_{33}H_{36}NO_4^+$ $[M+H]^+$: 510.2639; found: 510.2629.

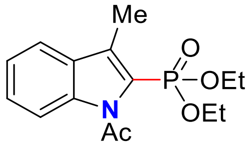

Diethyl (1-acetyl-3-methyl-1H-indol-2-yl)phosphonate (6e): colorless oil was obtained in 22% isolated yield. ^{1}H NMR (400 MHz, $CDCl_3$) δ 7.89 (d, *J* = 8.4 Hz, 1H), 7.64 (d, *J* = 7.9 Hz, 1H), 7.45 (t, *J* = 8.0 Hz, 1H), 7.31 (t, *J* = 7.6 Hz, 1H), 4.45 – 4.13 (m, 4H), 2.81 (s, 3H), 2.59 (d, *J* = 2.4 Hz, 3H), 1.51 – 1.31 (m, 6H). ^{13}C NMR (101 MHz, $CDCl_3$) δ 170.54, 137.60, 137.50, 133.26, 133.09, 130.31, 130.15, 127.76, 123.91, 123.16, 120.61, 114.53, 114.51, 62.84, 62.78, 27.29, 16.46, 16.39, 11.08. ^{31}P NMR (162 MHz, $CDCl_3$) δ 9.71.

Copies of product NMR Spectra

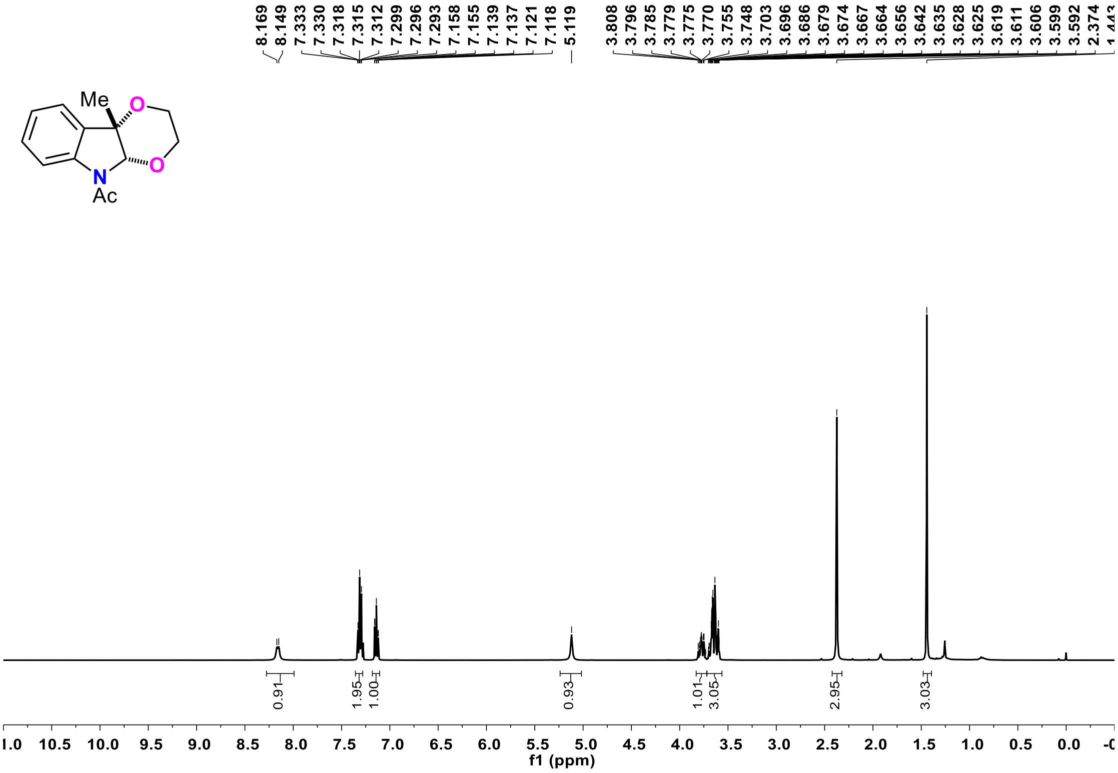

Supplementary Figure 8. ^{1}H NMR (400 MHz, $CDCl_3$) spectrum of 3aa

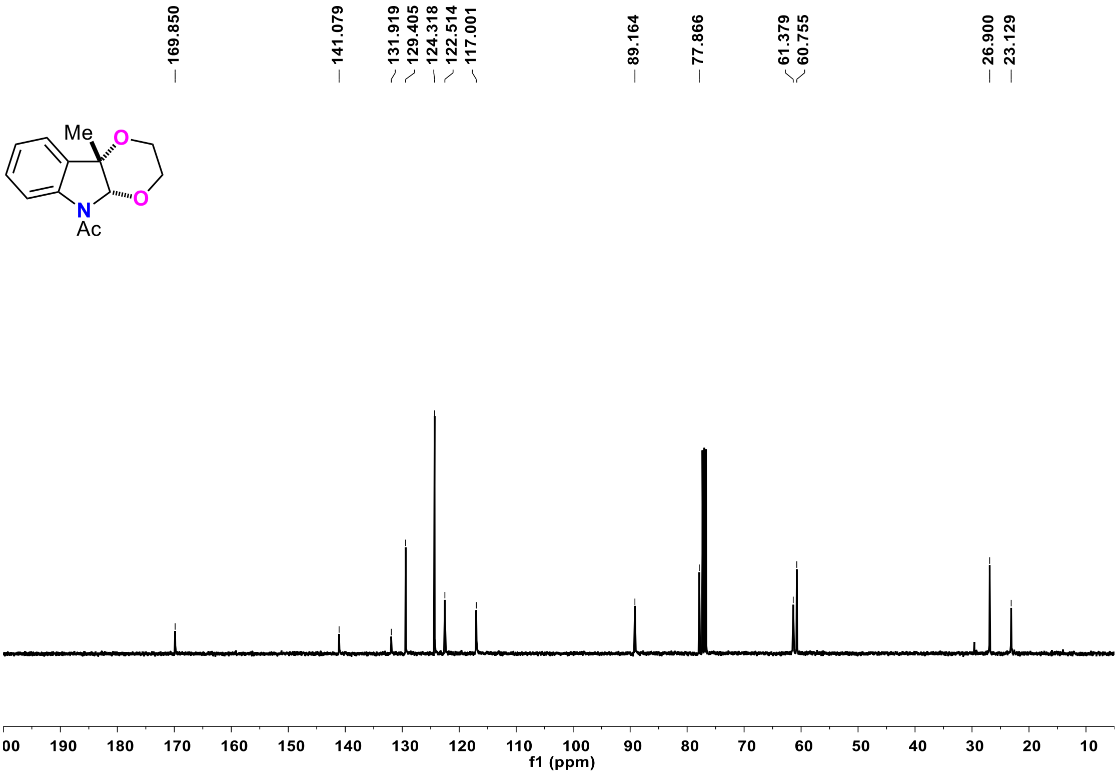

Supplementary Figure 9. ^{13}C NMR (101 MHz, $CDCl_3$) spectrum of 3aa

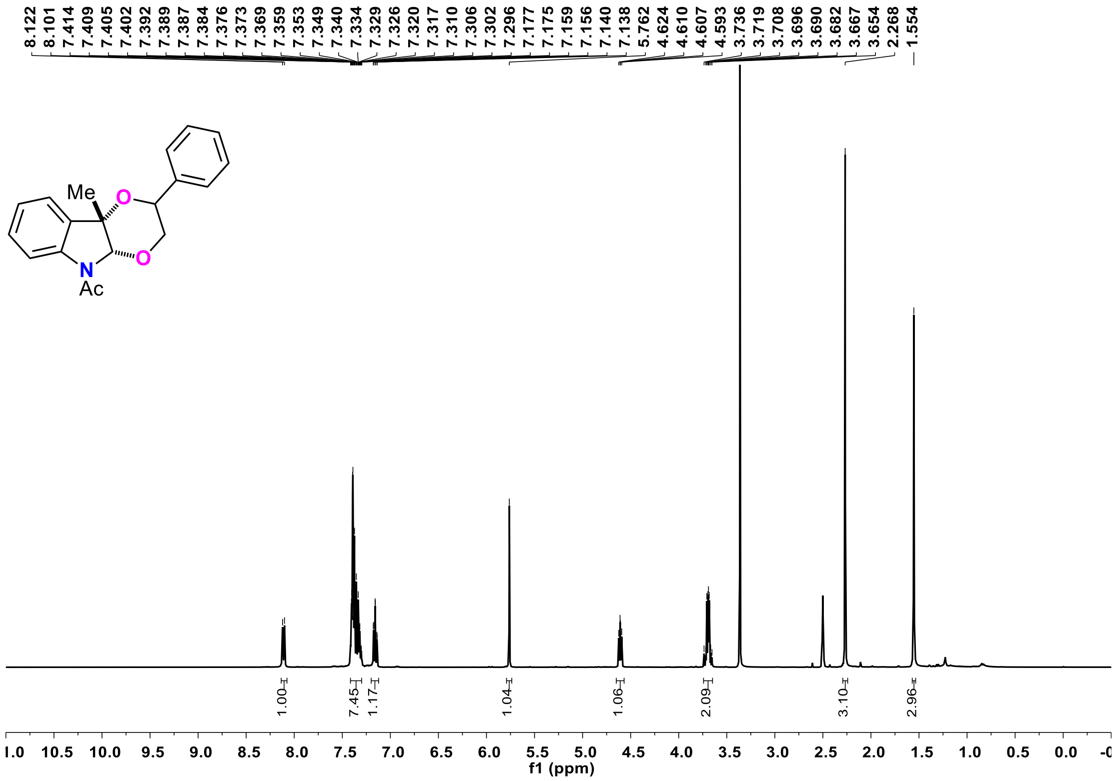

Supplementary Figure 10. ^{1}**H NMR (400 MHz, DMSO-d**$_{6}$**) spectrum of 3ab**

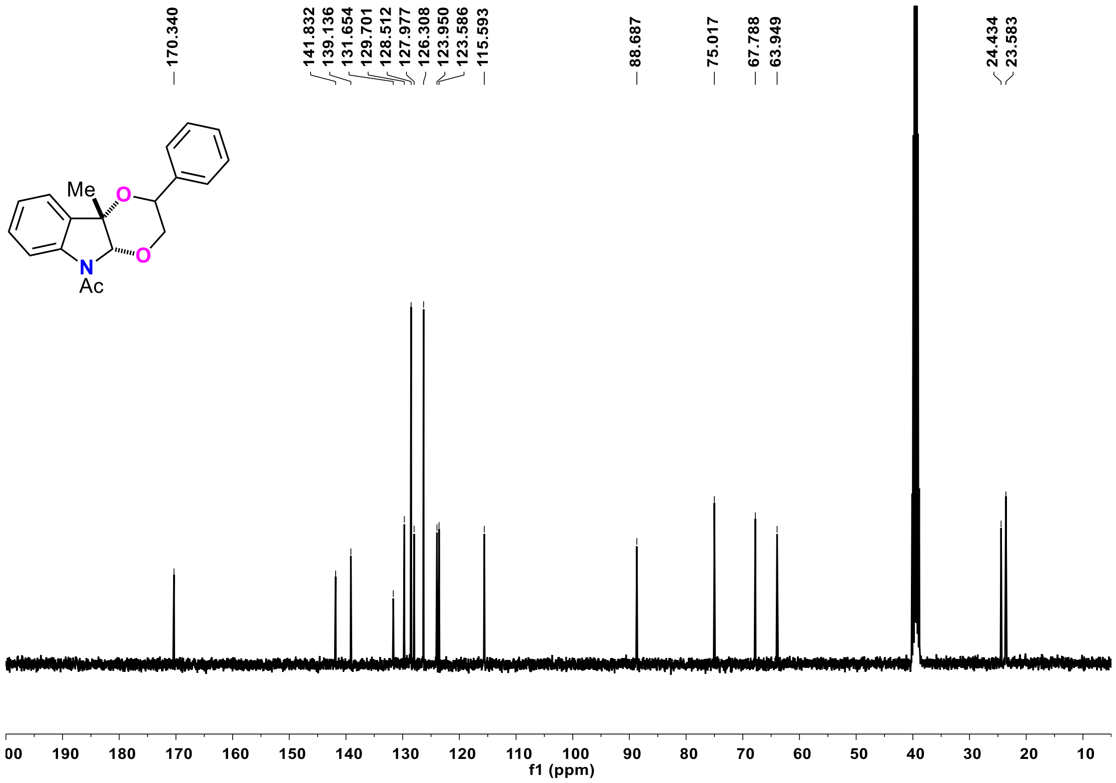

Supplementary Figure 11. ^{13}**C NMR (101 MHz, DMSO-d**$_{6}$**) spectrum of 3ab**

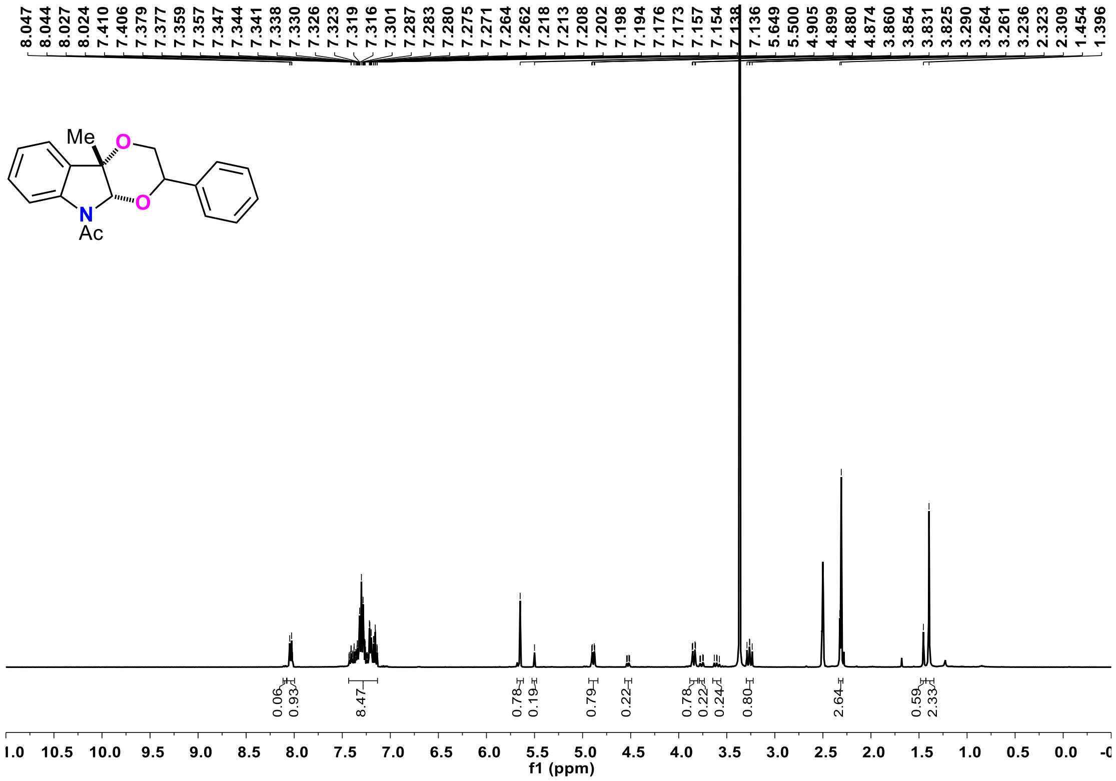

Supplementary Figure 12. ^{1}H **NMR (400 MHz, DMSO-d$_6$) spectrum of 3ab′**

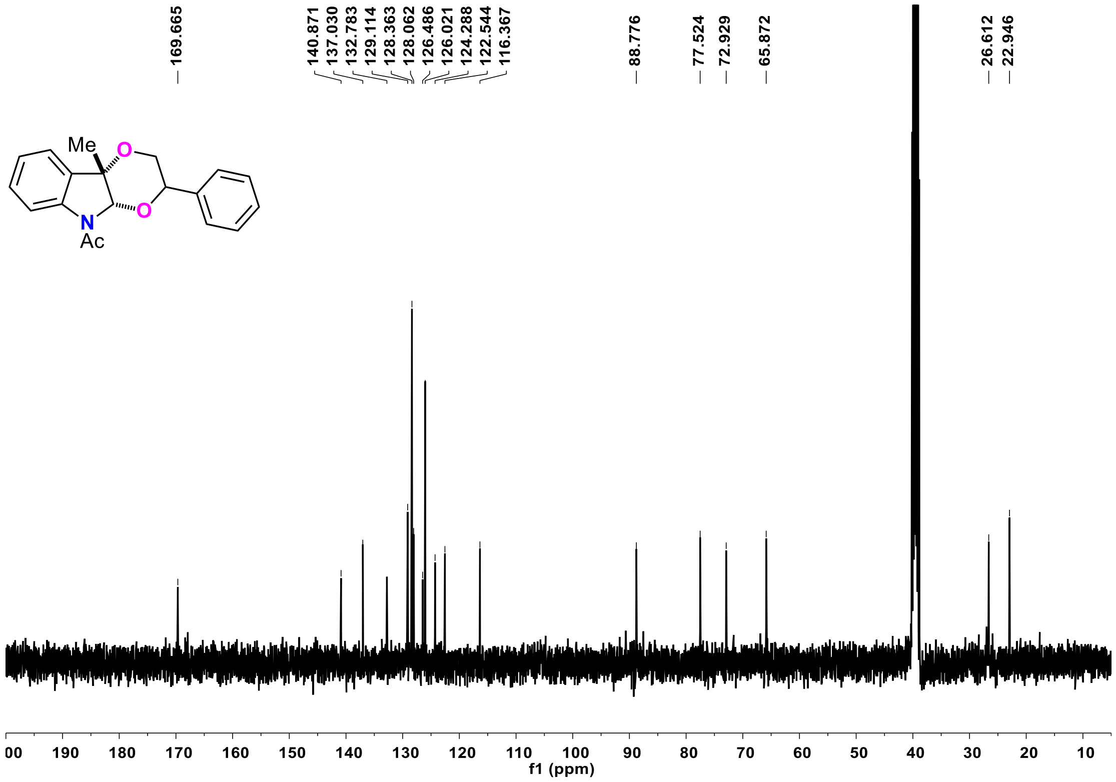

Supplementary Figure 13. ^{13}C **NMR (101 MHz, DMSO-d$_6$) spectrum of 3ab′**

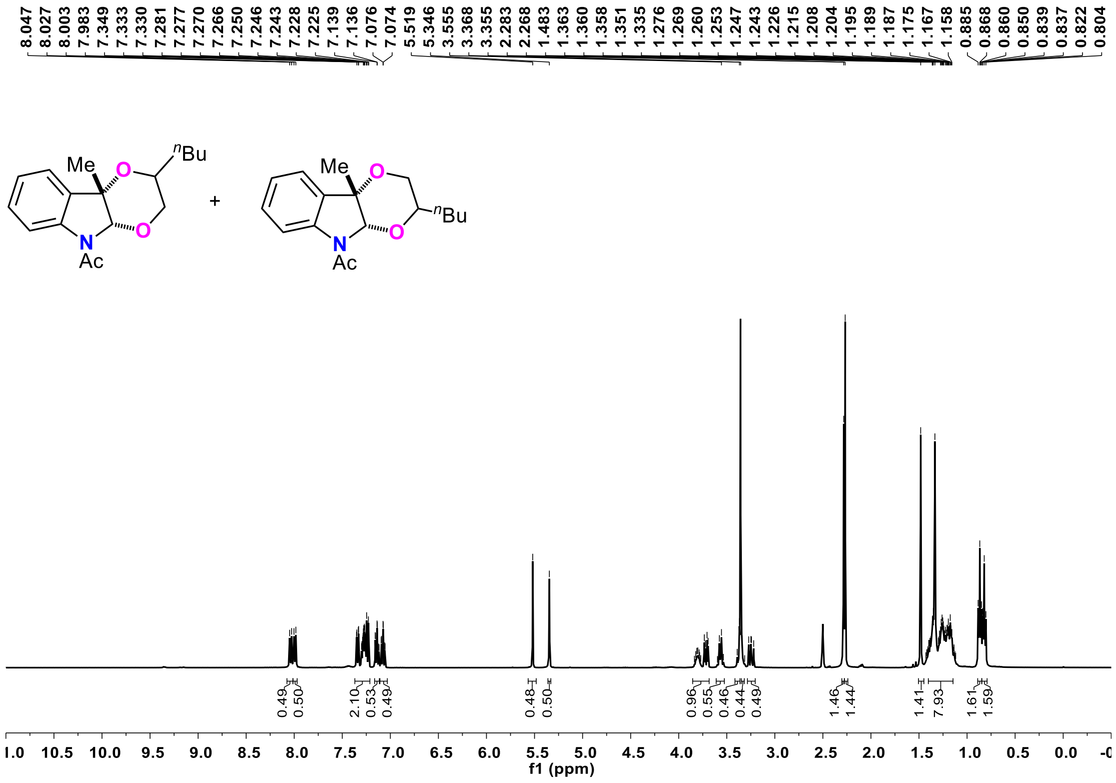

Supplementary Figure 14. ^{1}H NMR (400 MHz, DMSO-d$_6$) spectrum of 3ac

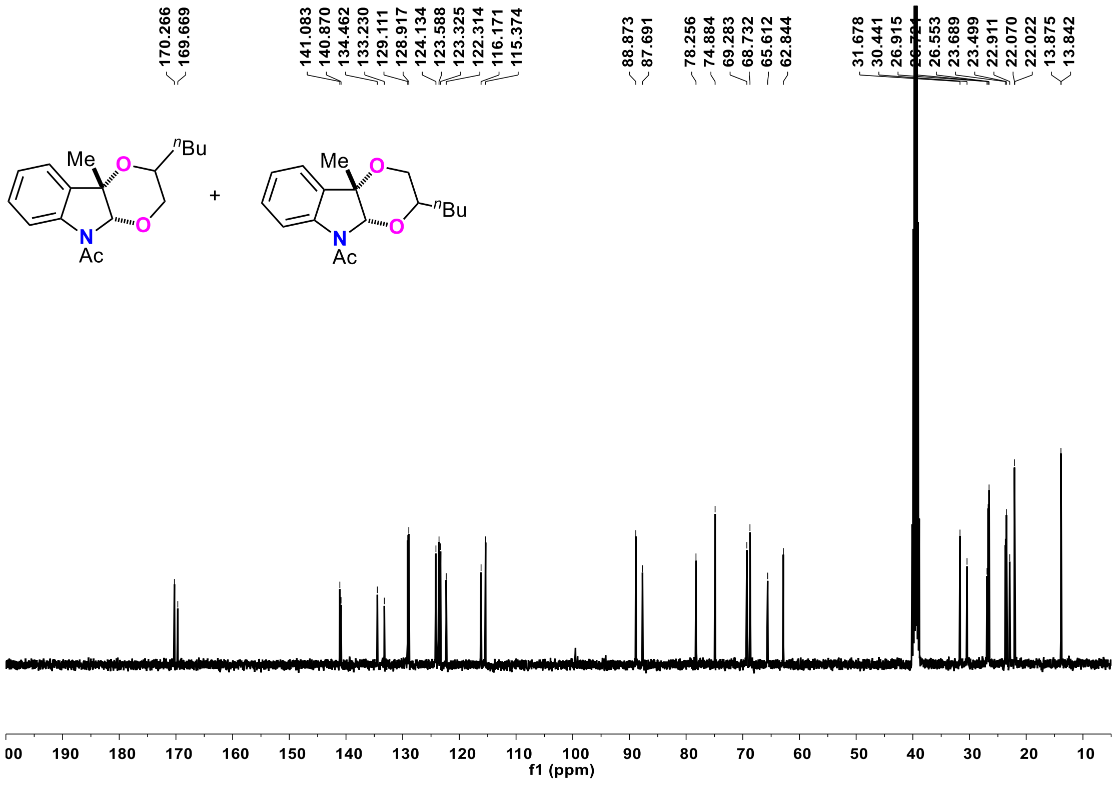

Supplementary Figure 15. ^{13}C NMR (101 MHz, DMSO-d$_6$) spectrum of 3ac

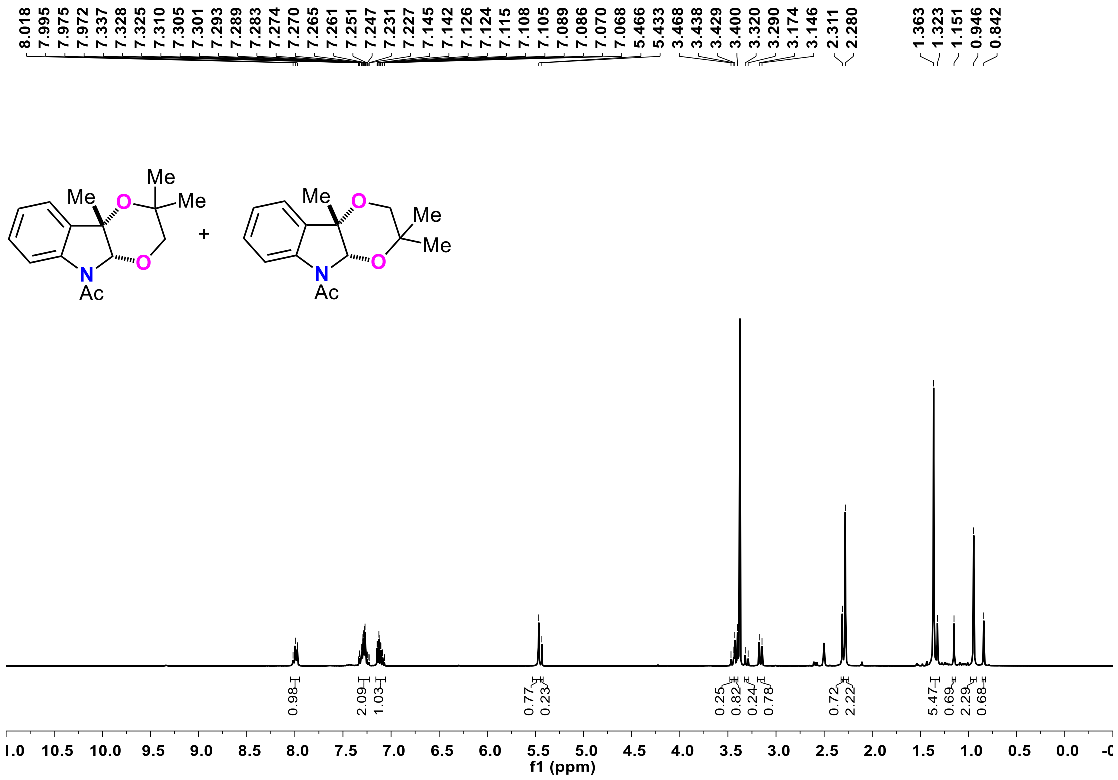

Supplementary Figure 16. ^{1}H NMR (400 MHz, DMSO-d_6) spectrum of 3ad

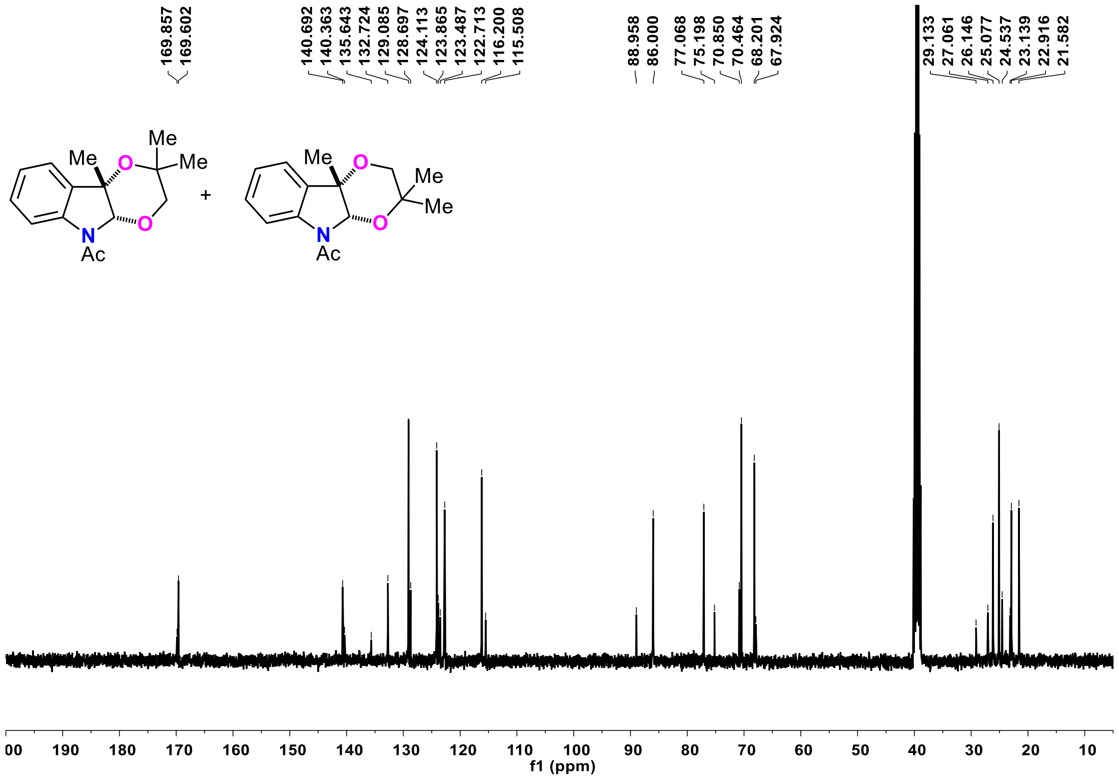

Supplementary Figure 17. ^{13}C NMR (101 MHz, DMSO-d_6) spectrum of 3ad

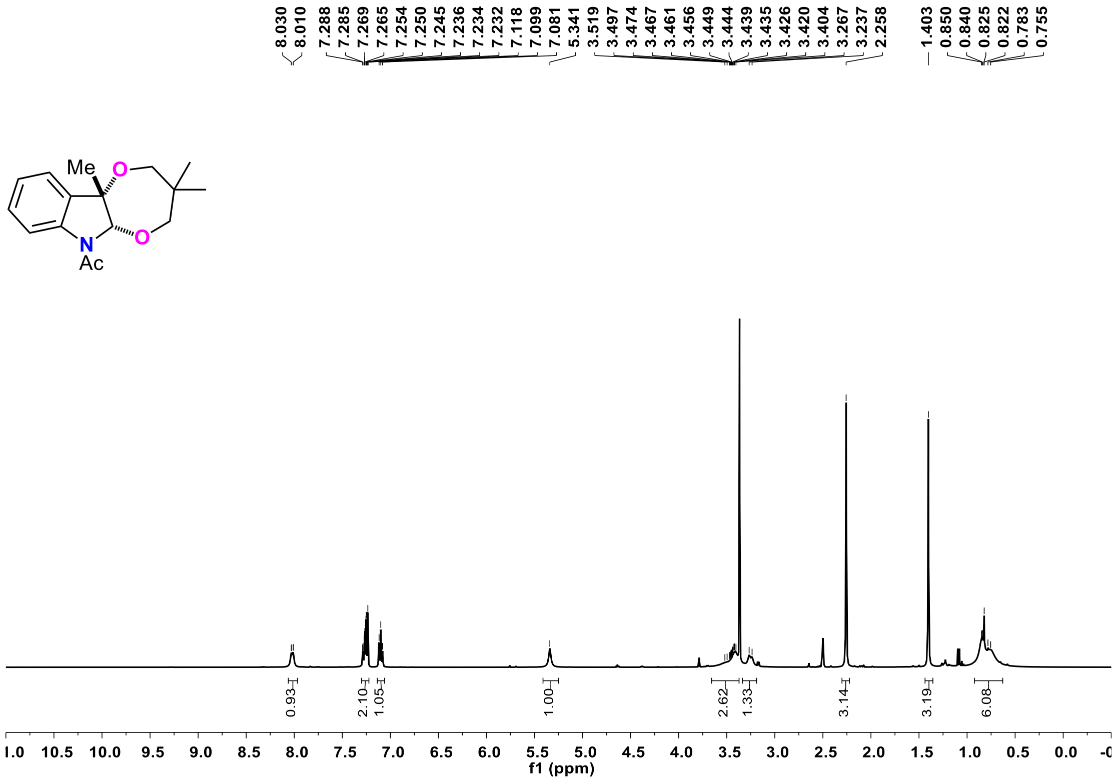

Supplementary Figure 18. ^{1}**H NMR (400 MHz, DMSO-d$_6$) spectrum of 3ae**

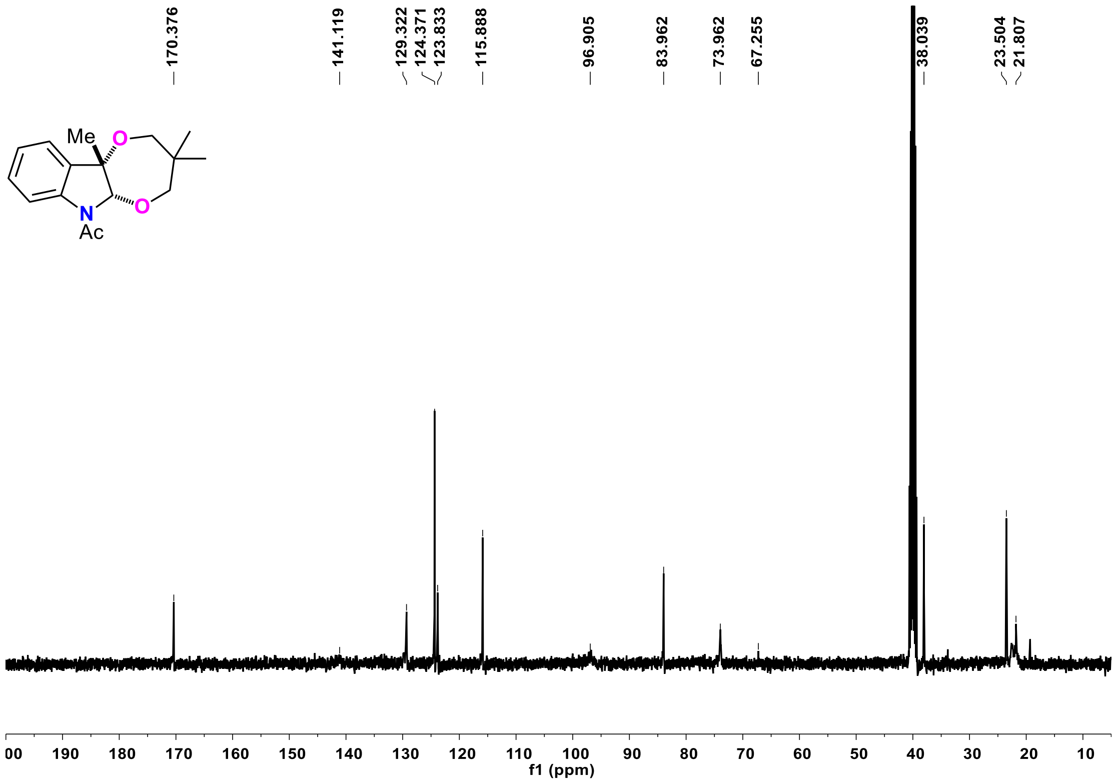

Supplementary Figure 19. ^{13}**C NMR (101 MHz, DMSO-d$_6$) spectrum of 3ae**

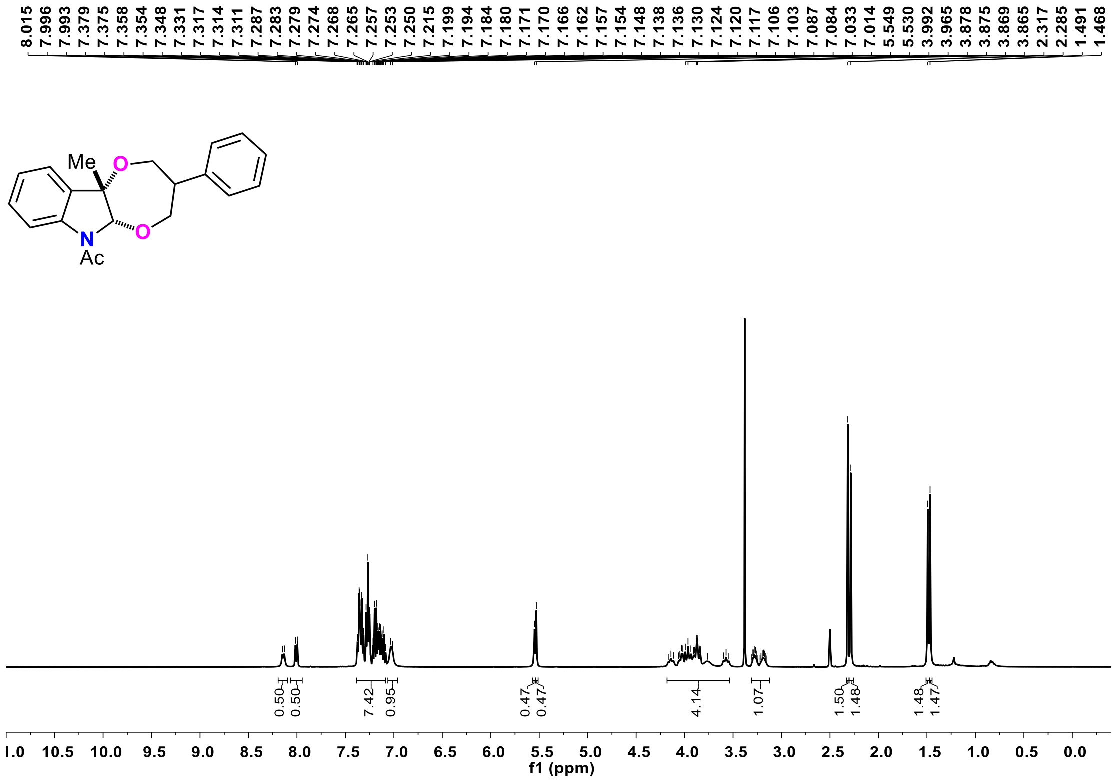

Supplementary Figure 20. ^{1}H NMR (400 MHz, DMSO-d_6) spectrum of 3af

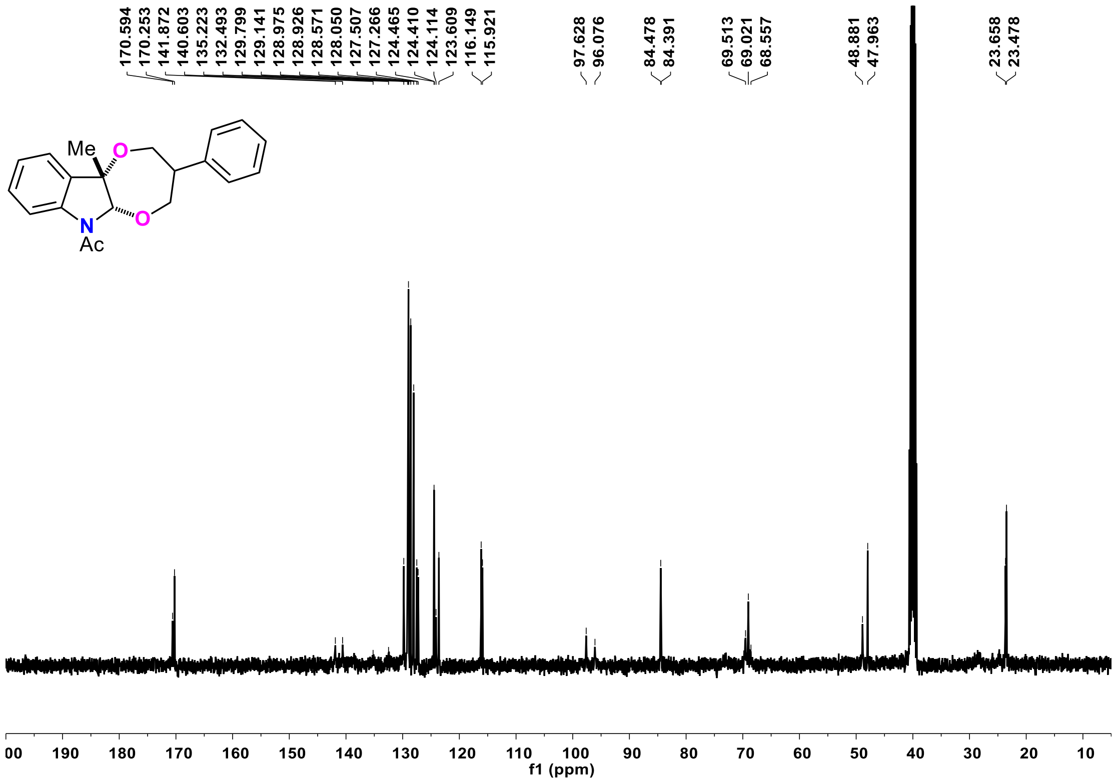

Supplementary Figure 21. ^{13}C NMR (101 MHz, DMSO-d_6) spectrum of 3af

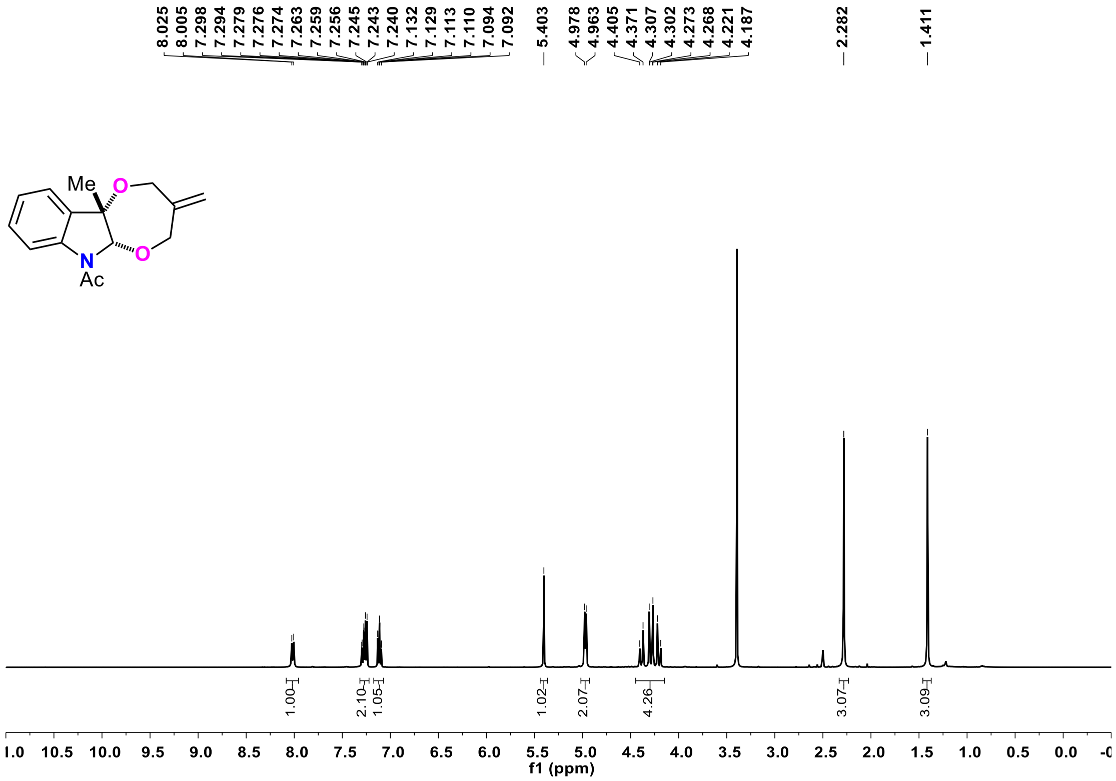

Supplementary Figure 22. ^{1}**H NMR (400 MHz, DMSO-d**$_{6}$**) spectrum of 3ag**

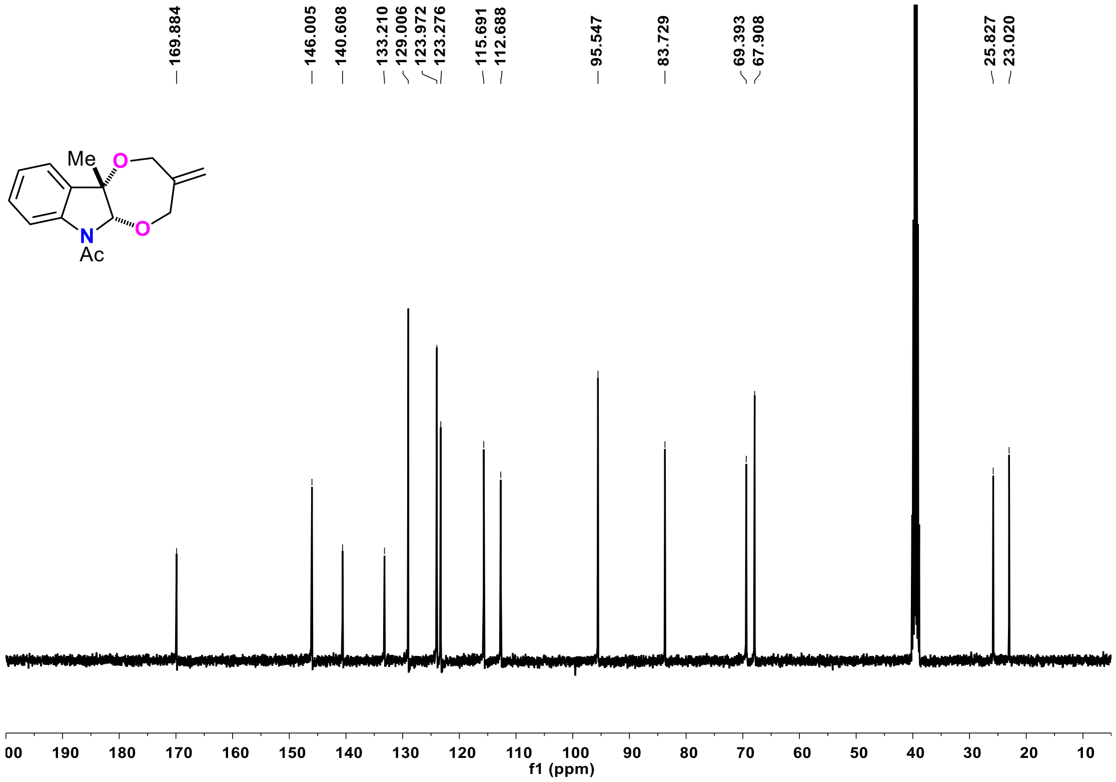

Supplementary Figure 23. ^{13}**C NMR (101 MHz, DMSO-d**$_{6}$**) spectrum of 3ag**

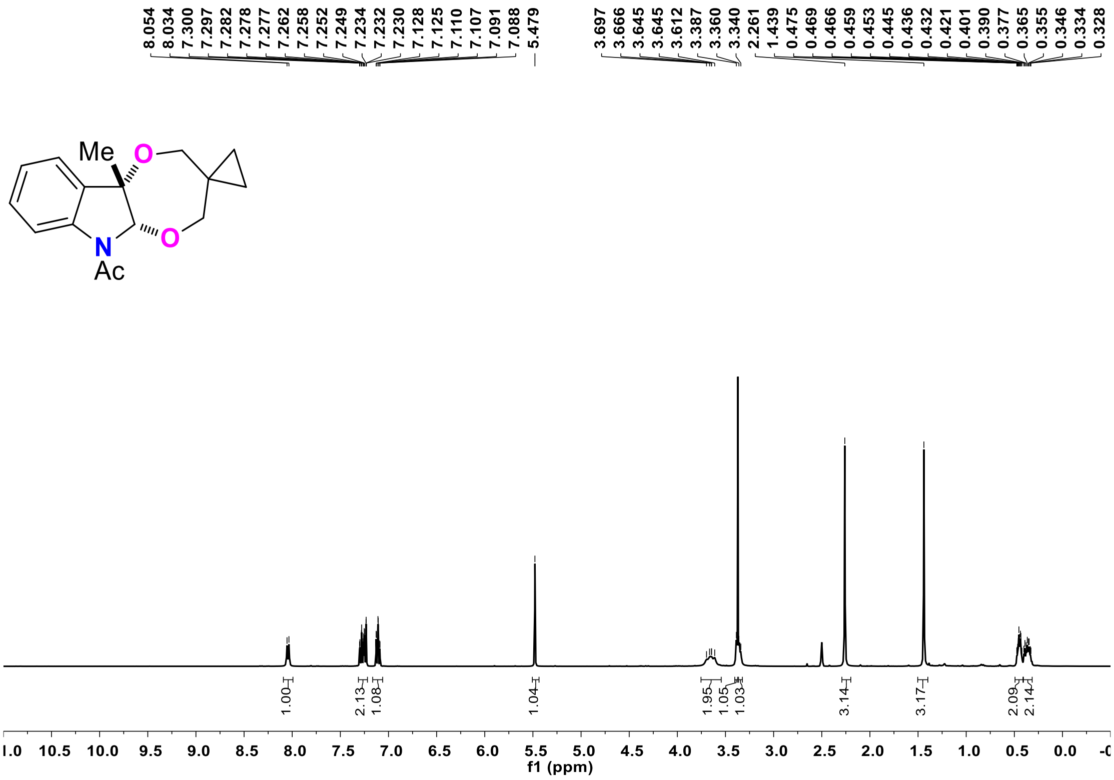

Supplementary Figure 24. ^{1}H NMR (400 MHz, DMSO-d_6) spectrum of 3ah

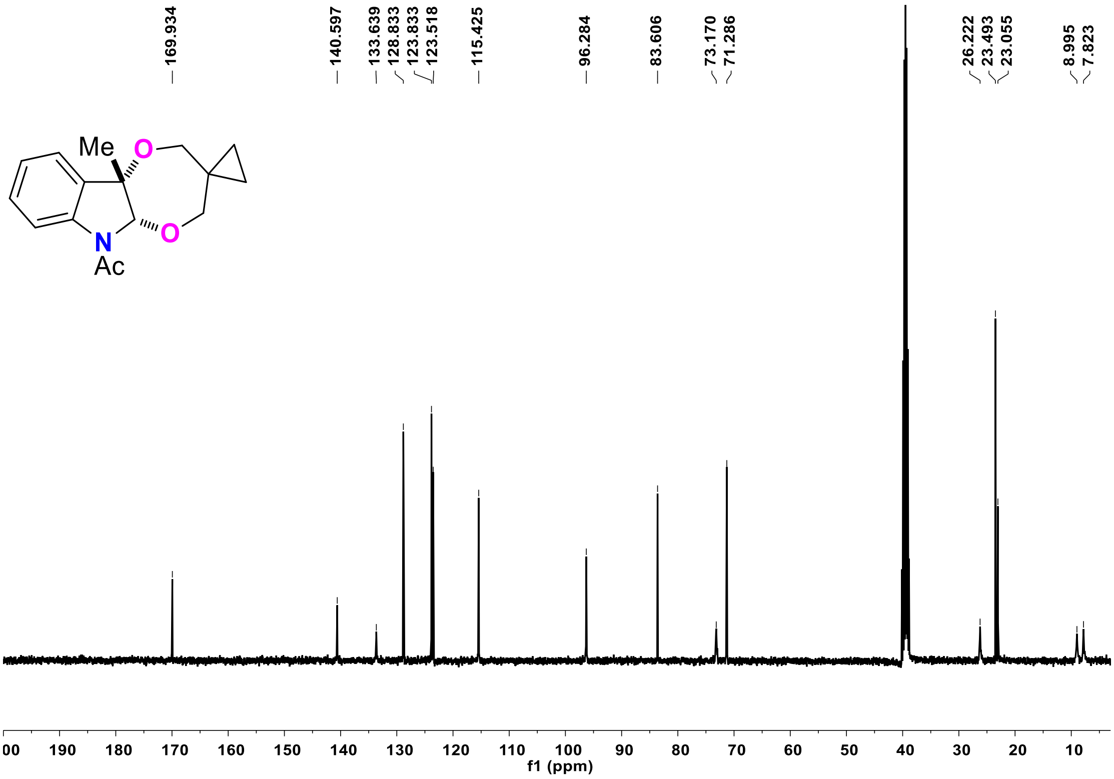

Supplementary Figure 25. ^{13}C NMR (101 MHz, DMSO-d_6) spectrum of 3ah

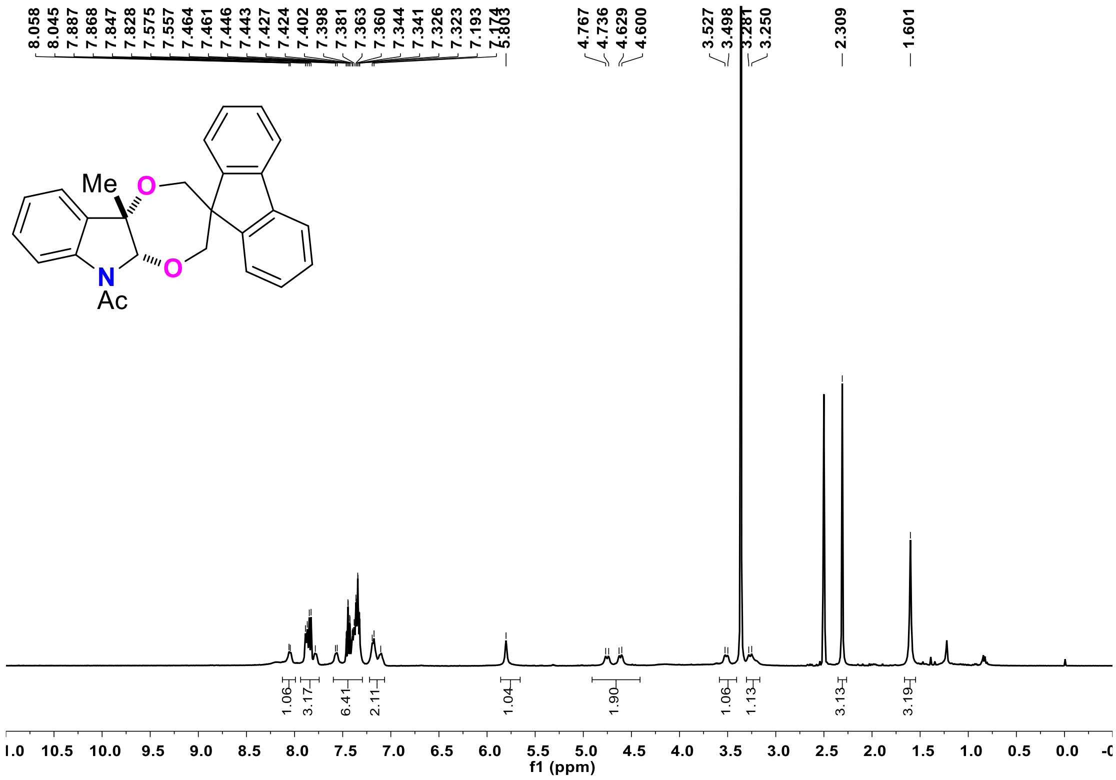

Supplementary Figure 26. ^{1}**H NMR (400 MHz, DMSO-d$_6$) spectrum of 3ai**

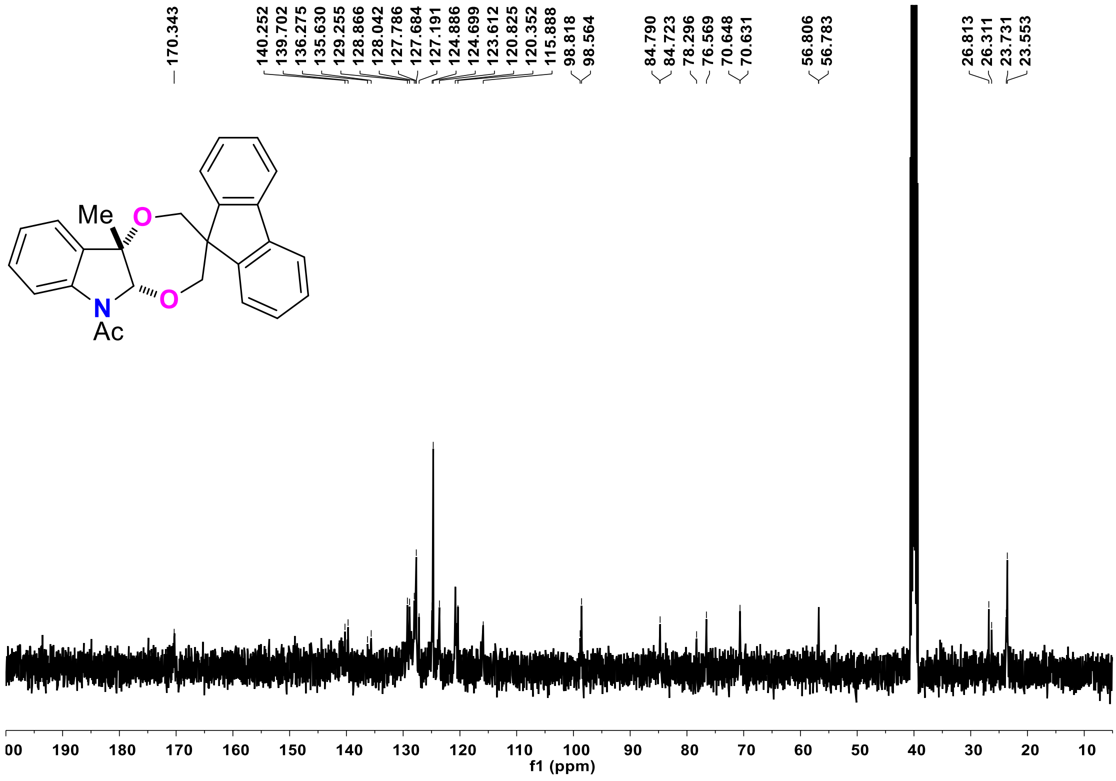

Supplementary Figure 27. ^{13}**C NMR (101 MHz, DMSO-d$_6$) spectrum of 3ai**

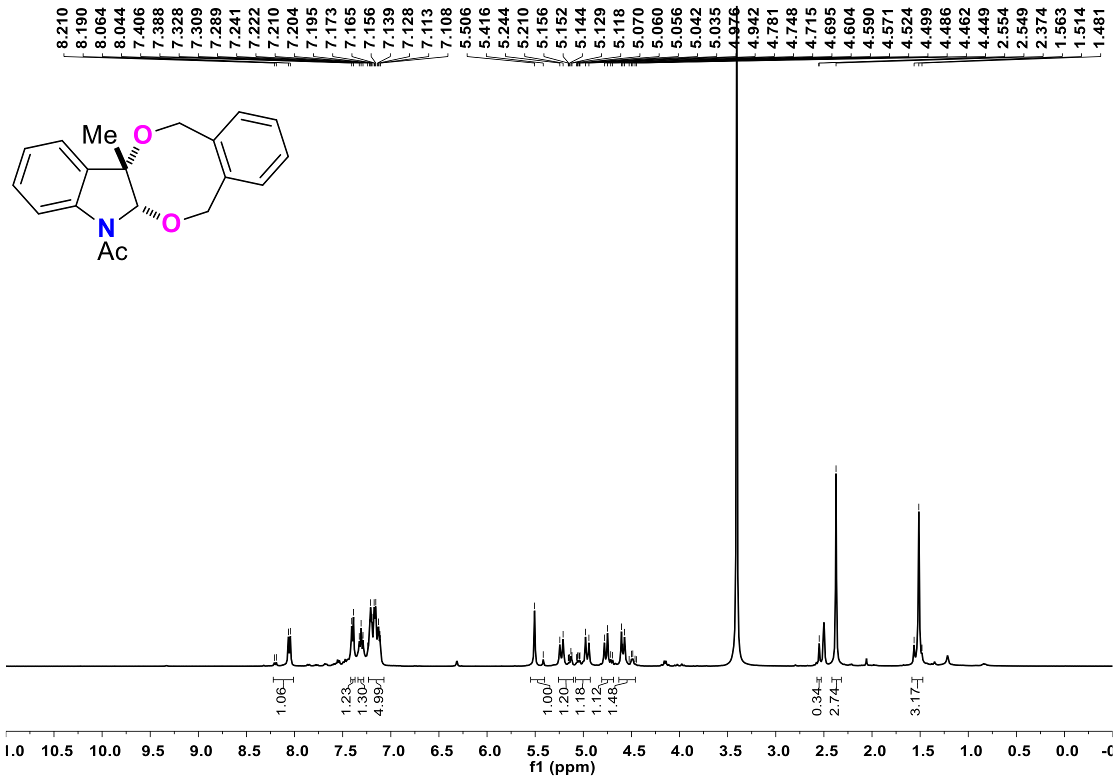

Supplementary Figure 28. ^{1}H NMR (400 MHz, DMSO-d$_6$) spectrum of 3aj

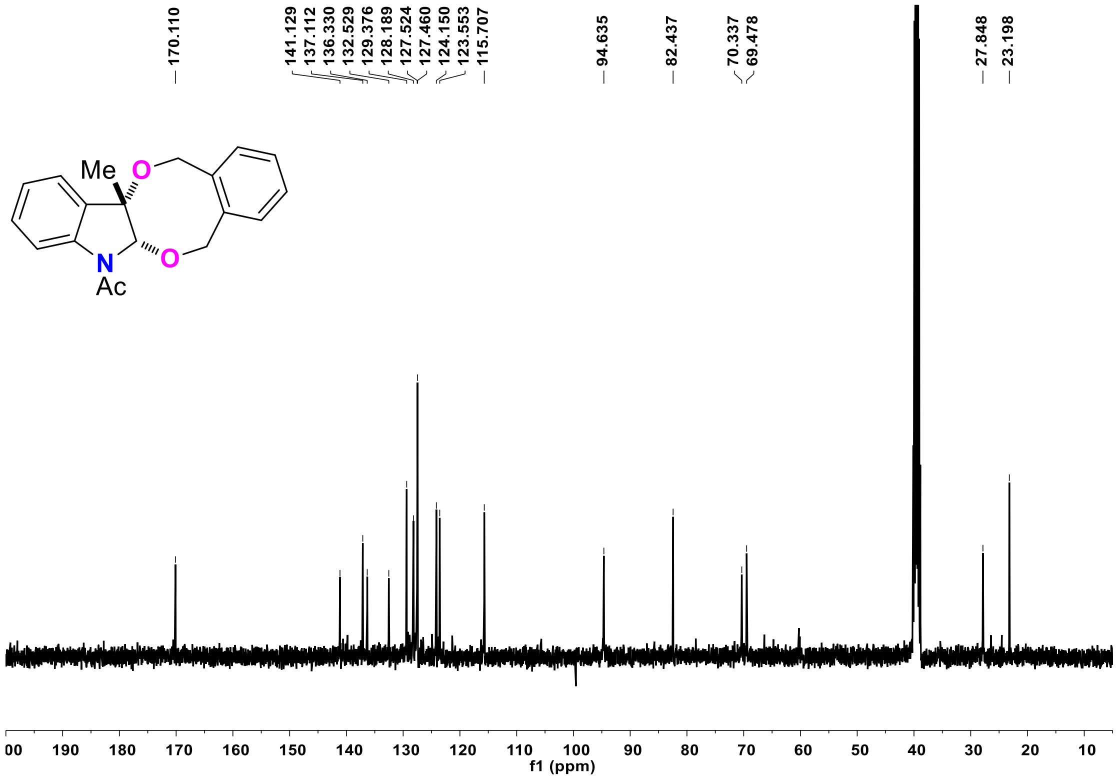

Supplementary Figure 29. ^{13}C NMR (101 MHz, DMSO-d$_6$) spectrum of 3aj

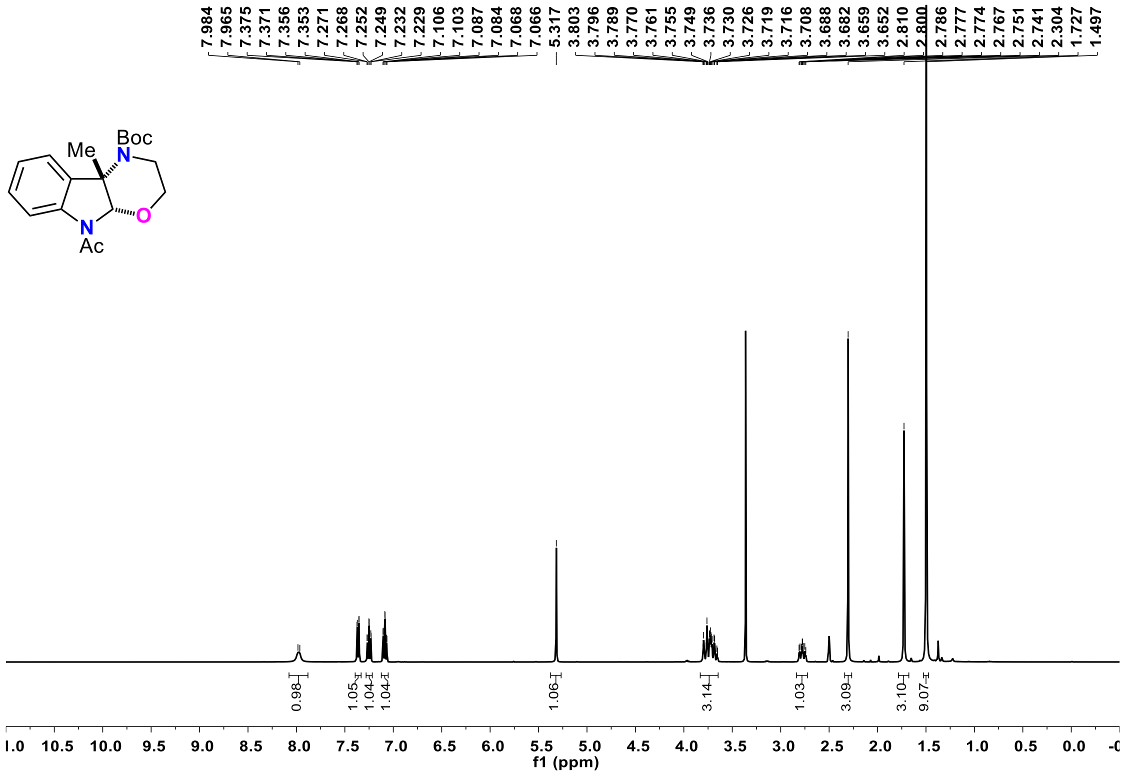

Supplementary Figure 30. ^{1}**H NMR (400 MHz, DMSO-d$_6$) spectrum of 3ak**

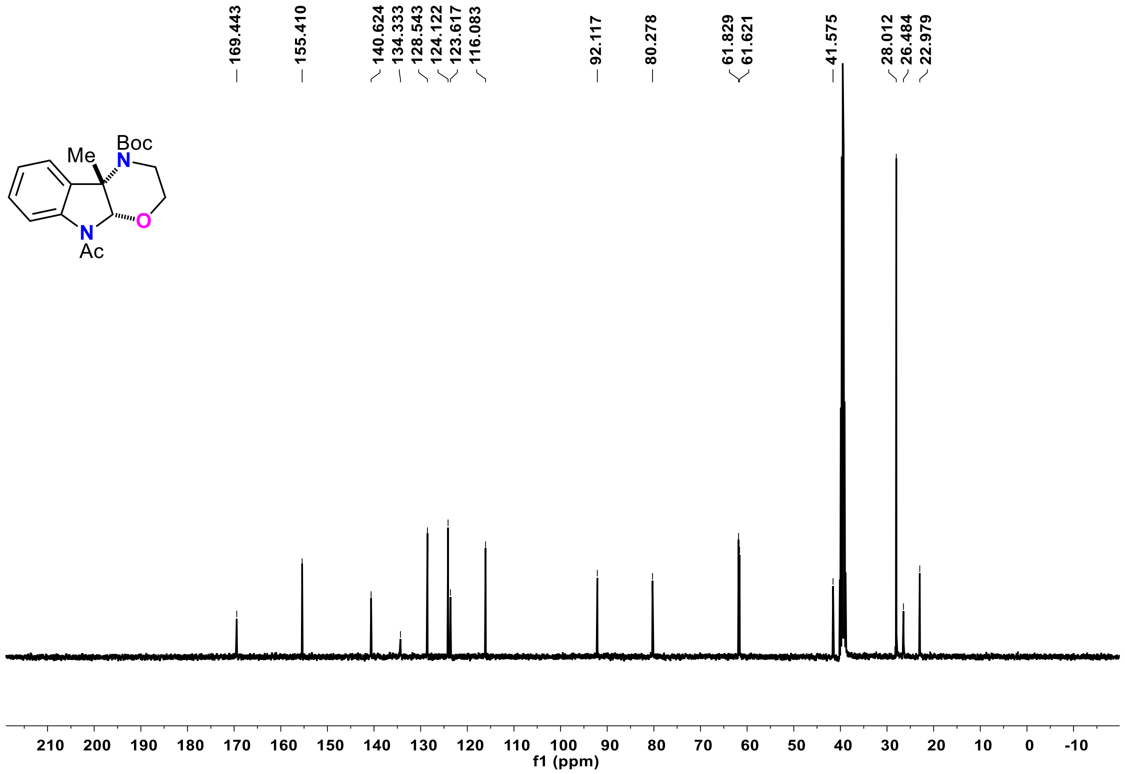

Supplementary Figure 31. ^{13}**C NMR (101 MHz, DMSO-d$_6$) spectrum of 3ak**

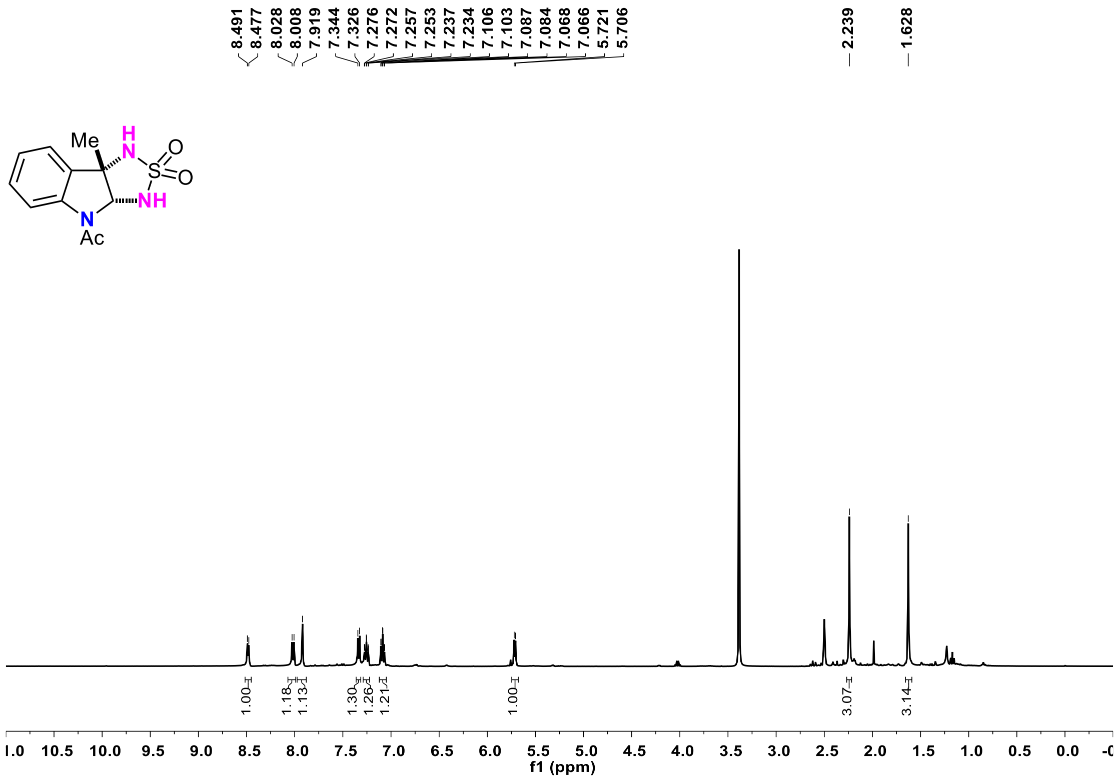

Supplementary Figure 32. ^{1}**H NMR (400 MHz, DMSO-d**$_{6}$**) spectrum of 3al**

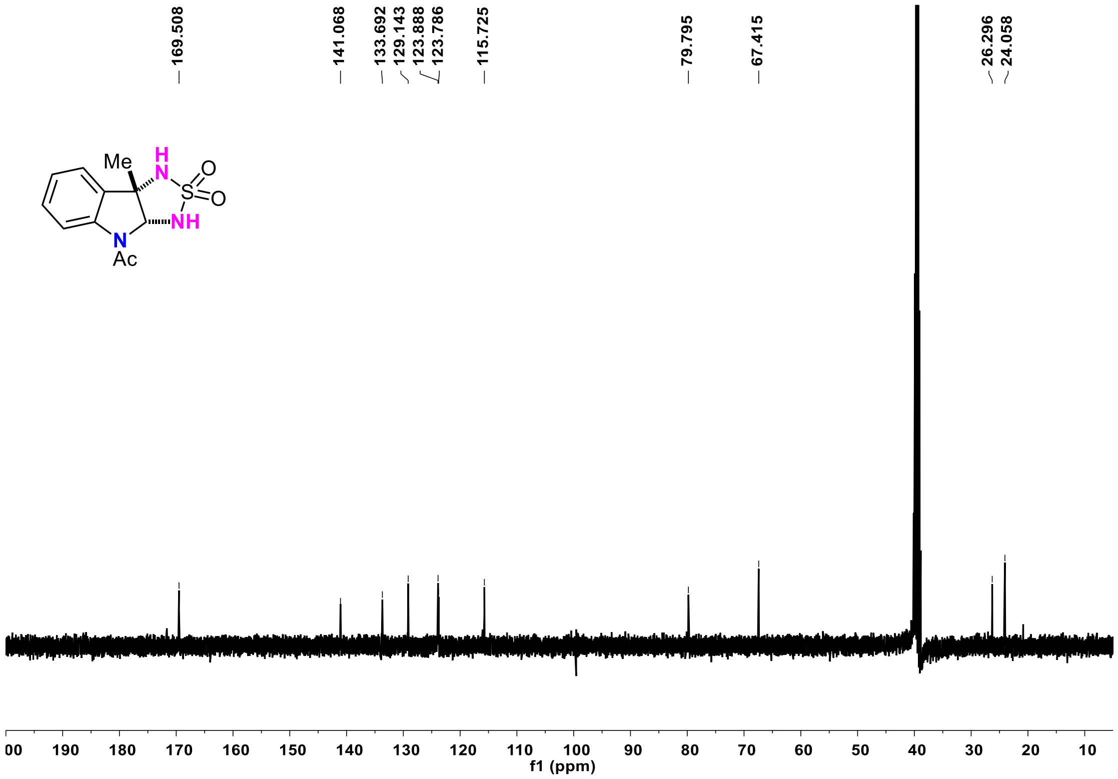

Supplementary Figure 33. ^{13}**C NMR (101 MHz, DMSO-d**$_{6}$**) spectrum of 3al**

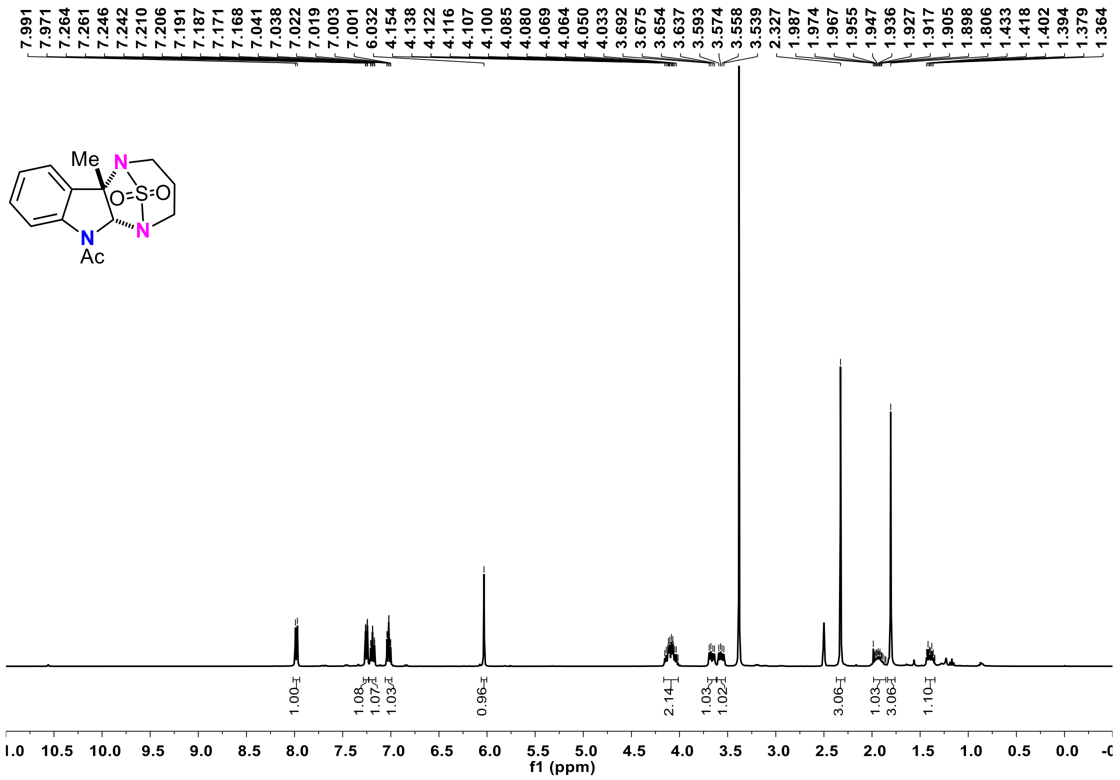

Supplementary Figure 34. ^{1}H NMR (400 MHz, DMSO-d_6) spectrum of 3am

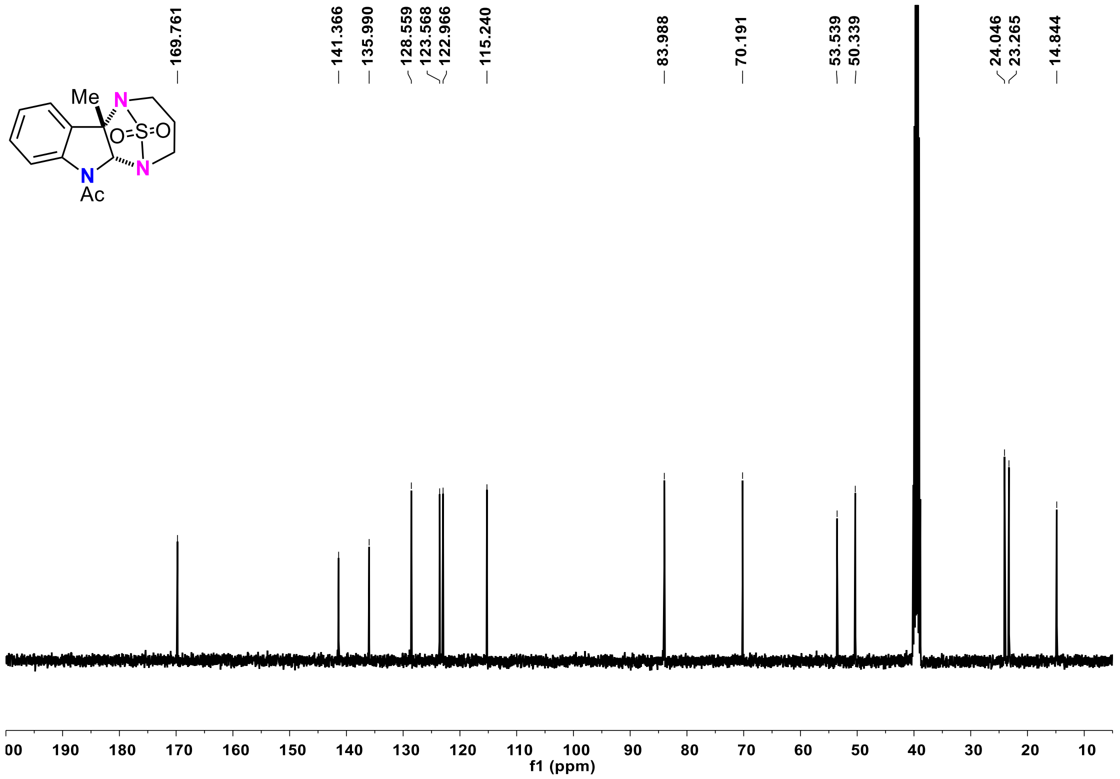

Supplementary Figure 35. ^{13}C NMR (101 MHz, DMSO-d_6) spectrum of 3am

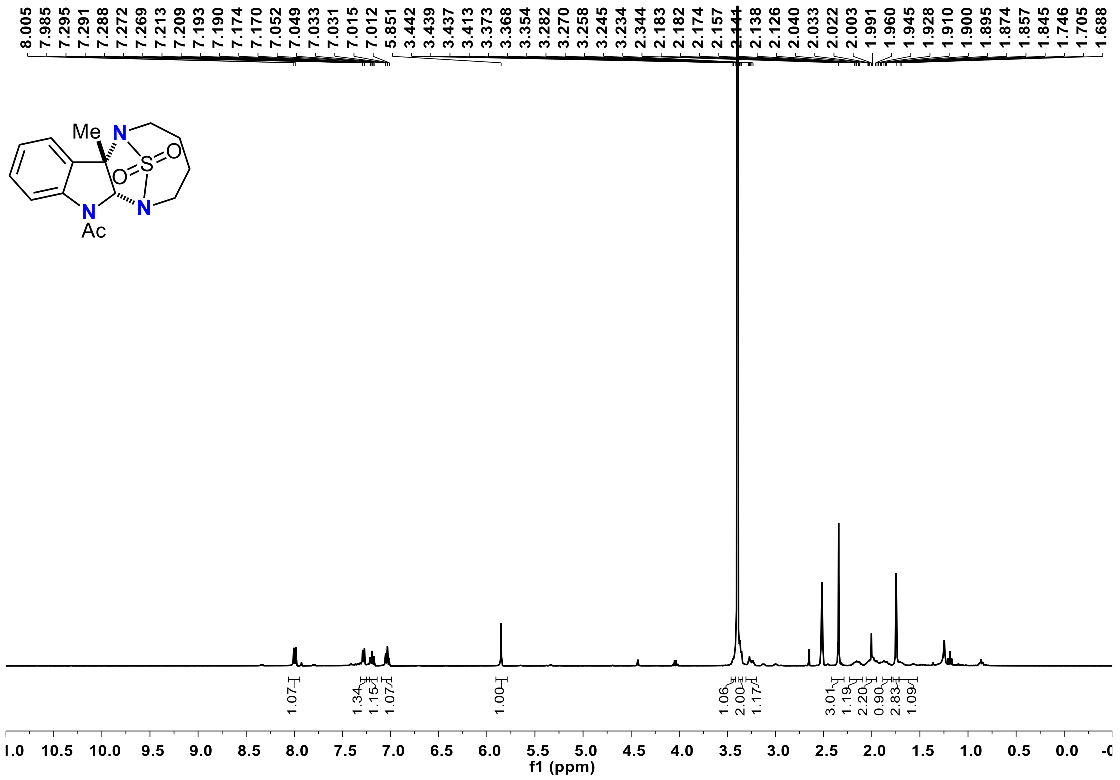

Supplementary Figure 36. ^{1}H NMR (400 MHz, DMSO-d$_6$) spectrum of 3an

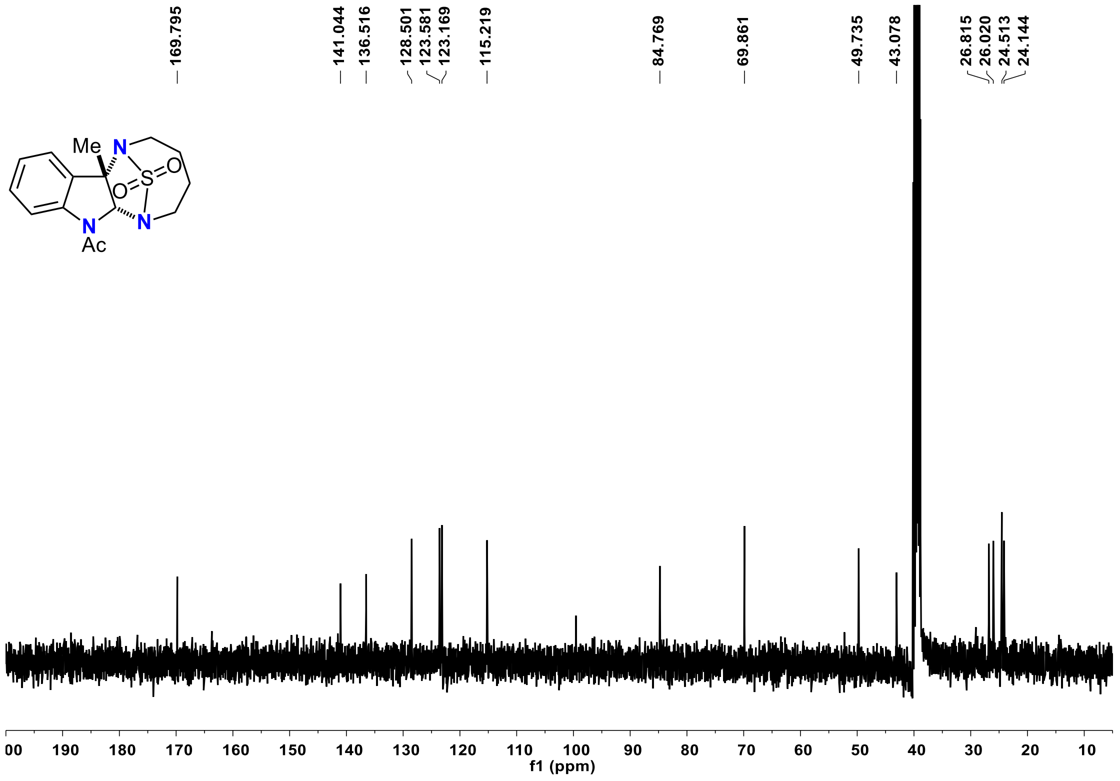

Supplementary Figure 37. ^{13}C NMR (101 MHz, DMSO-d$_6$) spectrum of 3an

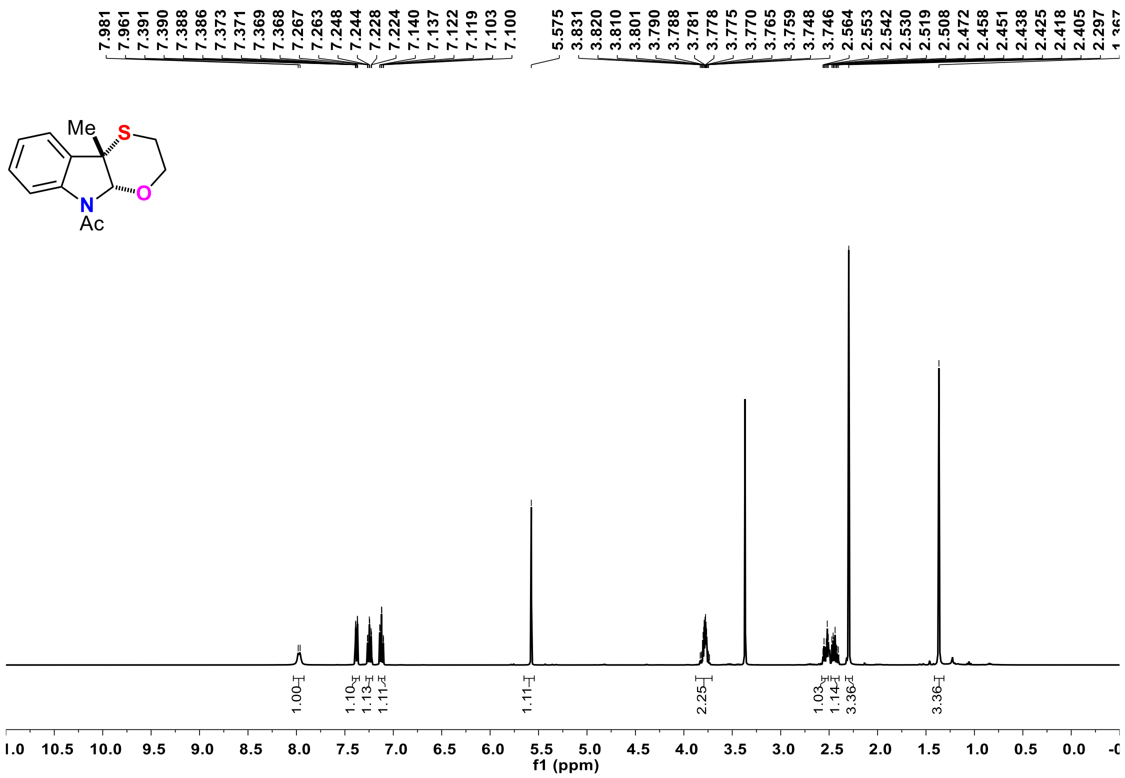

Supplementary Figure 38. ^{1}H NMR (400 MHz, DMSO-d_6) spectrum of 3ao

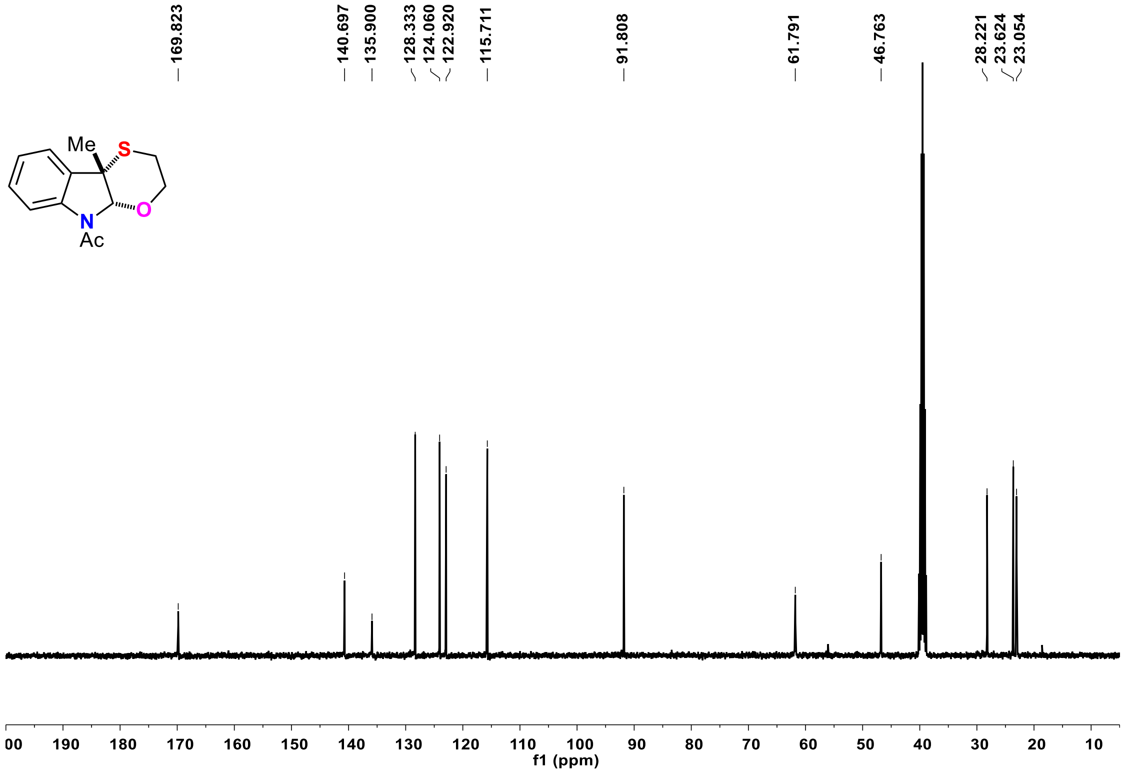

Supplementary Figure 39. ^{13}C NMR (101 MHz, DMSO-d_6) spectrum of 3ao

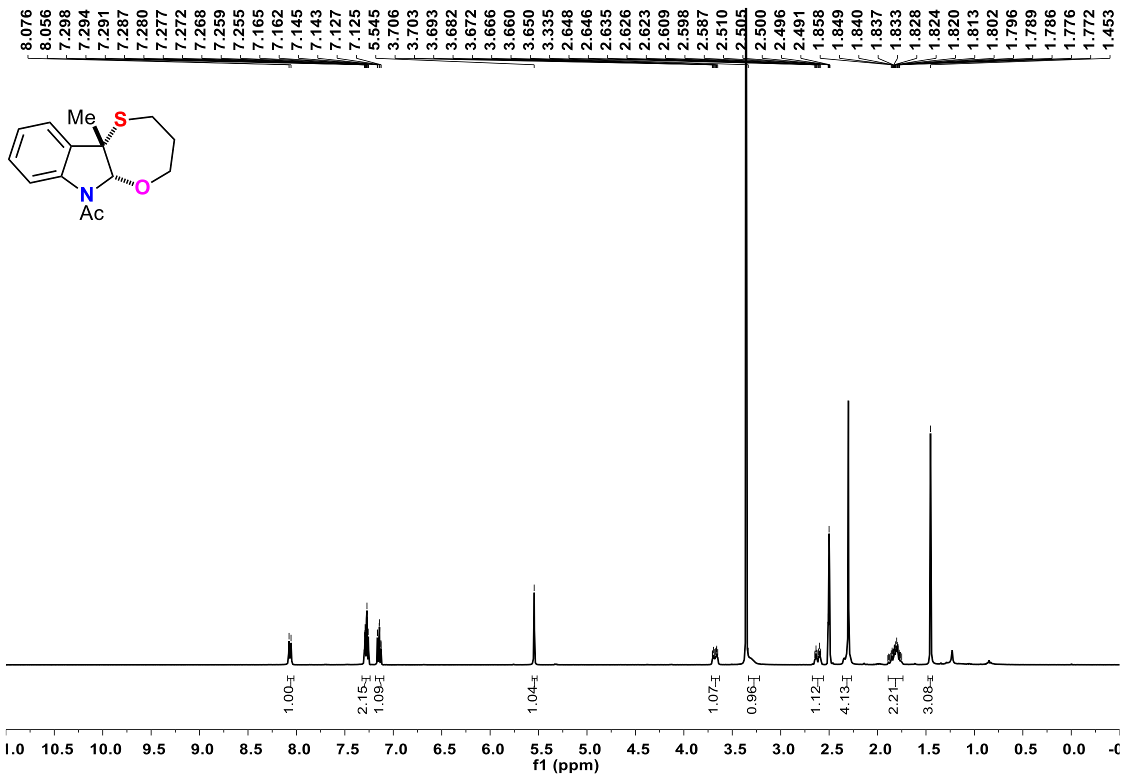

Supplementary Figure 40. **^{1}H NMR (400 MHz, DMSO-d_6) spectrum of 3ap**

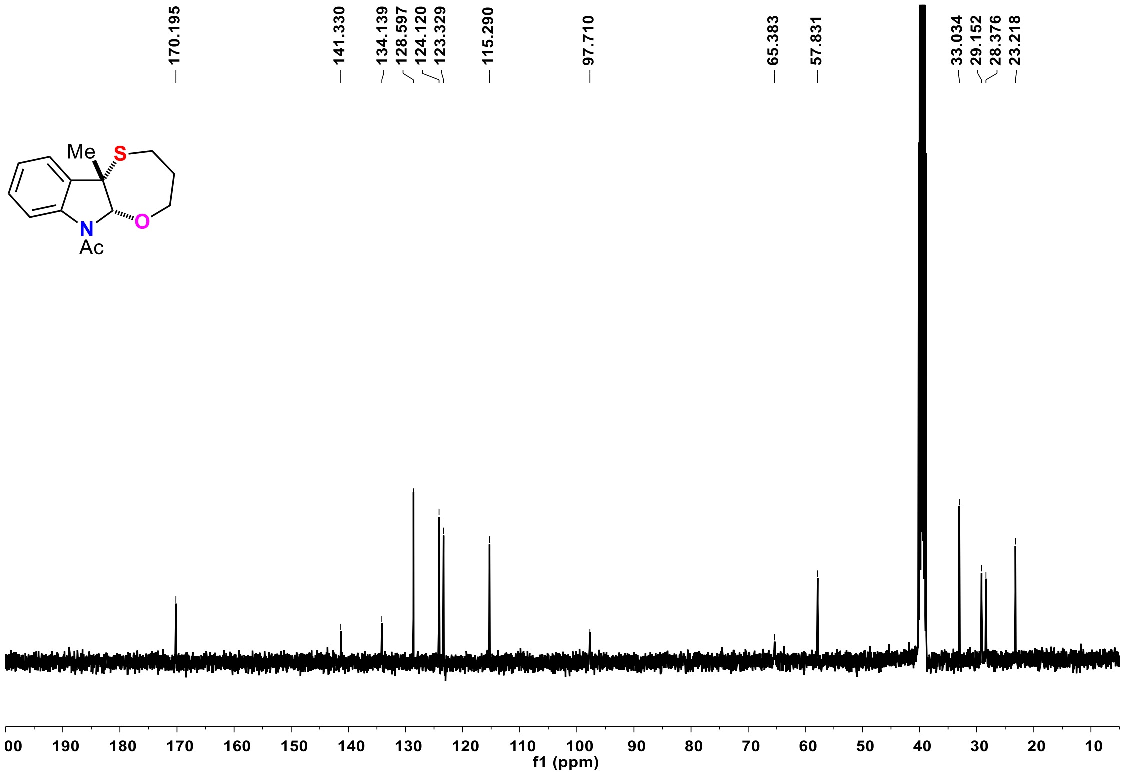

Supplementary Figure 41. **^{13}C NMR (101 MHz, DMSO-d_6) spectrum of 3ap**

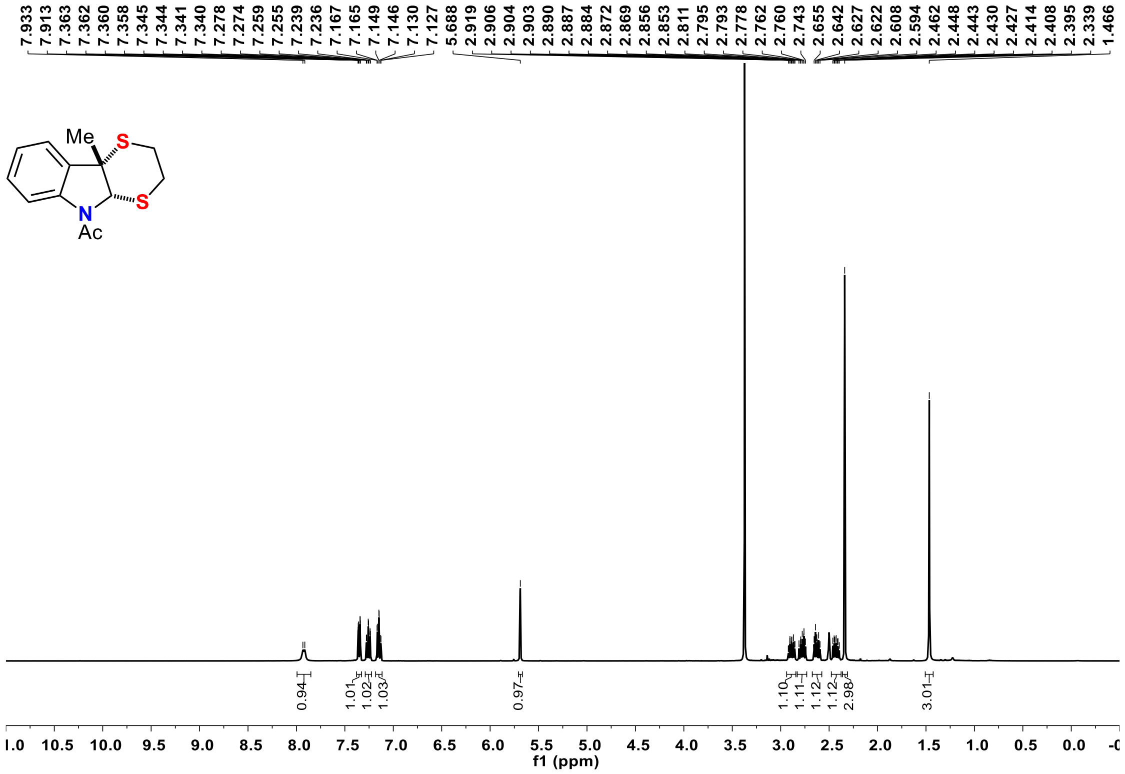

Supplementary Figure 42. ^{1}H **NMR (400 MHz, DMSO-d$_6$) spectrum of 3aq**

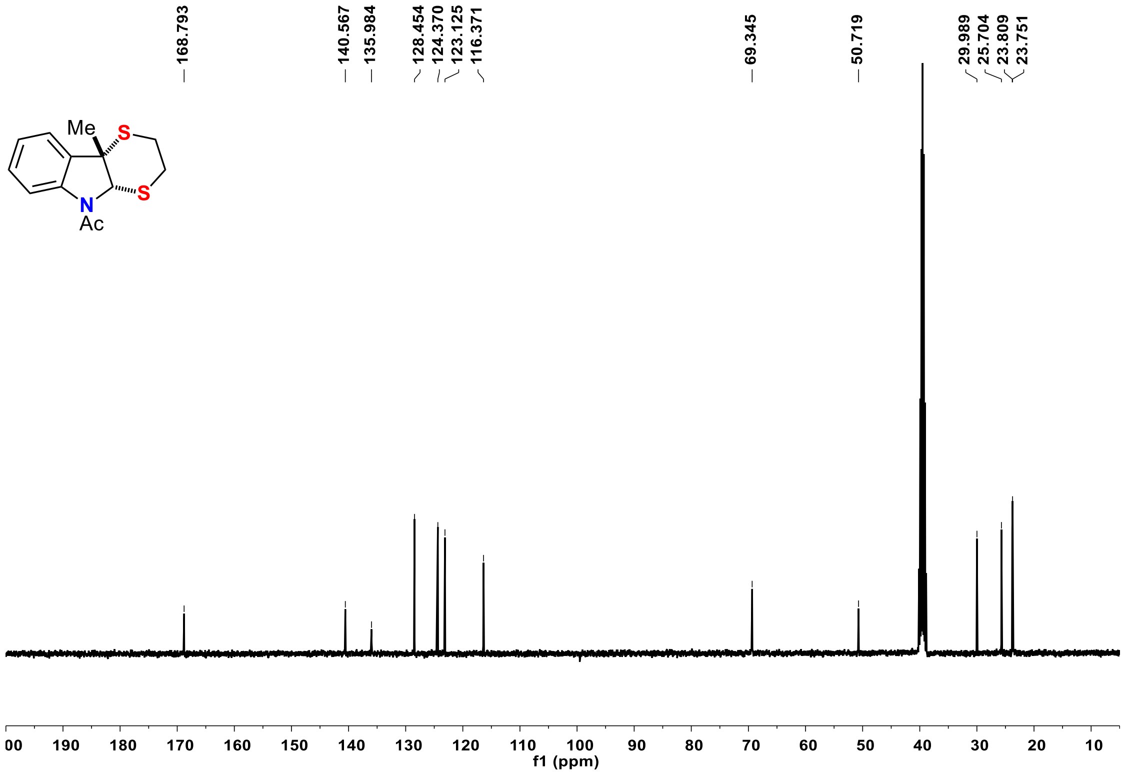

Supplementary Figure 43. ^{13}C **NMR (101 MHz, DMSO-d$_6$) spectrum of 3aq**

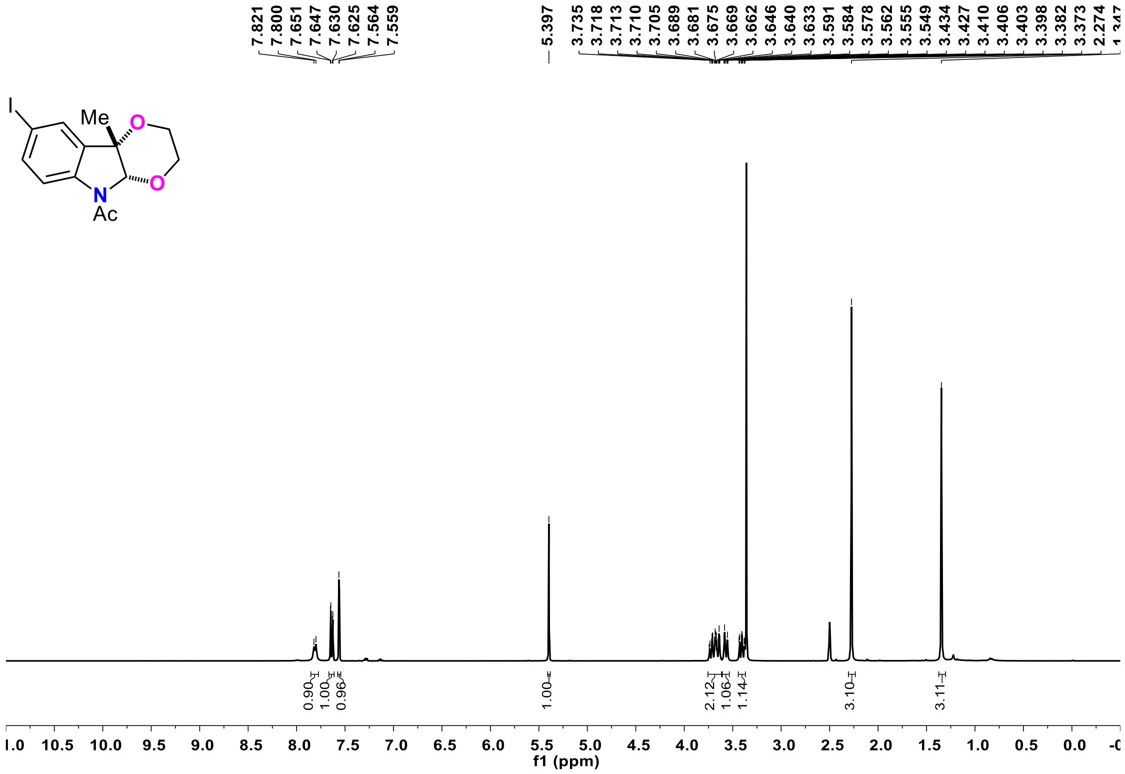

Supplementary Figure 44. ^{1}H NMR (400 MHz, DMSO-d$_6$) spectrum of 3ba

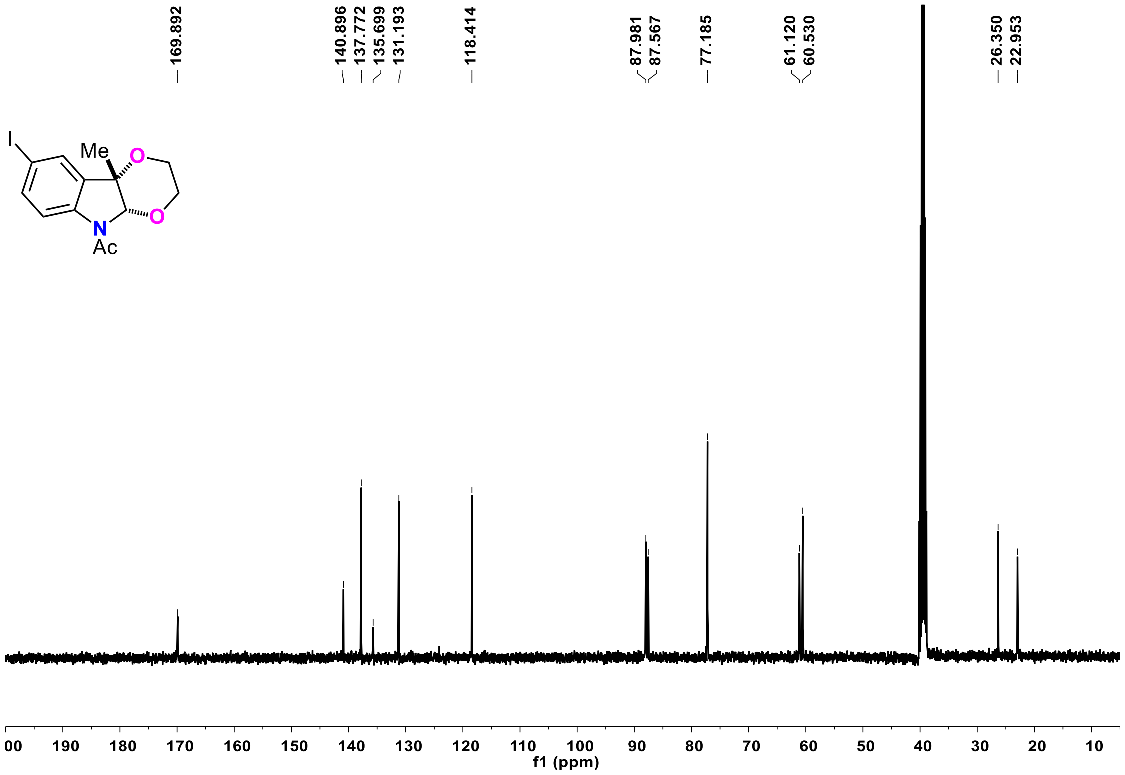

Supplementary Figure 45. ^{13}C NMR (101 MHz, DMSO-d$_6$) spectrum of 3ba

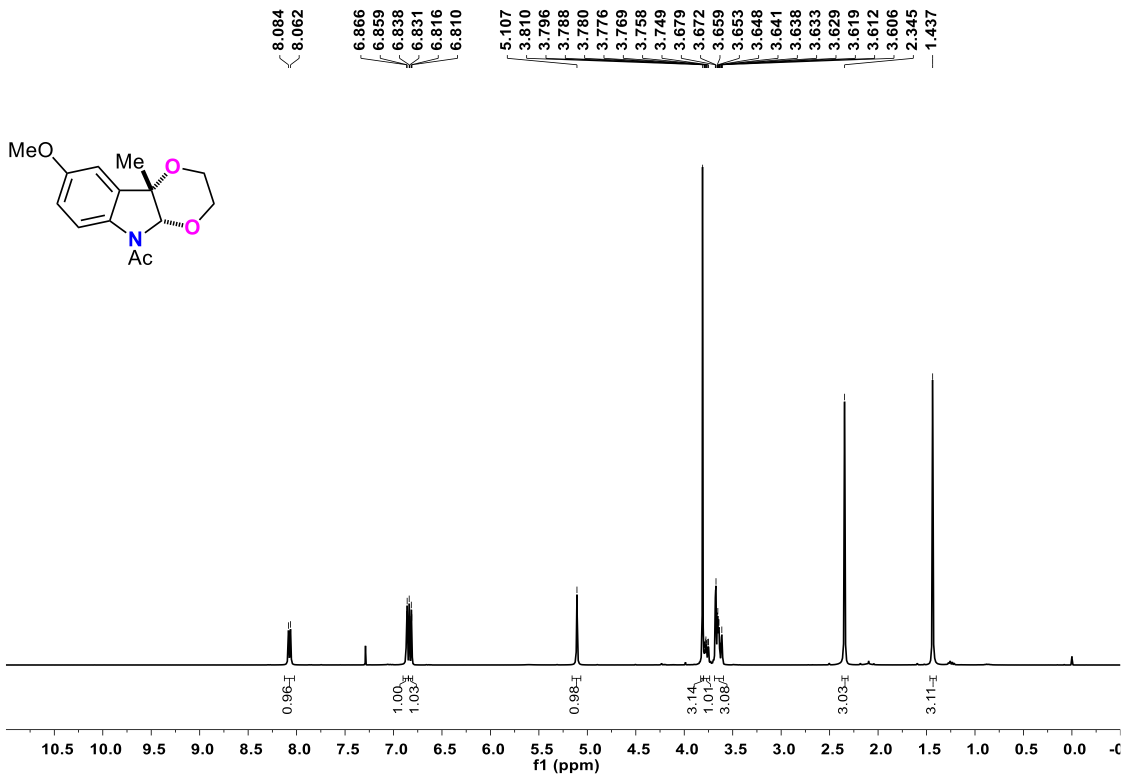

Supplementary Figure 46. ^{1}H **NMR (400 MHz,** $CDCl_3$**) spectrum of 3ca**

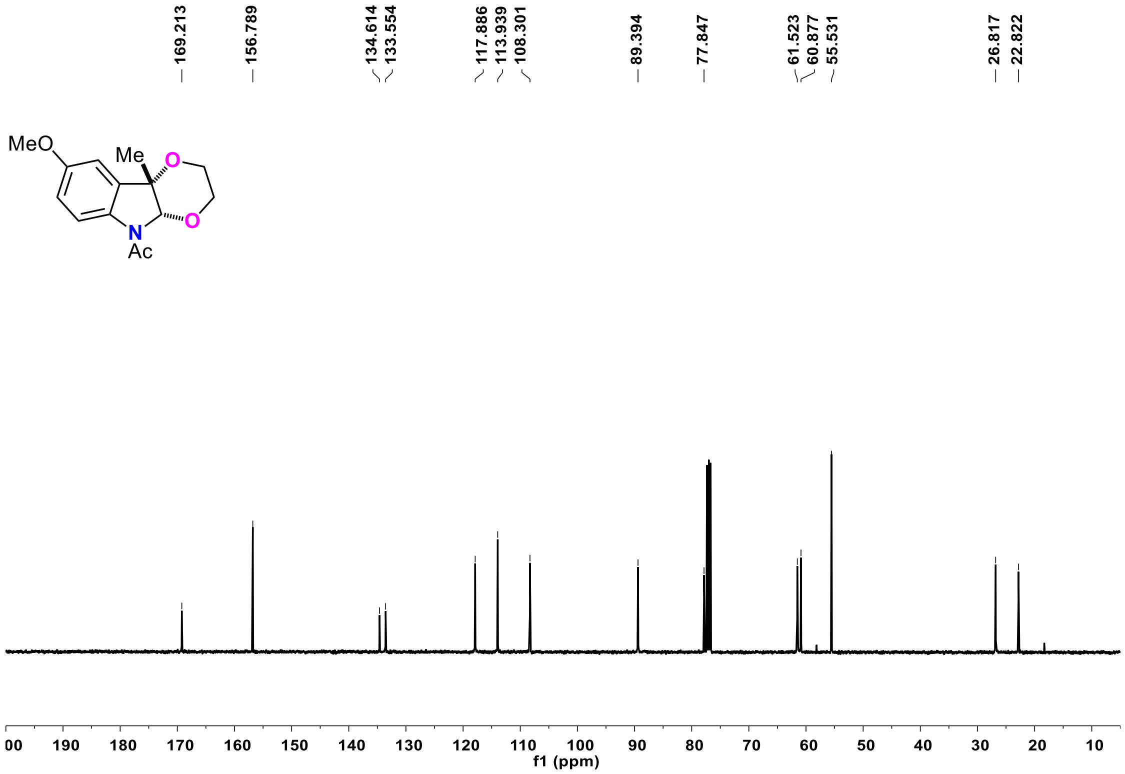

Supplementary Figure 47. ^{13}C **NMR (101 MHz,** $CDCl_3$**) spectrum of 3ca**

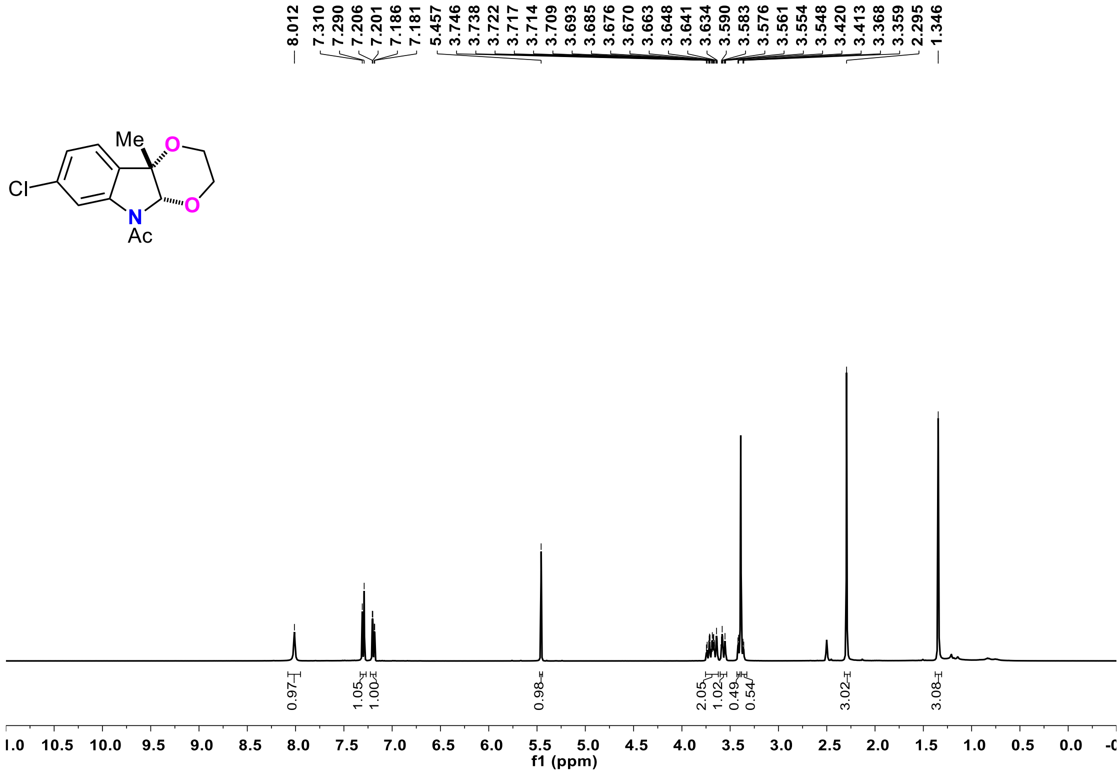

Supplementary Figure 48. ^{1}H NMR (400 MHz, DMSO-d$_6$) spectrum of 3da

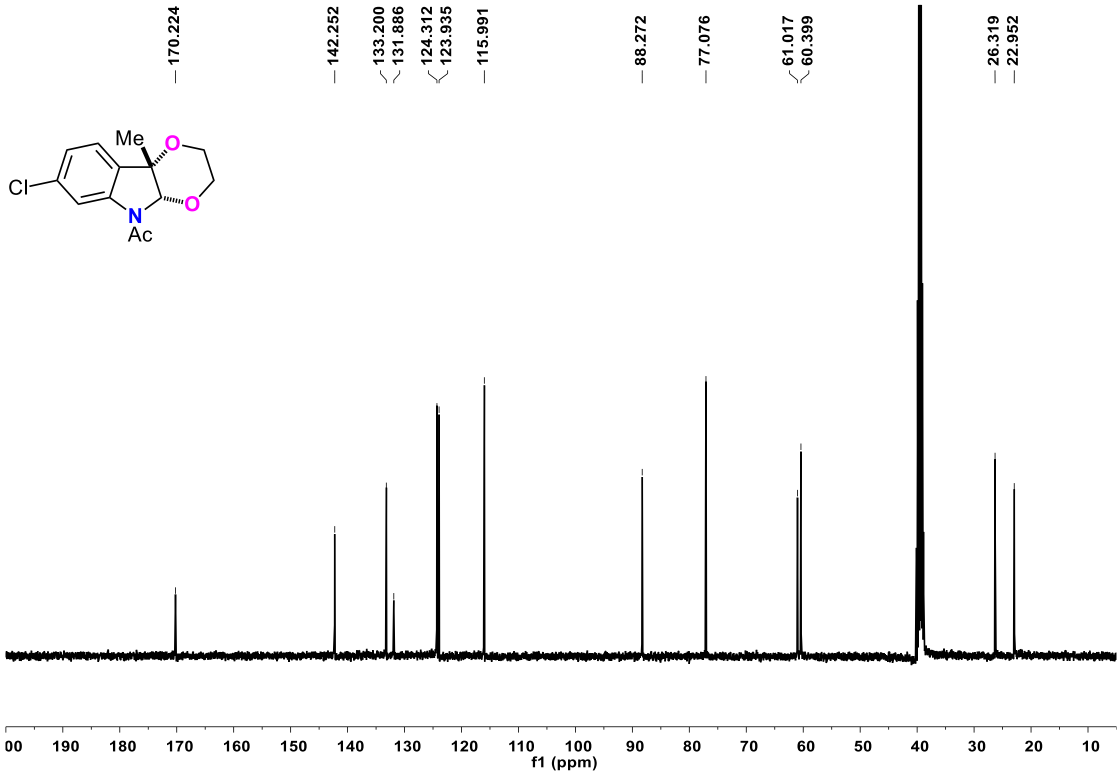

Supplementary Figure 49. ^{13}C NMR (101 MHz, DMSO-d$_6$) spectrum of 3da

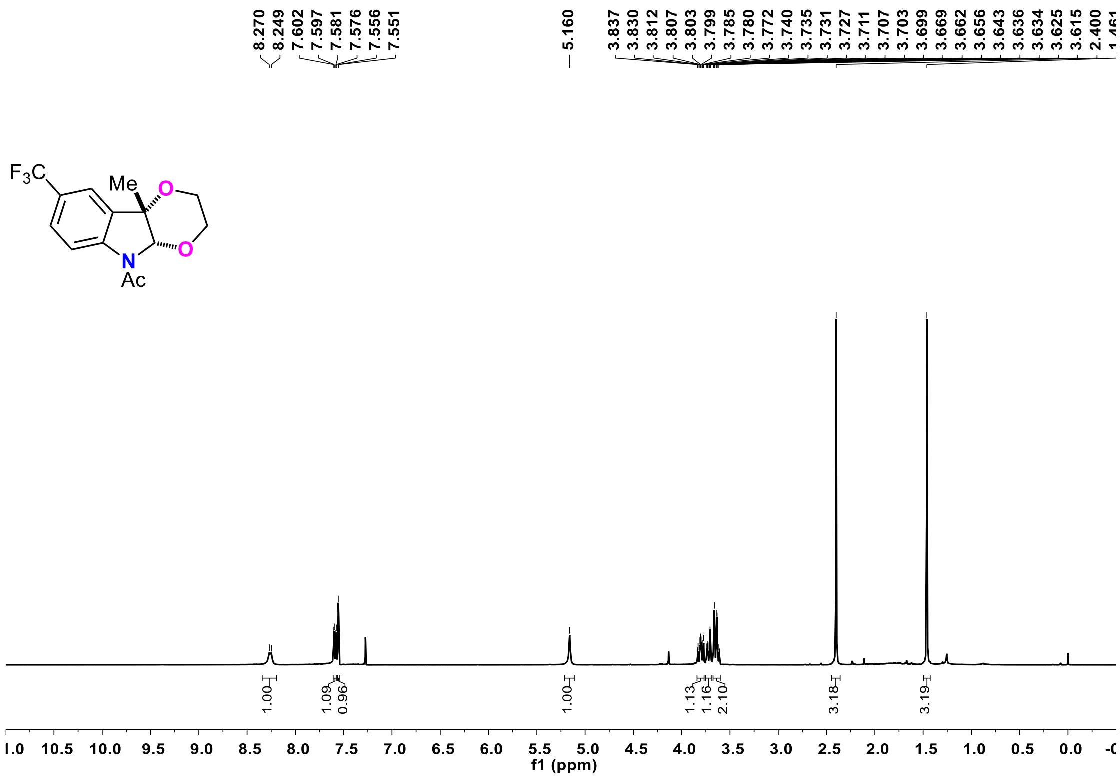

Supplementary Figure 50. ^{1}H NMR (400 MHz, $CDCl_3$) spectrum of 3ea

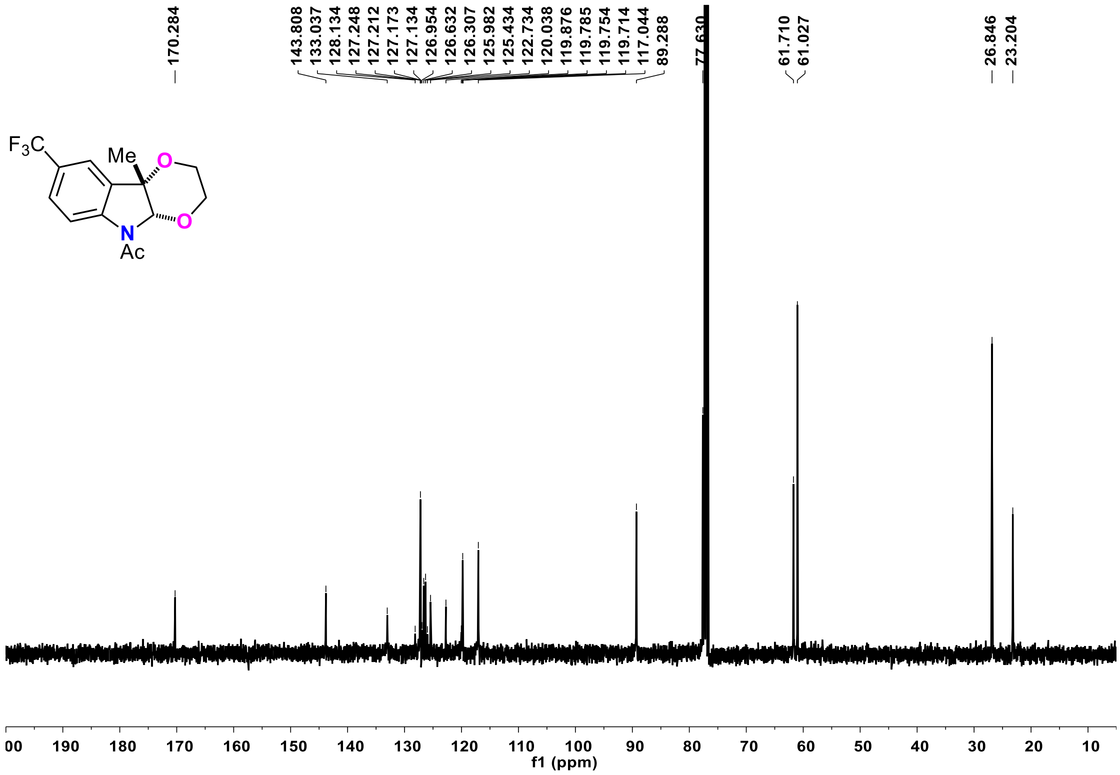

Supplementary Figure 51. ^{13}C NMR (101 MHz, $CDCl_3$) spectrum of 3ea

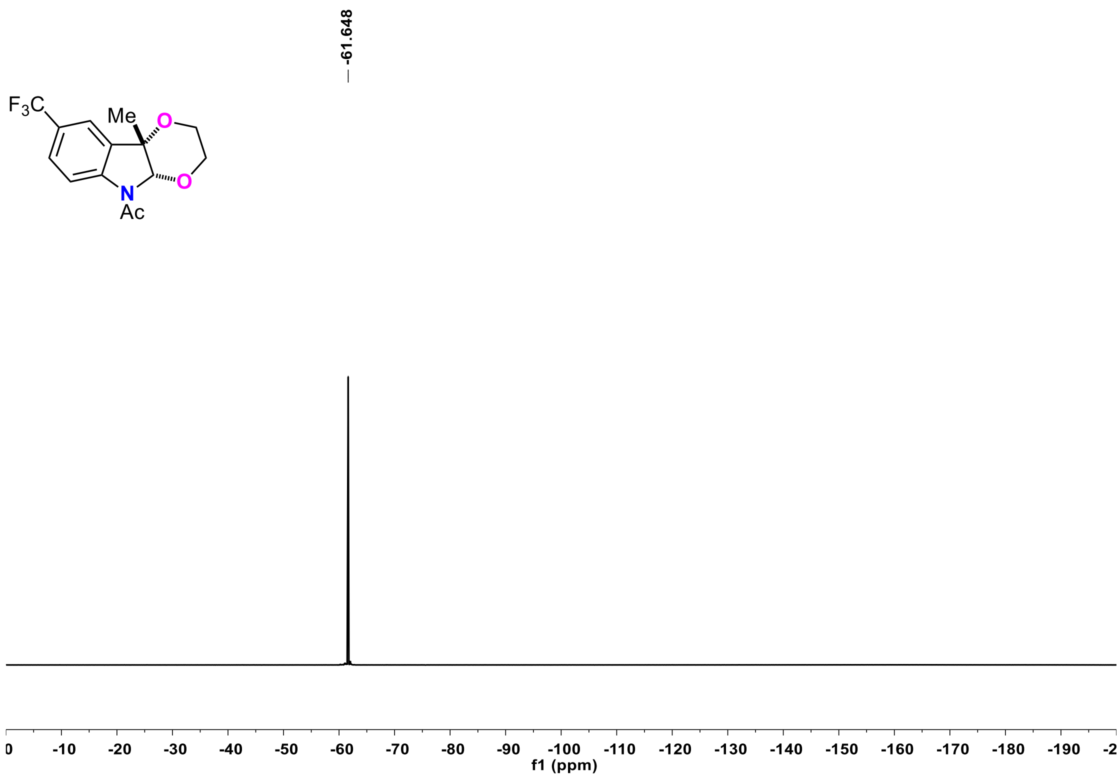

Supplementary Figure 52. ^{19}**F NMR (377 MHz, CDCl$_3$) spectrum of 3ea**

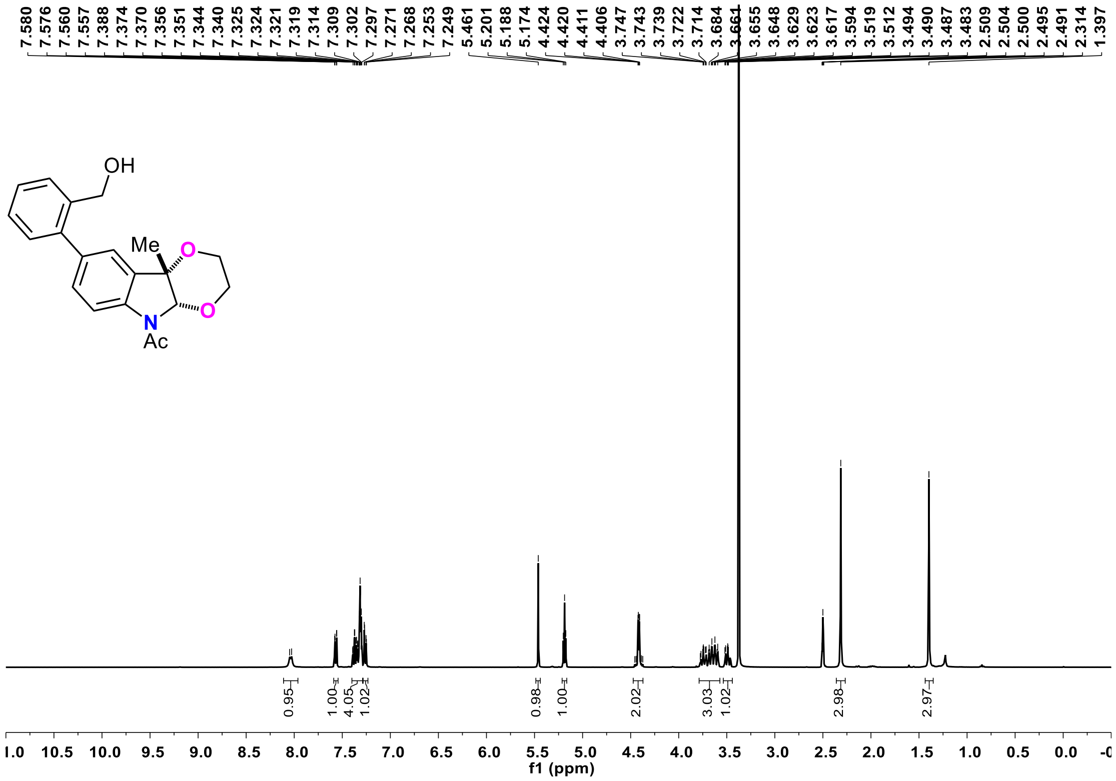

Supplementary Figure 53. **^{1}H NMR (400 MHz, DMSO-d$_6$) spectrum of 3fa**

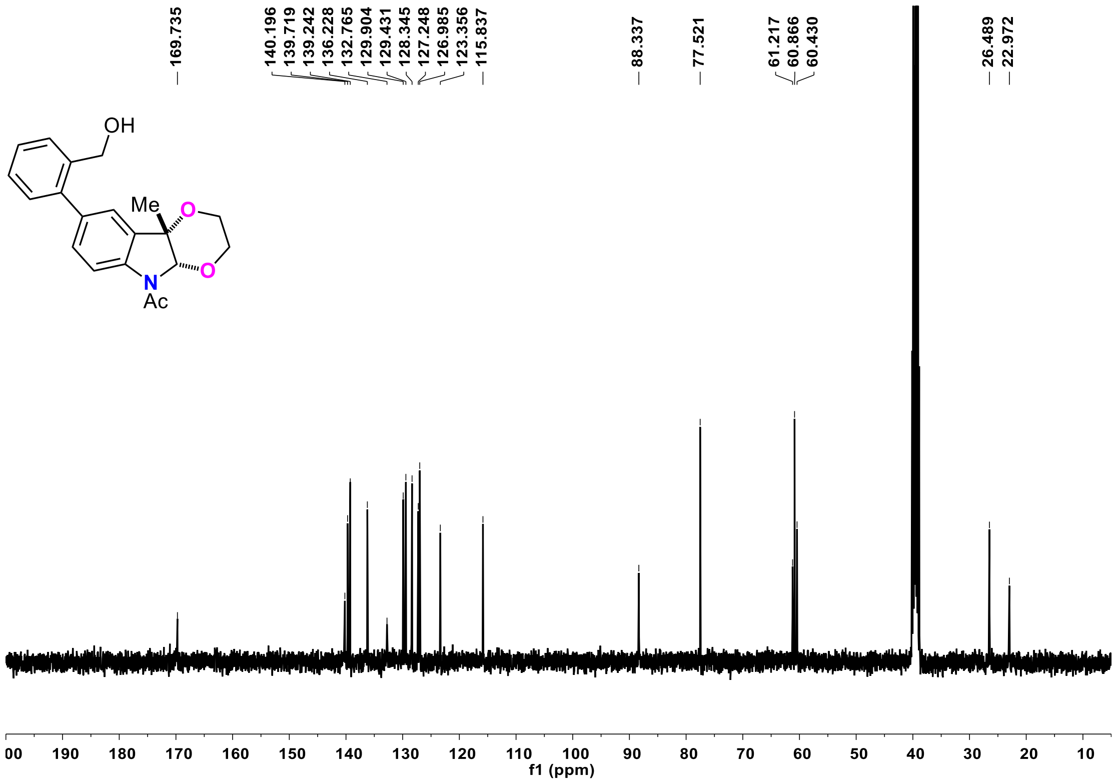

Supplementary Figure 54. **^{13}C NMR (101 MHz, DMSO-d$_6$) spectrum of 3fa**

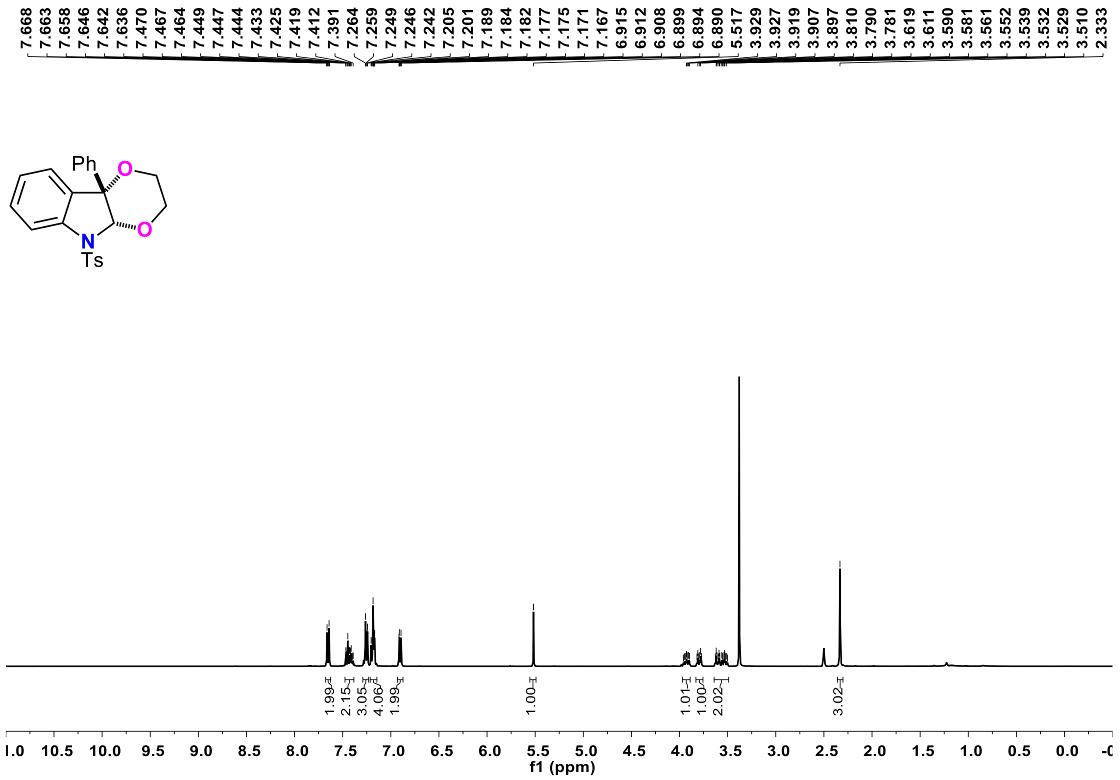

Supplementary Figure 55. ^{1}H NMR (400 MHz, DMSO-d$_6$) spectrum of 3ga

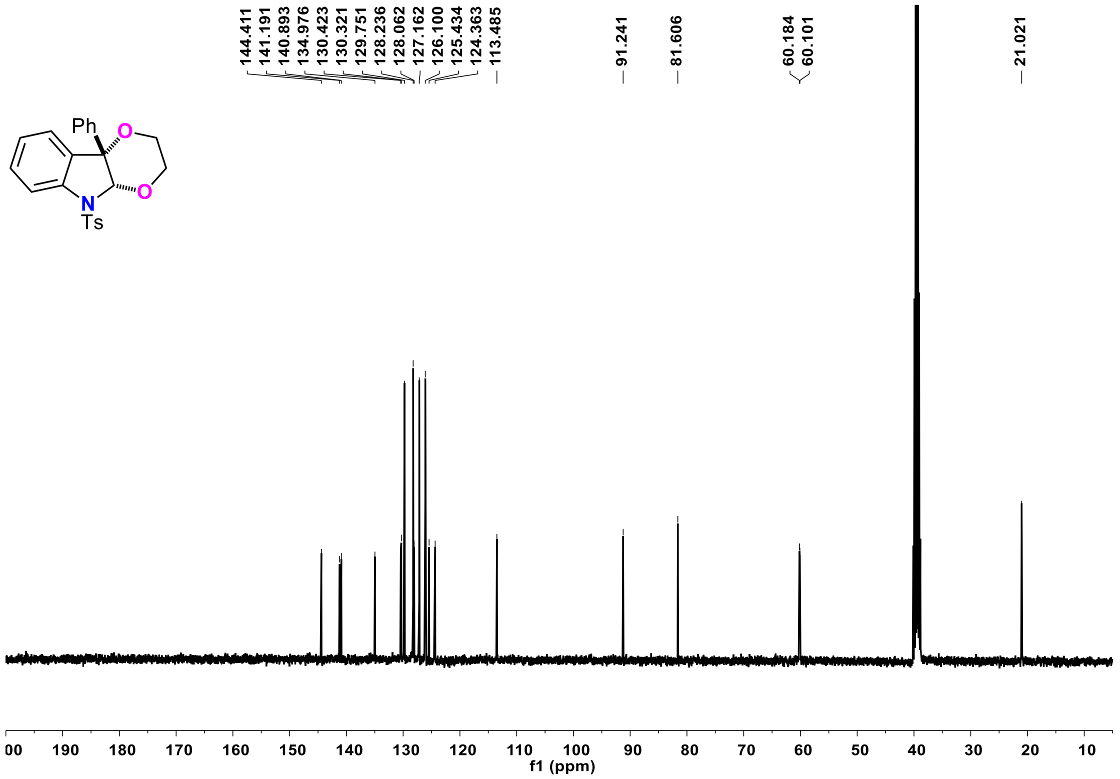

Supplementary Figure 56. ^{13}C NMR (101 MHz, DMSO-d$_6$) spectrum of 3ga

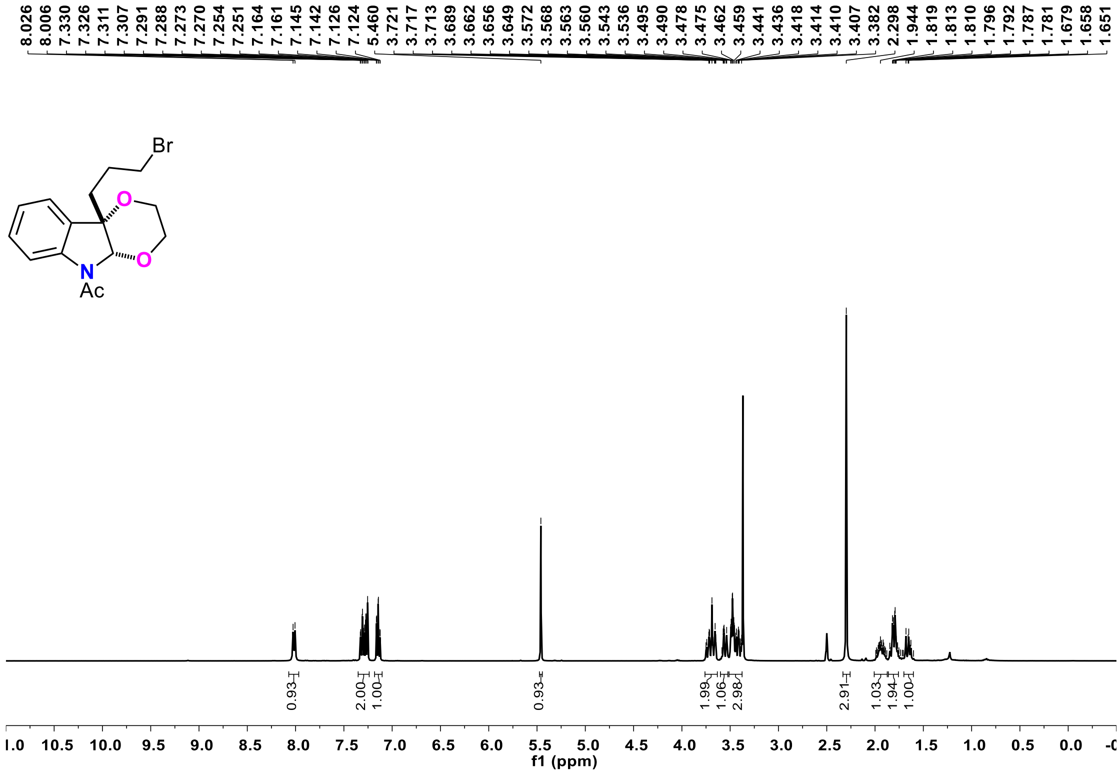

Supplementary Figure 57. ^1H NMR (400 MHz, DMSO-d_6) spectrum of 3ha

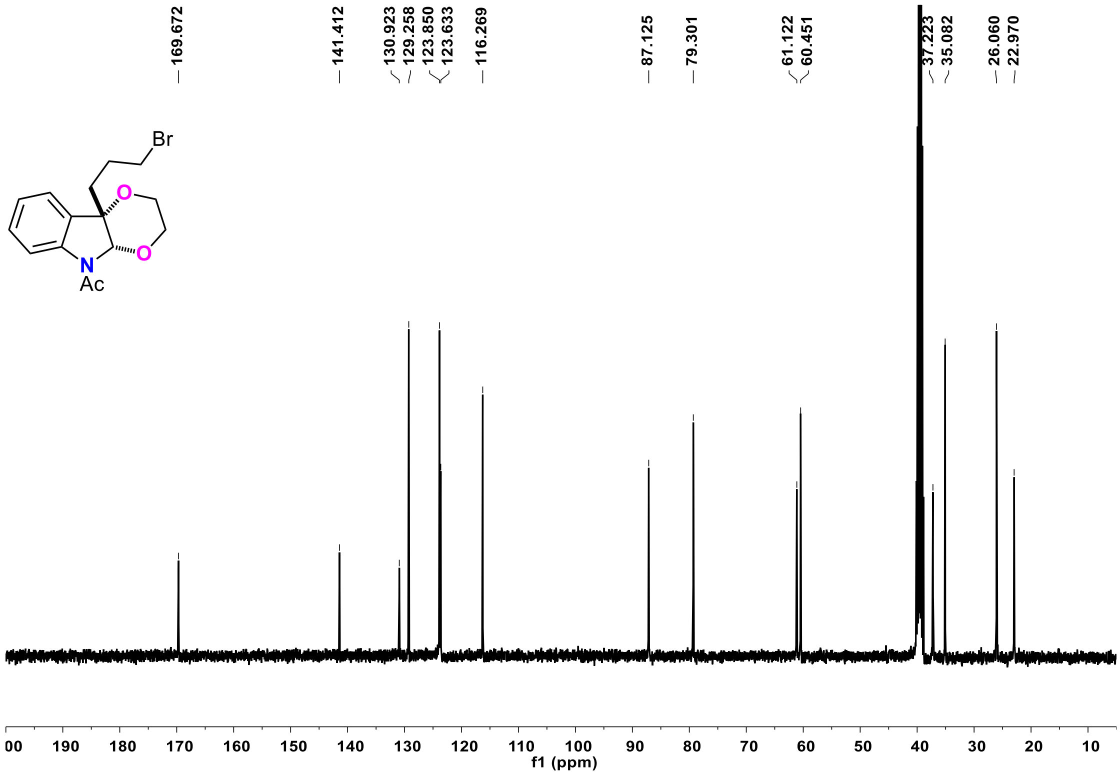

Supplementary Figure 58. ^{13}C NMR (101 MHz, DMSO-d_6) spectrum of 3ha

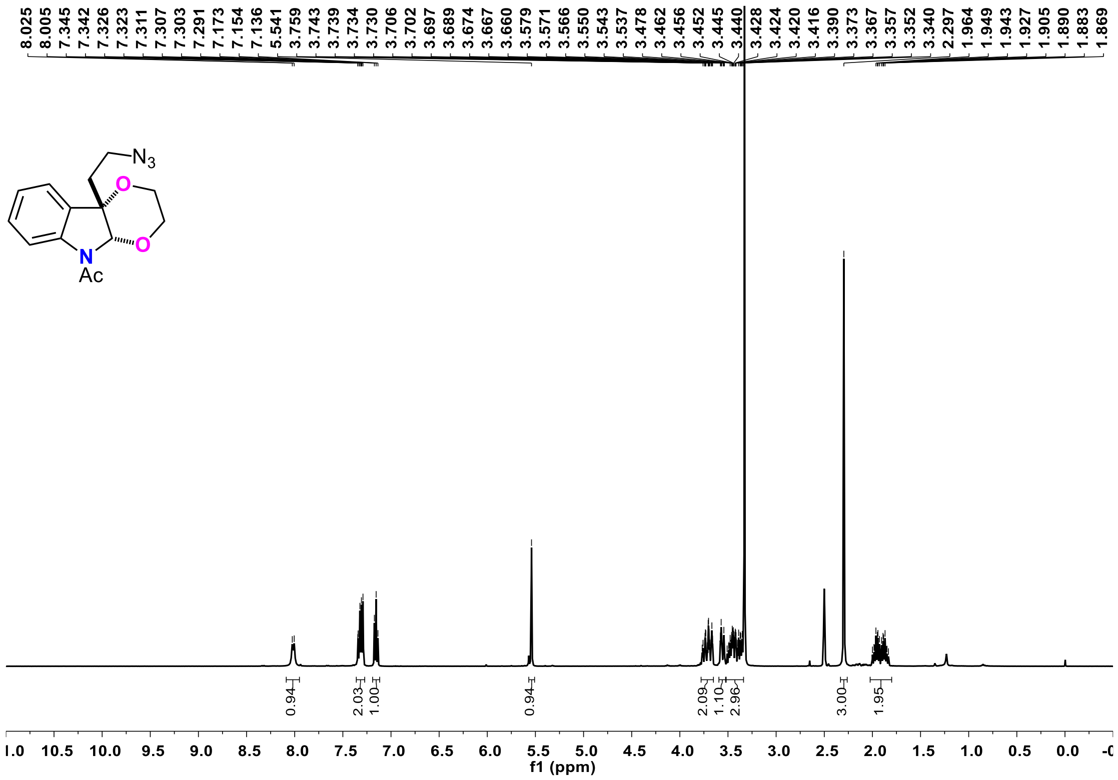

Supplementary Figure 59. ^{1}H NMR (400 MHz, DMSO-d$_6$) spectrum of 3ia

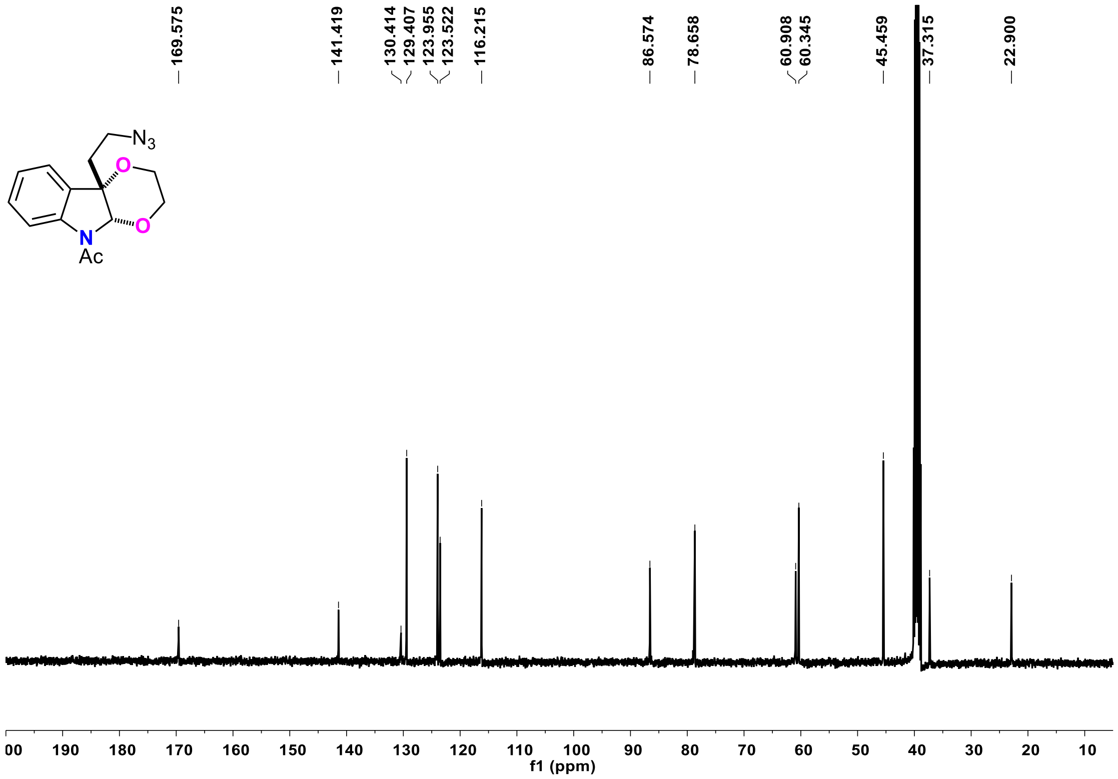

Supplementary Figure 60. ^{13}C NMR (101 MHz, DMSO-d$_6$) spectrum of 3ia

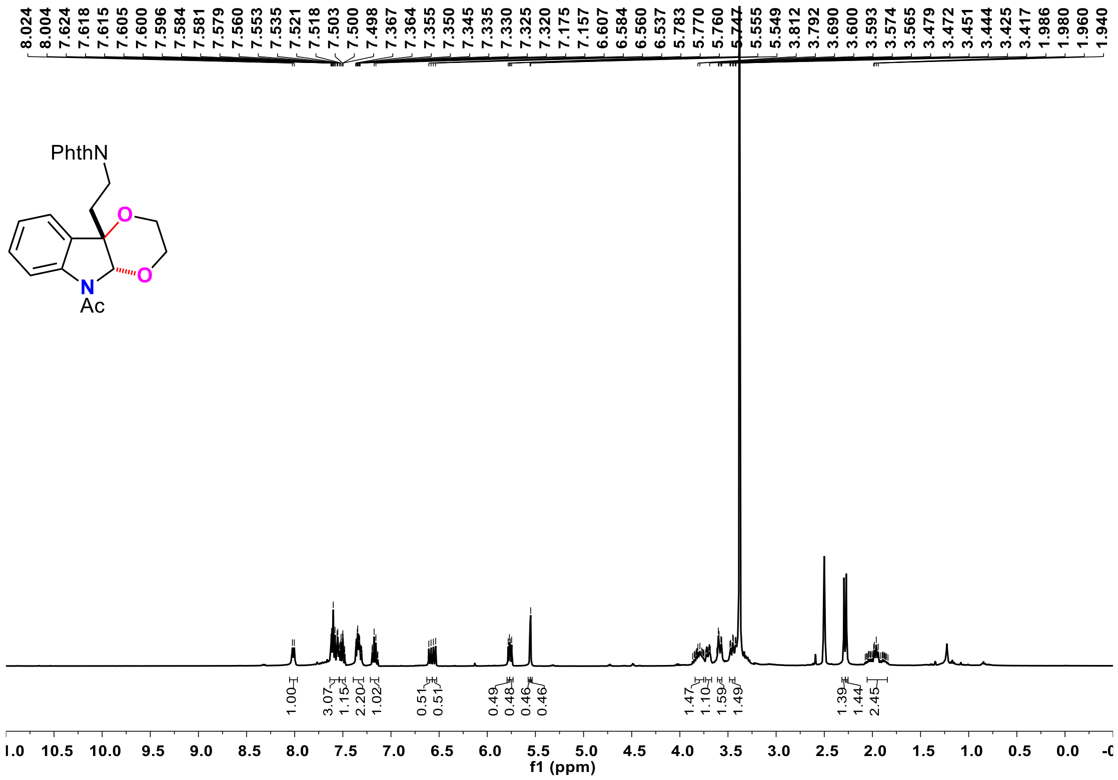

Supplementary Figure 61. ^{1}H NMR (400 MHz, DMSO-d_6) spectrum of 3ja

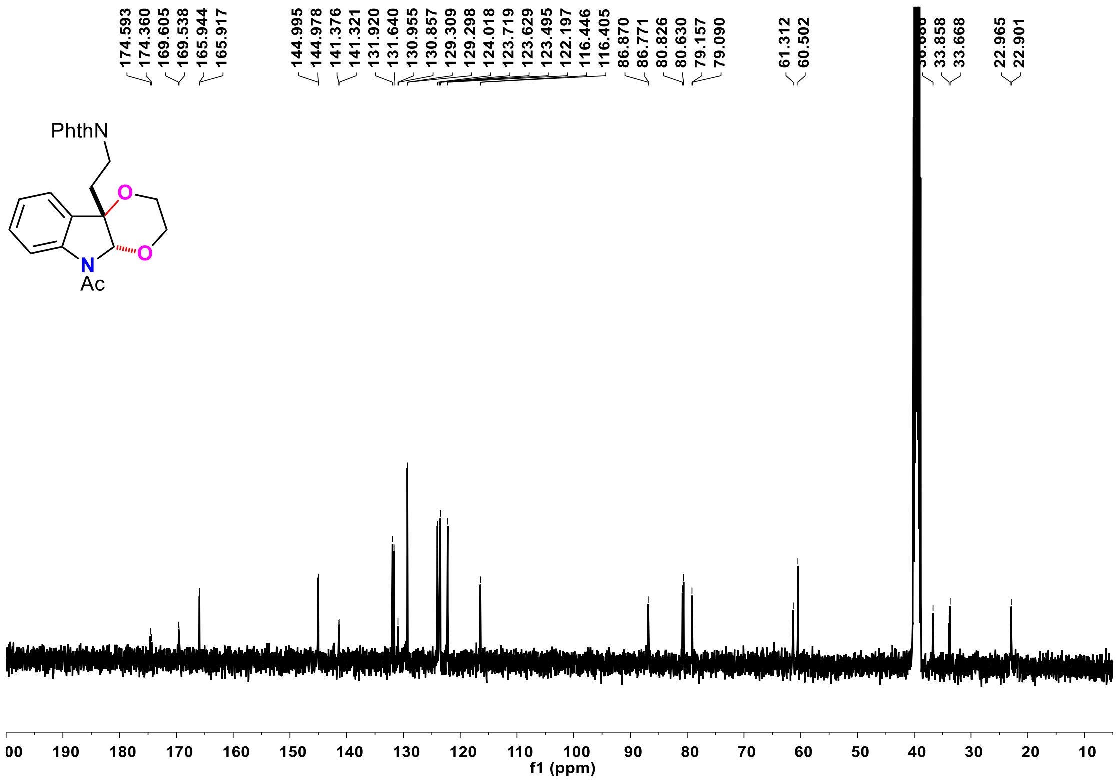

Supplementary Figure 62. ^{13}C NMR (101 MHz, DMSO-d_6) spectrum of 3ja

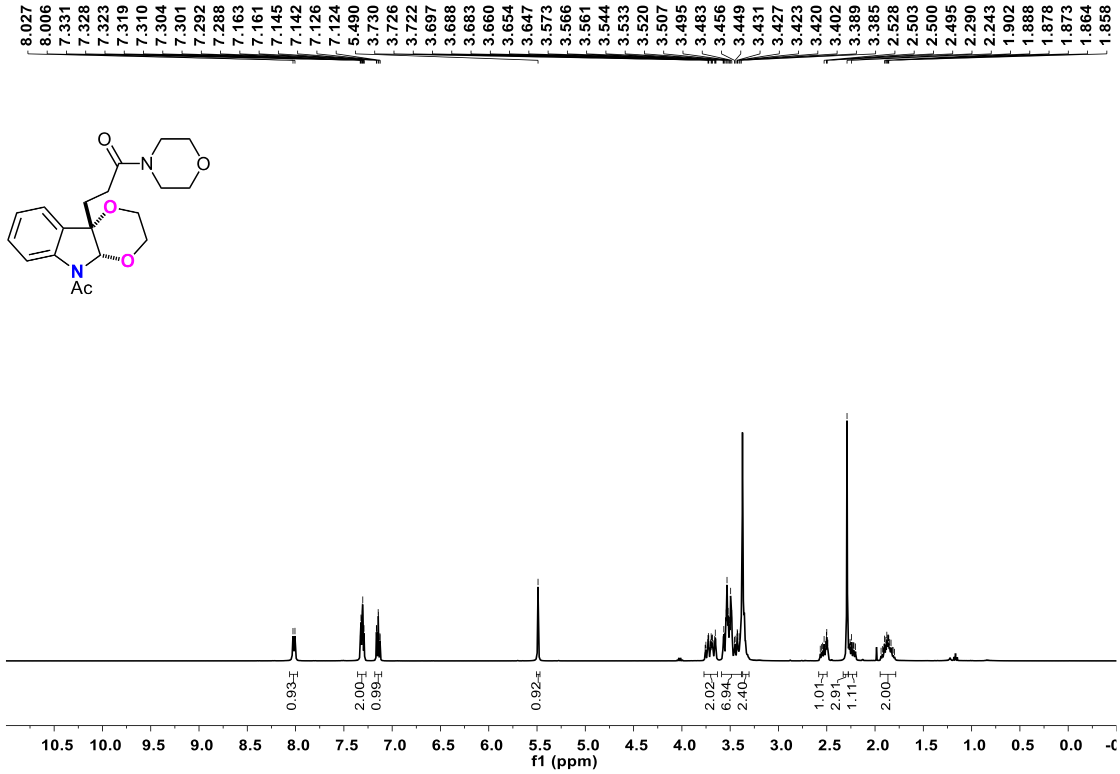

Supplementary Figure 63. ^{1}**H NMR (400 MHz, DMSO-d**$_{6}$**) spectrum of 3ka**

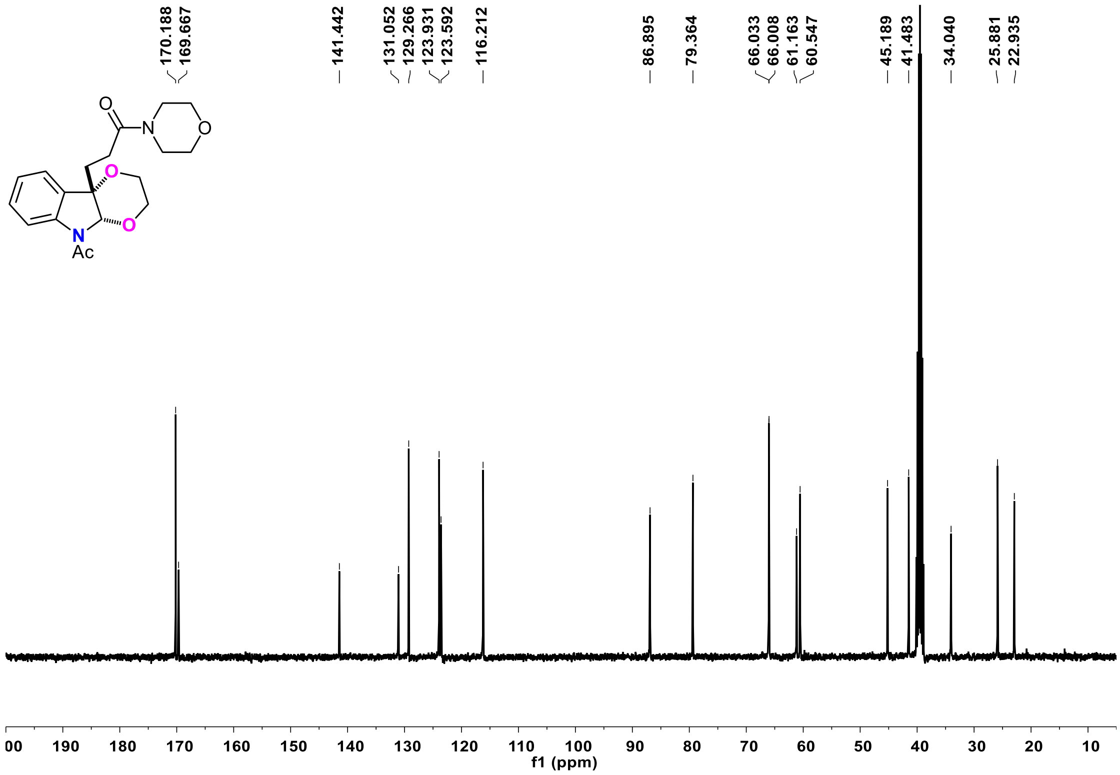

Supplementary Figure 64. ^{13}**C NMR (101 MHz, DMSO-d**$_{6}$**) spectrum of 3ka**

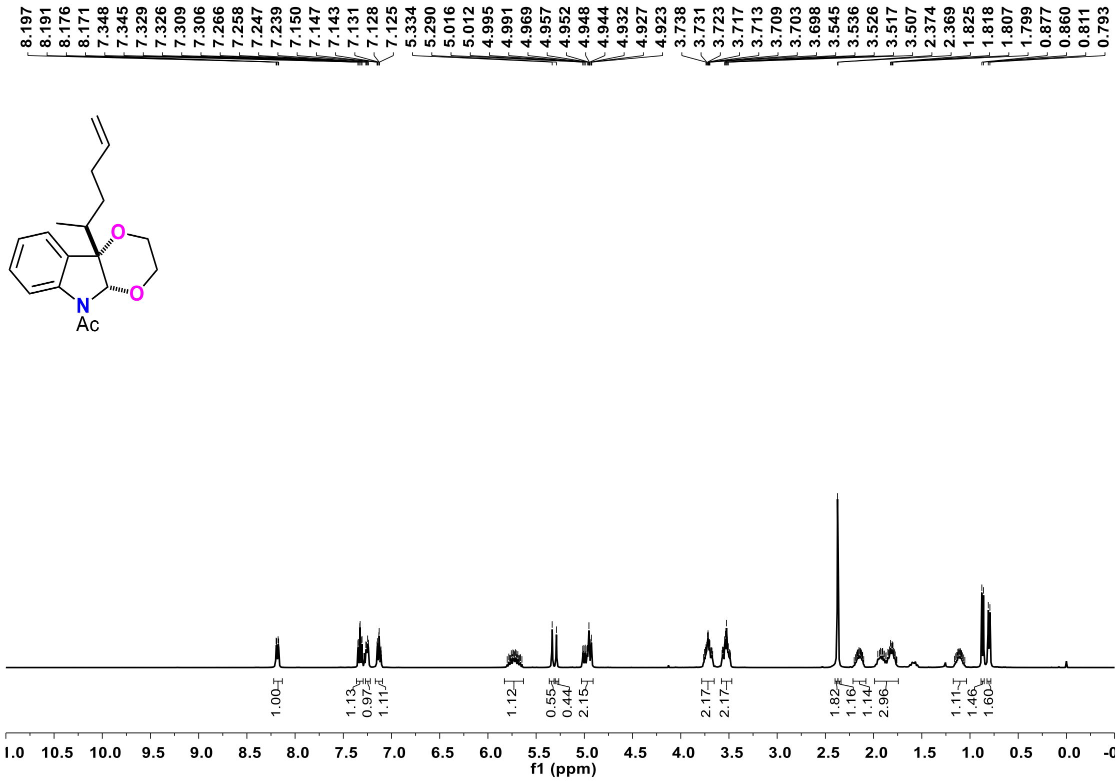

Supplementary Figure 65. ^{1}H NMR (400 MHz, $CDCl_3$) spectrum of 3la

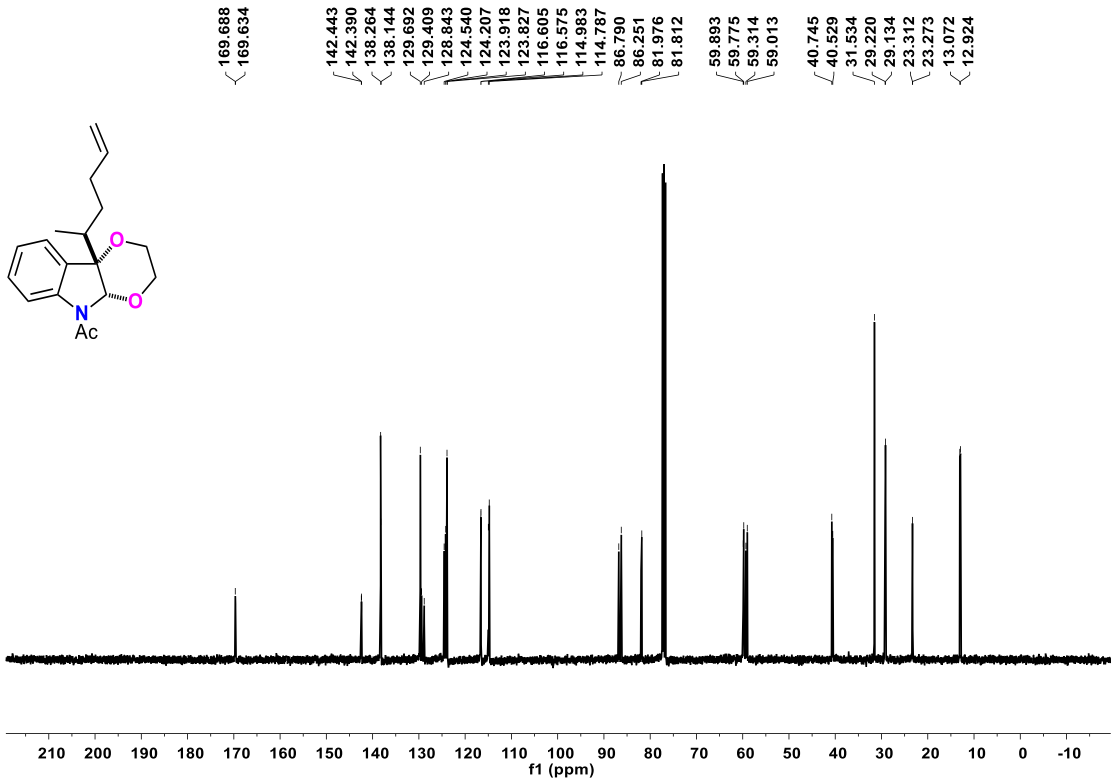

Supplementary Figure 66. ^{13}C NMR (101 MHz, $CDCl_3$) spectrum of 3la

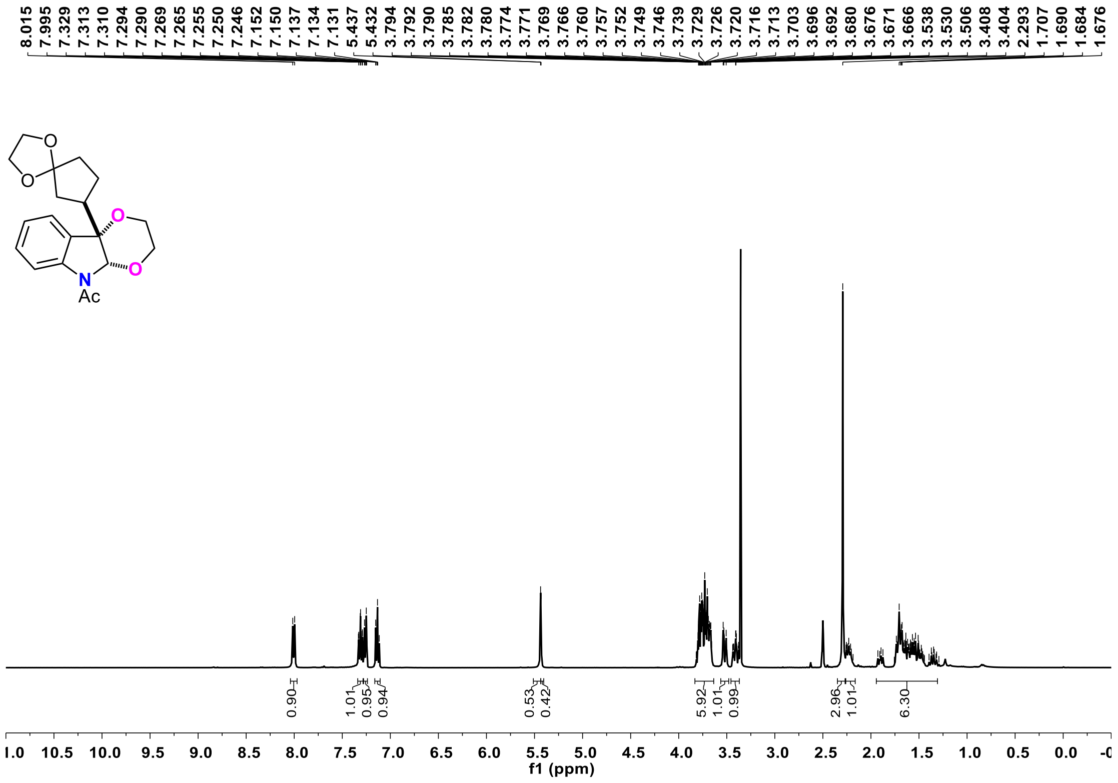

Supplementary Figure 67. ^{1}H NMR (400 MHz, DMSO-d$_6$) spectrum of 3ma

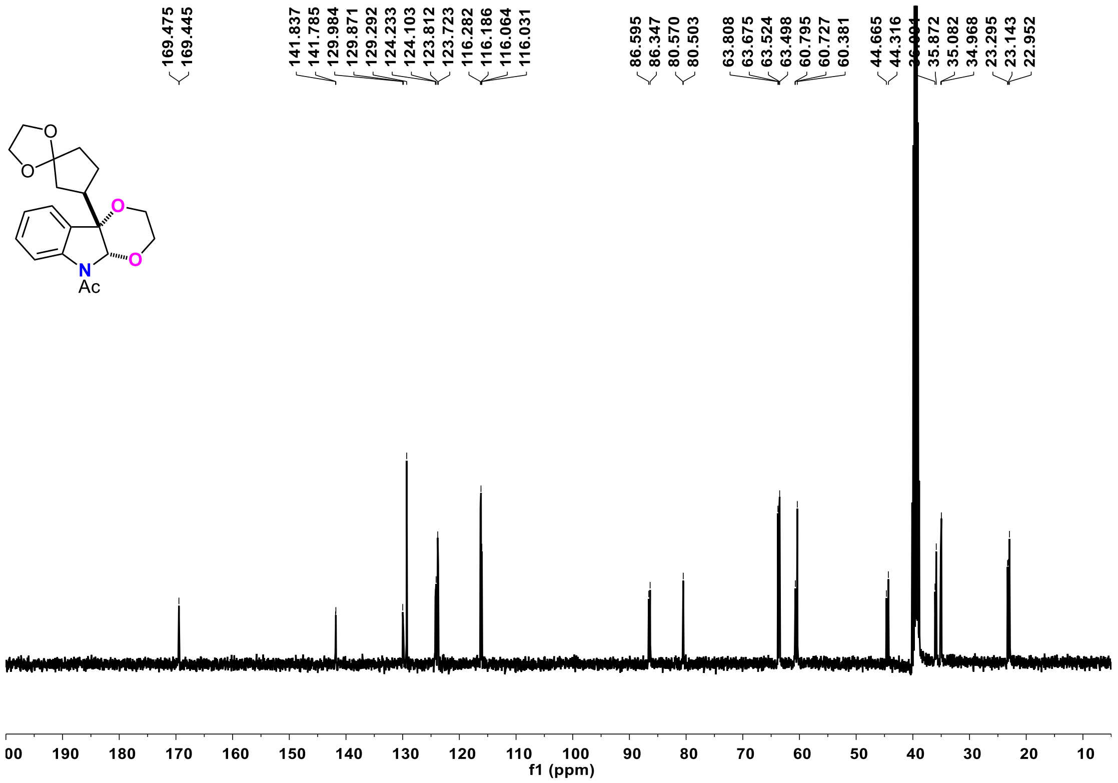

Supplementary Figure 68. ^{13}C NMR (101 MHz, DMSO-d$_6$) spectrum of 3ma

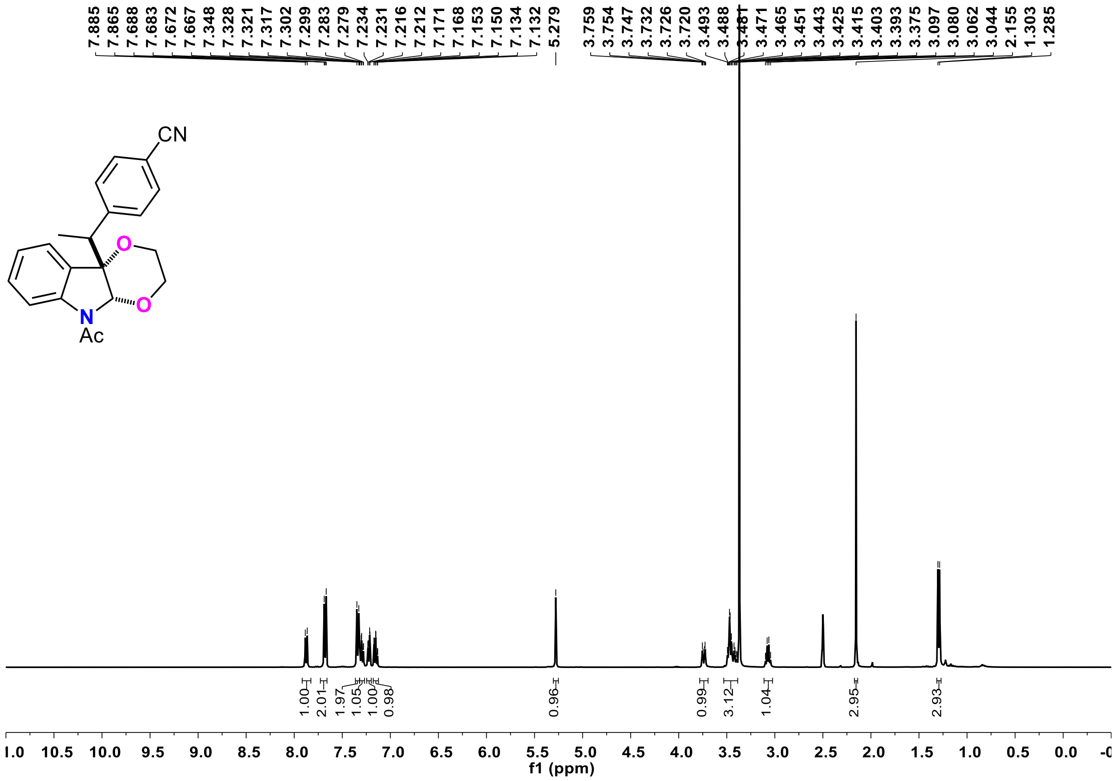

Supplementary Figure 69. ^{1}**H NMR (400 MHz, DMSO-d**$_6$**) spectrum of 3na**

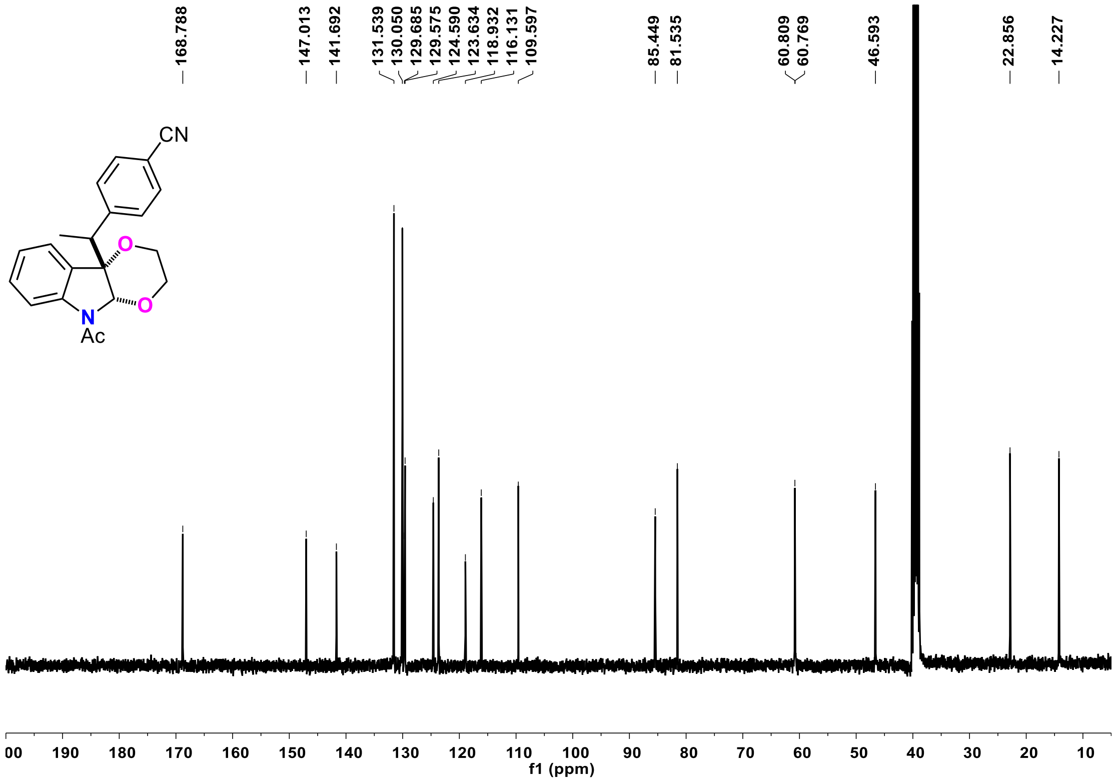

Supplementary Figure 70. ^{13}**C NMR (101 MHz, DMSO-d**$_6$**) spectrum of 3na**

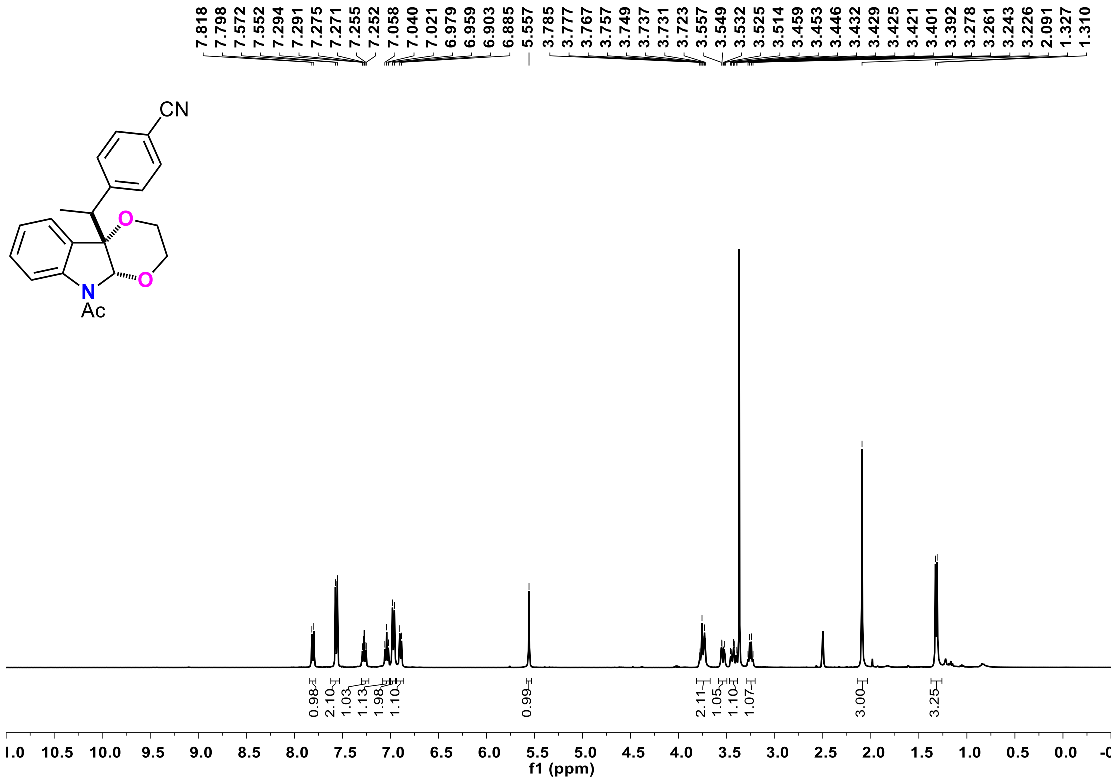

Supplementary Figure 71. ^{1}**H NMR (400 MHz, DMSO-d$_{6}$) spectrum of 3na′**

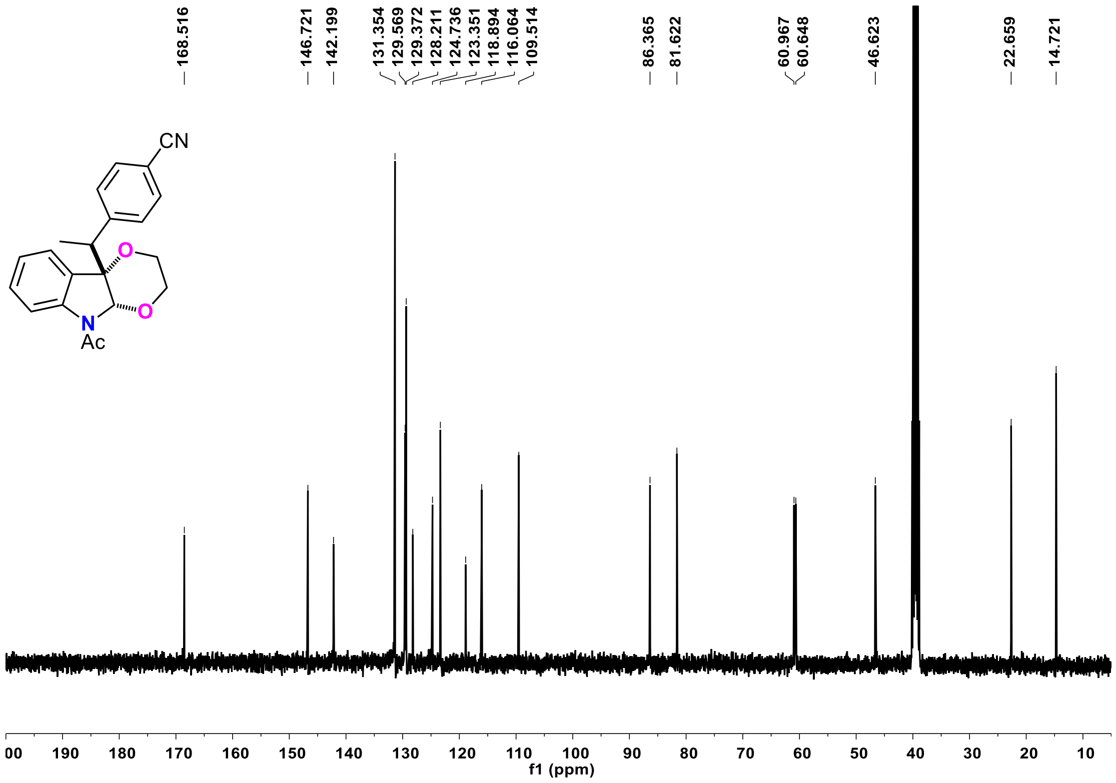

Supplementary Figure 72. ^{13}**C NMR (101 MHz, DMSO-d$_{6}$) spectrum of 3na′**

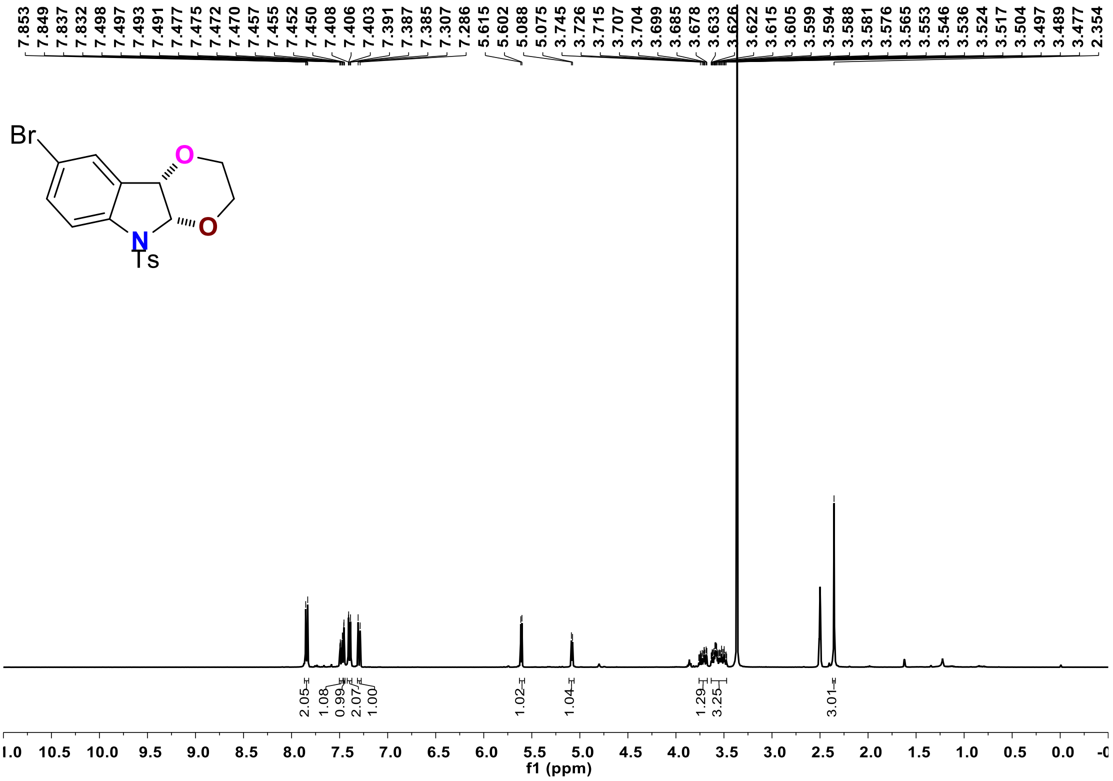

Supplementary Figure 73. ^{1}**H NMR (400 MHz, DMSO-d$_6$) spectrum of 3oa**

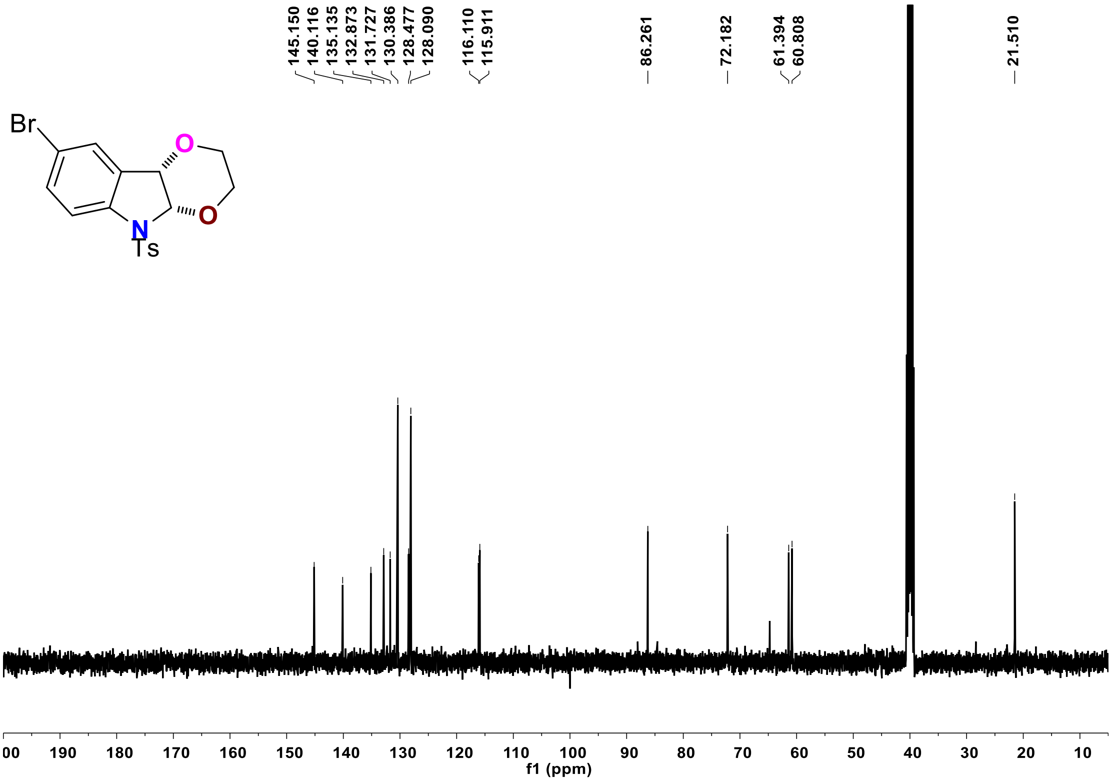

Supplementary Figure 74. ^{13}**C NMR (101 MHz, DMSO-d$_6$) spectrum of 3oa**

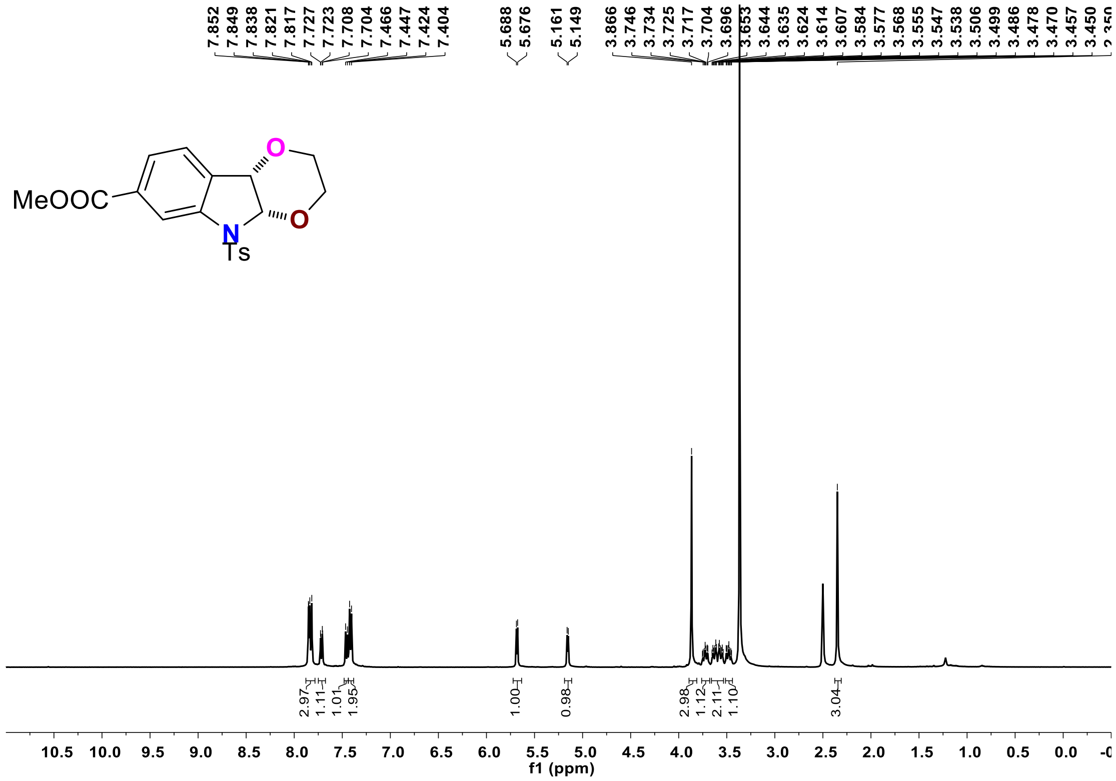

Supplementary Figure 75. ^{1}**H NMR (400 MHz, DMSO-d$_6$) spectrum of 3pa**

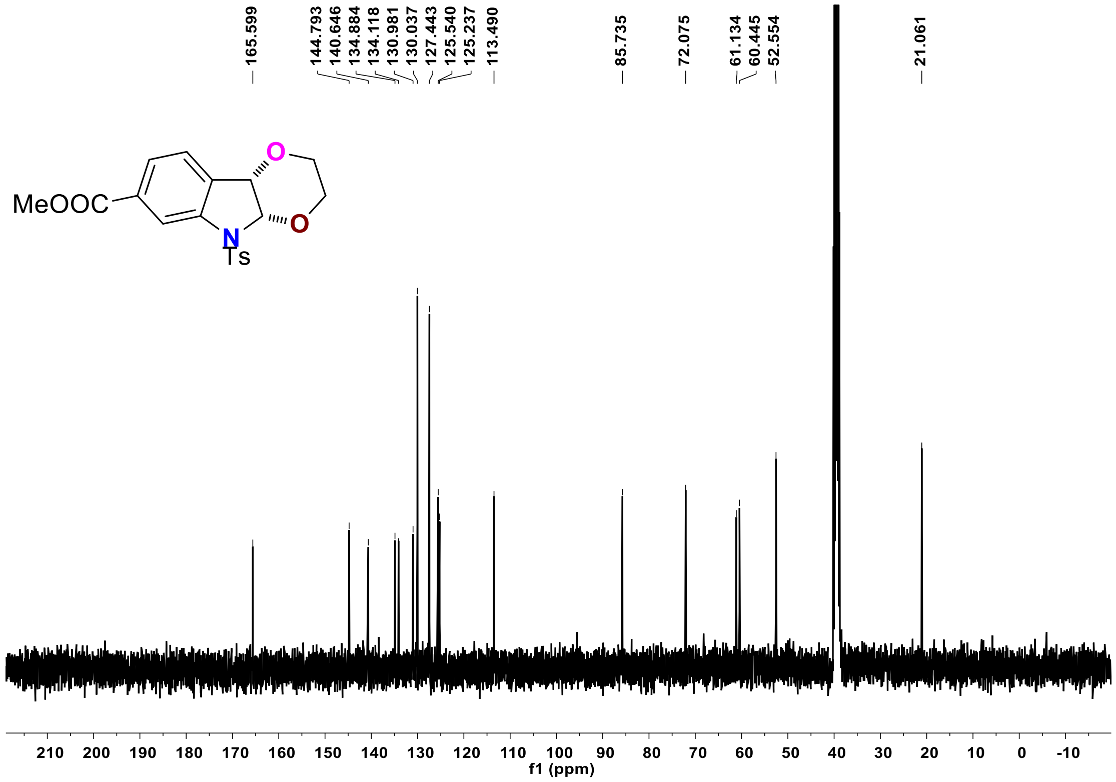

Supplementary Figure 76. ^{13}**C NMR (101 MHz, DMSO-d$_6$) spectrum of 3pa**

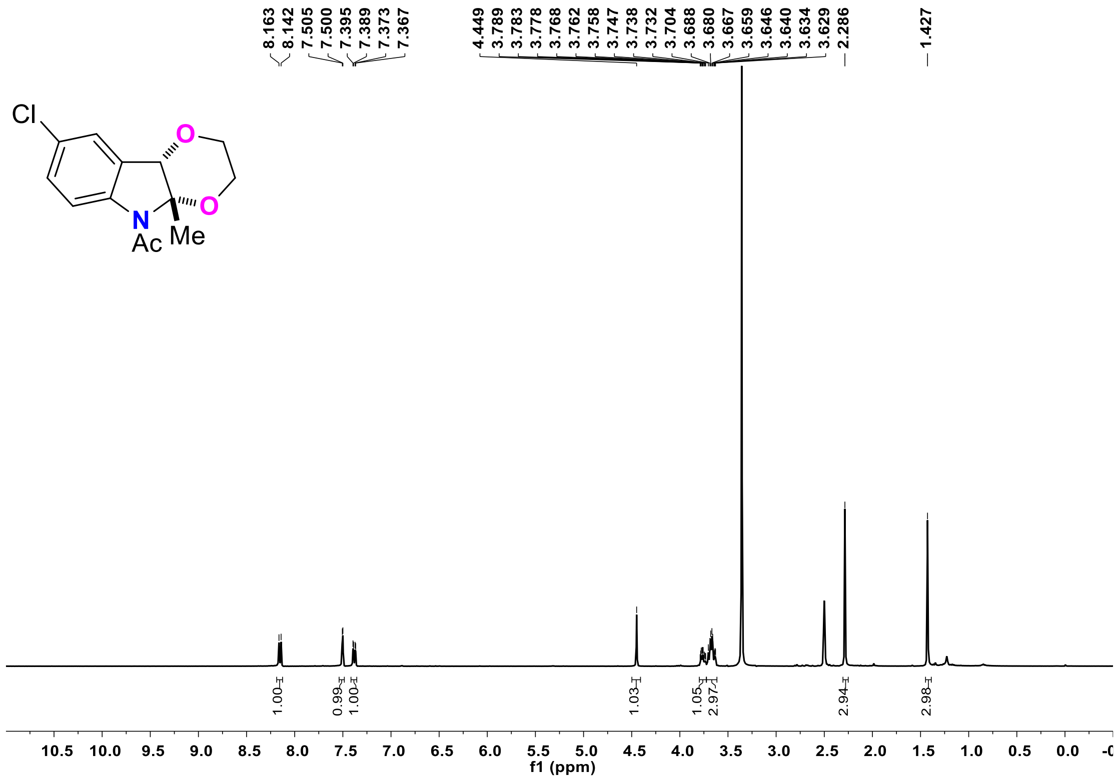

Supplementary Figure 77. ^{1}H NMR (400 MHz, DMSO-d$_6$) spectrum of 3qa

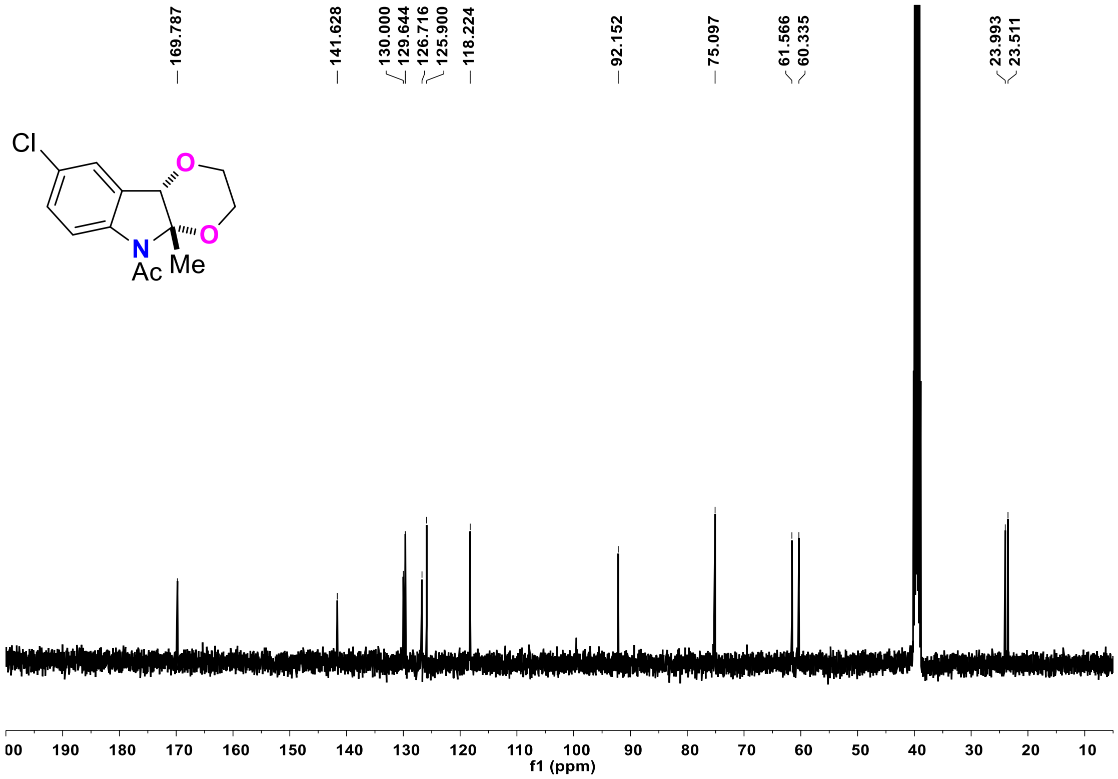

Supplementary Figure 78. ^{13}C NMR (101 MHz, DMSO-d$_6$) spectrum of 3qa

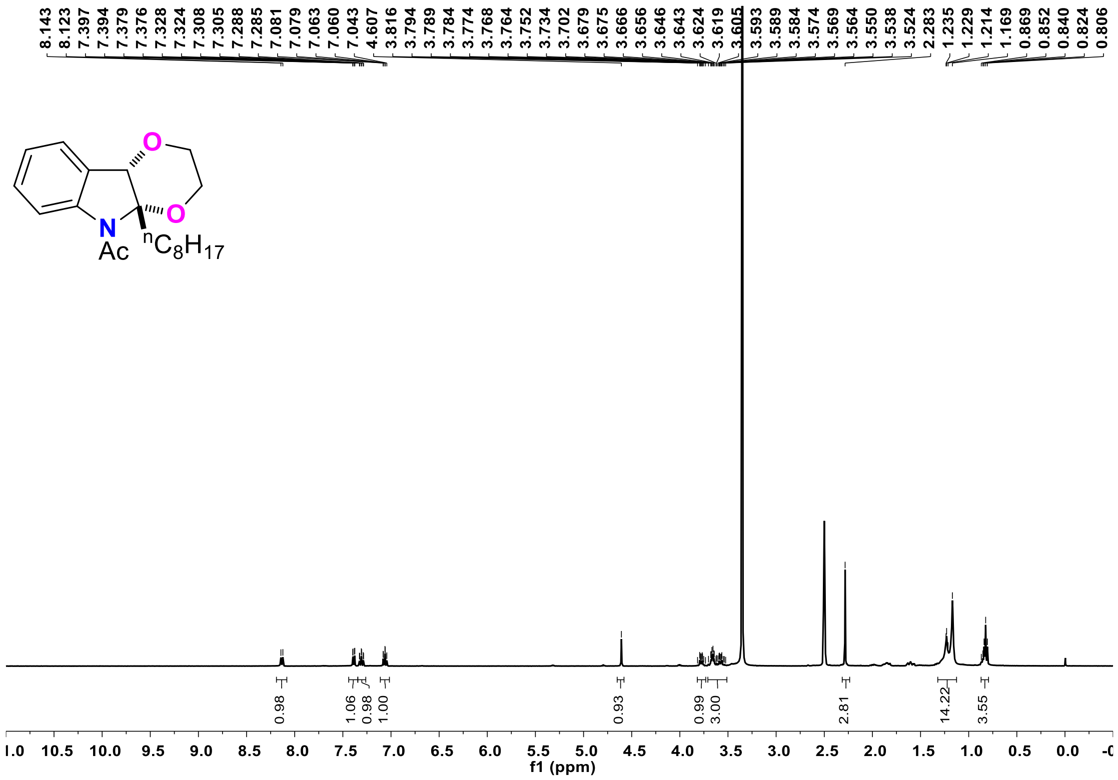

Supplementary Figure 79. ^{1}H NMR (400 MHz, DMSO-d_6) spectrum of 3ra

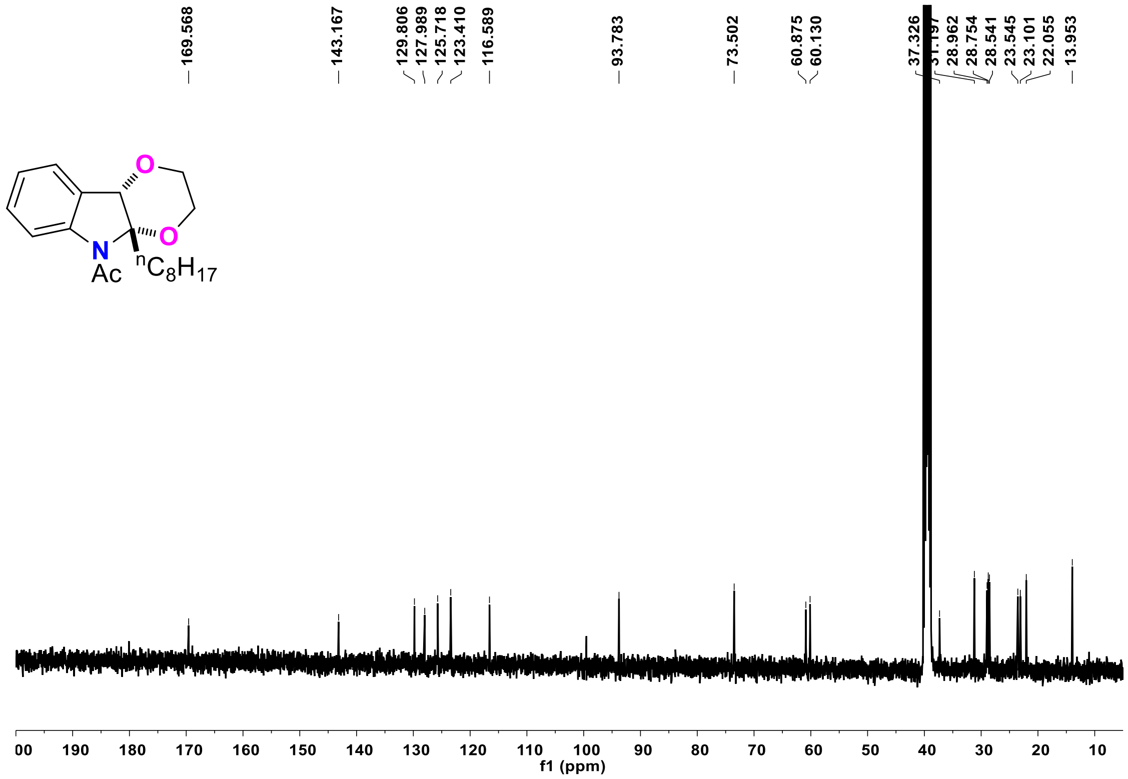

Supplementary Figure 80. ^{13}C NMR (101 MHz, DMSO-d_6) spectrum of 3ra

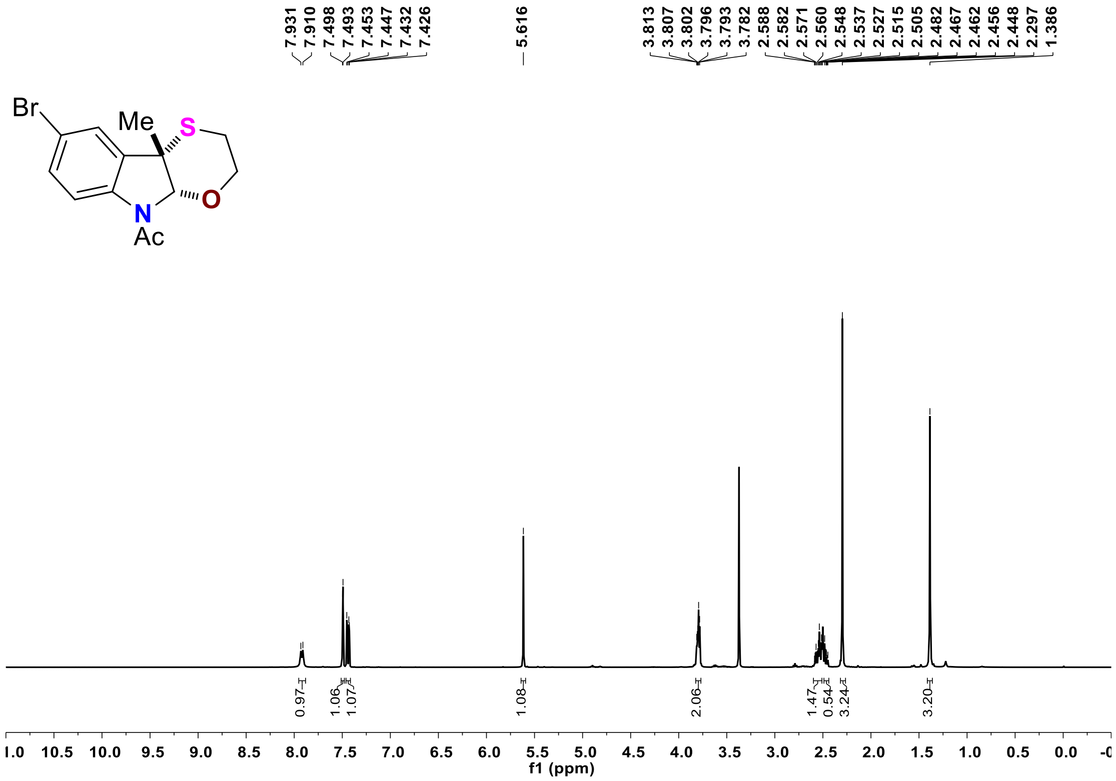

Supplementary Figure 81. ^{1}H **NMR (400 MHz, DMSO-d$_6$) spectrum of 3sa**

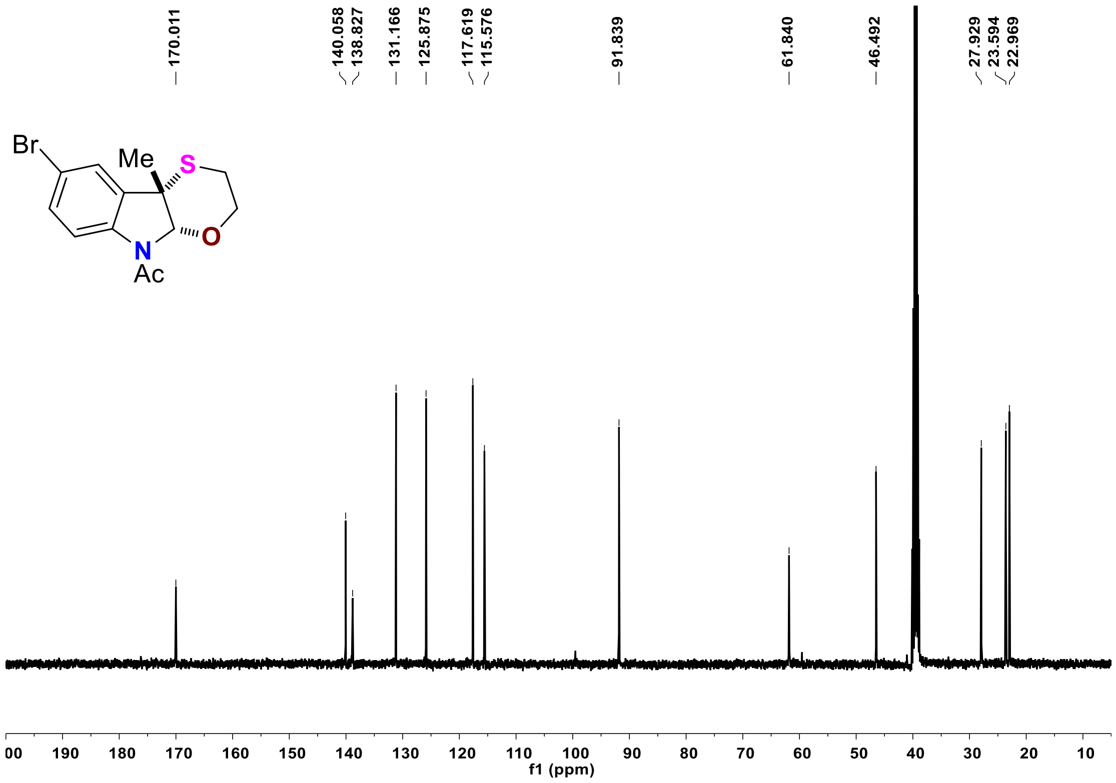

Supplementary Figure 82. ^{13}C **NMR (101 MHz, DMSO-d$_6$) spectrum of 3sa**

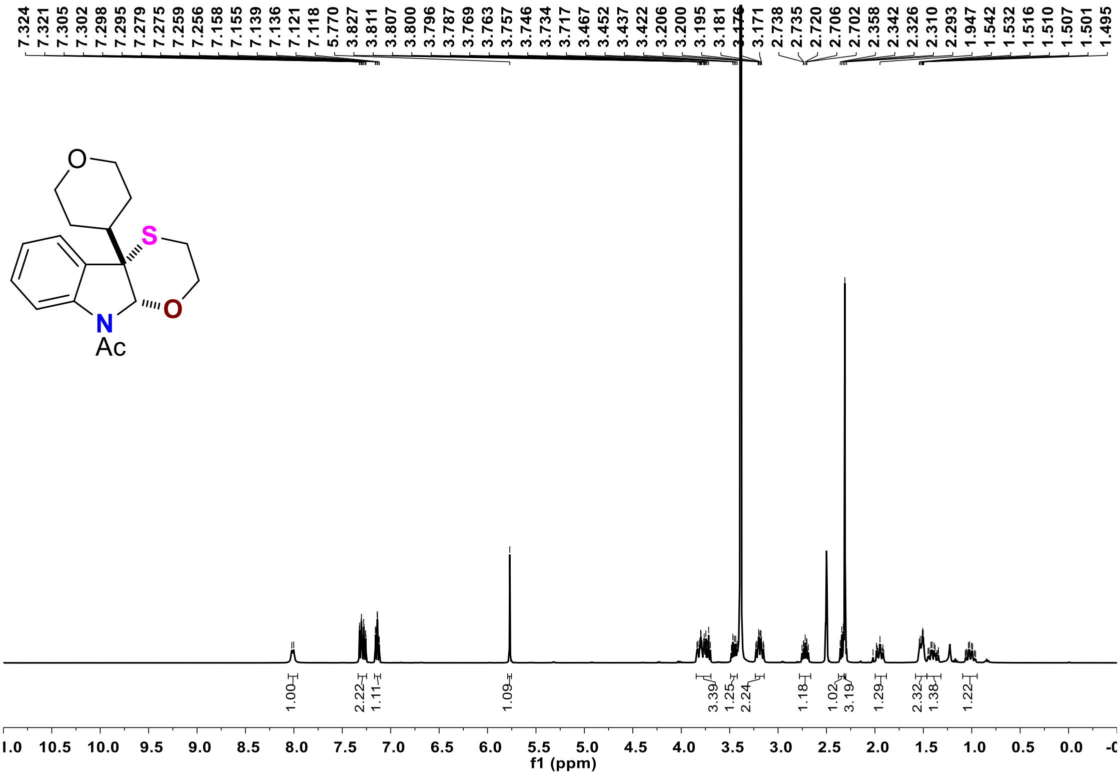

Supplementary Figure 83. ^{1}**H NMR (400 MHz, DMSO-d$_6$) spectrum of 3ta**

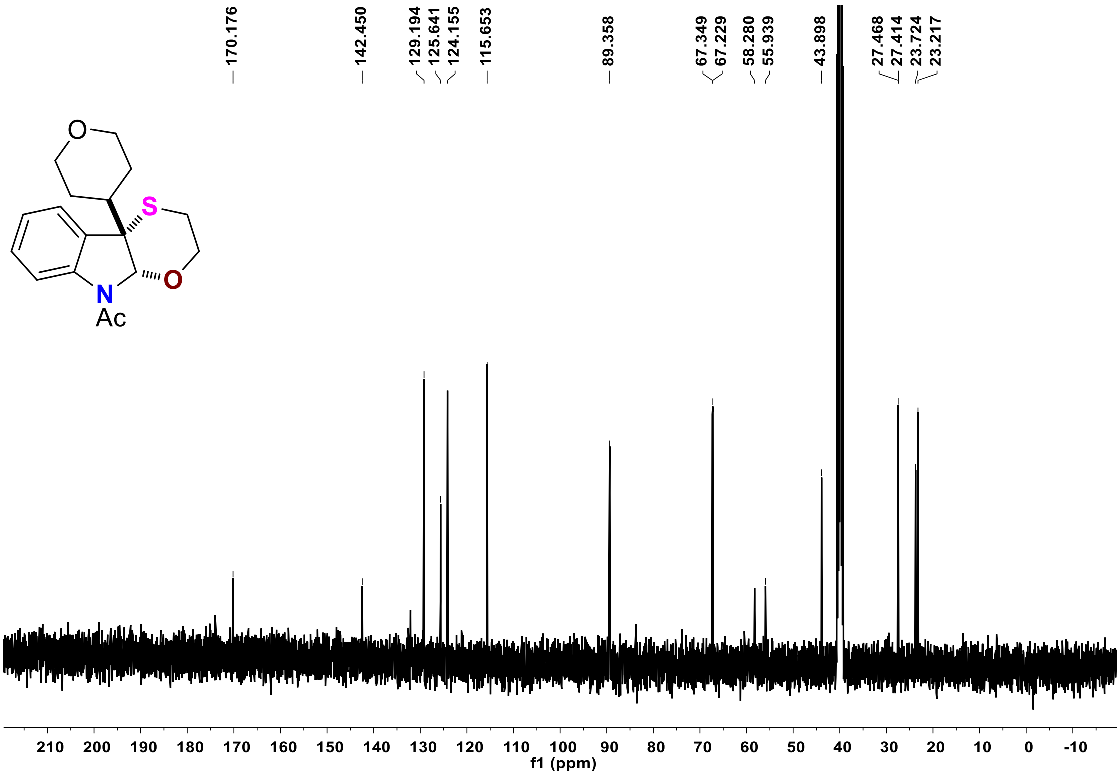

Supplementary Figure 84. ^{13}**C NMR (101 MHz, DMSO-d$_6$) spectrum of 3ta**

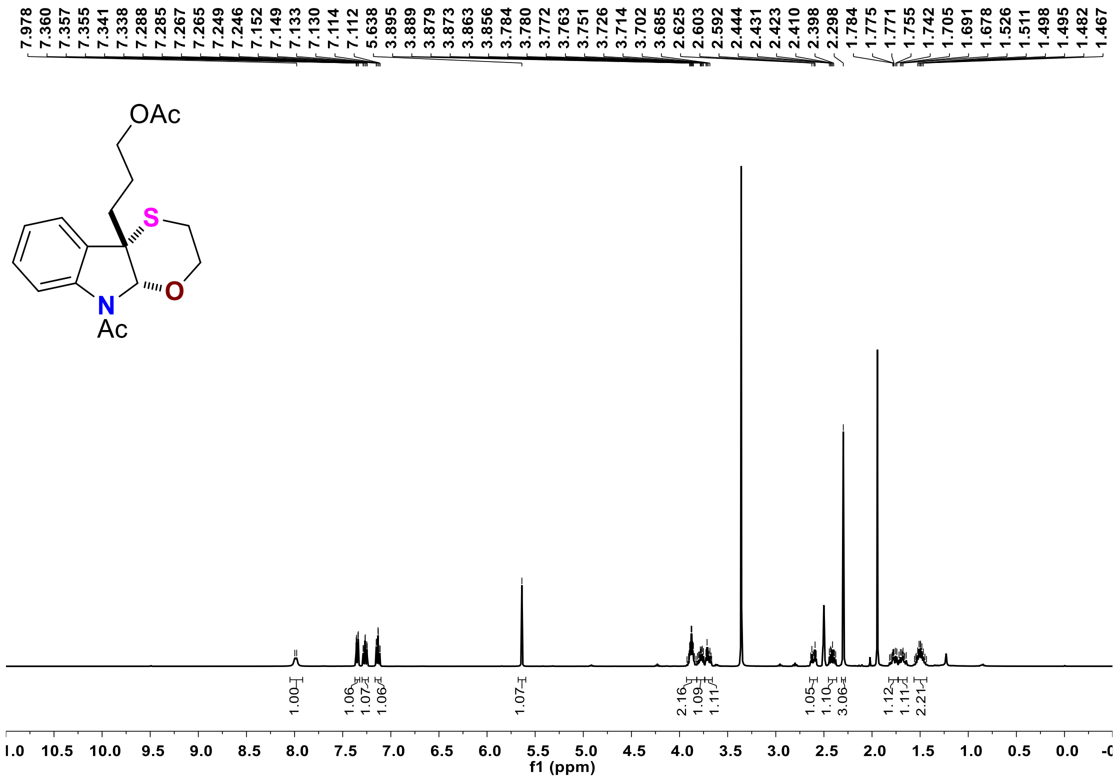

Supplementary Figure 85. ^{1}H **NMR (400 MHz, DMSO-d$_6$) spectrum of 3ua**

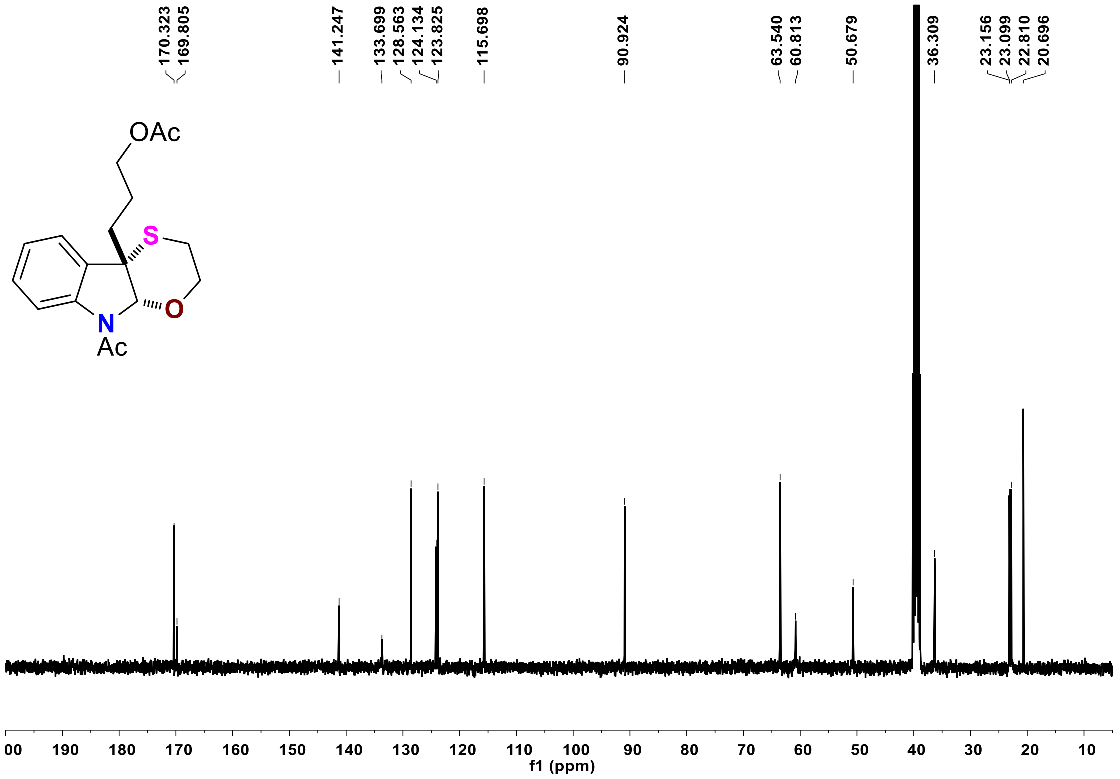

Supplementary Figure 86. ^{13}C **NMR (101 MHz, DMSO-d$_6$) spectrum of 3ua**

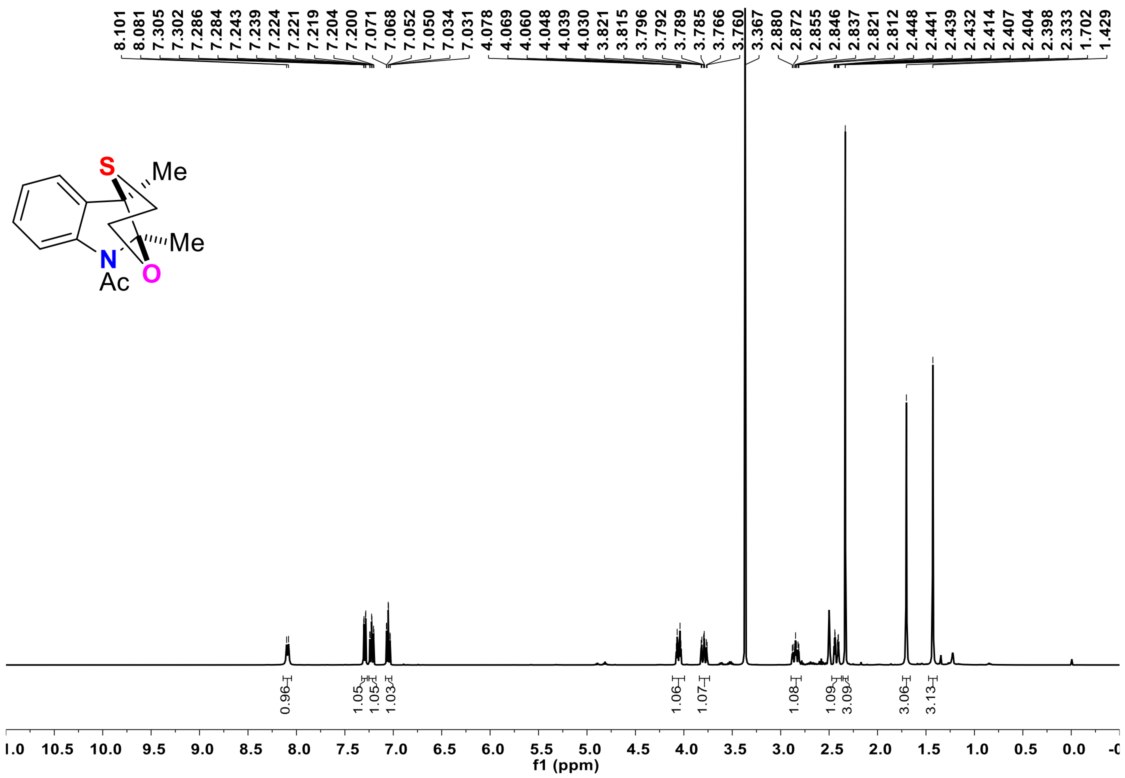

Supplementary Figure 87. ^{1}**H NMR (400 MHz, DMSO-d$_6$) spectrum of 3va**

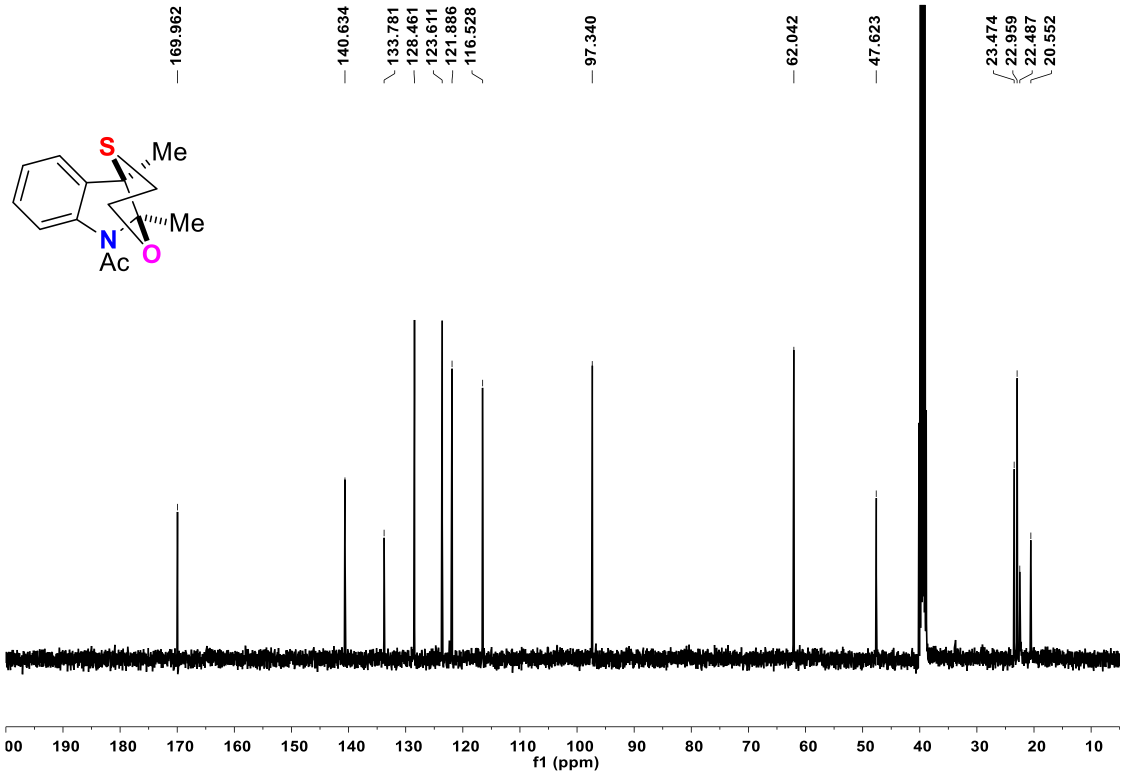

Supplementary Figure 88. ^{13}**C NMR (101 MHz, DMSO-d$_6$) spectrum of 3va**

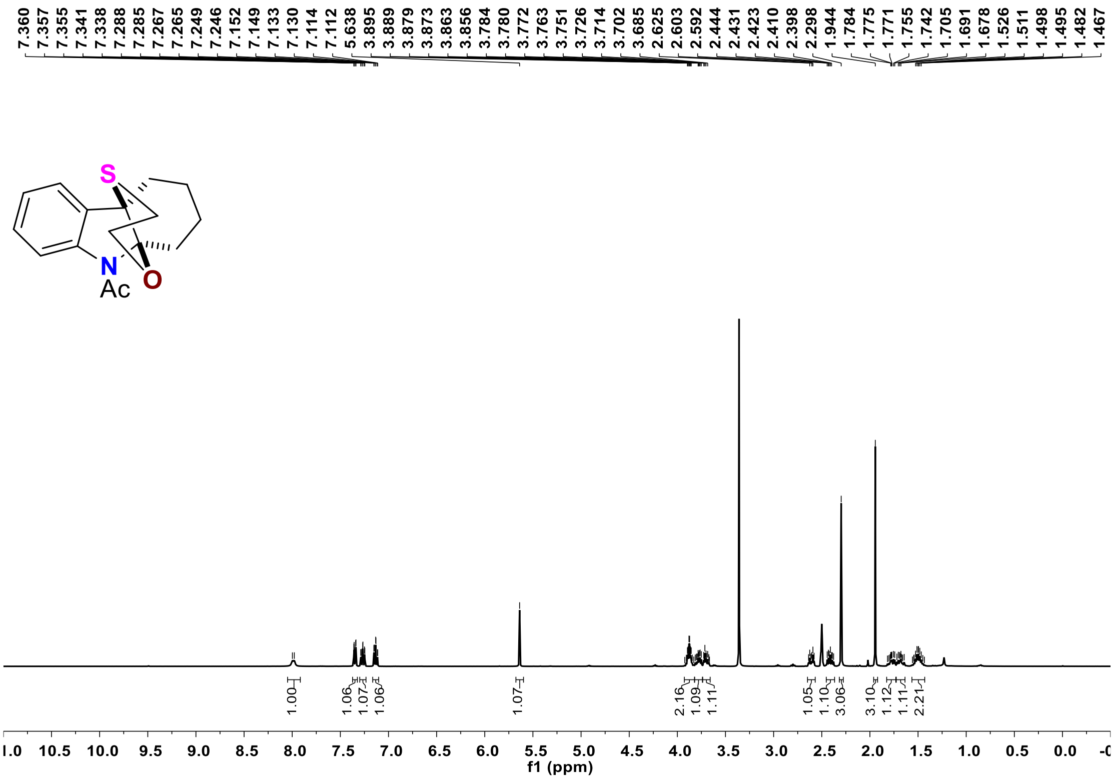

Supplementary Figure 89. ^{1}H NMR (400 MHz, DMSO-d$_6$) spectrum of 3wa

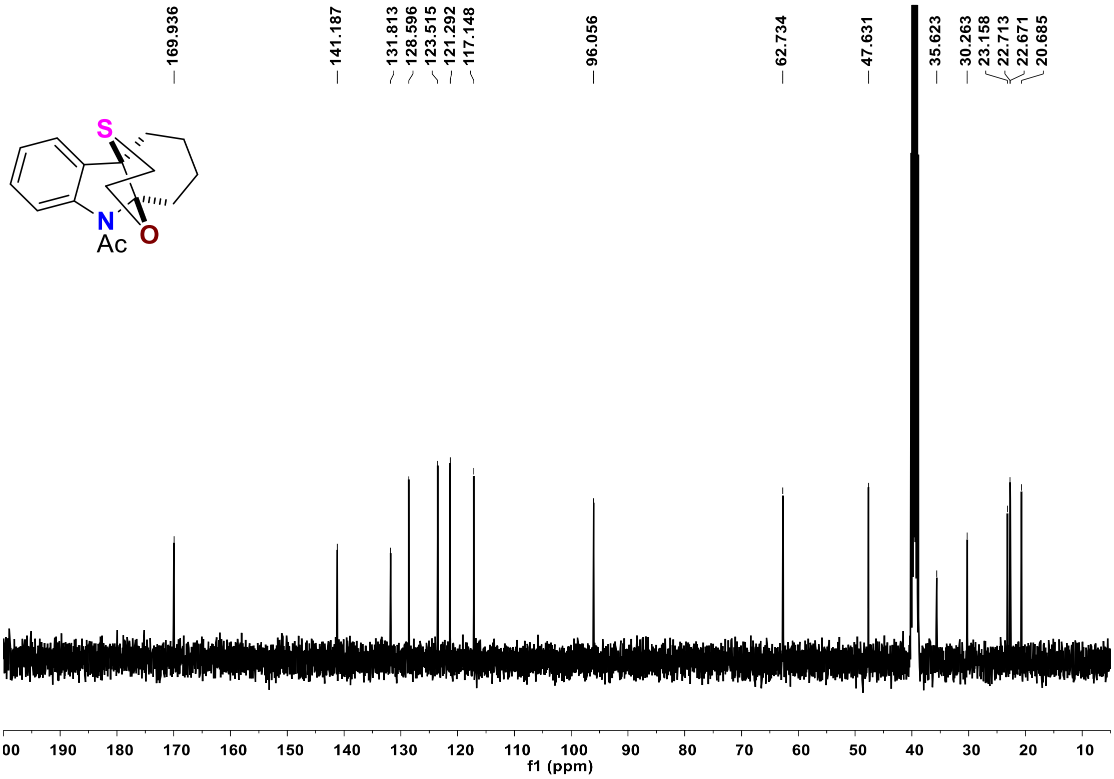

Supplementary Figure 90. ^{13}C NMR (101 MHz, DMSO-d$_6$) spectrum of 3wa

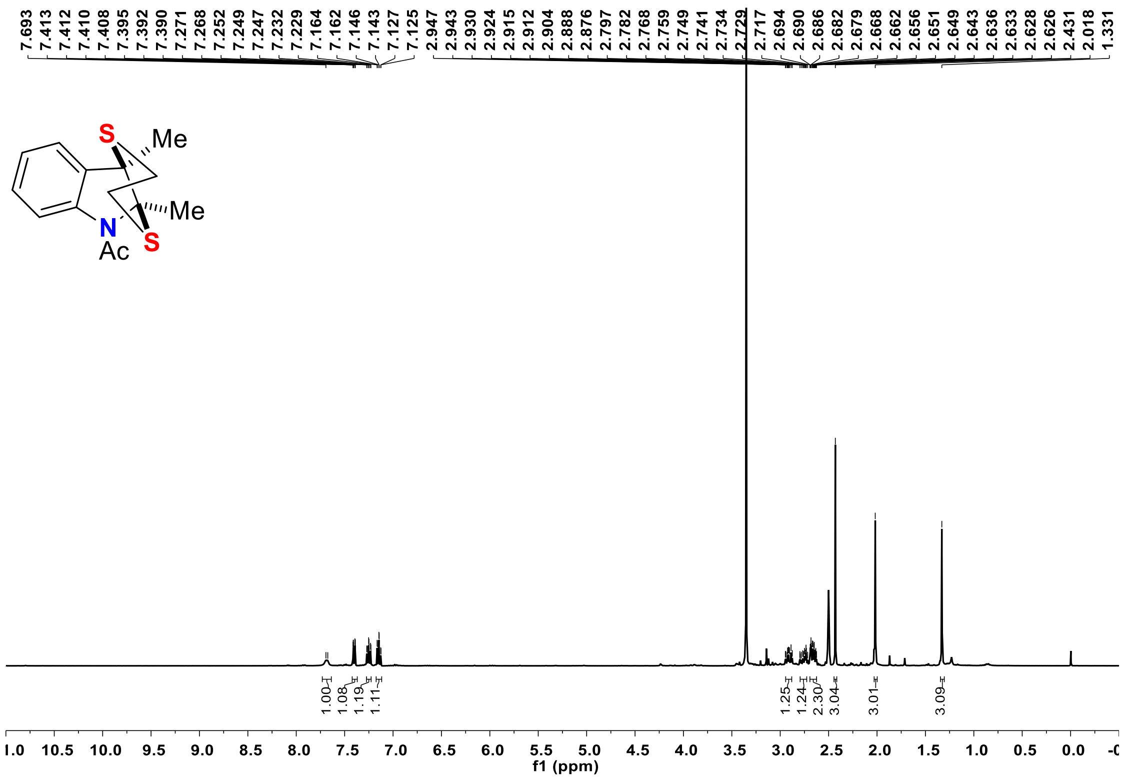

Supplementary Figure 91. ^{1}H NMR (400 MHz, DMSO-d$_6$) spectrum of 3xa

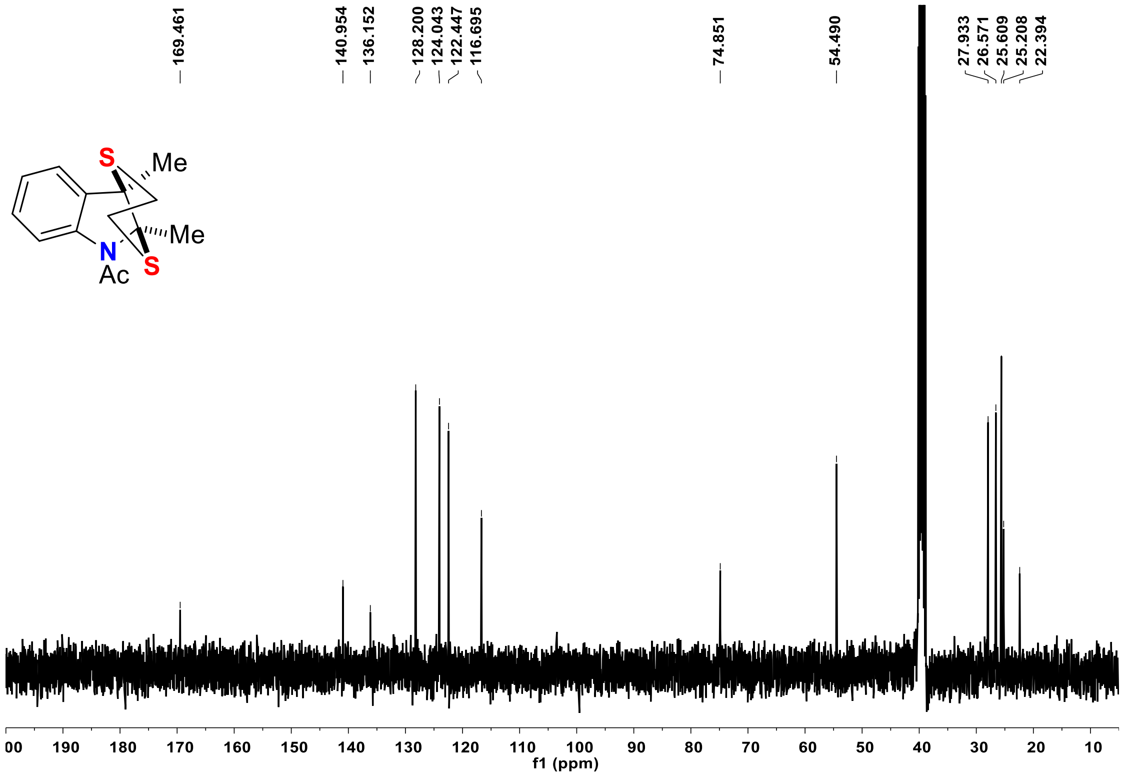

Supplementary Figure 92. ^{13}C NMR (101 MHz, DMSO-d$_6$) spectrum of 3xa

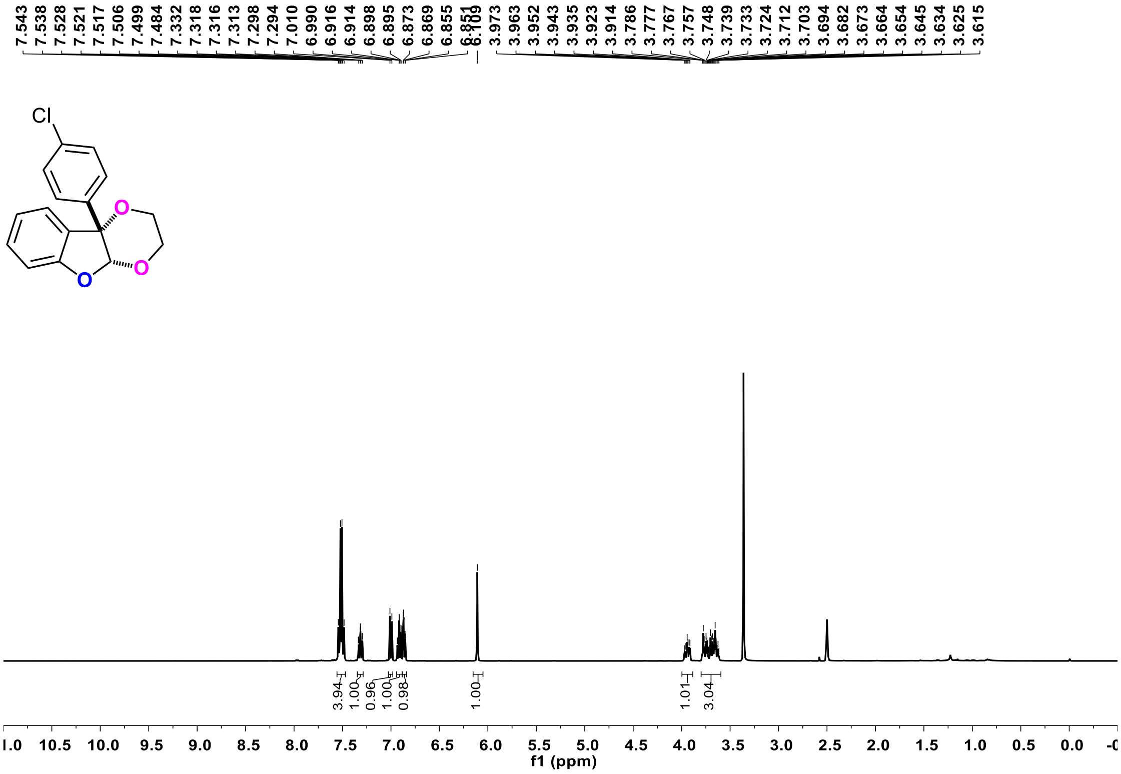

Supplementary Figure 93. ^{1}H **NMR (400 MHz, DMSO-d_6) spectrum of 5a**

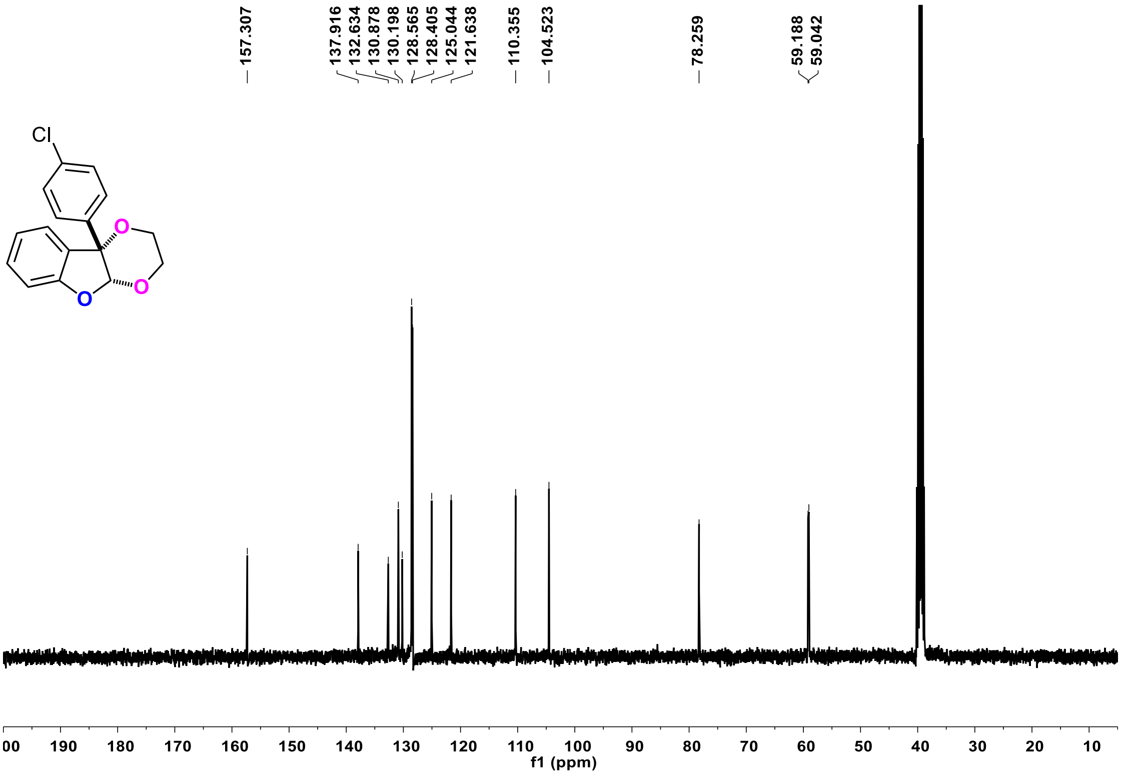

Supplementary Figure 94. ^{13}C **NMR (101 MHz, DMSO-d_6) spectrum of 5a**

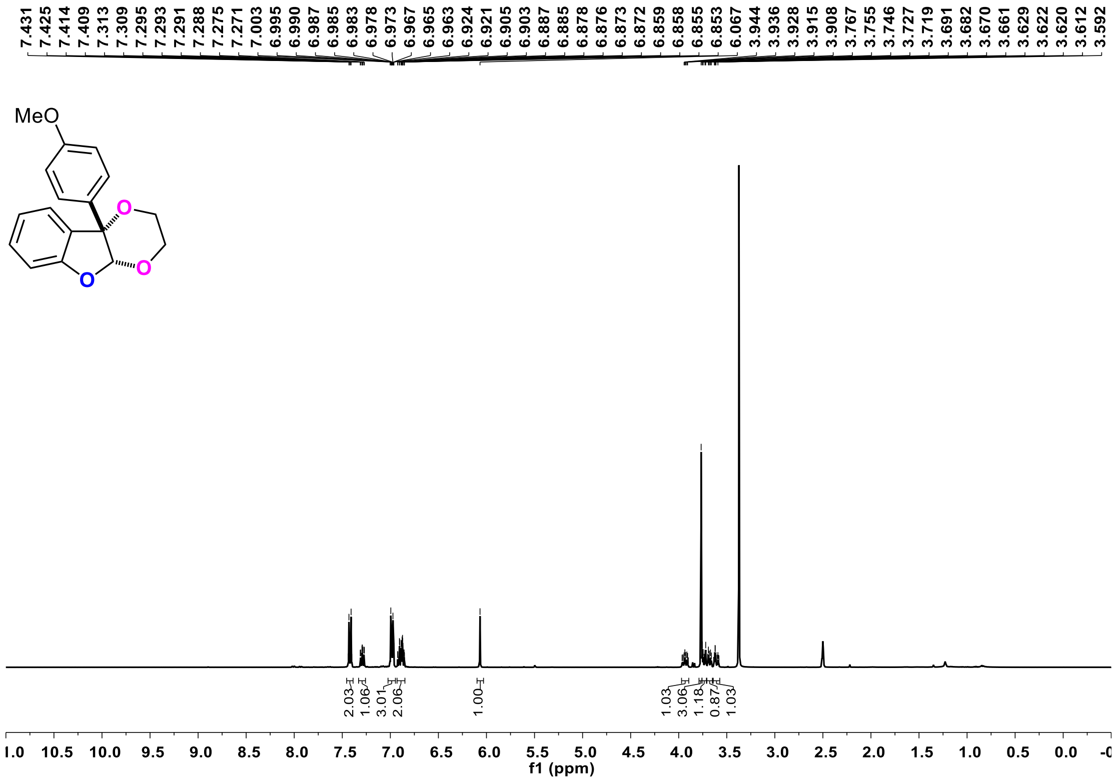

Supplementary Figure 95. ^{1}H NMR (400 MHz, DMSO-d_6) spectrum of 5b

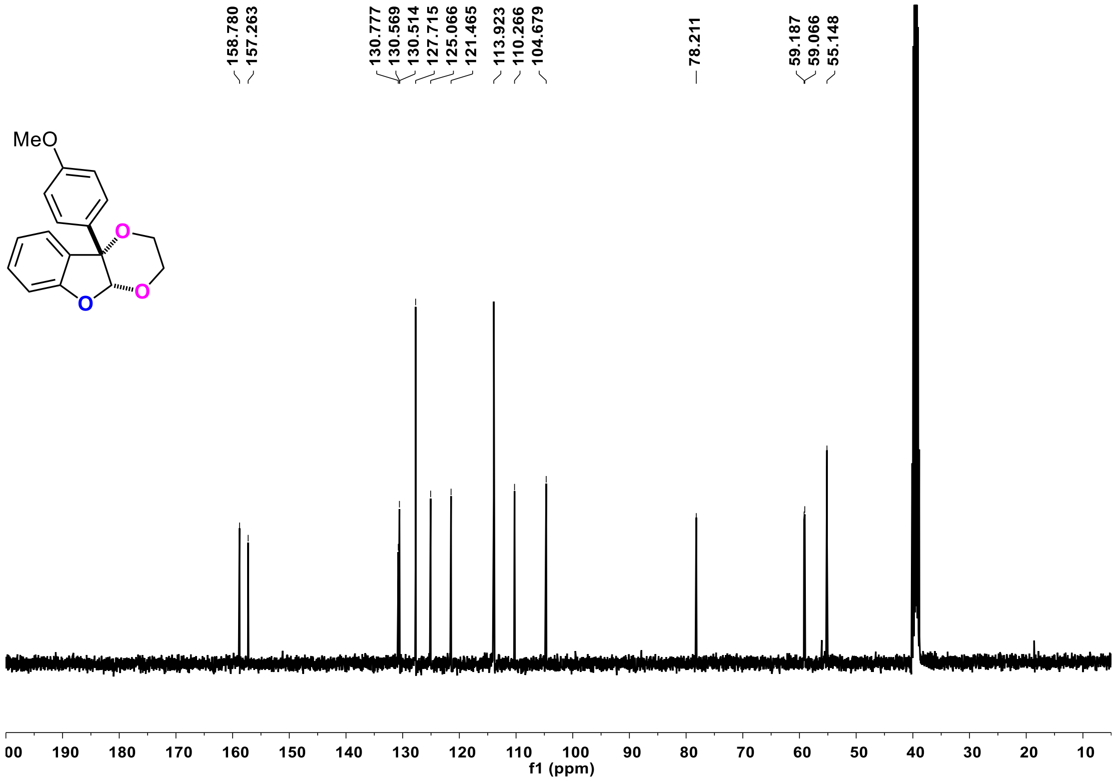

Supplementary Figure 96. ^{13}C NMR (101 MHz, DMSO-d_6) spectrum of 5b

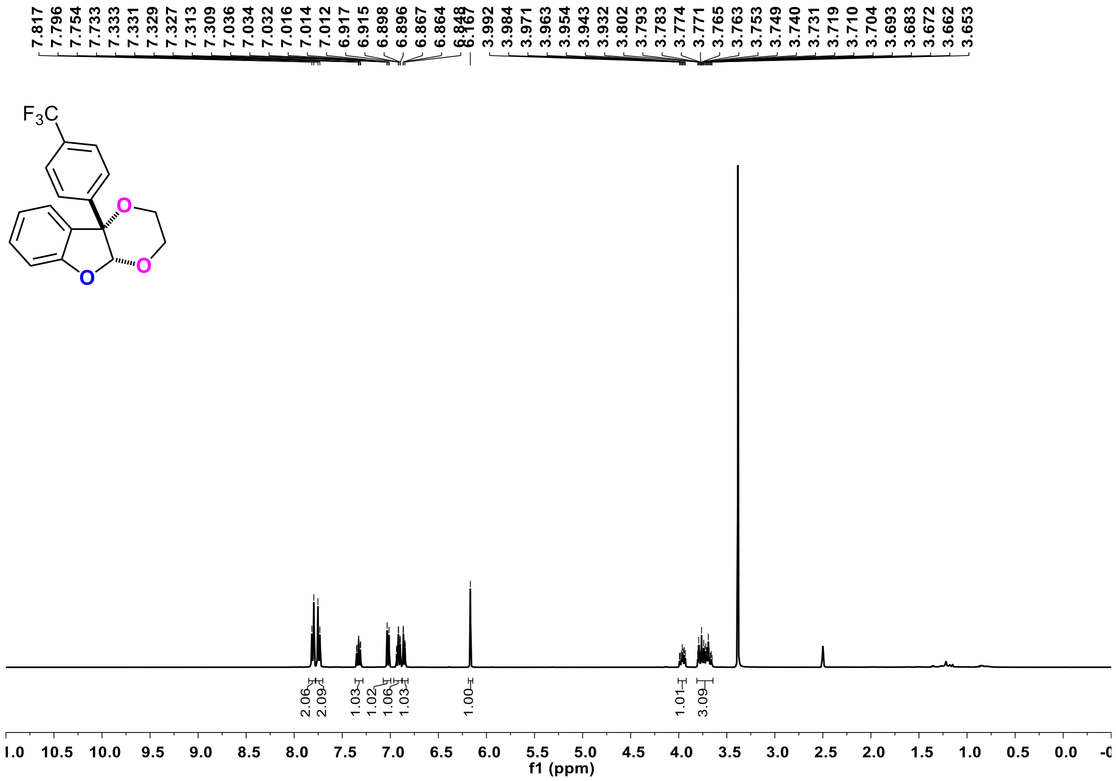

Supplementary Figure 97. ^{1}**H NMR (400 MHz, DMSO-d$_6$) spectrum of 5c**

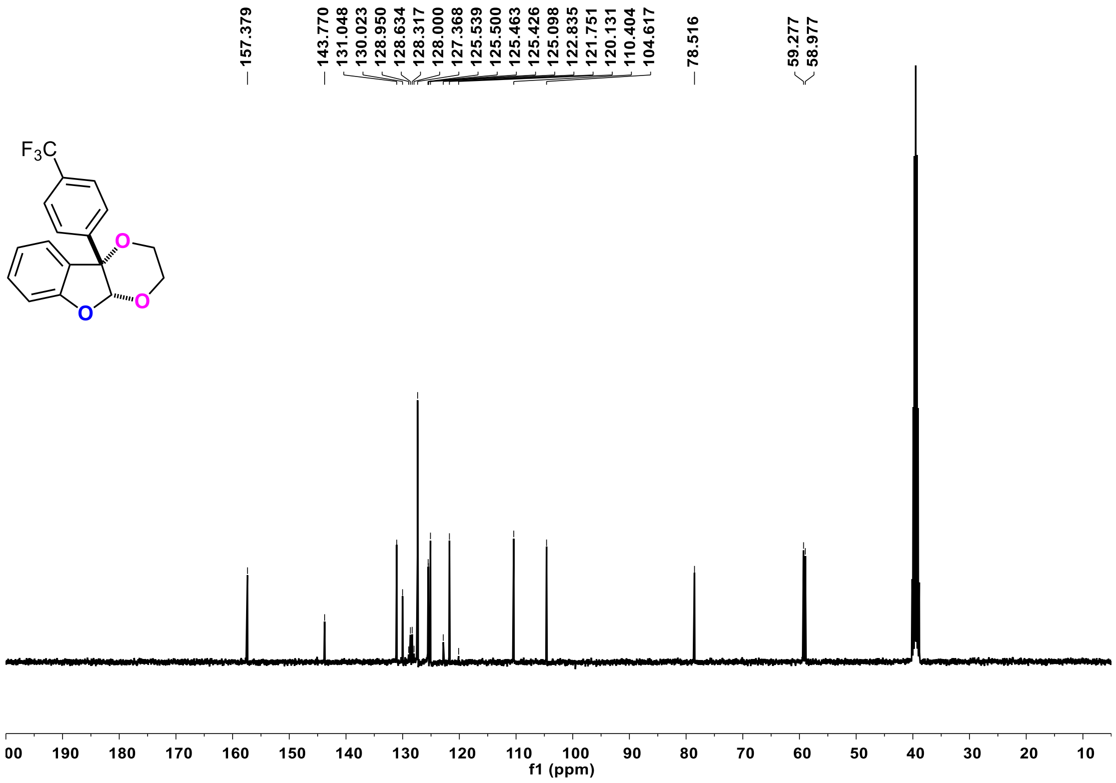

Supplementary Figure 98. ^{13}**C NMR (101 MHz, DMSO-d$_6$) spectrum of 5c**

^{19}F NMR

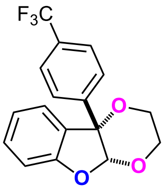

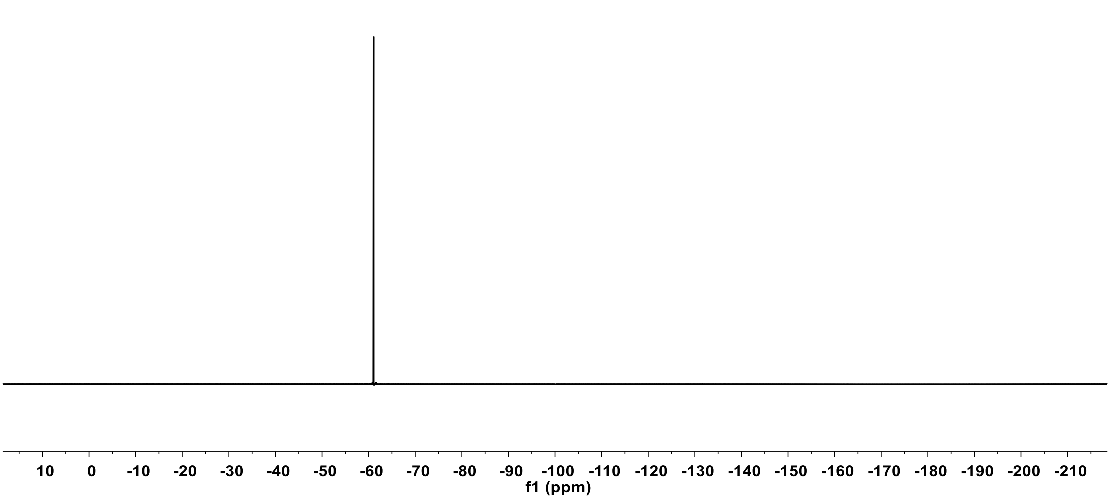

Supplementary Figure 99. ^{19}F NMR (377 MHz, DMSO-d_6) spectrum of 5c

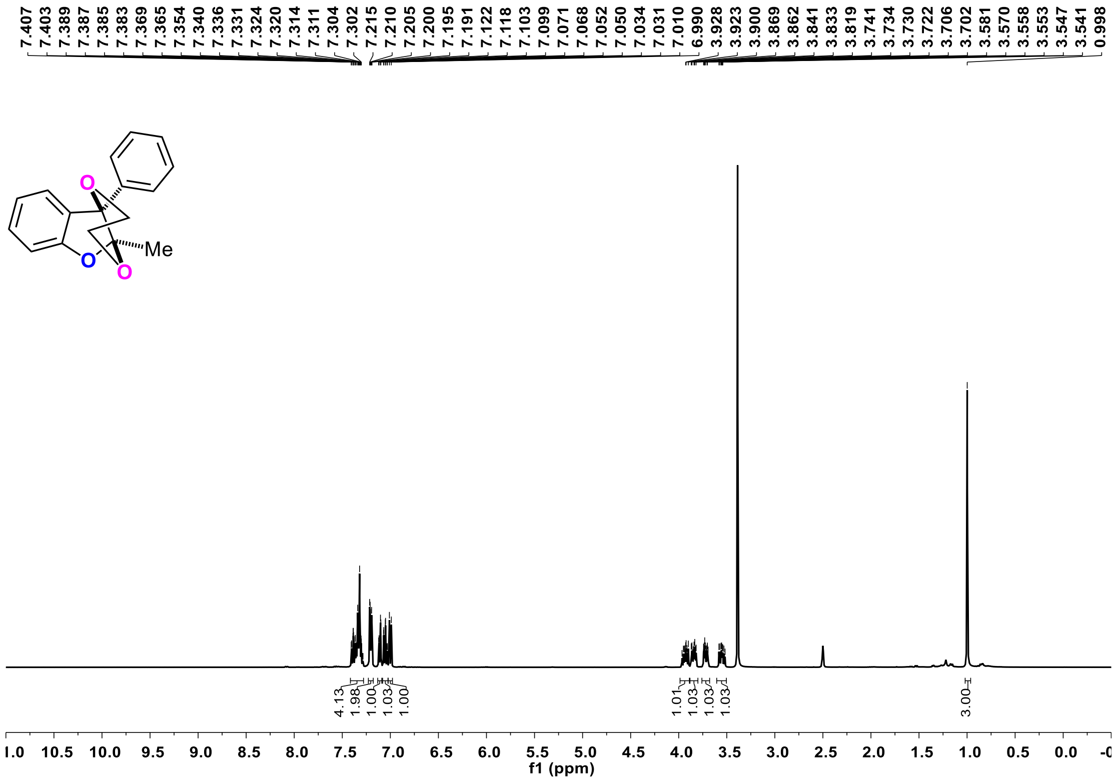

Supplementary Figure 100. ^{1}H NMR (400 MHz, DMSO-d_6) spectrum of 5d

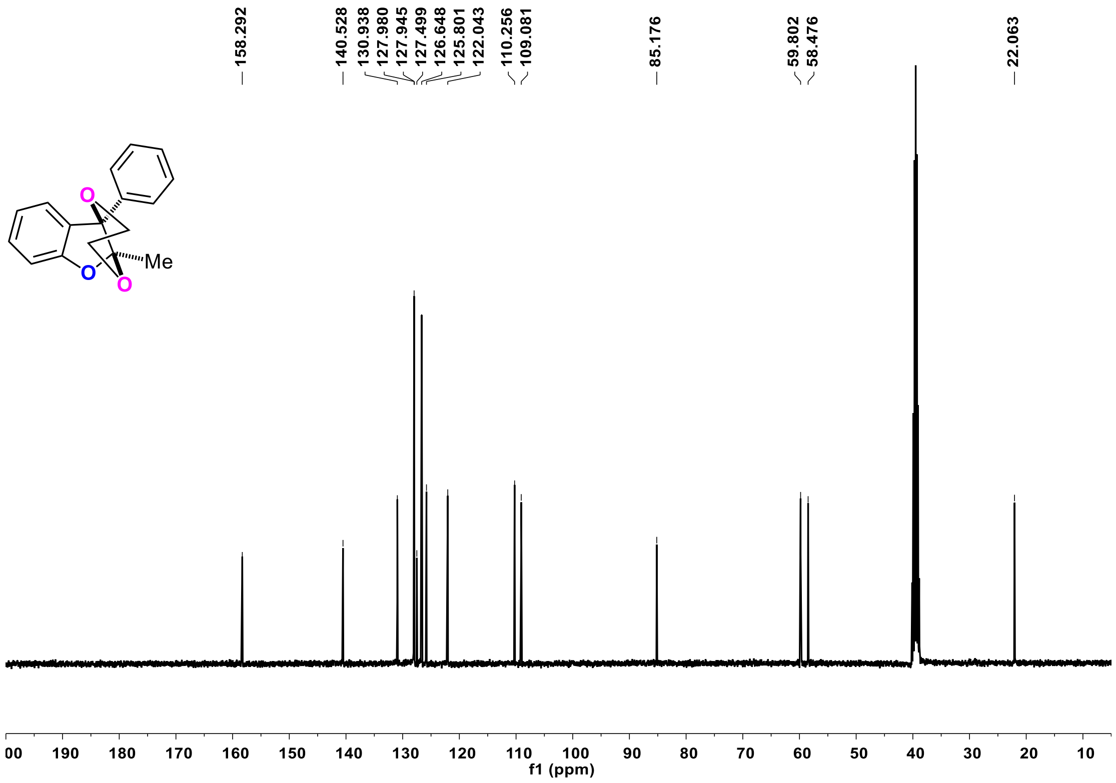

Supplementary Figure 101. ^{13}C NMR (101 MHz, DMSO-d_6) spectrum of 5d

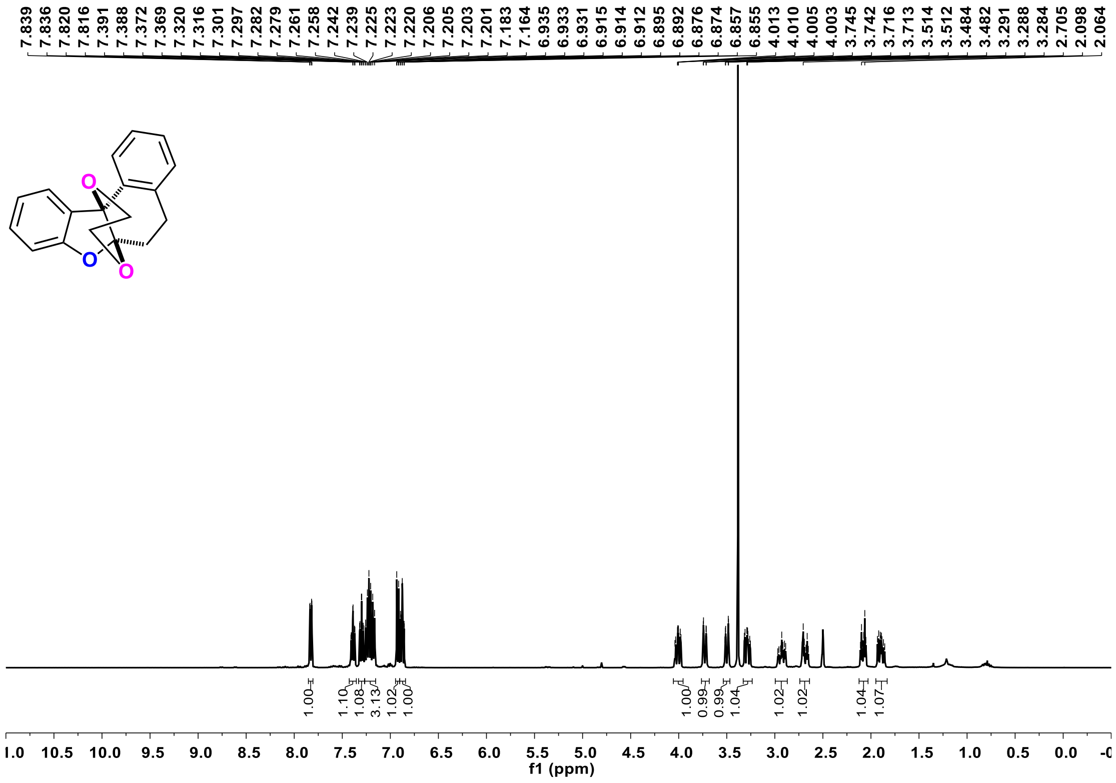

Supplementary Figure 102. **^{1}H NMR (400 MHz, DMSO-d_6) spectrum of 5e**

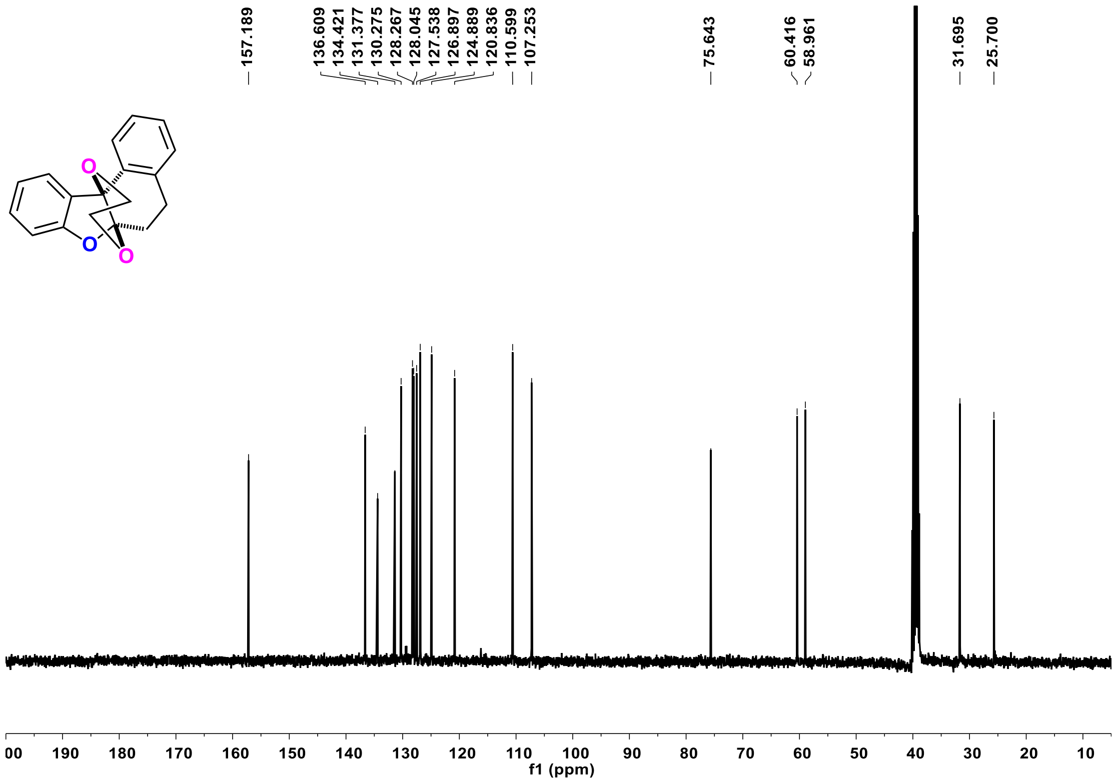

Supplementary Figure 103. **^{13}C NMR (101 MHz, DMSO-d_6) spectrum of 5e**

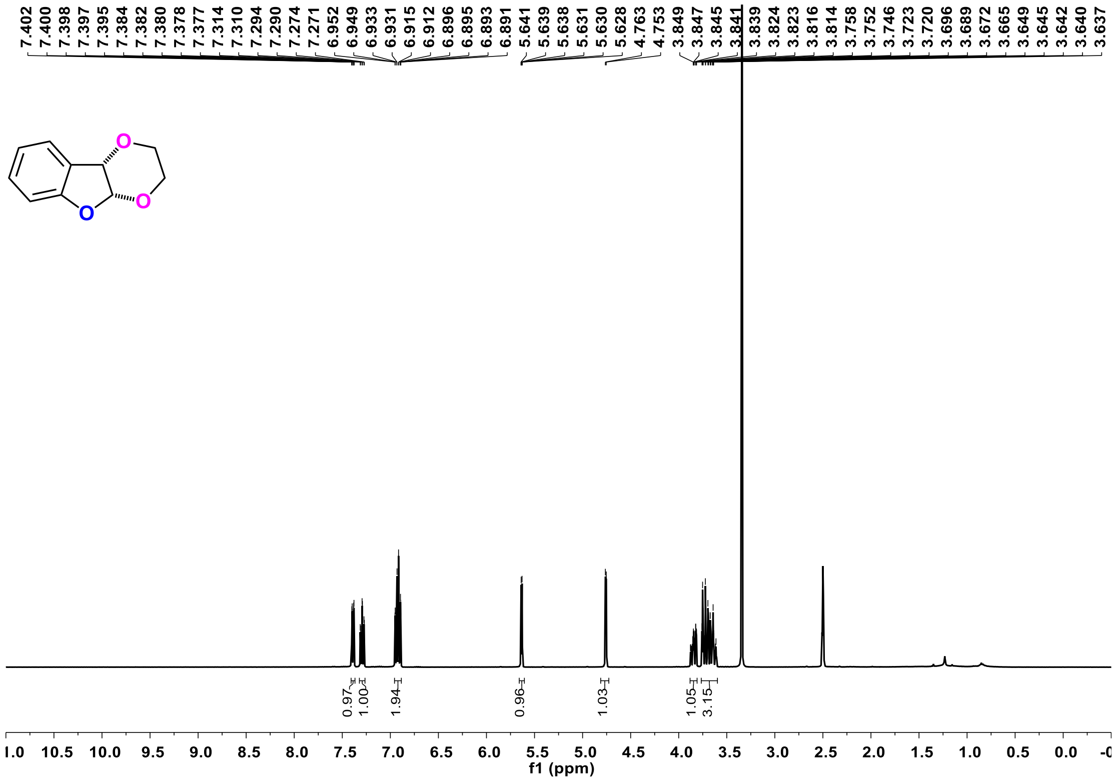

Supplementary Figure 104. ^{1}H NMR (400 MHz, DMSO-d$_6$) spectrum of 5f

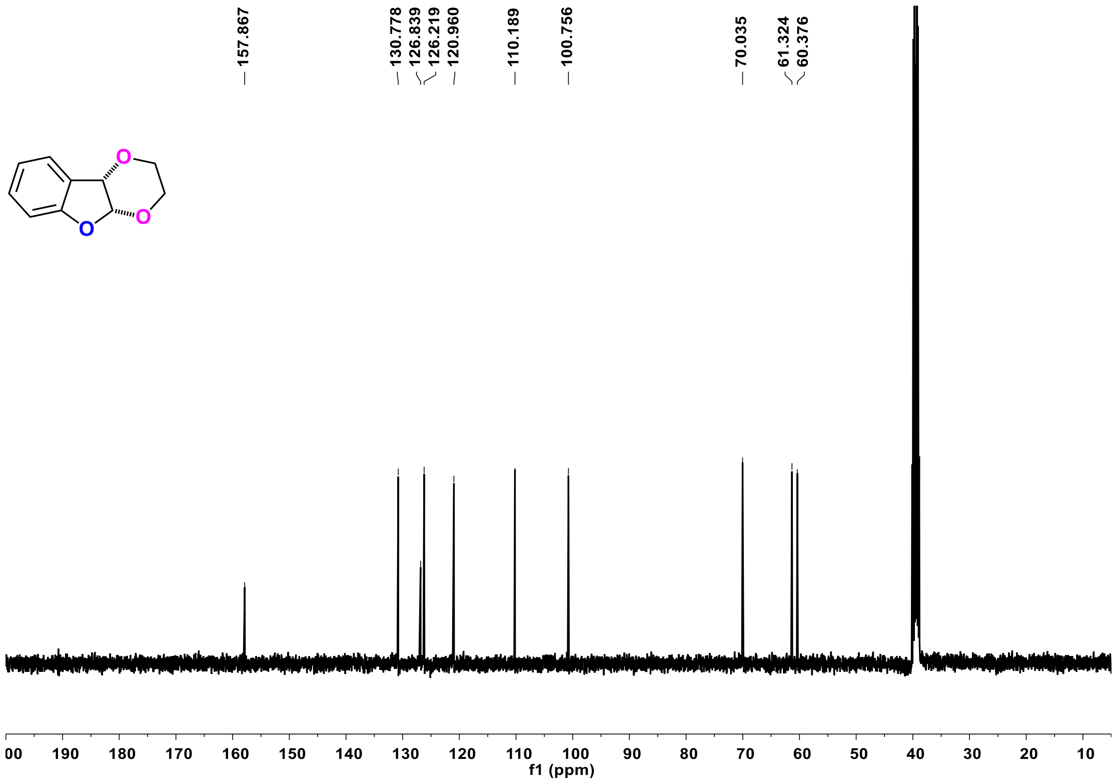

Supplementary Figure 105. ^{13}C NMR (101 MHz, DMSO-d$_6$) spectrum of 5f

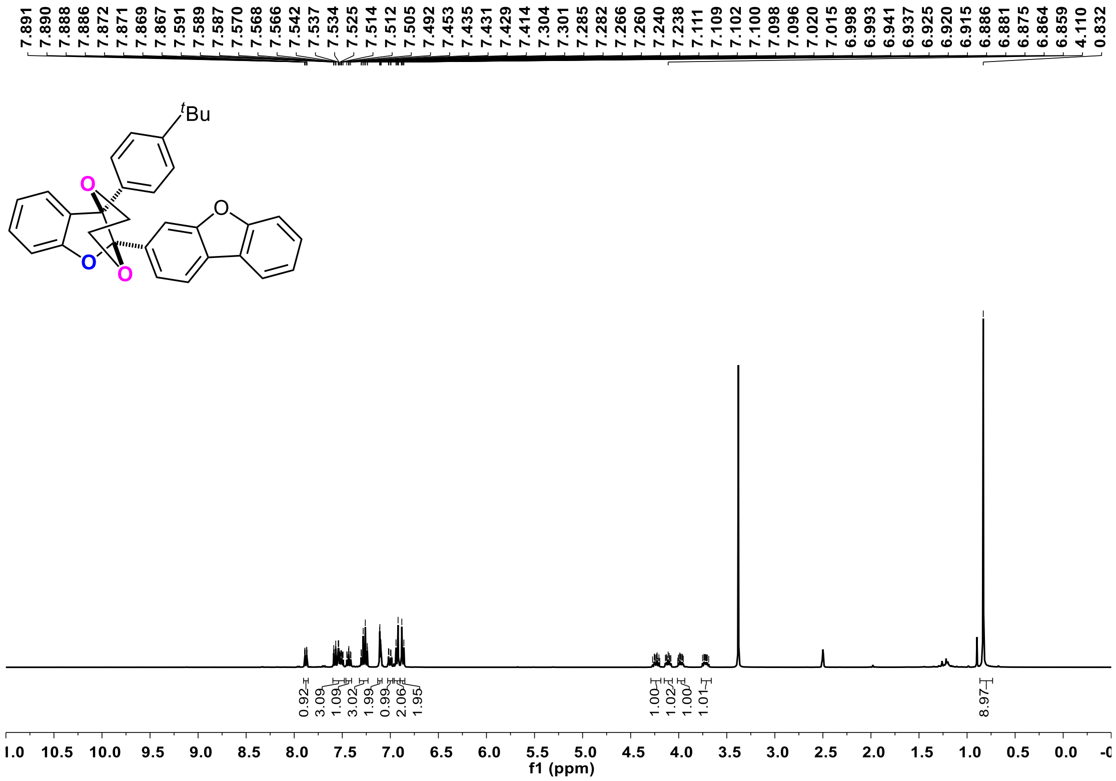

Supplementary Figure 106. ^{1}**H NMR (400 MHz, DMSO-d$_6$) spectrum of 5g**

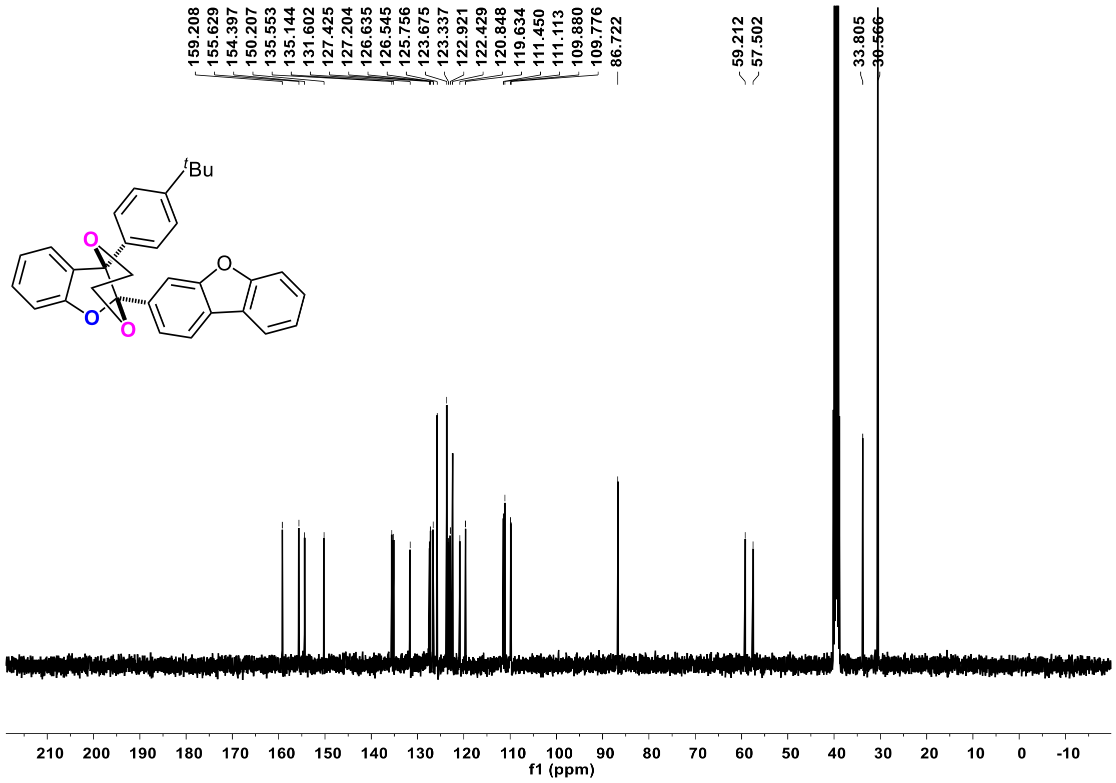

Supplementary Figure 107. ^{13}**C NMR (101 MHz, DMSO-d$_6$) spectrum of 5g**

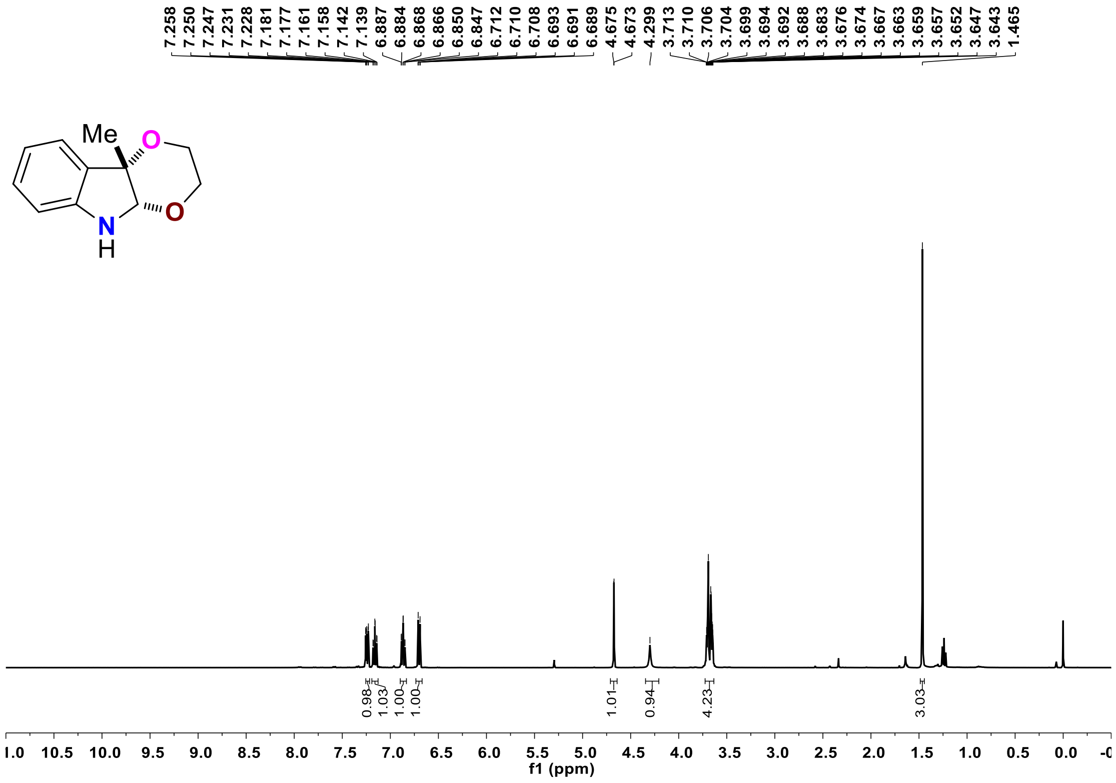

Supplementary Figure 108. ^{1}H NMR (400 MHz, DMSO-d$_6$) spectrum of 6a

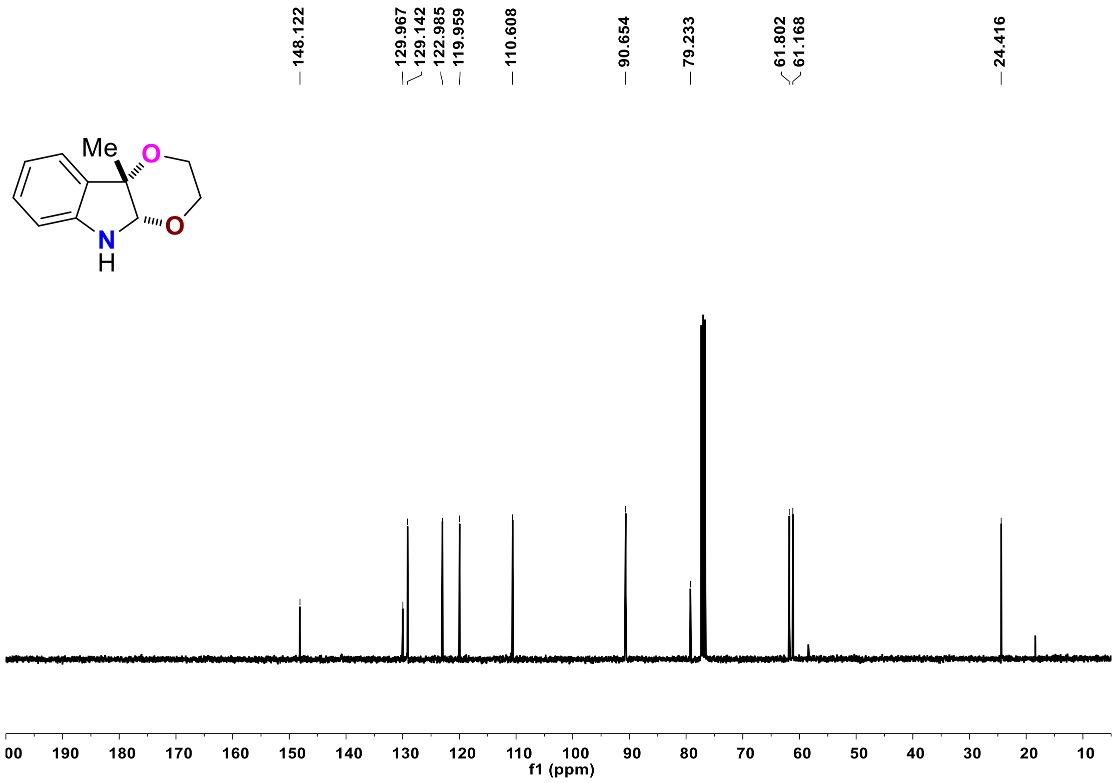

Supplementary Figure 109. ^{13}C NMR (101 MHz, DMSO-d$_6$) spectrum of 6a

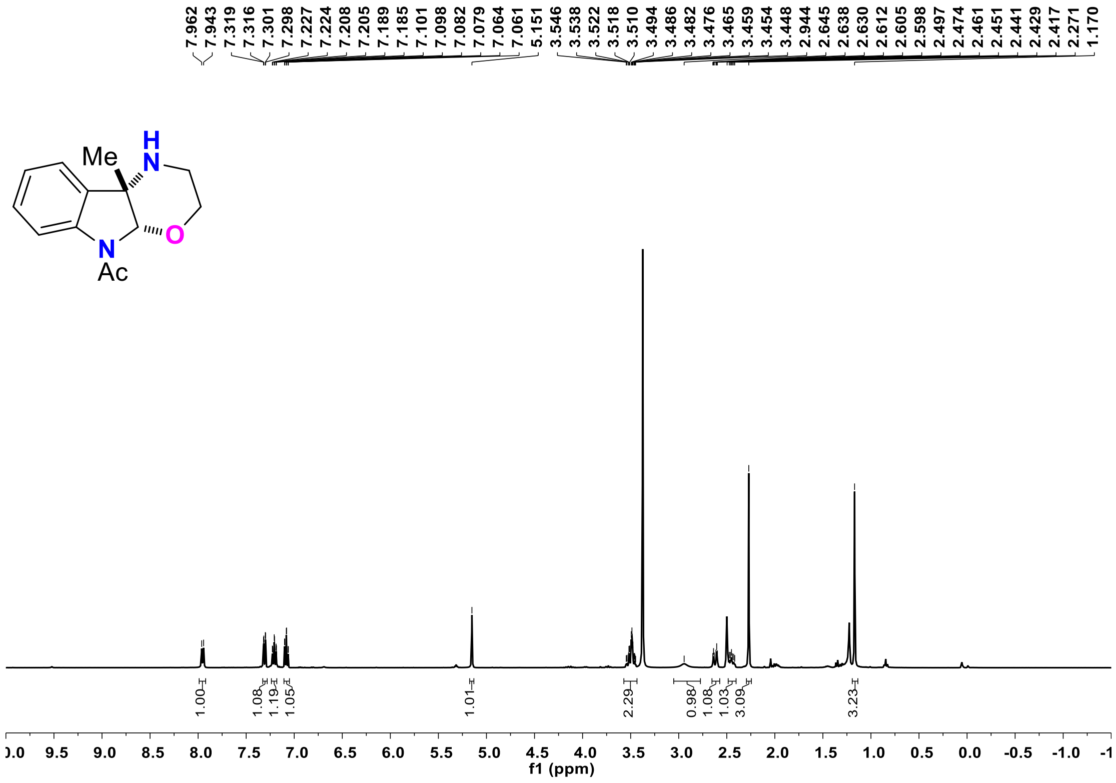

Supplementary Figure 110. ^{1}H NMR (400 MHz, DMSO-d_6) spectrum of 6b

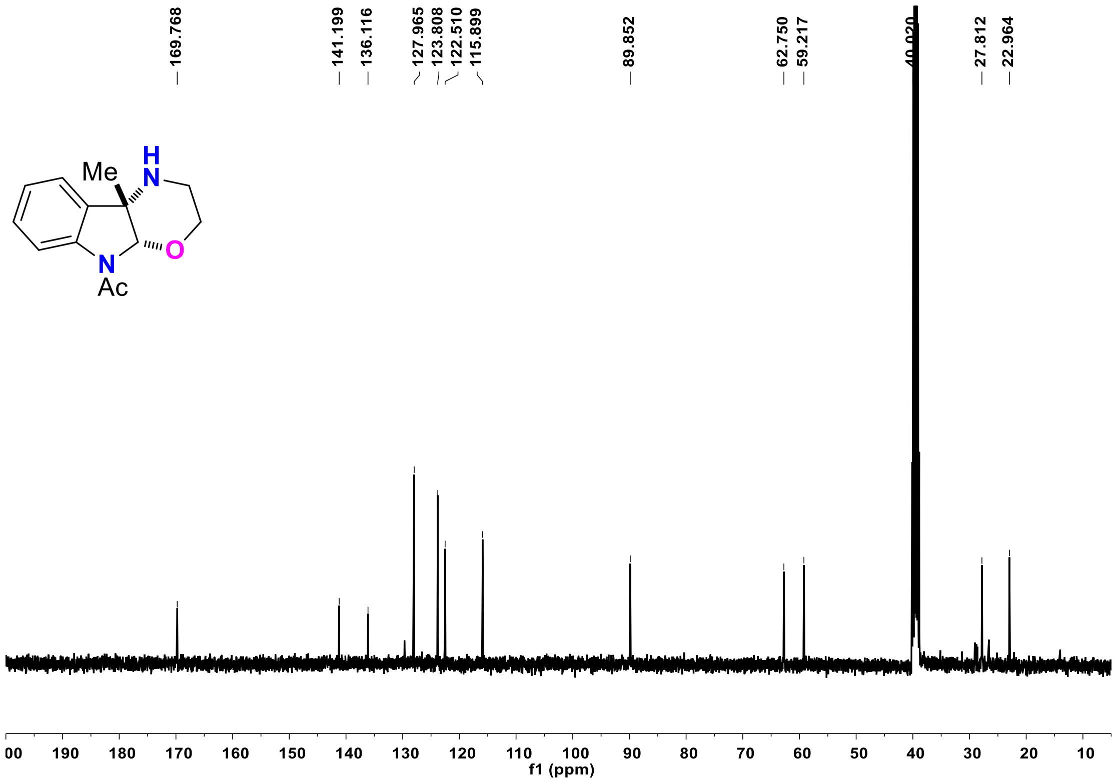

Supplementary Figure 111. ^{13}C NMR (101 MHz, DMSO-d_6) spectrum of 6b

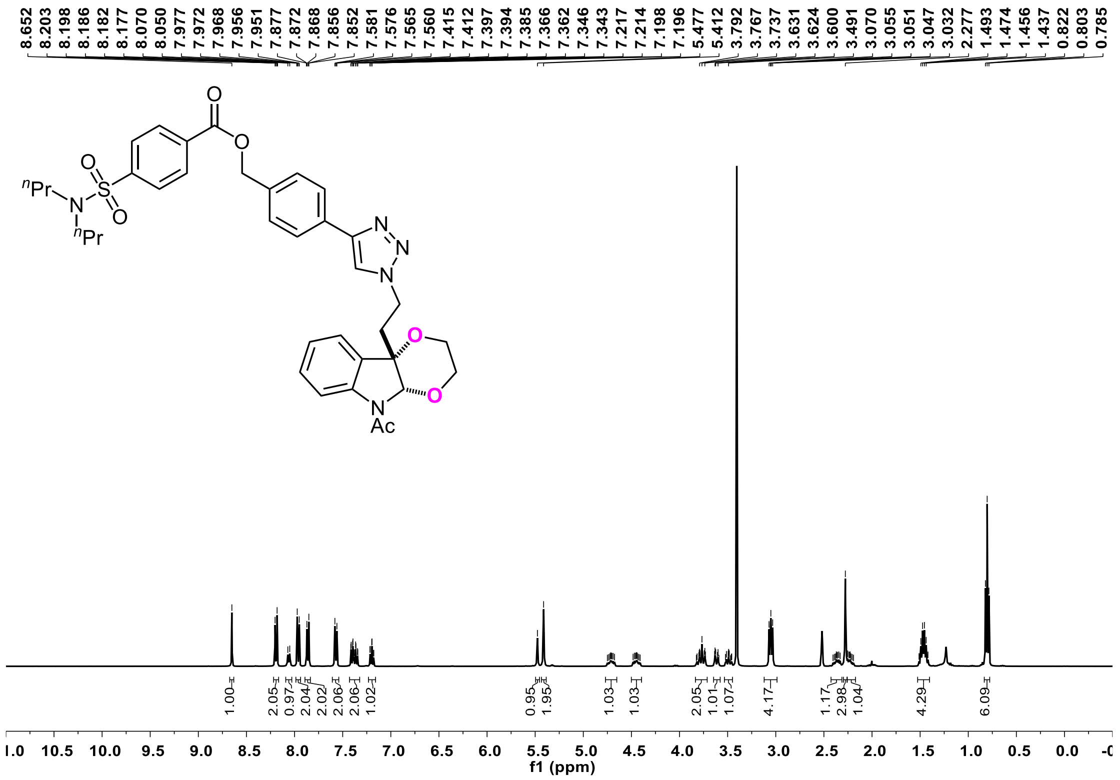

Supplementary Figure 112. ^{1}H NMR (400 MHz, DMSO-d_6) spectrum of 6c

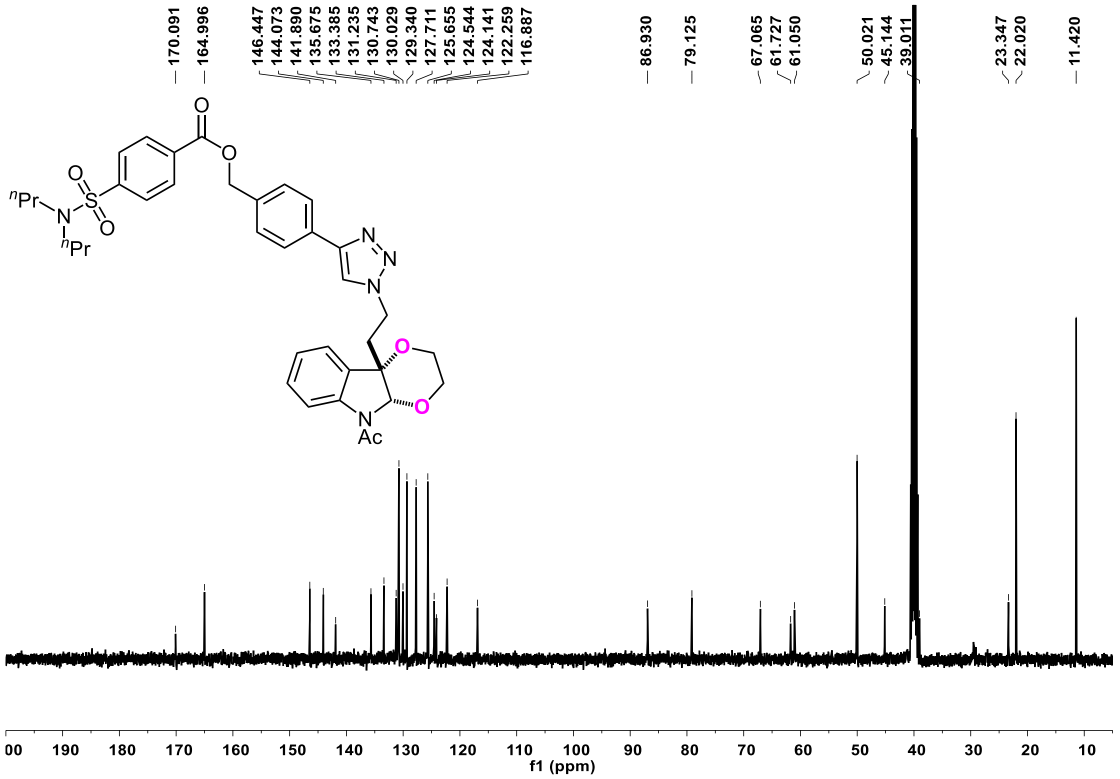

Supplementary Figure 113. ^{13}C NMR (101 MHz, DMSO-d_6) spectrum of 6c

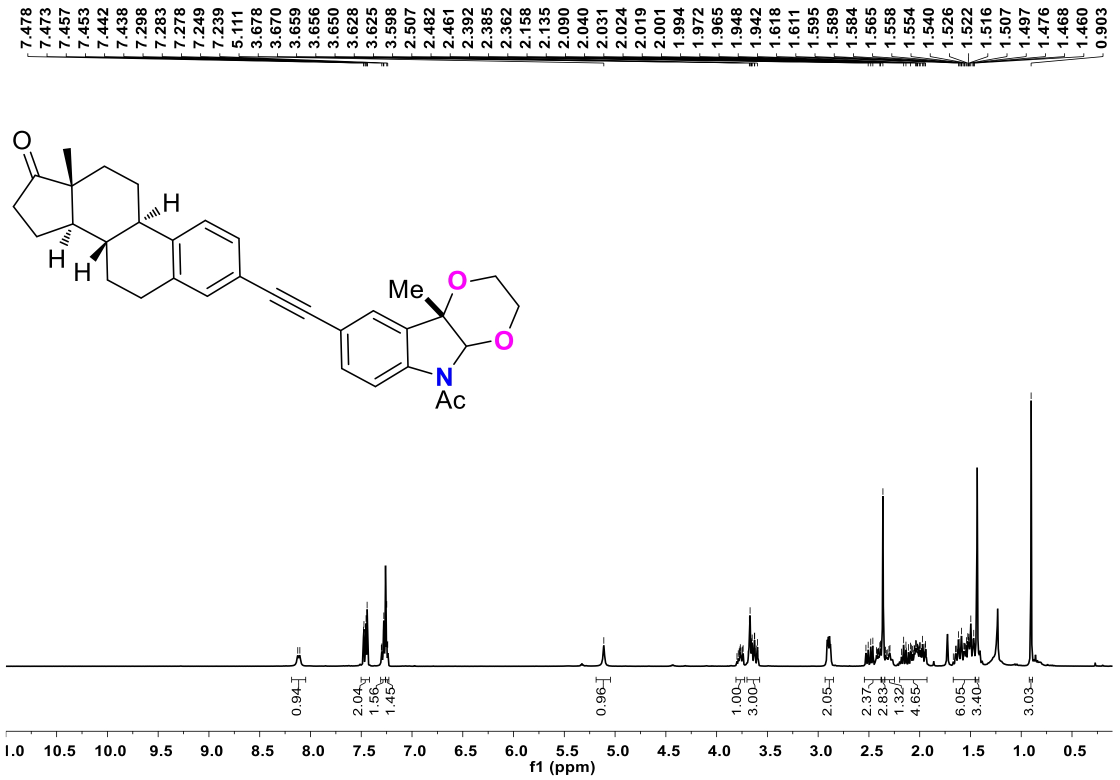

Supplementary Figure 114. **^{1}H NMR (400 MHz, $CDCl_3$) spectrum of 6d**

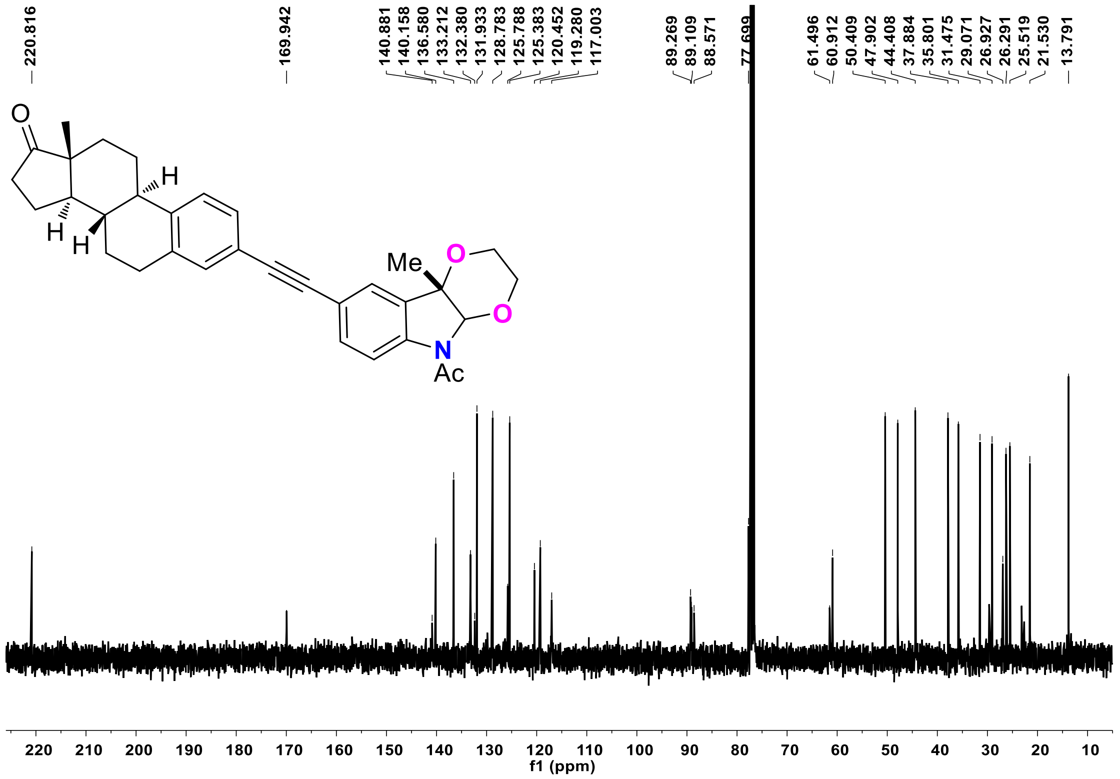

Supplementary Figure 115. **^{13}C NMR (101 MHz, $CDCl_3$) spectrum of 6d**

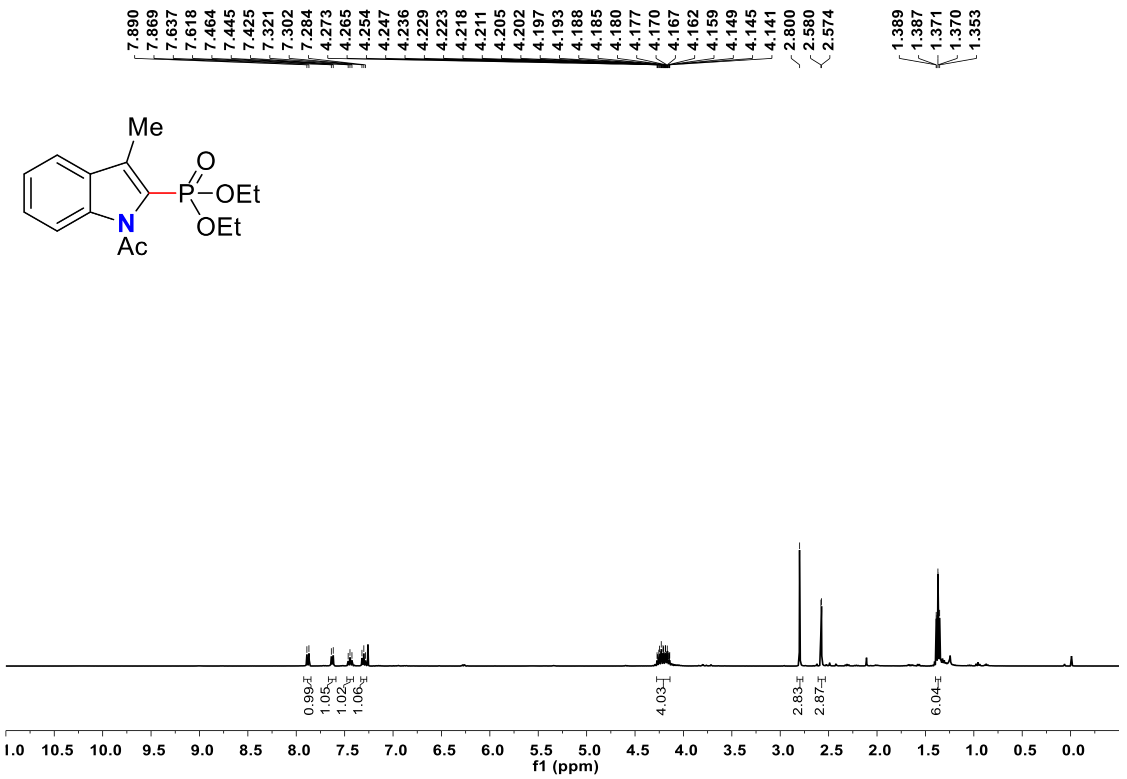

Supplementary Figure 116. ^{1}H **NMR (400 MHz,** $CDCl_3$**) spectrum of 6e**

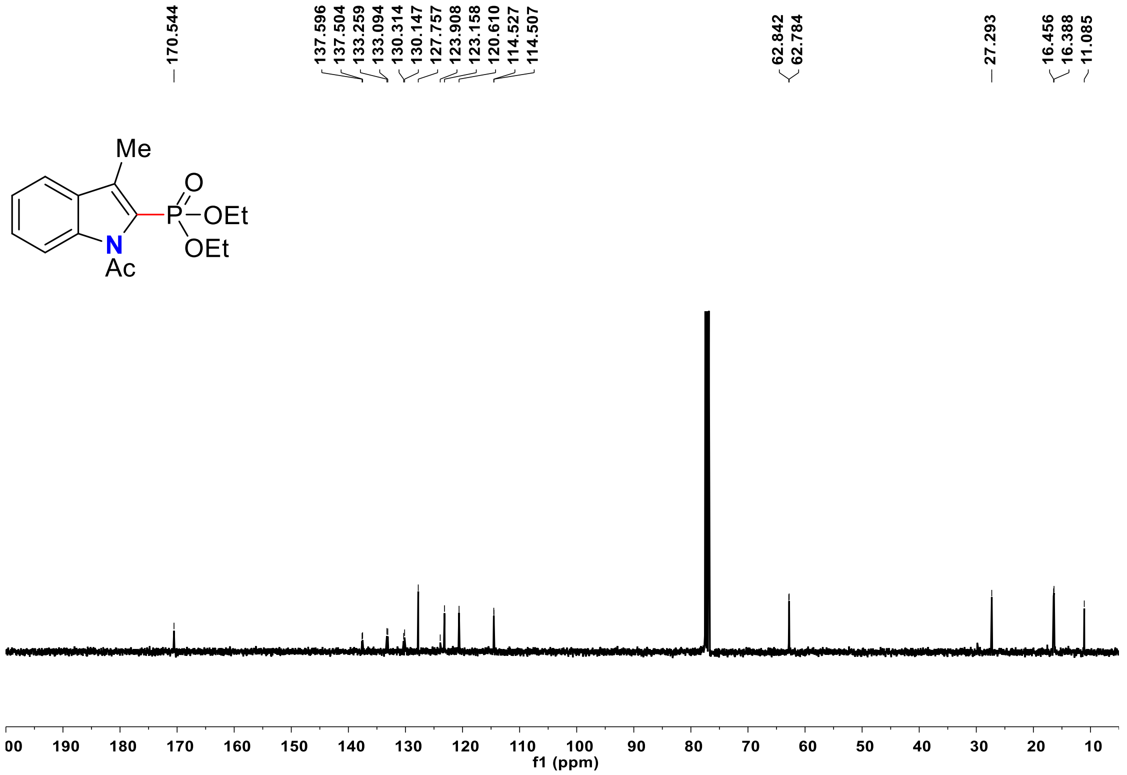

Supplementary Figure 117. ^{13}C **NMR (101 MHz,** $CDCl_3$**) spectrum of 6e**

^{31}P NMR

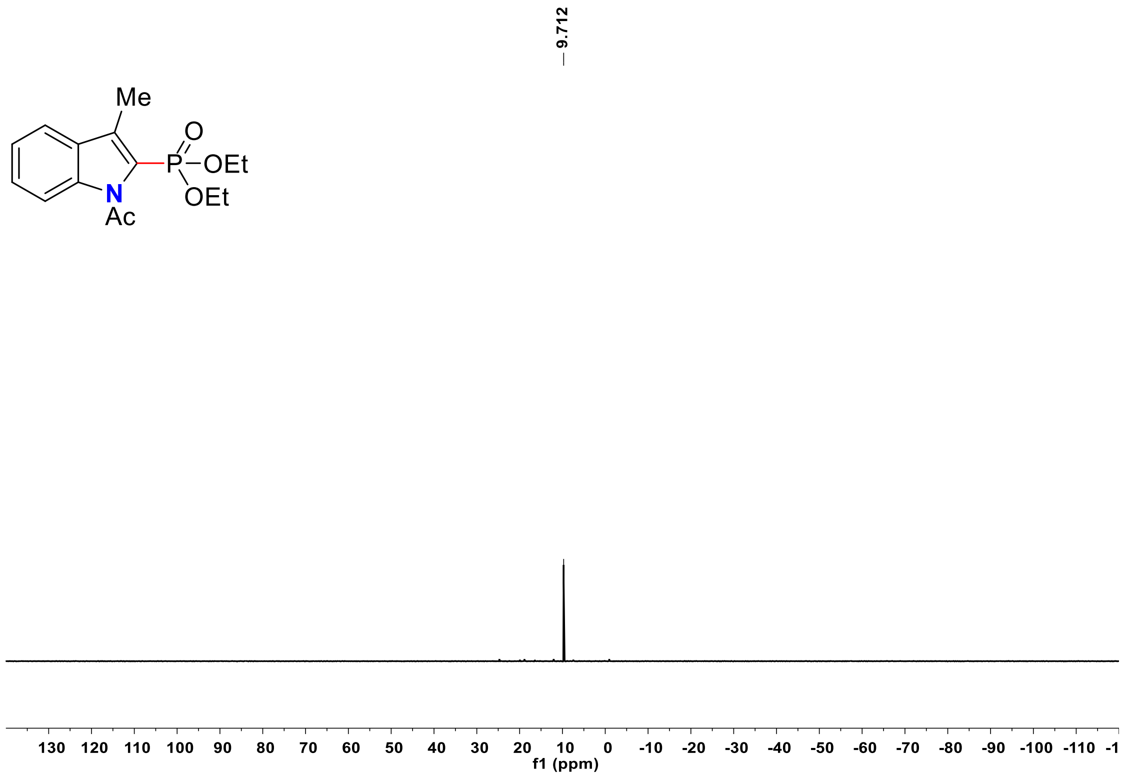

Supplementary Figure 118. ^{13}**C NMR (101 MHz, $CDCl_3$) spectrum of 6e**

Supplementary References

1. Tomakinian T, Guillot R, Kouklovsky C, Vincent G. Direct Oxidative Coupling of N-Acetyl Indoles and Phenols for the Synthesis of Benzofuroindolines Related to Phalarine. *Angew Chem Int Ed.* **2014**, *53*, 11881-11885.
2. Rizzo J.R., Alt C.A., Zhang T.Y.，An expedient synthesis of 3-substituted indoles via reductive alkylation with ketone, *Tetrahedron. Lett.* **2008**, *49*, 6749-6751.
3. Yadav J. S., Subba Reddy B. V., Raju A., Ravindar K., Baishya G., 1-(Chloromethyl)-4-fluoro-1,4-diazoniabicyclo-[2,2,2]octane Bis(tetrafluoroborate) as Novel and Efficient Reagent for the Conjugate Addition of Indoles to α,β-Unsaturated Ketones, *Chem. Lett.* **2007**, *36*, 1056 – 1057.
4. Kraus, G. A.; Schroeder, J. D.; Halogen-Metal Exchange/Cyclization of Iodoketones: A Direct Synthesis of 3-Arylbenzofurans, *Synlett* **2005**, *16*, 2504-2506.
